# Decarboxylative tandem C-N coupling with nitroarenes via S_H_2 mechanism

**DOI:** 10.1038/s41467-022-30176-z

**Published:** 2022-05-04

**Authors:** Shuaishuai Wang, Tingrui Li, Chengyihan Gu, Jie Han, Chuan-Gang Zhao, Chengjian Zhu, Hairen Tan, Jin Xie

**Affiliations:** 1 State Key Laboratory of Coordination Chemistry, Jiangsu Key Laboratory of Advanced Organic Materials, Chemistry and Biomedicine Innovation Center (ChemBIC), School of Chemistry and Chemical Engineering, Nanjing University, 210023 Nanjing, China; 2 State Key Laboratory of Organometallic Chemistry, Shanghai Institute of Organic Chemistry, 200032 Shanghai, China; 3 Green Catalysis Center, College of Chemistry and Molecular Engineering, Zhengzhou University, 450001 Zhengzhou, China; 4 National Laboratory of Solid-State Microstructures, Collaborative Innovation Center of Advanced Microstructures, Jiangsu Key Laboratory of Artificial Functional Materials, College of Engineering and Applied Sciences, Nanjing University, 210023 Nanjing, China; 5 Advanced Catalytic Engineering Research Center of the Ministry of Education, Hunan University, 410082 Changsha, China

**Keywords:** Synthetic chemistry methodology, Photocatalysis

## Abstract

Aromatic tertiary amines are one of the most important classes of organic compounds in organic chemistry and drug discovery. It is difficult to efficiently construct tertiary amines from primary amines via classical nucleophilic substitution due to consecutive overalkylation. In this paper, we have developed a radical tandem C-N coupling strategy to efficiently construct aromatic tertiary amines from commercially available carboxylic acids and nitroarenes. A variety of aromatic tertiary amines can be furnished in good yields (up to 98%) with excellent functional group compatibility under mild reaction conditions. The use of two different carboxylic acids also allows for the concise synthesis of nonsymmetric aromatic tertiary amines in satisfactory yields. Mechanistic studies suggest the intermediacy of the arylamine–(TPP)Fe(III) species and might provide a possible evidence for an S_H_2 (bimolecular homolytic substitution) pathway in the critical C-N bond formation step.

## Abstract (web summary)

Aromatic tertiary amines are versatile building blocks in organic synthesis. In this article, the authors report on an iron-catalysed reaction for the decarboxylative C-N coupling from carboxylic acids and nitroarenes, leading to non-symmetric tertiary aromatic amines.

## Introduction

**Fig. 1:**
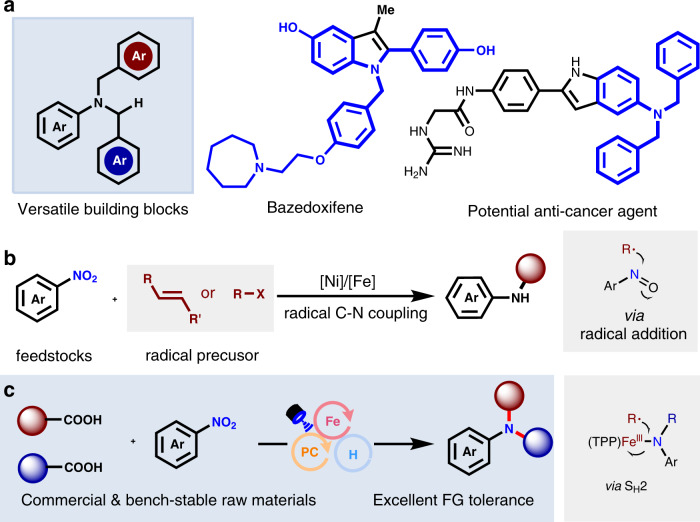
Summary of previous and current studies. **a** The significance of aromatic benzylic tertiary amines. **b** Recent radical progress on catalytic synthesis of secondary amines with cheap nitroarenes. **c** Synergistic catalytic tertiary amines synthesis from commercially abundant feedstocks, cheap nitroarenes, and carboxylic acids.

Aromatic tertiary amines, especially bearing benzylic units, are versatile building blocks in organic synthesis, as they contain differentiable C(sp^3^)-H, C-N bond, and C(sp^2^)-H bonds^1^. The biological activity of aromatic tertiary amines and their ability to influence natural neurotransmitter transmission routes enable them highly effective for drug discovery, in areas that range from the treatment of bone deterioration (such as a fracture)^2^ to cytopathy (such as cancer)^3^ (Fig. 1a). Consequently, the development of synthetic methods to forge C-N bonds for the synthesis of tertiary amines has gained considerable attention in recent decades^4–9^. Classical synthetic methods to access these tertiary amines are mainly derived from the reductive amination or nucleophilic substitution from aromatic amines that are produced industrially from nitroarenes^10–12^. However, the nucleophilic substitution of aromatic amines with benzylic halides may result in overalkylation side reactions to some extent, and despite being an efficient process, reductive amination requires the use of reactive aldehydes and reductants.

Nitro(hetero)arenes are readily available and commercially cheap feedstock reagents^13^. They have recently been used as amination reagents with the reduction in-situ, efficiently avoiding the hydrogenation step and thus improving step-economy and reducing cost^14–21^. The direct use of nitrobenzene as potential amines has gained highly active research interests^22–32^. Besides the classical ionic-type reductive amination with nitroarenes, the emerging radical reductive amination of nitroarenes with alkenes^33,34^ and organic halides^35,36^ has been successfully developed, however only practical for the construction of secondary amines (Fig. 1b). To the best of our knowledge, the direct use of nitroarenes for the construction of tertiary amines is still underdeveloped. Its success would represent a significant step forward to simplify tertiary amines synthesis from bench-stable and commercially cheap nitroarenes. As our continual interests in the highly efficient organic transformation of carboxylic acids^37–40^, we dedicate to developing a catalytic decarboxylative C-N coupling^41–48^ from carboxylic acids.

Herein, we develop a highly efficient catalytic strategy for decarboxylative C-N coupling using both commercially cheap carboxylic acids and nitroarenes, delivering a wide range of aromatic tertiary amines in 35–98% yields (Fig. 1c). Moreover, the use of two different carboxylic acids can allow for the concise synthesis of non-symmetric tertiary amines in synthetically acceptable yields.

## Results

### Reaction optimization

To initiate our study, we selected phenylacetic acid (**1**) and nitrobenzene (**2**) as model substrates with which to explore the optimized reaction conditions (Table 1). After a series of conditional screening, the standard conditions include 10 mol% FeI_2_, 1 mol% 4CzIPN, 10 mol% ligand (**L1**), and reductant (EtO)_3_SiH by means of irradiation of blue LEDs at 65 °C (entry 1). Under the standard conditions, the desired amine (**4**) can be obtained in 91% isolated yield. Notably, the classical S_N_2 nucleophilic substitution reaction of aromatic amines and benzylic halides is challenging to deliver tertiary amine (**4**) because of the competing overalkylation, instead of the formation of a quaternary ammonium salt. Under the optimized conditions, the use of other ligands (**L1** to **L5**) can decrease the yield (entries 2–5, Table 1). It is found that FeI_2_ is an important factor for the success as replacing with other iron-based catalysts resulted in a reduction of yields (entries 6, 7 and 10). We envisioned that ligands might affect the ligand-metal-charge-transfer (LMCT) process in the excited state of iron-complex^49–53^. Also, we found that other silanes in place of (EtO)_3_SiH cannot further increase the reaction efficiency (entry 8). Interestingly, the decarboxylative C-N coupling reaction could still occur in the absence of photocatalyst (4CzIPN) albeit with moderate yield (59% vs 91%). Much to our surprise, we found that if increasing the reaction time, the desired yield can still be obtained in 88% yield without photocatalyst (entry 9). This indicates that photocatalyst is not crucial but can improve reaction efficiency. This phenomenon has also been reported by others^54–58^ and we envisioned that the photocatalytic cycle could increase the concentration of benzyl radical (from decarboxylation of the carboxyl radical). The control experiment confirmed that the decarboxylative C-N coupling requires light irradiation since no reaction occurred in the dark otherwise under optimized conditions (entry 11). In addition, it is found that ligand (**L1**) has a great influence on the reaction yield. As a consequence, we measured the UV-Vis spectra of FeI_2_, ligand (**L1**), and their complexes. It is found that FeI_2_ has two absorption peaks at 292 nm and 363 nm. The ligand (**L1**) has one absorption peak at 412 nm, while their complex of FeI_2_ and **L1** has a new absorption peak at 513 nm (Supplementary Fig. 7). We speculated that the addition of ligand can not only affect the LMCT process but also may play a key role in the subsequent reduction of N-based intermediates.

**Table 1:** Optimization of catalytic amination conditions^a^.


Entry	Variation of standard conditions	Yield (%)^b^
1	None	92 (91^c^)
2	**L2** instead of **L1**	37
3	**L3** instead of **L1**	66
4	**L4** instead of **L1**	63
5	**L5** instead of **L1**	27
6	FeCl_3_ instead of FeI_2_	7
7	Fe(acac)_3_ instead of FeI_2_	nd
8	PhSiH_3_ instead of (EtO)_3_SiH	79
9	Without 4CzIPN	59 (88^d^)
10	Without FeI_2_	nd
11	Without light irradiation	nd

*nd* not detected, *blue LED* blue light-emitting diode.

^a^Standard conditions: 4CzIPN (1 mol%), FeI_2_ (10 mol%), **L1** (10 mol%), **1** (0.6 mmol), **2** (0.2 mmol), 2,6-lutidine (3.0 equiv.), (EtO)_3_SiH (2.0 equiv.), MeCN (2 mL), blue LEDs, 65 °C, 24 h.

^b^GC yield. ^c^Isolated yield.

^d^Time = 44 h. See Supplementary Information for additional optimization experiments.

### Substrate scope

**Fig. 2:**
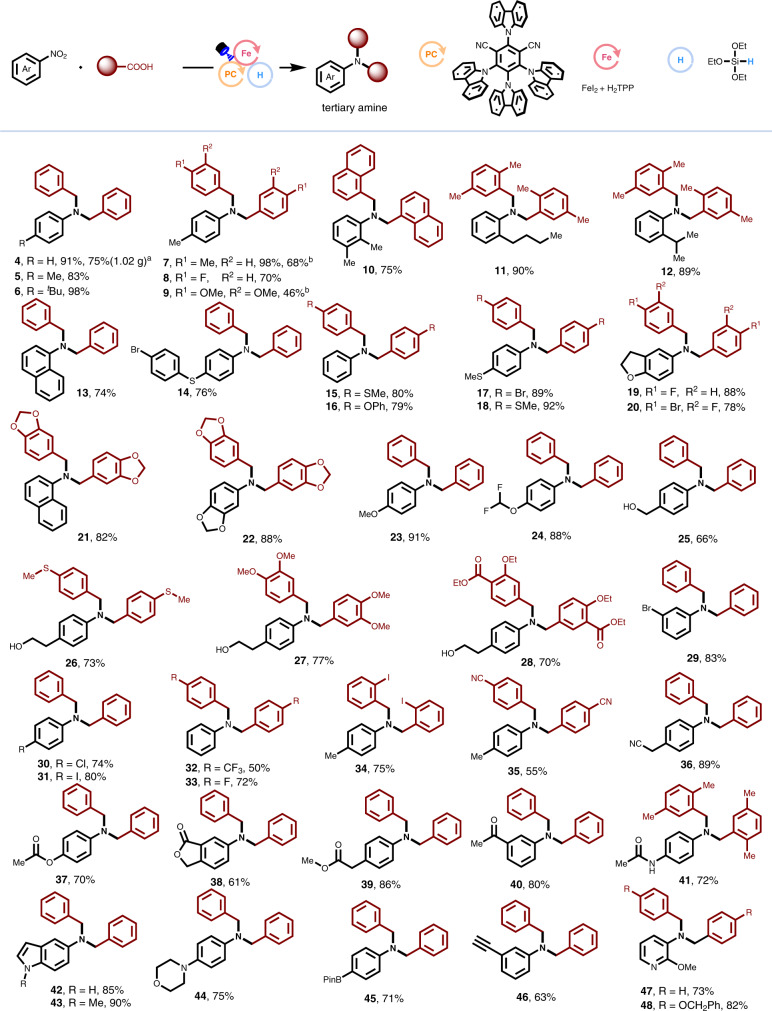
Scope of the carboxylic acids and the nitro compounds. Reaction conditions: 4CzIPN (1 mol%), FeI_2_ (10 mol%), **L1** (10 mol%), nitrobenzene (0.2 mmol), carboxylic acid (0.6 mmol), 2,6-lutidine (3.0 equiv.), (EtO)_3_SiH (2.0 equiv.), MeCN (2 mL), blue LEDs, 65 °C, 24 h. ^a^Gram-scale test with 0.2 mol% photocatalyst. Standard conditions for gram-scale experiment: **PC 3** (0.2 mol%), L1 (10 mol%), FeI_2_ (10 mol%), phenylacetic acid 1 (15 mmol), nitrobenzene **2** (5 mmol), 2,6-lutidine (15 mmol), (EtO)_3_SiH (10 mmol), MeCN (25 mL), blue LEDs, 65 °C, 72 h. ^b^Reaction time = 45 h, 25 °C.

With the optimal conditions in hand, we explored the substrate's scope of carboxylic acids and nitro compounds in an orthogonal way. The representative examples are shown in Fig. 2. In general, this decarboxylative C-N coupling protocol holds satisfactory functional group compatibility. A wide range of carboxylic acids and nitroarenes can proceed with this decarboxylative C-N coupling to construct tertiary amines (**4–48**) in moderate to excellent yields (45 examples, up to 98%). It was found that the substituents of nitroarenes on *ortho*-, *meta*-, and *para*-position on the phenyl rings can all occur in this transformation. More importantly, the sterically hindered methyl (**10**), *n*-butyl (**11**), and *i*-propyl (**12**) groups at the ortho-position of nitroarenes have little influence on the reaction efficiency. This indicates that steric hindrance hardly decreased the reactivity. A great number of versatile functional groups, such as ether (**16, 23, 24**), sulfide (**14, 15, 18**), hydroxyl (**25–28**), bromo (**29**), chloro (**30**), iodo (**31, 34**), fluoro (**33**), ester (**37–39**), ketone (**40**), amide (**41**), heterocycle (**42–43, 47–48**), boronic acid ester (**45**), and alkyne (**46**) remain intact during decarboxylative C-N couplings. These useful functional groups enable this protocol promising for the synthesis of highly functionalized tertiary amines. Another interesting feature of this decarboxylative tertiary amine synthesis is that it has excellent selectivity among several nucleophilic functional groups. For example, the electron-rich NH-free indole-based nitro compound can be applied for this decarboxylative C-N coupling (**42**), which readily undergoes nucleophilic addition and substitution. The tertiary amines units that are generally incompatible in traditional nucleophilic substitution reactions are also suitable for this strategy (**44**). In addition, nitroarenes bearing active C-H bonds also tolerated the reaction conditions well (**25–28, 36, 39–41**).

Besides the good functional group tolerance concerning nitroarenes, it was also found that a set of functionalized carboxylic acids are competent coupling partners as shown in Fig. 2. Both the electron-rich and -poor functional groups on the phenyl rings (**4–48**) can undergo this decarboxylative C-N coupling process, where the electron-rich groups usually give rise to the desired products in relatively better yields. We postulated that the electron-withdrawing groups on the arenes would decrease the nucleophilic features of the resulting benzylic radical, which is a key intermediate after decarboxylation. A wide range of substituted phenylacetic acid derivatives have been employed in this protocol and they can undergo this decarboxylative C-N coupling smoothly, affording the desired products (**4–48**) in 46–98% yields. Furthermore, with modified standard conditions using only 0.2 mol% 4CzIPN, a scaled-up experiment of 5 mmol can be conducted smoothly to afford the desired product (**4**) in 75% yield. However, due to the shorter lifetime of alkyl radicals, it is still difficult for common alkyl carboxylic acids at present.

**Fig. 3:**
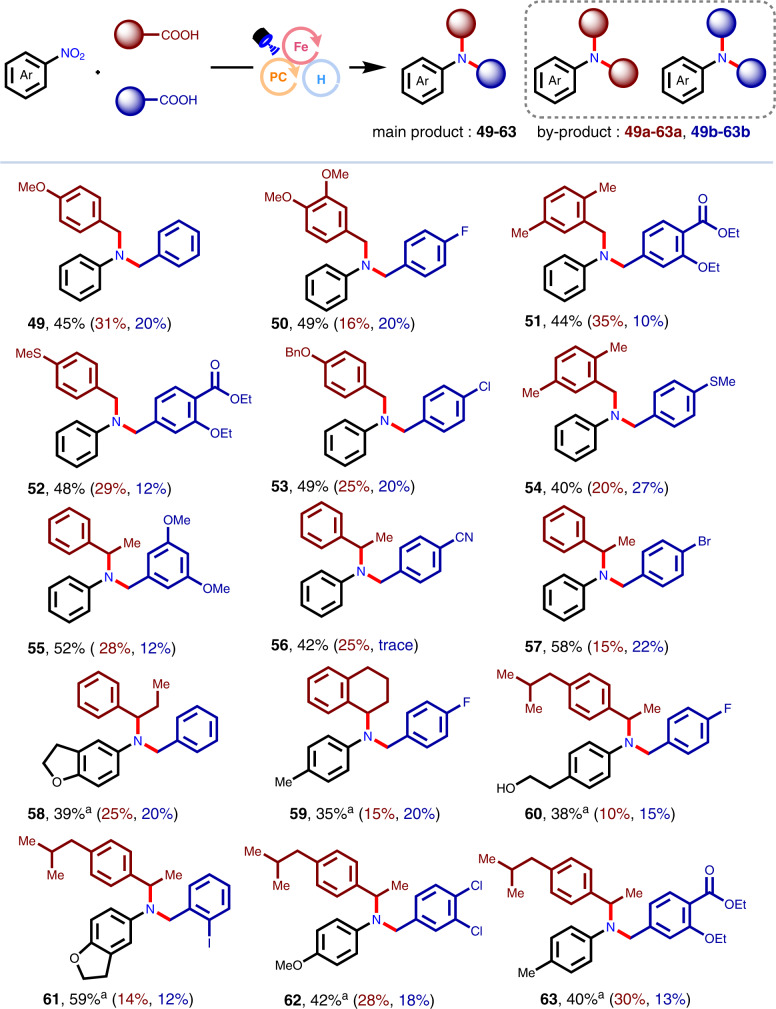
Representative examples for three-component coupling reactions. Isolated yield (main products: **49–63**; by-products: **49a–63a**, **49b–63b**) after chromatography were shown in turn. Reaction conditions: 4CzIPN (1 mol%), FeI_2_ (10 mol%), **L1** (10 mol%), carboxylic acid **A** (0.3 mmol), carboxylic acid **B** (0.3 mmol), nitroarenes (0.2 mmol), 2,6-lutidine (3.0 equiv.), (EtO)_3_SiH (2.0 equiv.), MeCN (2 mL), Blue LEDs, 65 °C, 24 h. ^a^Reaction time = 32 h.

Subsequently, two kinds of carboxylic acids have been employed to investigate a three-component reaction, which is able to construct non-symmetric aromatic tertiary amines (Fig. 3). Considering the differences in decarboxylation rates and generated radical stability of  aromatic acetic acids with different substituents, we subjected two different carboxylic acids to this protocol, successfully affording non-symmetric aromatic tertiary amines (15 examples) in 35–59% yield. It was found that secondary carboxylic acids were also tolerated. They can give rise to the desired products (**55–63**) in acceptable results, indicating that the steric hindrance of the aromatic benzyl group hardly affected the success of the radical decarboxylative C-N coupling reaction. Besides the cross-coupling products of two aryl acetic acids, a small amount of homocoupling by-products (**49a–63a**, **49b–63b**) were detected (Supplementary Fig. 3).

### Synthetic application

**Fig. 4:**
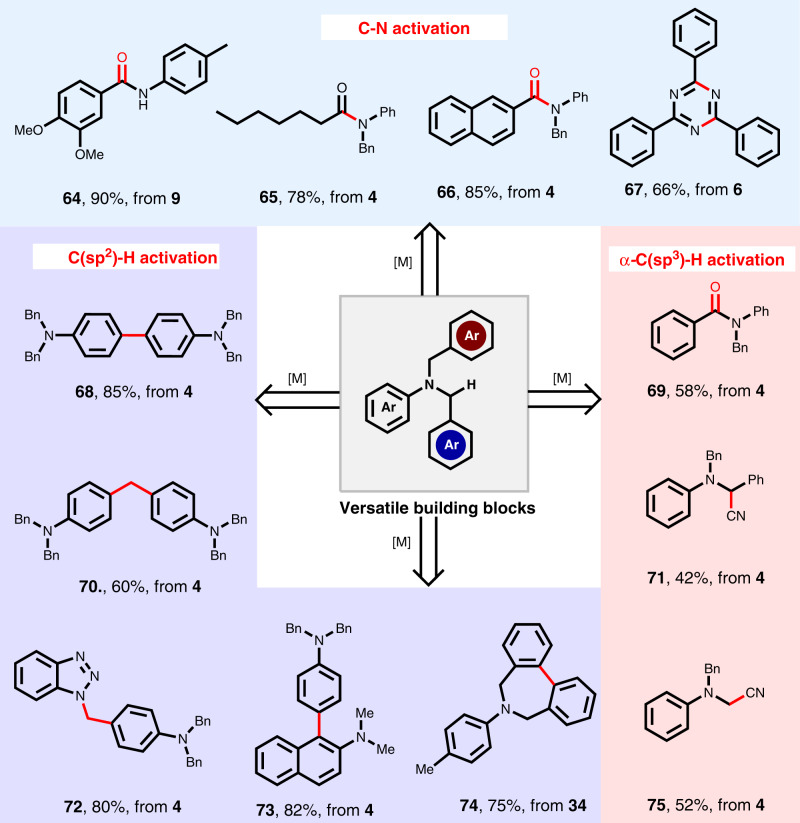
Synthetic application of derivatization of aromatic tertiary amines. [M] = Metal reagent. See Supplementary Information for detailed experimental conditions.

The versatile transformations of aromatic tertiary amines as building blocks in the field of organic synthesis are explored (Fig. 4). Three different amides (**64–66**) and triazine (**67**) were obtained through selective C-N bond functionalization. Similarly, the amino group, as one of the main groups regulating the electrical properties of aromatic rings, can significantly affect the sp^2^ C-H bonds of para positions. Several new valuable compounds (**68**, **70**, **72, 73**) have been obtained via controllable para-C-H functionalization of aromatic tertiary amines. Also, the α-C-H bonds adjacent to nitrogen atoms can be easily activated, furnishing products (**69**, **71**, **75**) in moderate yields. The cyclic aromatic tertiary amines (**74**) can also be obtained by aromatic ring dehalogenation coupling from product **34**. The success of these derivatized products suggests the potential application of this protocol in organic synthesis.

### Mechanistic studies

**Fig. 5:**
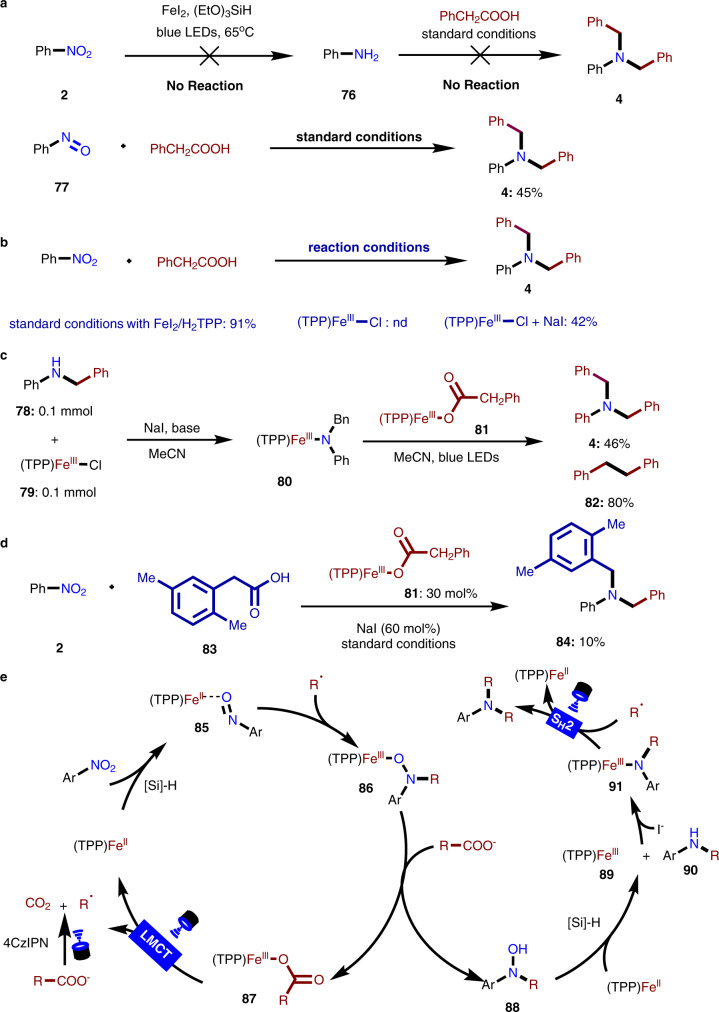
Mechanism study. **a** Controlled experiment. **b** Effects of various iron catalysts, ligand **L1**, and additive NaI on reaction and the study of S_H_2. **c** Cross-over experiment between **80** and **81**. **d** The three-component cross-coupling. **e** Plausible mechanism.

In order to explore the mechanism of the reaction, the following experiments were carried out. First, under the standard conditions, when 2 equiv. of TEMPO (2,2,6,6-tetramethylpiperidine-1-oxyl radical) was added into the reaction mixture, the reaction was completely inhibited and the corresponding benzyl radical was trapped by TEMPO (Supplementary Fig. 4), which was identified by high-resolution mass spectroscopy (HR-MS). The results of controlled experiments showed that nitrobenzene (**2**) cannot be reduced to aniline under experimental conditions. On the other hand, when aniline was employed to react with corresponding carboxylic acids, the desired product (**4**) could not be formed. Based on the above results, we hypothesized that nitrobenzene may be involved in the Fe(II)/Fe(III) catalytic cycle as an oxidant. In the light of other’s work^14–36^, we speculated that nitrobenzene might be reduced to form unstable and high activity N-based intermediates. When relatively stable nitrosobenzene (**77**) was added into the reaction mixture to replace nitrobenzene, the target product (**4**) was obtained in 45% yields. Accordingly, we hypothesized that nitrosobenzene (**77**) may be one important intermediate of this reaction (Fig. 5a).

Interestingly, when Fe(TPP)(III)Cl was used as the catalyst, no decarboxylative C-N coupling occurred (Fig. 5b). However, when 50 mol% amount of NaI was added to the above mixture, the desired reaction can readily proceed to afford the target product (**4**) in 42% yield. This may imply that the presence of iodide anion is important for such transformation. Accordingly, corresponding Fe-amine complex (**80**) and Fe-carboxylate complex (**81**) were prepared, respectively. Under the irradiation of blue light, the desired C-N bonds coupling between Fe-amine complex (**80**) and Fe-carboxylate complex (**81**) smoothly proceed to afford the product (**4**) in 46% yield (Fig. 5c). On the other hand, the byproduct, 1,2-diphenylethane (**82**) was formed during this process, suggesting the success of the decarboxylative process to generate benzyl radical from Fe-carboxylate complex (**81**). It is worth noting that blue light and NaI are crucial conditions for this reaction. Finally, the three-component reaction of nitrobenzene (**2**), the carboxylic acid (**83**) and Fe-carboxylate complex (**81**) can successfully give rise to the desired cross-coupling product (**84**) in 10% yield, which again confirms the Fe-carboxylate complex (**81**) would be a potential intermediate of the reaction (Fig. 5d).

According to the above mechanism experiments, a plausible mechanism was proposed and is shown in Fig. 5e. Nitro compounds are able to be reduced by (TPP)Fe(II) catalyst with (EtO)_3_SiH to form Fe-based complexes (**85**)^34^. The alkyl radical, which is generated via either the visible-light-mediated single-electron oxidation of carboxylic anion or light-mediated LMCT process of Fe-carboxylate (**87**), then attacks the **85** or **77** (Fig. 5a) to form the Fe(III) complex (**86**)^34,59^. Subsequently, it can undergo anion exchange with carboxylic anion to produce the hydroxylamine compound (**88**) and regenerate the Fe-species (**87**). Alternatively, the direct radical addition of alkyl radical to nitrosobenzene and subsequent recombination with (TPP)Fe(II) to produce **86** is also likely. The excited complex of carboxylic acid and iron(III) (**87**) proceeds through homolysis to form alkyl radical and iron catalyst (TPP)Fe(II) through a ligand-to-metal charge transfer (LMCT) pathway^49–53^. Simultaneously, in the light of Baran’s pioneering work, the resulting hydroxylamine compound (**88**) is able to react with (TPP)Fe(II) species to generate secondary intermediate (**90)** and (TPP)Fe(III) (See Supplementary Information)^33^. In the presence of iodide anion, the resulting (TPP)Fe(III) species and intermediate (**90)** may recombine to form the Fe-based complex (**91**). With the irradiation of blue LEDs, the homolytic S_H_2^60–66^ process can occur between **91** and alkyl radical, resulting in the formation of tertiary amines and the regeneration of (TPP)Fe(II). The by-products of the reaction might also confirm this mechanism (Supplementary Fig. 5).

## Discussion

In summary, we have developed a method to construct two C-N bonds at once enabled by the catalysis of (TPP)Fe-complex with light irradiation, efficiently constructing a variety of synthetically valuable tertiary amines (up to 98% yields). A wide range of bench-stable carboxylic acids and nitroarenes are competent coupling partners in this decarboxylative C-N coupling. The use of two different carboxylic acids allows for the concise synthesis of nonsymmetric tertiary amines in synthetically useful yields. It represents a complementary manner to construct aromatic tertiary amines and can avoid the overalkylation limitation of the classical nucleophilic substitution process of anilines. The mechanistic studies suggest an S_H_2 pathway, and thus hold advances in the selectivity among different nucleophilic functional groups. The detailed mechanistic study and exploration of this meaningful bonding-formation manner is ongoing in our group.

## Methods

### General procedure for amidation

4CzIPN (1.58 mg, 1 mol%), **L1** (12.3 mg, 10 mol%), FeI_2_ (6.2 mg, 10 mol%) were placed in an 8 mL transparent vial equipped with a stirring bar. This vial was carried into the glovebox, which is equipped with nitrogen. Then aromatic acid (0.6 mmol), nitroarenes (0.2 mmol), MeCN (1.0 mL), 2,6-lutidine (70 μL, 0.6 mmol) and (EtO)_3_SiH (80 μL, 0.4 mmol), were added in sequence under N_2_ atmosphere. The reaction mixture was stirred under the irradiation of blue LEDs (distance app. 5.0 cm from the bulb) at 65 °C for 24 h. When the reaction finished, the mixture was quenched with water and it was extracted with ethyl acetate (3 × 10 mL). The organic layers were combined together and concentrated under vacuo. The product was purified by flash column chromatography on silica gel (petroleum ether: ethyl acetate).

## Supplementary information

Supplementary Information

## Data Availability

We declare that all other data supporting the findings of this study are available within the article and Supplementary Information files.
