# Supplementary material for: Decarboxylative tandem C-N coupling with nitroarenes via SH2 mechanism
Source: Nat Commun. 2022 May 4;13:2432. doi: 10.1038/s41467-022-30176-z (PMC9068905; doi:10.1038/s41467-022-30176-z)
Supplement: Supplementary file 1 — Supplementary Information [file 41467_2022_30176_MOESM1_ESM.pdf]

Supplementary Information

Decarboxylative Tandem C-N Coupling with Nitroarenes via S_H2 Mechanism

Wang et al.

Supplementary Notes

1 General information

All the reactions were conducted in transparent vials under Argon atmosphere unless otherwise noted. All solvents were obtained from commercial suppliers and used without further purification. Anhydrous MeCN was purified from MeCN (≥99.9%, HPLC) by Solvent Purification System. Reagents were purchased from Energy Chemical, Adamas-beta, and etc. Flash column chromatographic purification of products was accomplished using forced-flow chromatography on Silica Gel (300-400 mesh).

^{1}H NMR, ^{13}C NMR spectra were recorded on a 400 or 500 MHz spectrometer in $CDCl_3$ (δH = 7.26 ppm, δC = 77.0 ppm as standard). Data for ^{1}H NMR are reported as follows: chemical shift (ppm, scale), multiplicity, coupling constant (Hz), and integration. Data for ^{13}C NMR are reported in terms of chemical shift (ppm, scale), multiplicity, and coupling constant (Hz). The following abbreviations were used for ^{1}H NMR spectra to indicate the signal multiplicity: s (singlet); brs (broad singlet), d (doublet), t (triplet), q (quartet), quint (quintet), sext (sextet), sept (septet) and m (multiplet) as well as combinations of them. Gas chromatographic (GC) analyses were performed on a GC equipped with a flame-ionization detector and an rtx@-65 (30 m × 0.32 mm ID × 0.25 μm df) column. GC-MS analyses were performed on a GC-MS with an EI mode. High-resolution mass spectra were obtained by ESI on a TOF mass analyzer. The blue LEDs light was purchased from Kessil.

Supplementary Methods

2 Optimization of the Reaction Conditions

Supplementary Table 1. Screen of ligands.

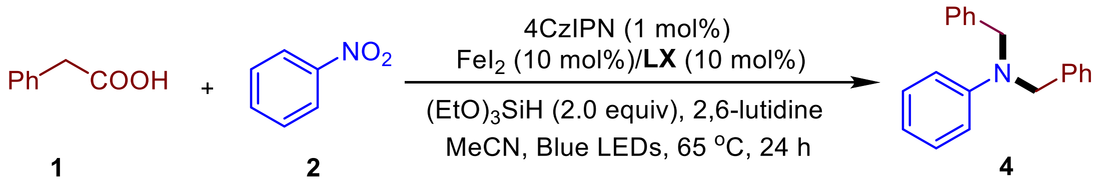

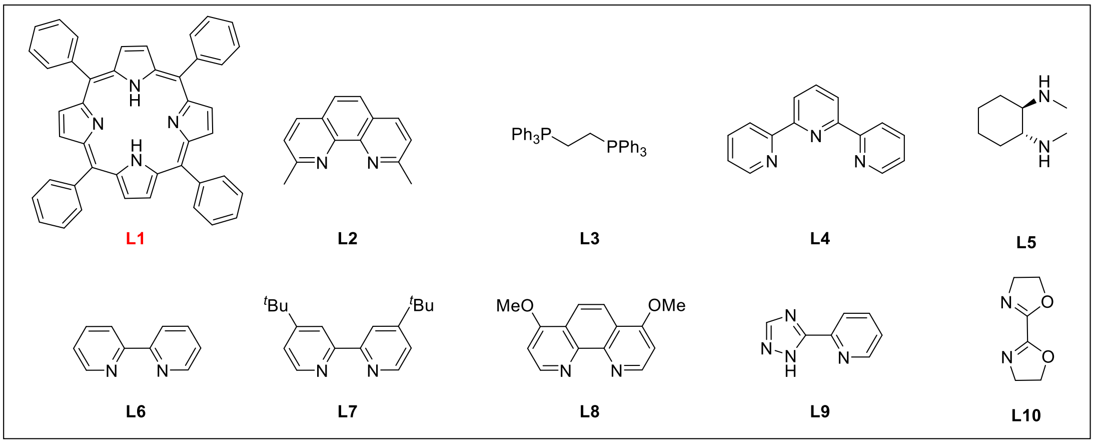

Entry	Ligand	Yield (%)
1	L1	92
2	L2	0
3	L3	66
4	L4	63
5	L5	27
6	L6	35
7	L7	13
8	L8	37
9	L9	16
10	L10	30

Reaction conditions: **1** (0.6 mmol), **2** (0.2 mmol), 4CzIPN (1 mol%), FeI_2 (10 mol%), ligand (10 mol%), 2,6-lutidine (3.0 equiv), silanes (2.0 equiv), MeCN (1.0 ml), blue LEDs, 65 ℃, 24 h. Measured by GC using biphenyl as internal standard.

Supplementary Table 2. Screening of iron catalyst.

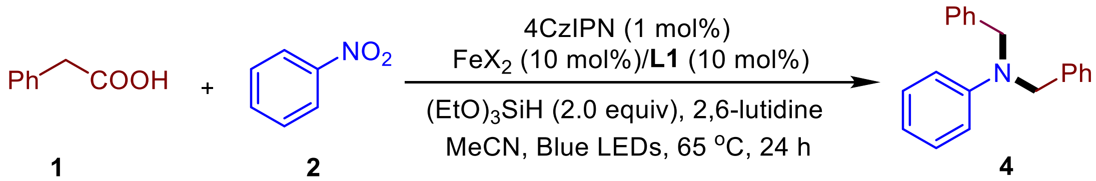

Entry	Iron	Yield (%)
1	FeI_2	92
2	$FeBr_2$	23
3	$FeCl_2$	trace
4	FeF_2	trace
5	$Fe(acac)_3$	0
6	$FeCl_3$	7
7	$NiCl_2$	0
8	$NiBr_2$	0

Reaction conditions: **1** (0.6 mmol), **2** (0.2 mmol), 4CzIPN (1 mol%), FeX_2 (10 mol%), **L1** (10 mol%), 2,6-lutidine (3.0 equiv), silanes (2.0 equiv), MeCN (1.0 ml), blue LEDs, 65℃, 24 h. Measured by GC using biphenyl as internal standard.

Supplementary Table3. The initial testes for aliphatic carboxylic acids

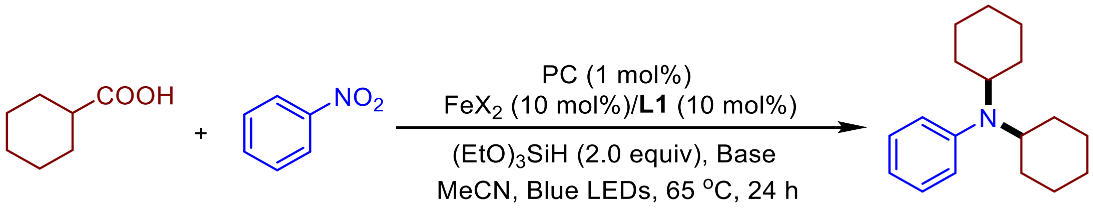

Entry	Iron	Yield (%)
1	FeI_2	trace
2	$FeBr_2$	0
3	$FeCl_2$	0
4	FeF_2	0
5	$Fe(acac)_3$	0
6	$FeCl_3$	0
7	$FeBr_3$	trace
8	$Fe(OTf)_3$	0

Reaction conditions: **1** (0.6 mmol), **2** (0.2 mmol), 4CzIPN (1 mol%), FeX_2 (10 mol%), **L1** (10 mol%), 2,6-lutidine (3.0 equiv), silanes (2.0 equiv), MeCN (1.0 ml), blue LEDs, 65℃, 24 h. Measured by GC using biphenyl as internal standard.

3 General procedure for tertiary amine

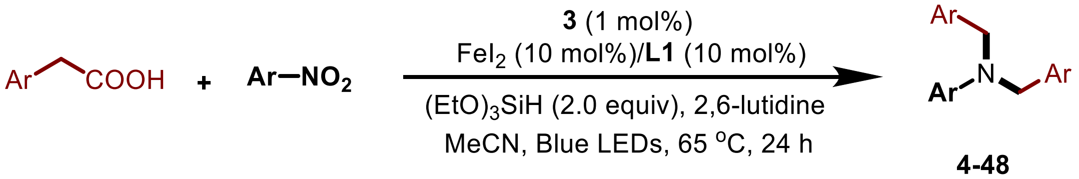

4-48

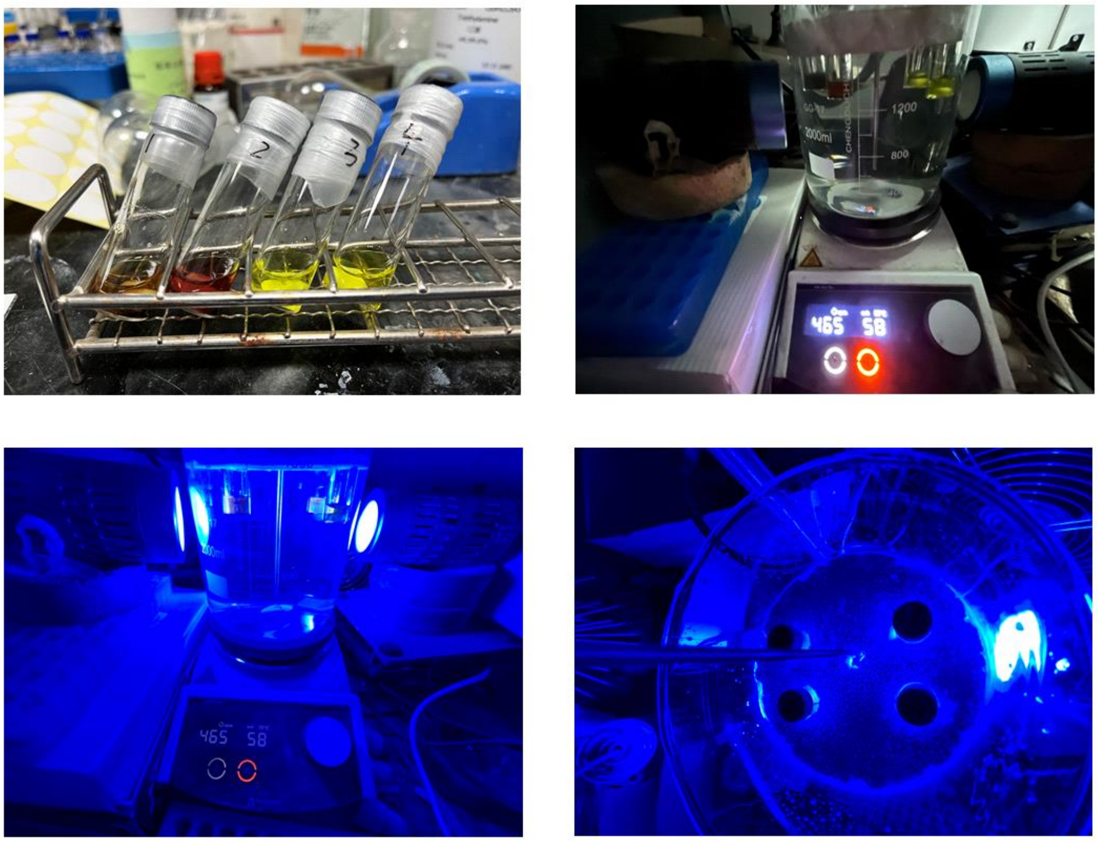

Supplementary Figure 1. Reaction set-up

0.2 mmol scale: 4CzIPN (1.58 mg, 1 mol%), **L1** (12.3 mg, 10 mol%), FeI_2 (6.2 mg, 10 mol%) were placed in an 8 mL transparent vial equipped with a stirring bar. This vial was carried into the glovebox which is equipped with nitrogen. Then aromatic acid (0.6 mmol), nitroarenes (0.2 mmol), MeCN (1.0 mL), 2,6-lutidine (70 μL, 0.6 mmol) and $(EtO)_3SiH$ (80 μL, 0.4 mmol), were added in sequence under N_2 atmosphere. The reaction mixture was stirred under the irradiation of 45 W blue LEDs (distance app. 5.0 cm from the bulb) at 65 °C for 24 h. When the reaction finished, the mixture was quenched with water and it was extracted with ethyl acetate (3 x 10 mL). The organic layers were combined together and concentrated under vacuo. The product was purified by flash column chromatography on

silica gel (petroleum ether: ethyl acetate).

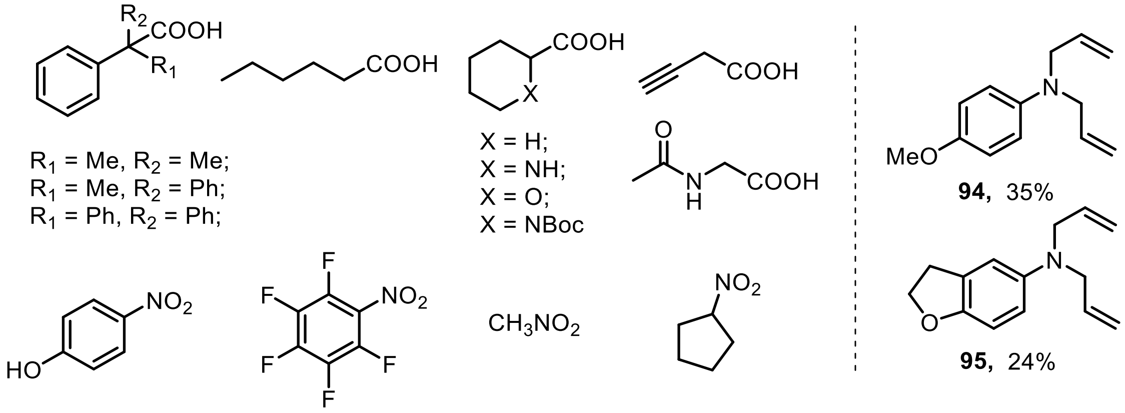

Supplementary Figure 2. Some unsuccessful examples and low yield examples.

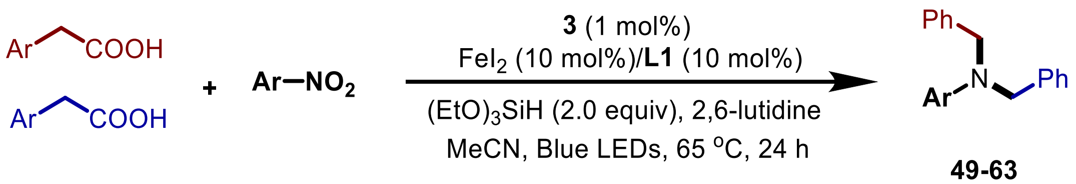

0.2 mmol scale: 4CzIPN (1.58 mg, 1 mol%), **L1** (12.3 mg, 10 mol%), FeI_2 (6.2 mg, 10 mol%) were placed in an 8 ml transparent vial equipped with a stirring bar. Then the vial was carried into glovebox which is equipped with nitrogen. Then two kind of aromatic acids (0.3 mmol, 0.3 mmol), nitroarenes (0.2 mmol), MeCN (1.0 ml), 2,6-lutidine (70 μL, 0.6 mmol) and $(EtO)_3SiH$ (80 μL, 0.4 mmol), were added in sequence under N_2 atmosphere. The reaction mixture was stirred under the irradiation of blue LEDs (distance app. 5.0 cm from the bulb) at 65℃ for 24 h. When the reaction finished, the mixture was quenched with water and extracted with ethyl acetate (3 x 10 mL). The organic layers were combined together and concentrated under vacuo. The product was purified by flash column chromatography on silica gel (petroleum ether : ethyl acetate).

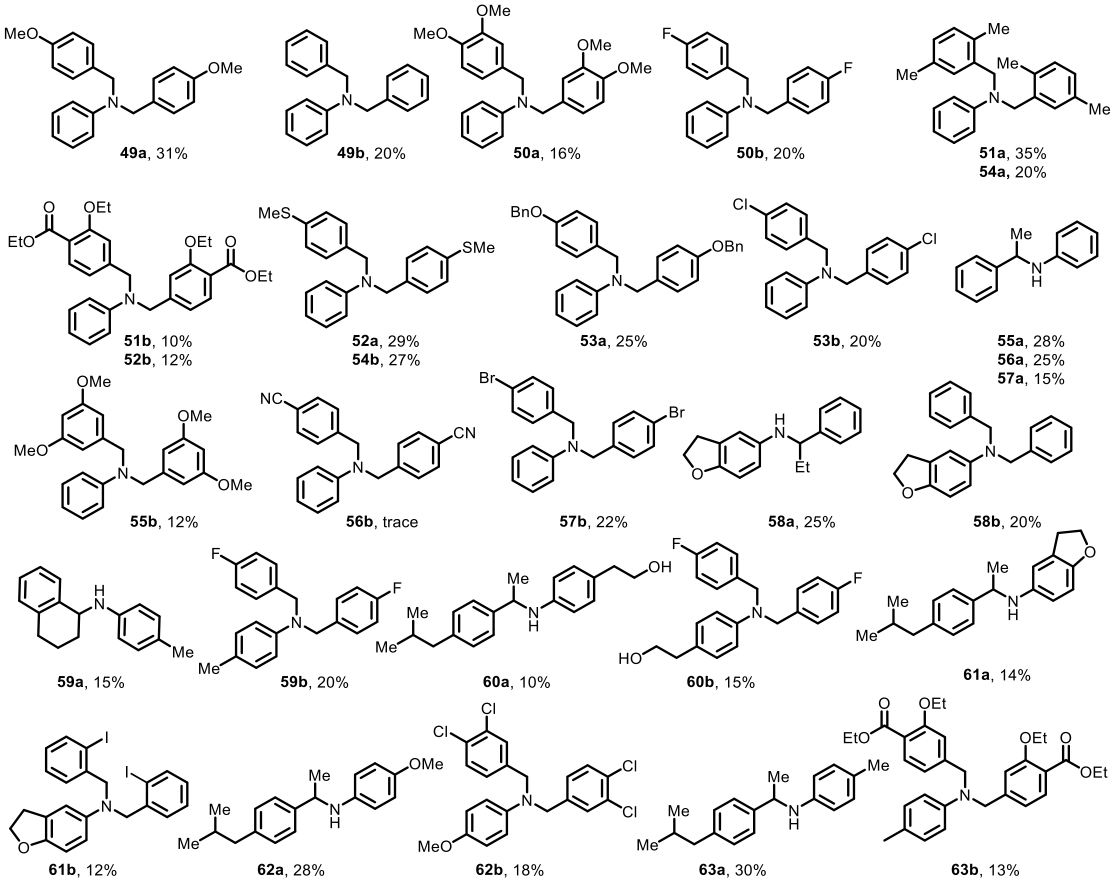

Supplementary Figure 3. Homocoupling products (by-products): **49a-63a**, **49b-63b**

Gram-scale with 0.2 mol% photocatalyst:

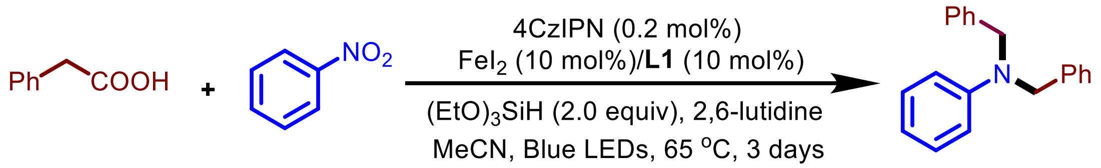

To a 100 mL round-bottom flask equipped with a stirring bar, FeI_2 (155.0 mg, 10 mol%), 4CzIPN (7.9 mg, 0.2 mol%), **L1** (307.5 mg, 10 mol%), carboxylic acids **1** (2.0 g, 15 mmol) were added successively. Then the vial was carried into glovebox which is equipped with nitrogen. Then MeCN (25 mL), nitroarene **2** (5 mmol), $(EtO)_3SiH$ (10 mmol), 2,6-lutidine (15 mmol) and nitroarenes **2** (5 mmol) were added in sequence under N_2 atmosphere. The reaction mixture was stirred under the irradiation of blue LEDs (distance app. 5.0 cm from the bulb) at 65℃ for 3 days. When the reaction finished, the mixture was quenched with water and extracted with ethyl acetate (3 x 50 mL). The organic layers were combined and concentrated under vacuo. The product was purified by flash column chromatography on

silica gel (petroleum ether : ethyl acetate = 50:1) to afford the product **4** as 1.02 g (75%), white solid.

4 Investigation of the reaction mechanism

4.1 Radical inhibition experiments

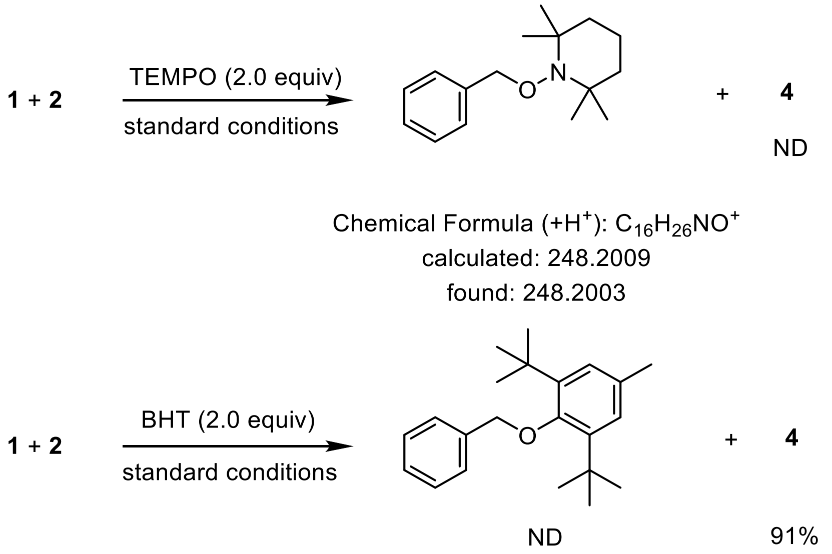

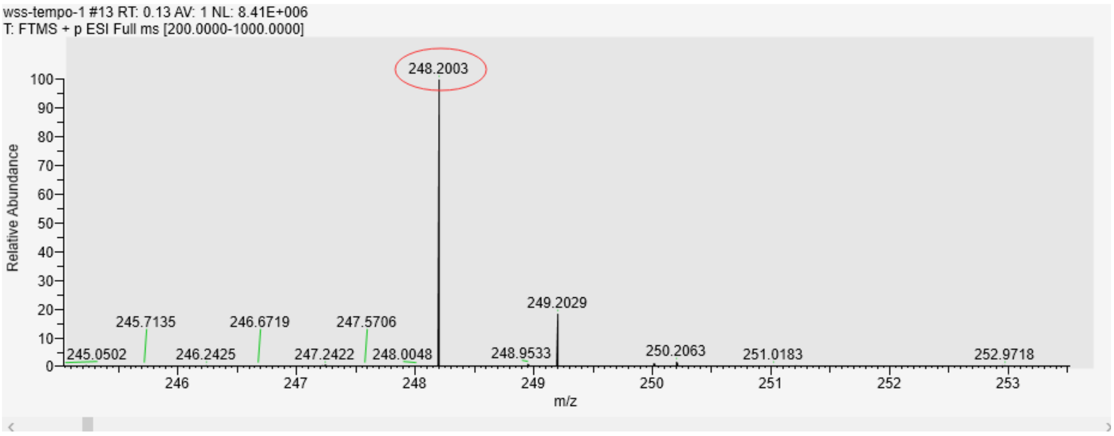

Supplementary Figure 4. Radical inhibition experiments

The reaction was nearly completely inhibited by TEMPO, but BHT didn't work. The N-O compound was detected by HRMS, which suggest that a benzyl free radical might be involved in this transformation. These results indicated that the reaction probably proceeded via a free radical process.

4.2 GC-MS monitors compounds in the reaction system.

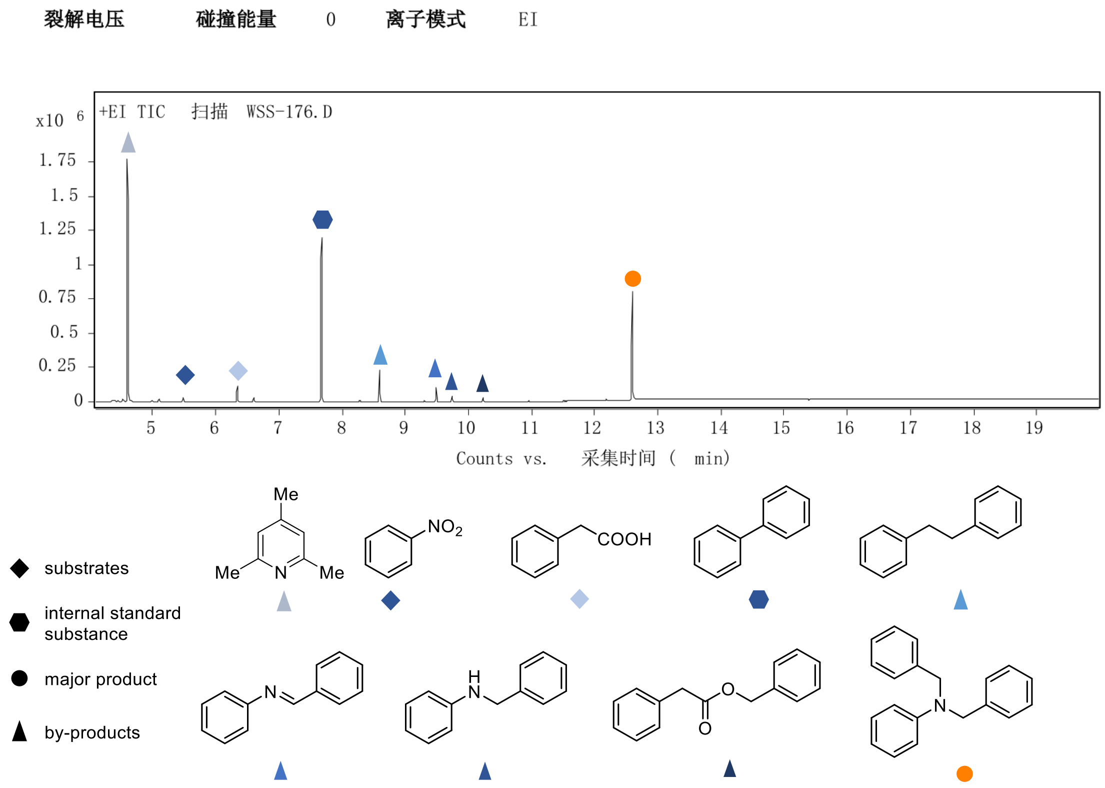

Supplementary Figure 5. GC-MS data

Using 2,4,6-Collidine instead of 2,6-Lutidine as base (The result is not affected), we can get data graph containing all the by-products, which may be caused by the different properties of the base. Unfortunately, the by-products ($(EtO)_4Si$ and $(EtO)_3Si$-O-$Si(EtO)_3$) of $(EtO)_3SiH$ are not shown. Through GCMS monitoring, we can get some reliable data based on molecular weight and peak time. When the catalytic cycle rate is not matched, two substrates conversion is incomplete. The formation of self-coupling products of benzyl radicals again indicated the presence of benzyl radicals in the system. We suspect that the secondary amine and imine come from the reduction or dehydration of **88**. To a certain extent, by-products proved the rationality of the mechanism (Fig. 5).

4.3 The light on/off experiments[14]

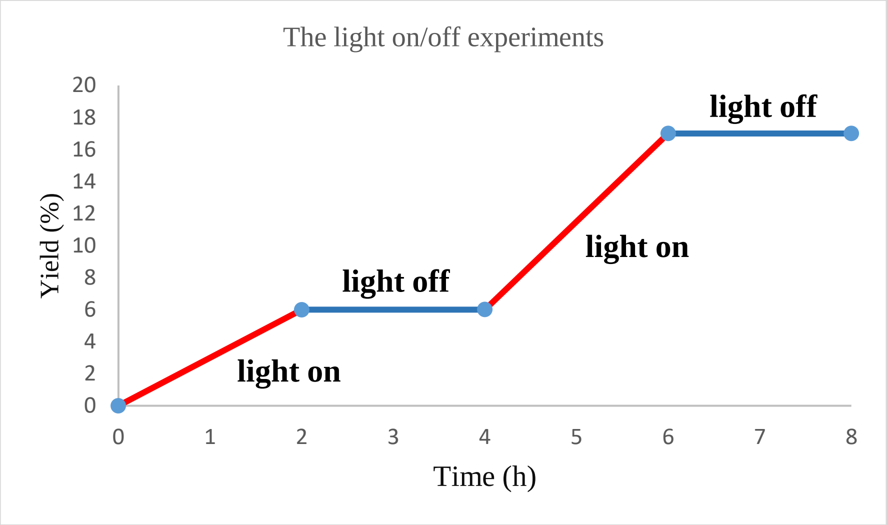

Supplementary Figure 6. The light on/off experiments

When the light is turned on, the reaction takes place. When the lights are turned off, the reaction stops. Experiments show that the reaction involving a free radical chain reaction might be less likely.

4.4 UV-Vis spectra

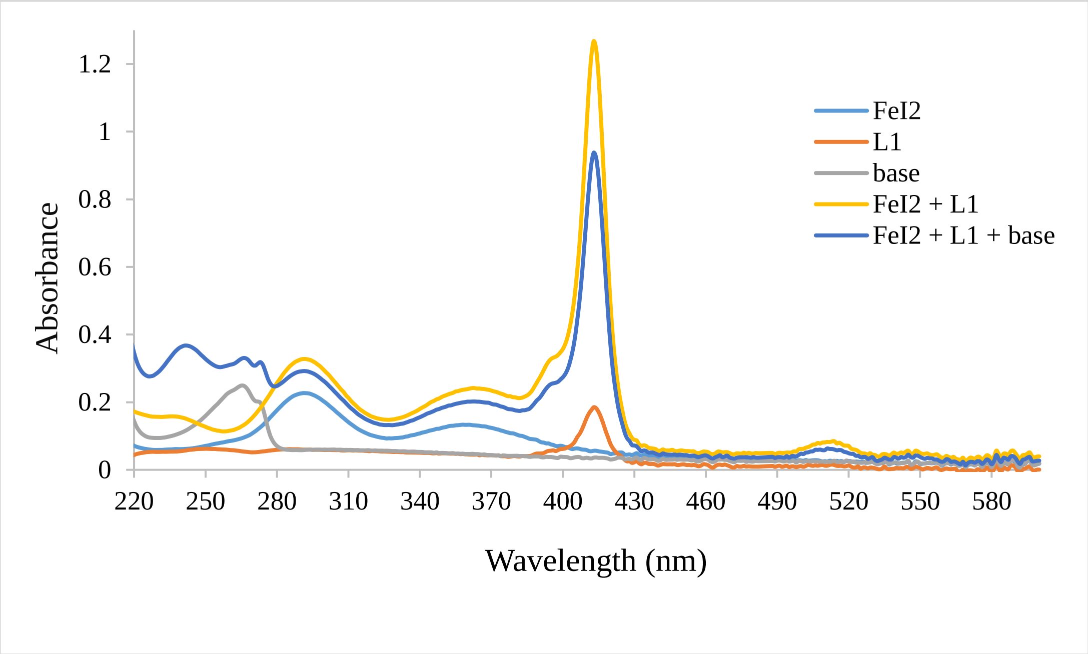

Supplementary Figure 7. UV-Vis spectra

According to the experimental results, the ligand **L1** has absorption peaks at 415nm, the catalyst FeI_2 has two absorption peaks at 290nm and 360nm, and the absorption peak of the base is less than 300nm. When the ligand **L1** and catalyst FeI_2 are mixed, not only is

there an absorption peak at 415nm, but a new absorption peak is also added at 514nm. The above phenomenon indicates that the ligand **L1** and FeI_2 form coordination, forming a new optical catalytic active center, and increasing the catalytic activity of iron. This is also consistent with the experimental results.

4.5 Time-Yield curve

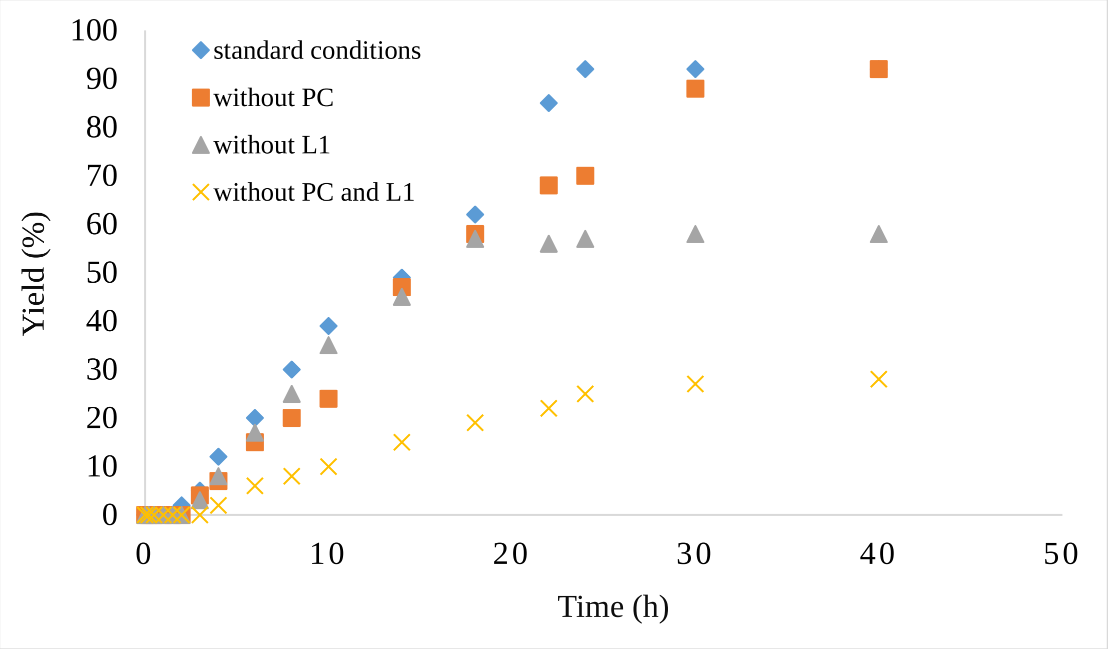

Supplementary Figure 8. Yield curve with time

The experimental results showed that 92% yield of the target product could be obtained in 24 hours under standard conditions. At the same time, without ligand **L1** and photocatalyst 4CzIPN, the reaction started slowly and the ideal yield could not be reached by prolonging the reaction time. The ideal yield can be obtained by prolonging the reaction time without adding photocatalyst. These results indicate that ligands are crucial to the catalytic cycle rate and efficiency of the reaction. 4CzIPN and FeI_2 can cooperate to improve efficiency.

4.6 Control experiment

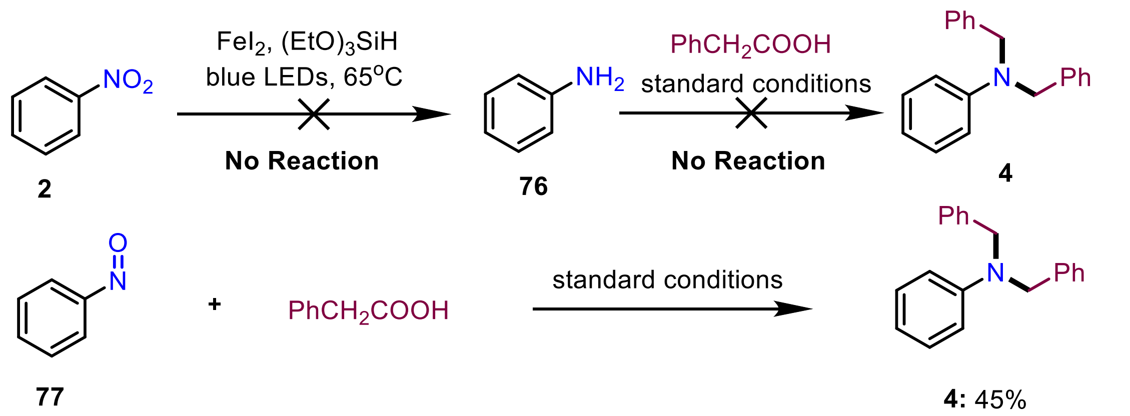

Through experiments, we found that no matter heated or blue light irradiation, nitrobenzene **2** could not be reduced to aniline **76**. Subsequently, we substituted aniline **76** for nitrobenzene **2** under standard conditions, but the target product could not be obtained. Therefore, we believe that aniline **76** may not be the intermediate of the reaction. Next, under the conditions, by substituting nitrosobenzene **77** for nitrobenzene **2** in the reaction, the target product **4** can be obtained in 45% yield. We hypothesized that nitrosobenzene **77** may be one of the active intermediates of the reaction.

4.7 Study of possible intermediates

*4.7.1 Synthesis of Fe-N complex **80***[1,2]

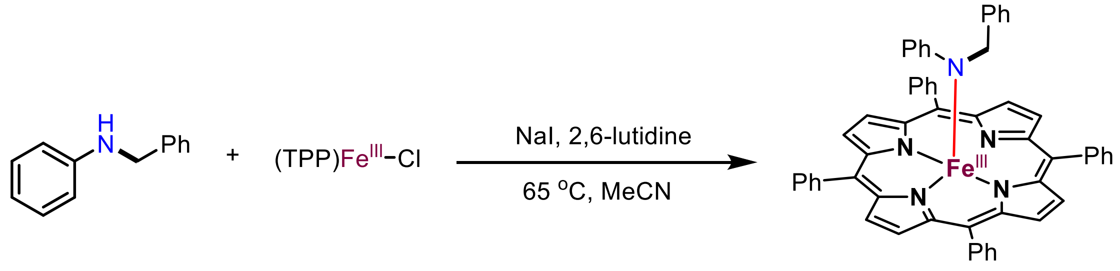

80

The reaction tube was charged with N-Phenylbenzylamine (1.0 mmol), iron catalysts (0.9 mmol), NaI (20 mmol%) and 2,6-lutidine (1.5 mmol) in MeCN (10 ml). The reaction mixture was stirred in the presence of N_2 under 60 °C for 24 h. After completion of the reaction, the solvent was evaporated under reduced pressure. The crude product **80** was dried to get black powder, 372 mg, 52% yield.

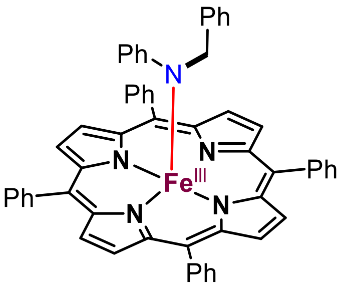

^{1}H NMR (500 MHz, Chloroform-*d*) δ 80.30 (b, 4H), δ 13.34 (s, 5H), 12.16 (s, 5H), 8.25 – 7.00 (m, 14H), 6.85 – 5.97 (m, 6H), 5.48 – 3.48 (m, 6H).

*4.7.2 Synthesis of Fe-O complex **81**.* [3]

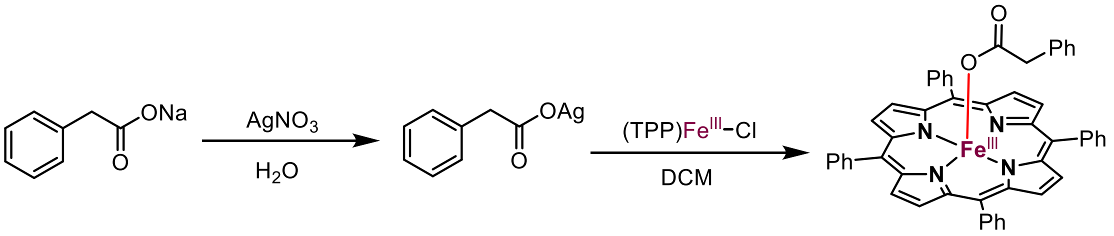

81

The reaction tube was charged with sodium phenylacetate (5.0 mmol) and silver nitrate (5.0 mmol) in H_2O (20 ml). The reaction mixture was stirred and precipitated white solid. The crude carboxylate silver product was washed with distilled water, was filtered and dried before being put into the next reaction.

The reaction tube was charged with carboxylate silver product (1.0 mmol) and (TPP)Fe^{III}-Cl (1.0 mmol) in DCM (20 ml). The reaction mixture was stirred in the presence of N_2 for 24 h. After completion of the reaction, the solvent was evaporated under reduced pressure. Then dissolve the solids with DCM, filter the mixture and dry the black power. The crude products **81** obtained were used in subsequent research.

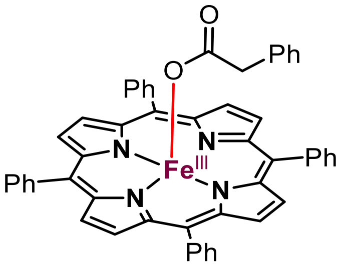

^{1}H NMR (500 MHz, Chloroform-*d*) δ 79.12 (b, 4H), 13.54 (s, 4H), 12.35 (s, 5H), 11.38 (s, 5H), 8.33 – 7.54 (m, 12H), 6.74 (s, 5H).

*4.7.3 Evidence for LMCT pathway: stoichiometric reaction of Fe-O complex **81**.*

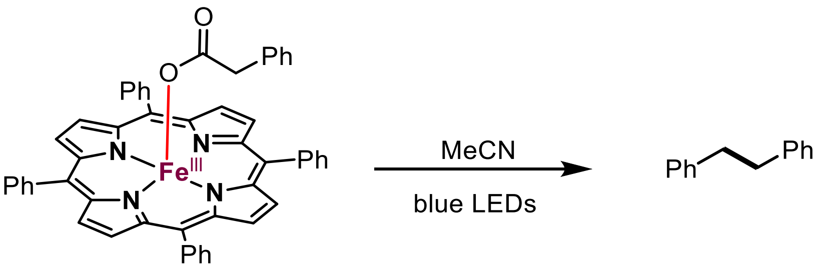

81 **82**

To an 8 mL vial equipped with a stir bar was added Fe-O complex **81** (50.0 mg, 0.05 mmol). After adding MeCN (1.0 ml), the vial was capped and placed under a nitrogen-atmosphere. The vial was stirred under the irradiation of blue LEDs (distance app. 5.0 cm from the bulb) at 65℃ for 24 hours. We can get product **82** about 80% yields.

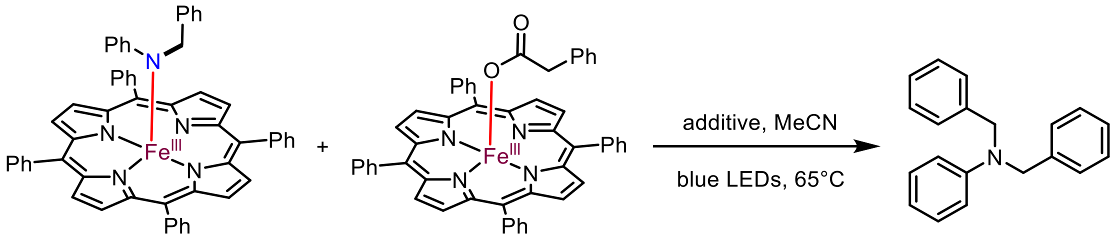

80 **81** **4**

The 8 ml reaction vial was charged with Fe-N complex **80** (8.5 mg, 0.01 mmol, 1 equiv.), Fe-O complex **81** (16.1 mg, 0.02 mmol, 2 equiv.) and MeCN (1ml) was added. Four such vials were prepared in parallel. And two vials were added NaI (1.5 mg, 0.01 mmol, 1equiv.). These vials were stirred under the irradiation of blue LEDs (distance app. 5.0 cm from the bulb) or at 65℃ for 24 hours. These vials were analyzed by GC with direct comparison to the authentic product of **4**, and yields were determined using 1,2-Diphenylethane as a standard.

	without light/65°C	blue LEDs
none	nd	nd
add NaI	nd	46%

Supplementary Figure 9. GC yields.

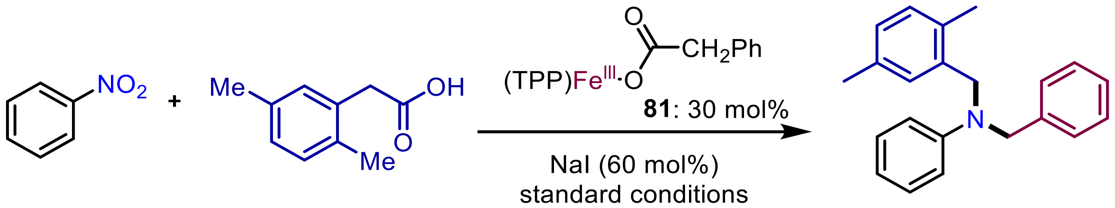

2 **83** **84**

To an 8 mL vial equipped with a stir bar was added nitrobenzene **2** (0.2 mmol, 1.0 equiv.), acid **83** (0.4 mmol, 2.0 equiv.), NaI (60 mol%), 2,4-lutidine (0.4 mmol, 2.0 equiv.), $(EtO)_3SiH$ (0.4 mmol, 2.0 equiv.) and Fe-O complex **81** (50.0 mg, 0.05 mmol). After adding MeCN (1.0 ml), the vial was capped and placed under a nitrogen-atmosphere. The vial was stirred under the irradiation of blue LEDs (distance app. 5.0 cm from the bulb) at 65 ℃ for 24 hours. We can get product **84** about 10% yields by analyzing GC-MS data.

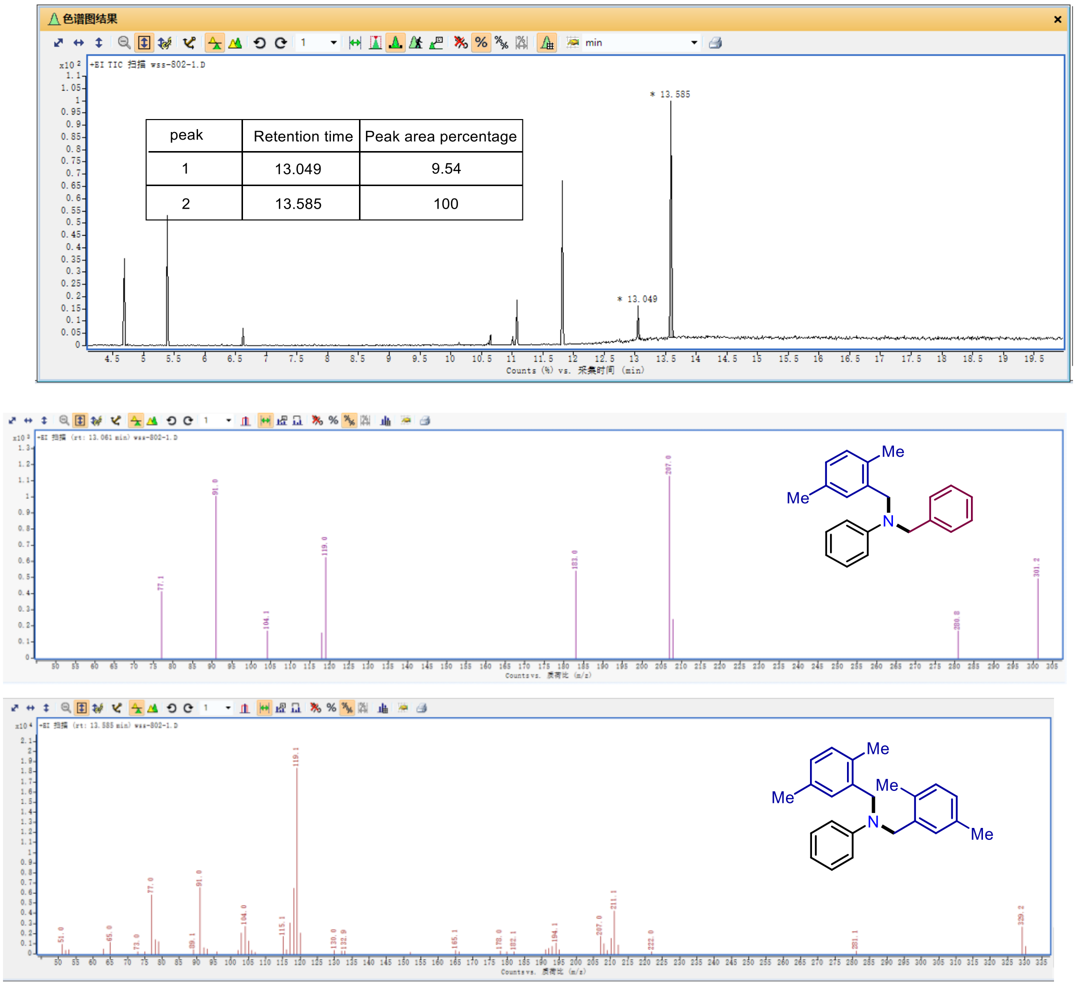

peak	Retention time	Peak area percentage
1	13.049	9.54
2	13.585	100

Supplementary Figure 10. GC-MS data

*4.7.4 Evidence for reduction of hydroxylamine **92**.*

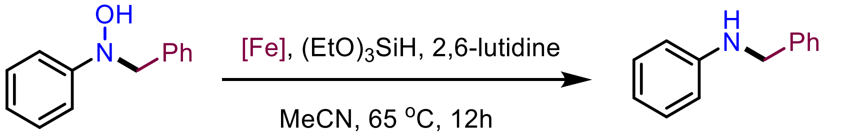

92 **93**

To an 8 mL vial equipped with a stir bar was added hydroxylamine **92** (0.1 mmol, 1.0 equiv.), 2,4-lutidine (0.1 mmol, 1.0 equiv.), $(EtO)_3SiH$ (0.4 mmol, 4.0 equiv.). Three such vials were prepared in parallel. One without catalyst, another with FeI_2 (10 mol%), and the last with FeI_2 (10 mol%) and ligand **L1** (10 mol%). After adding MeCN (1.0 ml), these vials were capped and placed under a nitrogen-atmosphere. These vials were stirred at 65℃ for 12 hours. We can get product **93** about 0%, 47% and 94% yields.

4.7.5 Explore the role of iodide ion.

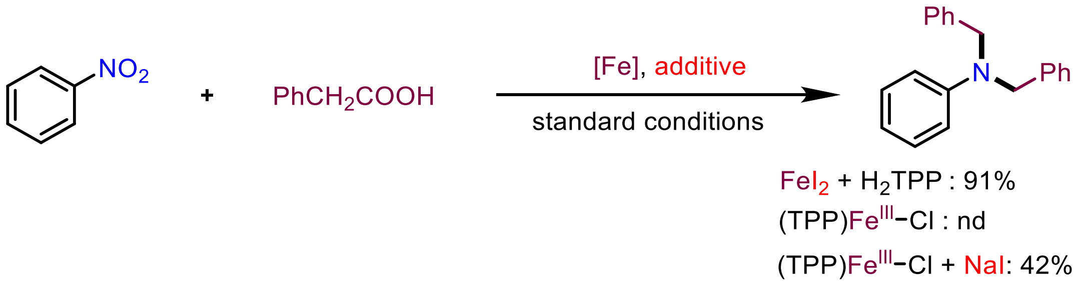

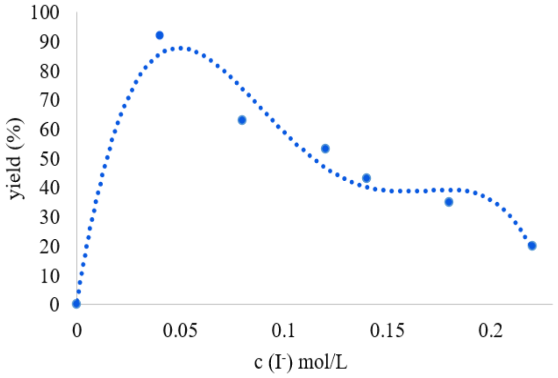

	c (I^-) mol/L	yield (%)
1	0.00	0
2	0.04	92
3	0.08	63
4	0.12	53
5	0.14	43
6	0.18	35
7	0.22	20

Supplementary Figure 11. Yield curve with I^- concentration

We used (TPP)Fe(III)-Cl instead of FeI2 and ligand **L1**, the reaction was not possible, and when we added 50 mmol% NaI, 42% yield was obtained. Through controlled experiments with catalysts and additives, we can see that iodide ions are essential for this reaction. Next, the influence of I^- concentration on the reaction results was obtained by adding different amounts of NaI under standard conditions. The results show that a suitable concentration of I^- can promote the reaction.

4.7.6 Investigation of the role of light

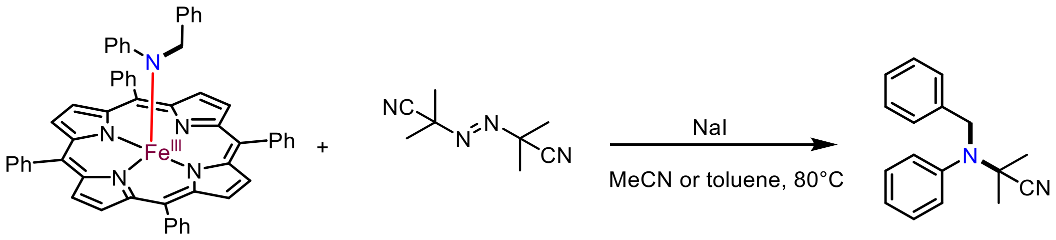

80 AIBN

	without NaI	add NaI
Toluene	nd	nd
MeCN	nd	nd

Supplementary Figure 12. The experiments of **80** with AIBN

5 Derivatization of tertiary amines

3,4-dimethoxy-N-(p-tolyl)benzamide (**64**) [4]

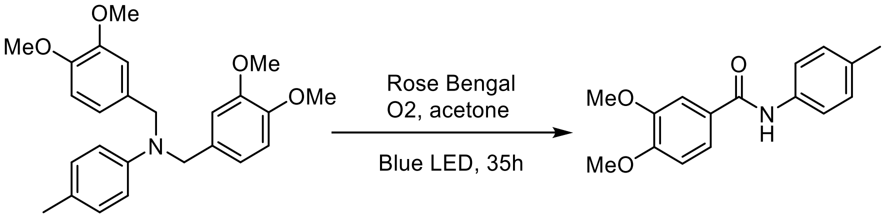

9 64

The reaction tube was charged with tertiary amine **9** (0.2 mmol) and rose bengal (5 mol %) in acetone (6 ml). The reaction mixture was stirred in the presence of O_2 (balloon) under blue light for 35 h. After completion of the reaction, the solvent was evaporated under reduced pressure. The crude product was purified by column chromatography using EA/PE =1:5 as an eluent to furnish the amide compounds **64**, 48.8 mg, 90% yield.

N-benzyl-N-phenylheptanamide (**65**) [5]

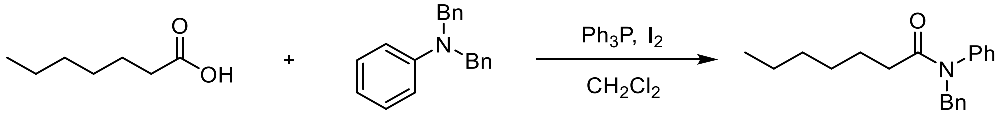

4 65

To a solution of PPh_3 (0.3 mmol) in dichloromethane (1 ml) was added I_2 (0.3 mmol) at 0 °C under N_2. The resulting solution was then added with tertiary amine **4** (0.6 mmol) at 0 °C and continue stirring at this temperature for 10 min. After that, a carboxylic acid (0.2 mmol) was added to the mixture and the solution was allowed to warm up to room temperature and stirred until completion of the reaction. The crude material was purified by column

chromatography using ethyl acetate/hexanes as the eluent to afford pure product **65**, 46.1 mg, 78% yield.

N-benzyl-N-phenyl-2-naphthamide (**66**)[6]

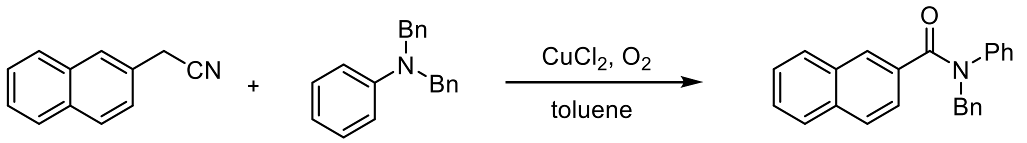

4 **66**

The reaction tube was charged with tertiary amine **4** (0.24 mmol), 2-naphthylacetonitrile (0.20 mmol) and 5 mol% $CuCl_2$, then the reaction tube was evacuated and back-filled with O_2. Toluene (1 ml) were added at room temperature. And the reaction mixture was stirred at 110°C for 24 h. The reaction was monitored by GC or GC-MS. After completion of the reaction, the resulting solution was cooled to room temperature and neutralized with a saturated aqueous solution of NH_4Cl. The product was extracted with EtOAc and the extracts were dried over anhydrous Na_2SO_4 and concentrated in vacuo. The crude product was purified by flash column chromatography on silica gel to give the desired product **66**, 57.3 mg, 85% yield.

2,4,6-triphenyl-1,3,5-triazine (**67**)[7]

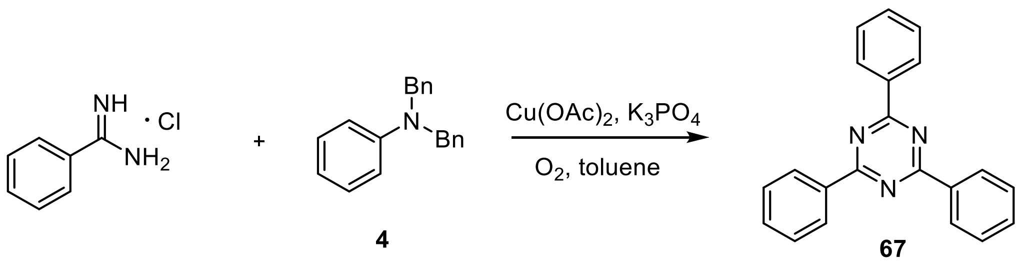

4 **67**

The reaction tube was charged with tertiary amine **4** (0.2 mmol), benzamidine hydrochloride (0.2 mmol), K_3PO_4 (0.4 mmol) and Cu $(OAc)_2$ (5 mol %) in toluene (1 ml). The mixture was stirred under 1 atm O_2 atmosphere at 100 °C for 15 h. After the reaction was completed, 10 ml ethyl acetate (3 × 10 ml) was added into the tube. The combined organic layers were washed with brine to neutral, dried over $MgSO_4$, and concentrated in vacuum. Purification of the residue on a preparative TLC afforded the product **67**, 40.9 mg, 66% yield.

N4,N4,N4',N4'-tetrabenzyl-[1,1'-biphenyl]-4,4'-diamine (**68**)[8]

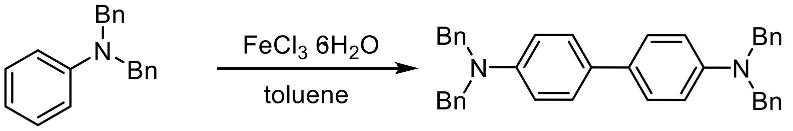

4 **68**

The reaction tube was charged with tertiary amine **4** (0.4 mmol) and $FeCl_3 \cdot 6H_2O$ (0.5 mmol)

in toluene (2 ml). The mixture was stirred at 85 °C for 3 h in the atmosphere. After it was cooled to room temperature, the reaction mixture was quenched by aqueous ammonia solution (mass fraction: 25−28%, 10 ml) and extracted with dichloromethane (10 ml per time) until no product was observed in the extract, as monitored by TLC. The combined extract was washed with water followed by saturated NaCl solution. The organic layer was dried over anhydrous $MgSO_4$, filtered, and concentrated under reduced pressure to give crude product, which was chromatographed on a silica gel column to afford isolated product **68**, 92.5 mg, 85% yield.

N-benzyl-N-phenylbenzamide (**69**)[9]

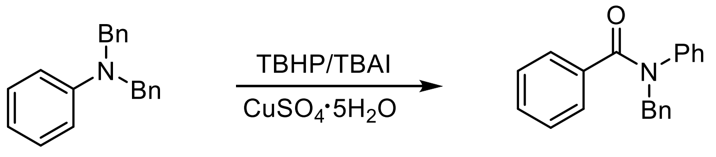

4 **69**

0.2 mmol of amine **4** was dissolved in the mixed solvent composed of 0.5 ml acetonitrile and 0.1 ml water, then add TBHP (70% wt in water, 3.0 equv.), CuSO4·5H_2O (10 mol%) and TBAI (0.3 equiv.) into the mixture, stirring at 80°C for 12h. And the crude product was purified by flash chromatography on silica gel by gradient elution with ethyl acetate in petroleum ether to obtain the amide product **69**, 33.3 mg, 58% yield.

4,4'-methylenebis(N,N-dibenzylaniline) (**70**)[10]

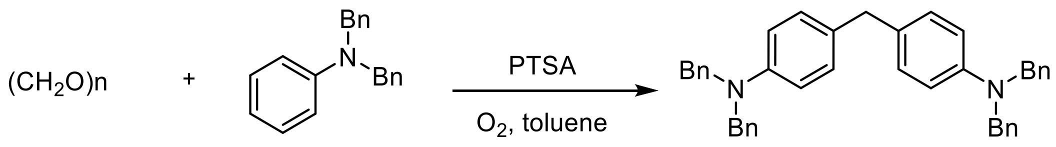

4 **70**

Tertiary amine **4** (0.2 mmol), 0.6 mmol formaldehyde were added into the flask charged with 0.02 mmol PTSA in toluene solvent (2 ml). The mixture was stirred at 80°C for 5 h, then cooled down to room temperature, diluted with 10 ml dichloromethane and washed with 10 ml H_2O. The aqueous layer was extracted twice with dichloromethane (5 ml) and the combined organic phase was dried over anhydrous $MgSO_4$. After evaporation of the solvents, the residue was purified by silica gel chromatography or thin layer chromatography (TLC) to afford isolated product **70**, 33.5 mg, yield 60%.

2-(benzyl(phenyl)amino)-2-phenylacetonitrile (**71**) & *2-(benzyl(phenyl)amino)acetonitrile* (**75**)[11]

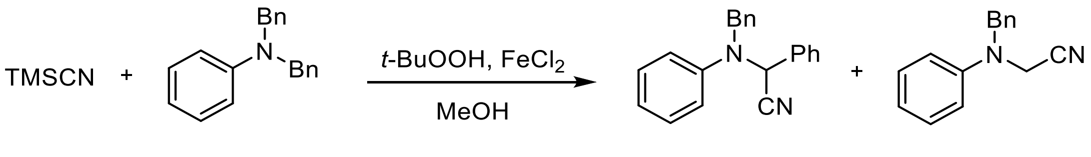

4 **71** **75**

Under an atmosphere of dry N_2, a Schlenk flask was charged with iron (II) chloride (10 mol%) and tertiary amine **4** (0.2 mmol). The trimethylsilyl cyanide (0.4 mmol), and MeOH (1.0 ml) were added successively by syringe. To the mixture was added dropwise tert-butyl hydroperoxide (0.5 mmol, 5.5M solution in decane) over a period of 5 min. The mixture was stirred at room temperature for the indicated time. The crude product was purified by column chromatography on silica gel to get product **71**, 25.0 mg, yield 42%, and to get product **75**, 23.1 mg, yield 52%.

4-((1H-benzo[d][1,2,3]triazol-1-yl)methyl)-N,N-dibenzylaniline (**72**)[10]

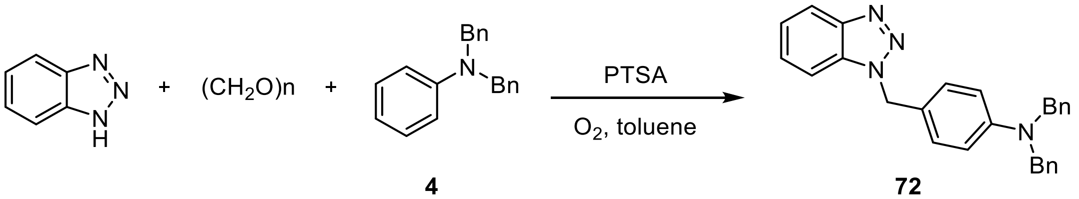

Benzotriazole (0.2 mmol), 0.6 mmol formaldehyde and 0.24 mmol tertiary amine **4** were added into the flask charged with 0.02 mmol PTSA in toluene solvent (2 ml). The mixture was stirred at 80°C for 5 h, then cooled down to room temperature, diluted with 10 ml dichloromethane and washed with 10 ml H_2O. The aqueous layer was extracted twice with dichloromethane (5 ml) and the combined organic phase was dried over anhydrous $MgSO_4$. After evaporation of the solvents, the residue was purified by silica gel chromatography or thin layer chromatography (TLC) to afford isolated product **72**, 64.7 mg, yield 80%.

1-(4-(dibenzylamino)phenyl)-N,N-dimethylnaphthalen-2-amine (**73**)[12]

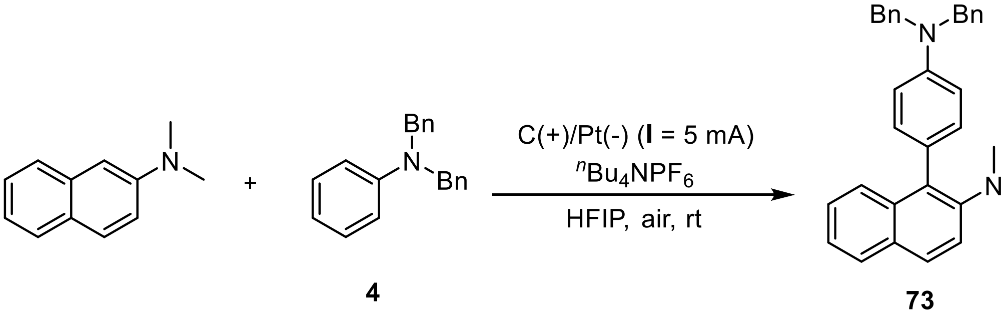

To an undivided three-necked bottle (10 ml) were added N,N-dimethylnaphthalen-2-amine (0.2 mmol), tertiary amine **4** (0.3 mmol) and HFIP (6 ml). The bottle was equipped with platinum electrodes (1.0×1.0 cm^2) as cathode and graphite electrode as anode under air. The reaction mixture was stirred and electrolyzed at a constant current of 5 mA at room temperture for 4 h until complete consumption of **4** as monitored by TLC and/or GC-MS analysis. After the reaction was finished, the solution was extracted with EtOAc (3×10 ml). The combined organic layer was dried with Na_2SO_4, filtered and concentrated in vacuum. The resulting residue was purified by silica gel column chromatography (hexane/ethyl acetate) to afford the desired products **73**, 72.5 mg, yield 82%.

6-(p-tolyl)-6,7-dihydro-5H-dibenzo[c,e]azepine (**74**)[13]

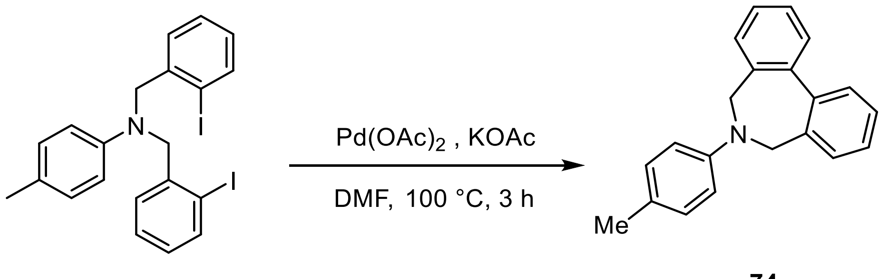

A mixture of tertiary amine **34** (0.2 mmol), Pd $(OAc)_2$ (10 mol%), dppf (the indicated loading), and KOAc (1.0 mmol) was stirred in DMF (1 ml) at 100°C for the indicated reaction time until complete consumption of starting material as monitored by TLC and GC–MS analysis. Then the mixture was washed with saturated NaCl and extracted with diethyl ether. The organic layers were dried with anhydrous Na_2SO_3 and evaporated under vacuum, the residue was purified by flash column chromatography (hexane/ethyl acetate) to afford the pure product **74**, 42.8 mg, yield 75%.

6 Characterization of products

N,N-dibenzylaniline (**4**)

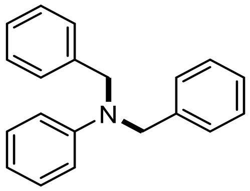

According to the general procedure in 0.2 mmol scale using 1.0 equiv. nitrobenzene with reaction time of 45 h at 25°C and 24h at 65°C; purified by flash chromatography (eluent: PE / EA = 50:1), 44.9 and 49.8 mg, 82% and 91% yield, white solid, m. p. = 69 – 70 °C; R_f = 0.8 (PE / EA = 50:1). **^{1}H NMR** (400 MHz, Chloroform-*d*) δ 7.31 – 6.99 (m, 12H), 6.68 – 6.57 (m, 3H), 4.55 (s, 4H).**^{13}C NMR** (101 MHz, $CDCl_3$) δ 149.1, 138.6, 129.2, 128.6, 126.8, 126.6, 116.7, 112.4, 54.1. **IR (ATR)**: ν = 3030, 3032, 2850, 1597, 1504, 1451, 174, 693, cm^{-1}. **HRMS m/z (ESI)** calcd for $C_{20}H_{20}N$ $(M + H)^+$: 274.1590; found: 274.1584.

N,N-dibenzyl-4-methylaniline (**5**)

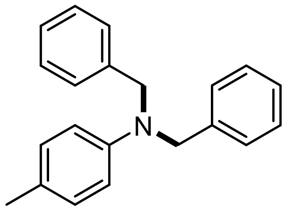

According to the general procedure in 0.2 mmol scale using 1.0 equiv. nitrobenzene with reaction time of 24 h at 65 °C; purified by flash chromatography (eluent: PE / EA = 50:1), 47.7 mg, 83% yield, white solid, m. p. = 55 – 57 °C; R_f = 0.8 (PE / EA = 50:1). **^{1}H NMR** (400 MHz, Chloroform-*d*) δ 7.31 – 7.15 (m, 10H), 6.76 – 6.70 (m, 2H), 6.69 – 6.64 (m, 2H), 4.53

(s, 4H), 3.68 (s, 3H). **^{13}C NMR** (101 MHz, $CDCl_3$) δ 151.6, 143.7, 138.9, 128.5, 126.9, 126.8, 114.7, 114.5, 55.6, 55.1. **IR (ATR)**: ν = 3061, 3027, 2920, 2858, 1520, 1235, 802, 734 cm^{-1}. **HRMS m/z (ESI)** calcd for $C_{21}H_{22}N$ (M + H)$^+$: 288.1747; found: 288.1740.

N,N-dibenzyl-4-(tert-butyl)aniline (**6**)

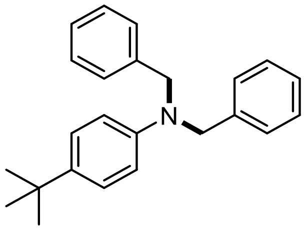

According to the general procedure in 0.2 mmol scale using 1.0 equiv. nitrobenzene with reaction time of 24 h at 65 °C; purified by flash chromatography (eluent: PE / EA = 50:1), 64.6 mg, 98% yield, white solid, m. p. = 71 – 72 °C; R_f = 0.7 (PE / EA = 50:1). **^{1}H NMR** (500 MHz, Chloroform-*d*) δ 7.24 – 7.11 (m, 10H), 7.09 (d, *J* = 8.9 Hz, 2H), 6.60 (d, *J* = 8.9 Hz, 2H), 4.52 (s, 4H), 1.17 (s, 9H). **^{13}C NMR** (126 MHz, $CDCl_3$) δ 147.0, 139.2, 138.9, 128.5, 126.8, 126.7, 125.9, 112.1, 54.3, 33.7, 31.5. **IR (ATR)**: ν = 3085, 3060, 3026, 2963, 2942, 2901, 2858, 1202, 840, 724 cm^{-1}. **HRMS m/z (ESI)** calcd for $C_{24}H_{28}N$ (M + H)$^+$: 330.2216; found: 330.2209.

N,N-dibenzyl-4-(tert-butyl)aniline (**7**)

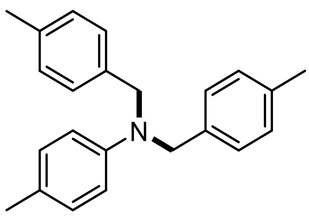

According to the general procedure in 0.2 mmol scale using 1.0 equiv. nitrobenzene with reaction time of 24 h at 25 °C and 65 °C; purified by flash chromatography (eluent: PE / EA = 50:1), 42.8 and 57.3 mg, 68% and 91% yield, colorless solid, m.p. = 75 – 76 °C; R_f = 0.7 (PE / EA = 50:1); **^{1}H NMR** (400 MHz, Chloroform-*d*) δ 7.15 – 7.06 (m, 8H), 6.98 – 6.93 (m, 2H), 6.67 – 6.61 (m, 2H), 4.54 (s, 4H), 2.31 (s, 6H), 2.21 (s, 3H). **^{13}C NMR** (101 MHz, $CDCl_3$) δ = 147.2, 136.3, 135.8, 129.7, 129.2, 126.6, 125.6, 112.6, 54.0, 21.0, 20.2. **IR (ATR)**: ν = 2971, 2919, 2901, 1392, 1236, 1066, 840 ,797, cm^{-1}. **HRMS m/z (ESI)** calcd for $C_{23}H_{26}N$ (M + H)$^+$: 316.2060; found: 316.2052.

N,N-bis(4-fluorobenzyl)-4-methylaniline (**8**).

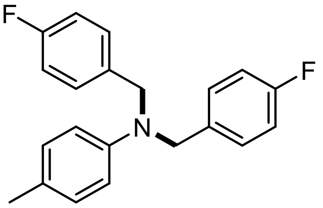

According to the general procedure in 0.2 mmol scale using 1.0 equiv. nitrobenzene with

reaction time of 24 h at 65 °C; purified by flash chromatography (eluent: PE / EA = 50:1), 45.2 mg, 70% yield, yellow oil; R_f = 0.8 (PE / EA = 50:1). **^{1}H NMR** (500 MHz, Chloroform-*d*) δ 7.22 – 7.17 (m, 4H), 7.04 – 6.97 (m, 6H), 6.68 – 6.61 (m, 2H), 4.54 (s, 4H), 2.24 (s, 3H). **^{13}C NMR** (126 MHz, Chloroform-*d*) δ 161.9 (d, *J* = 244.6 Hz), 146.7, 134.2 (d, *J* = 3.0 Hz), 129.8, 128.3 (d, *J* = 8.1 Hz), 126.5, 115.4 (d, *J* = 21.4 Hz), 113.1, 53.8, 20.2. **IR (ATR)**: ν = 3182, 2923, 2862, 1603, 1508, 1014, 820, 803 cm^{-1}. **HRMS m/z (ESI)** calcd for $C_{21}H_{20}F_2N$ (M + H)$^+$: 324.1558; found: 324.1550.

N,N-bis(3,4-dimethoxybenzyl)-4-methylaniline (**9**)

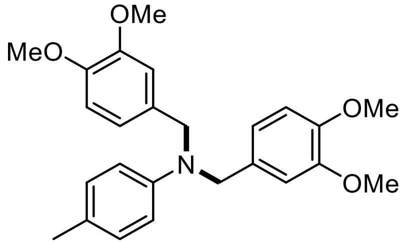

According to the general procedure in 0.2 mmol scale using 1.0 equiv. nitrobenzene with reaction time of 40 h at 25 °C; purified by flash chromatography (eluent: PE / EA = 30:1), 37.2 mg, 46% yield, white solid, m. p. = 83 – 84 °C; R_f = 0.5 (PE / EA = 30:1). **^{1}H NMR** (500 MHz, Chloroform-*d*) δ 7.04 – 6.97 (m, 1H), 6.84 – 6.77 (m, 1H), 6.76 (d, *J* = 2.0 Hz, 0H), 6.74 – 6.70 (m, 1H), 4.52 (s, 1H), 3.86 (s, 2H), 3.81 (s, 2H), 2.24 (s, 1H). **^{13}C NMR** (126 MHz, $CDCl_3$) δ = 149.1, 147.8, 147.3, 131.3, 129.6, 126.1, 118.9, 113.2, 111.1, 110.1, 55.9, 55.8, 54.1, 20.2. **IR (ATR)**: ν = 3180, 2920, 2860, 1612, 1512, 1380, 820, 802, 765 cm^{-1}. **HRMS m/z (ESI)** calcd for $C_{25}H_{30}NO_4$ (M + H)$^+$: 408.2169; found: 408.2163.

2,3-dimethyl-N,N-bis(naphthalen-1-ylmethyl)aniline (**10**)

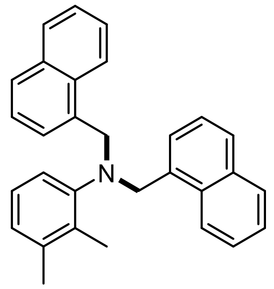

According to the general procedure in 0.2 mmol scale using 1.0 equiv. nitrobenzene with reaction time of 24 h at 65 °C; purified by flash chromatography (eluent: PE / EA = 50:1), 60.2 mg, 75% yield, white solid, m. p. = 81 – 83 °C; R_f = 0.8 (PE / EA = 50:1); **^{1}H NMR** (400 MHz, Chloroform-*d*) δ 7.81 – 7.70 (m, 6H), 7.65 (s, 2H), 7.47 – 7.36 (m, 6H), 7.00 – 6.84 (m, 3H), 4.21 (s, 4H), 2.45 (s, 3H), 2.27 (s, 3H). **^{13}C NMR** (101 MHz, $CDCl_3$) δ = 149.9, 137.9, 136.1, 133.2, 132.6, 132.4, 127.7, 127.6, 127.5, 127.1, 125.8, 125.5, 125.3, 125.3, 120.3, 57.3, 20.8, 14.4. **IR (ATR)**: ν = 3052, 2971, 2919, 1380, 1237, 854, 816, 749 cm^{-1}. **HRMS m/z (ESI)** calcd for $C_{30}H_{28}N$ (M + H)$^+$: 402.2216; found: 402.2204.

2-butyl-N,N-bis(2,5-dimethylbenzyl)aniline (**11**)

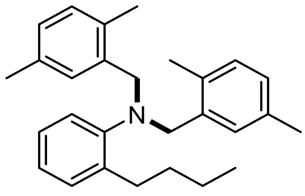

According to the general procedure in 0.2 mmol scale using 1.0 equiv. nitrobenzene with reaction time of 24 h at 65 °C; purified by flash chromatography (eluent: PE / EA = 50:1), 68.5mg, 90% yield, colorless oil; R_f = 0.8 (PE / EA = 50:1). **^{1}H NMR** (400 MHz, Chloroform-*d*) δ 7.20 – 7.14 (m, 1H), 7.14 – 7.07 (m, 4H), 7.03 – 6.99 (m, 1H), 6.98 – 6.94 (m, 2H), 6.92 – 6.87 (m, 2H), 4.03 (s, 4H), 2.60 – 2.54 (m, 2H), 2.22 (s, 6H), 2.12 (s, 6H), 1.44 – 1.27 (m, 4H), 0.89 (t, *J* = 7.0 Hz, 3H). **^{13}C NMR** (101 MHz, $CDCl_3$) δ = 150.3, 139.9, 136.4, 134.7, 133.5, 130.3, 129.9, 129.3, 127.3, 125.9, 124.1, 123.0, 56.5, 33.0, 29.9, 23.2, 20.9, 18.6, 14.0. **IR (ATR)**: ν = 2956, 2919, 2869, 1375, 1086, 875, 807, 738 cm^{-1}. **HRMS m/z (ESI)** calcd for $C_{28}H_{36}N$ $(M + H)^+$: 386.2842; found: 386.2831.

N,N-bis(2,5-dimethylbenzyl)-2-isopropylaniline (**12**)

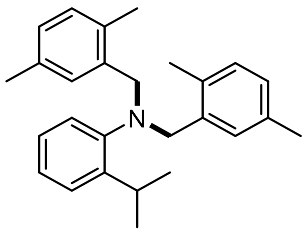

According to the general procedure in 0.2 mmol scale using 1.0 equiv. nitrobenzene with reaction time of 24 h at 65 °C; purified by flash chromatography (eluent: PE / EA = 50:1), 66.9mg, 89% yield, colorless oil; R_f = 0.8 (PE / EA = 50:1). **^{1}H NMR** (400 MHz, Chloroform-*d*) δ 7.33 – 7.25 (m, 1H), 7.17 – 7.08 (m, 2H), 7.10 – 7.01 (m, 1H), 7.01 – 6.92 (m, 4H), 6.92 – 6.85 (m, 2H), 4.02 (s, 4H), 3.59 (hept, *J* = 6.9 Hz, 1H), 2.20 (s, 6H), 2.15 (s, 6H), 0.86 (d, *J* = 6.9 Hz, 6H). **^{13}C NMR** (101 MHz, $CDCl_3$) δ = 149.0, 146.8, 136.4, 134.6, 133.9, 130.9, 129.9, 127.4, 126.2, 125.8, 124.9, 123.6, 57.4, 26.1, 23.9, 20.9, 18.6. **IR (ATR)**: ν = 3030, 2965, 2918, 2865, 1157, 872, 807, 754, cm^{-1}. **HRMS m/z (ESI)** calcd for $C_{27}H_{34}N$ $(M + H)^+$: 372.2686; found: 372.2675.

N,N-dibenzylnaphthalen-1-amine (**13**).

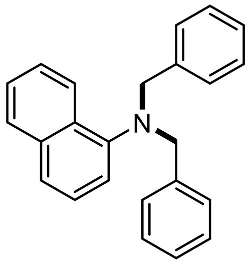

According to the general procedure in 0.2 mmol scale using 1.0 equiv. nitrobenzene with reaction time of 24 h at 65 °C; According to the general procedure in 0.2 mmol scale using 1.0 equiv. nitrobenzene with reaction time of 24 h; purified by flash chromatography (eluent: PE / EA = 50:1), 47.8 mg, 74% yield, yellow oil; R_f = 0.8 (PE / EA = 50:1). **^{1}H NMR** (400

MHz, Chloroform-*d*) δ 8.45 (d, *J* = 8.4 Hz, 1H), 7.79 – 7.70 (m, 1H), 7.50 – 7.35 (m, 3H), 7.25 – 7.07 (m, 11H), 6.83 (dd, *J* = 7.5, 1.1 Hz, 1H), 4.21 (s, 4H). **^{13}C NMR** (101 MHz, $CDCl_3$) δ 147.4, 138.2, 134.9, 129.7, 128.5, 128.4, 128.2, 126.9, 125.7, 125.5, 125.4, 123.7, 123.5, 118.4, 57.1. **IR (ATR)**: ν = 3059, 3027, 2924, 2841, 1203, 769, 773, 737 cm^{-1}. **HRMS m/z (ESI)** calcd for $C_{24}H_{22}N$ (M + H)$^+$: 324.1747; found: 324.1741.

N,N-dibenzyl-4-((4-bromophenyl)thio)aniline (**14**)

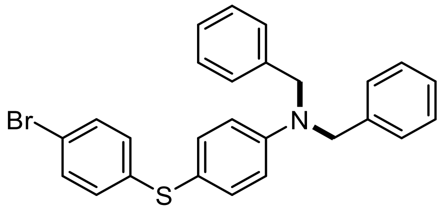

According to the general procedure in 0.2 mmol scale using 1.0 equiv. nitrobenzene with reaction time of 24 h at 65 °C; purified by flash chromatography (eluent: PE / EA = 50:1), 69.9 mg, 76% yield, white solid; m. p. = 80 – 82 °C; R_f = 0.6 (PE / EA = 50:1). **^{1}H NMR** (400 MHz, Chloroform-*d*) δ 7.38 – 7.19 (m, 14H), 7.00 – 6.94 (m, 2H), 6.76 – 6.65 (m, 2H), 4.68 (s, 4H). **^{13}C NMR** (101 MHz, $CDCl_3$) δ 149.7, 139.3, 137.8, 136.1, 131.7, 128.8, 128.6, 127.1, 126.5, 118.7, 117.5, 113.3, 54.2. **IR (ATR)**: ν = 3060, 3029, 2923, 1175, 850, 811, 732, 556 cm^{-1}. **HRMS m/z (ESI)** calcd for $C_{26}H_{23}BrNS$ (M + H)$^+$: 460.0729; found: 460.0721.

N,N-bis(4-(methylthio)benzyl)aniline (**15**)

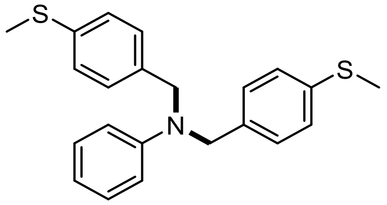

According to the general procedure in 0.2 mmol scale using 1.0 equiv. nitrobenzene with reaction time of 24 h at 65 °C; purified by flash chromatography (eluent: PE / EA = 30:1), 58.4 mg, 80% yield, yellow oil; R_f = 0.5 (PE / EA = 30:1). **^{1}H NMR** (400 MHz, Chloroform-*d*) δ 7.42 – 7.08 (m, 10H), 6.83 – 6.65 (m, 3H), 4.57 (s, 4H), 2.46 (s, 6H). **^{13}C NMR** (101 MHz, $CDCl_3$) δ = 149.0, 136.7, 135.5, 129.2, 127.3, 127.1, 116.9, 112.6, 8.68, 53.9, 16.1. **IR (ATR)**: ν = 2987, 2920, 1598, 1505, 1075, 820, 802, 695 cm^{-1}. **HRMS m/z (ESI)** calcd for $C_{22}H_{24}NS_2$ (M + H)$^+$: 366.1345; found: 366.1331.

N,N-bis(4-phenoxybenzyl)aniline (**16**)

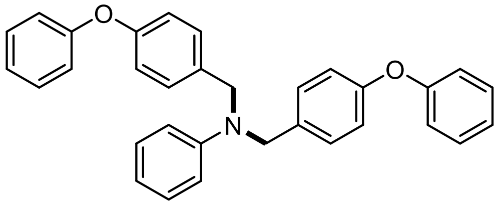

According to the general procedure in 0.2 mmol scale using 1.0 equiv. nitrobenzene with reaction time of 24 h at 65 °C; purified by flash chromatography (eluent: PE / EA = 30:1), 72.2 mg, 79% yield, yellow oil; R_f = 0.5 (PE / EA = 30:1). **^{1}H NMR** (400 MHz, Chloroform-*d*) δ 7.35 – 7.23 (m, 4H), 7.21 – 7.12 (m, 6H), 7.10 – 7.01 (m, 2H), 7.01 – 6.90 (m, 8H), 6.75 (d, *J* = 7.8 Hz, 2H), 6.69 (t, *J* = 7.4 Hz, 1H), 4.57 (s, 1H). **^{13}C NMR** (101 MHz, $CDCl_3$) δ = 157.2, 156.1, 149.0, 133.2, 129.7, 129.2, 128.0, 123.1, 119.0, 118.7, 116.9, 112.6, 53.6. **IR (ATR)**: ν = 3030, 1588, 1503, 1486, 1354, 869, 839, 747 cm^{-1}. **HRMS m/z (ESI)** calcd for $C_{32}H_{28}NO_2$ $(M + H)^+$: 458.2115; found: 458.2103.

N,N-bis(4-bromobenzyl)-4-(methylthio)aniline (**17**)

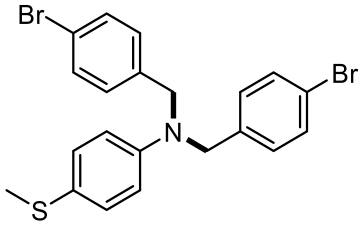

According to the general procedure in 0.2 mmol scale using 1.0 equiv. nitrobenzene with reaction time of 24 h at 65 °C; purified by flash chromatography (eluent: PE / EA = 1:1), 85.0 mg, 89% yield, colorless solid; m. p. = 80 – 81 °C; R_f = 0.7 (PE / EA = 5:1); **^{1}H NMR** (400 MHz, Chloroform-*d*) δ 7.50 – 7.36 (m, 4H), 7.23 – 7.16 (m, 2H), 7.13 – 7.01 (m, 4H), 6.78 – 6.56 (m, 2H), 4.54 (s, 4H), 2.40 (s, 3H). **^{13}C NMR** (101 MHz, $CDCl_3$) δ = 147.3, 137.1, 131.8, 130.9, 128.4, 124.7, 120.8, 113.4, 53.9, 18.6. **IR (ATR)**: ν = 2965, 2921, 1093, 807 cm^{-1}. **HRMS m/z (ESI)** calcd for $C_{21}H_{20}Br_2NS$ $(M + H)^+$: 475.9678; found: 475.9672.

4-(methylthio)-N,N-bis(4-(methylthio)benzyl)aniline (**18**)

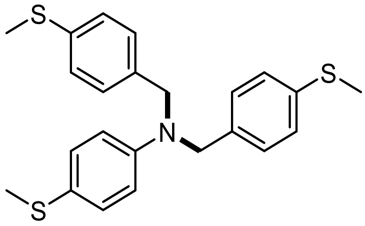

According to the general procedure in 0.2 mmol scale using 1.0 equiv. nitrobenzene with reaction time of 24 h at 65 °C; purified by flash chromatography (eluent: PE / EA = 5:1), 75.6 mg, 92% yield, colorless solid; m. p. = 82 – 84 °C; R_f = 0.8 (PE / EA = 5:1); **^{1}H NMR** (400 MHz, Chloroform-*d*) δ 7.29 – 7.05 (m, 10H), 6.80 – 6.46 (m, 2H), 4.55 (s, 4H), 2.46 (s, 6H), 2.39 (s, 3H). **^{13}C NMR** (101 MHz, $CDCl_3$) δ = 147.7, 136.9, 135.1, 131.1, 127.2, 127.0, 123.9, 113.3, 53.9, 18.8, 16.0. **IR (ATR)**: ν = 3074, 2972, 2916, 1251, 1231, 829, 801 cm^{-1}. **HRMS m/z (ESI)** calcd for $C_{23}H_{26}NS_3$ $(M + H)^+$: 412.1222; found: 412.1213.

N,N-bis(4-fluorobenzyl)-2,3-dihydrobenzofuran-5-amine (**19**)

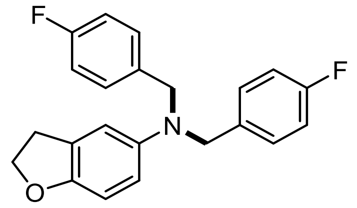

According to the general procedure in 0.2 mmol scale using 1.0 equiv. nitrobenzene with reaction time of 24 h at 65 °C; purified by flash chromatography (eluent: PE / EA = 5:1), 61.8 mg, 88% yield, colorless solid; m. p. = 79 – 81 °C; R_f = 0.7 (PE / EA = 5:1); **^{1}H NMR** (400 MHz, Chloroform-*d*) δ 7.22 – 7.13 (m, 4H), 7.05 – 6.90 (m, 4H), 6.73 – 6.65 (m, 1H), 6.64 – 6.59 (m, 1H), 6.57 – 6.49 (m, 1H), 4.47 (t, *J* = 8.6 Hz, 2H), 4.41 (s, 4H), 3.09 (t, *J* = 8.6 Hz, 2H). **^{13}C NMR** (101 MHz, Chloroform-*d*) δ 161.8 (d, *J* = 244.7 Hz), 152.9, 143.8, 134.4 (d, *J* = 3.1 Hz), 128.6 (d, *J* = 7.9 Hz), 127.8, 115.3 (d, *J* = 21.3 Hz), 114.3, 112.2, 109.2, 70.9, 55.0, 30.4. **IR (ATR)**: ν = 2987, 2971, 2900, 1222, 1066, 823, 726 cm^{-1}. **HRMS m/z (ESI)** calcd for $C_{22}H_{20}F_2NO$ $(M + H)^+$: 352.1507; found: 352.1497.

N,N-bis(4-bromo-3-fluorobenzyl)-2,3-dihydrobenzofuran-5-amine (**20**)

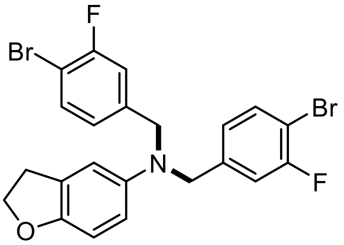

According to the general procedure in 0.2 mmol scale using 1.0 equiv. nitrobenzene with reaction time of 24 h at 65 °C; purified by flash chromatography (eluent: PE / EA = 5:1), 78.9 mg, 78% yield, colorless solid; m. p. = 87 – 89 °C; R_f = 0.7(PE / EA = 5:1); **^{1}H NMR** (400 MHz, Chloroform-*d*) δ 7.47 (dd, *J* = 8.2, 7.0 Hz, 2H), 7.02 (dd, *J* = 9.4, 2.0 Hz, 2H), 6.92 (dd, *J* = 8.2, 1.9 Hz, 2H), 6.66 – 6.58 (m, 2H), 6.49 (dd, *J* = 8.7, 2.7 Hz, 1H), 4.50 (t, *J* = 8.6 Hz, 2H), 4.40 (s, 4H), 3.12 (t, *J* = 8.6 Hz, 2H). **^{13}C NMR** (101 MHz, Chloroform-*d*) δ 159.3 (d, *J* = 247.9 Hz), 153.5, 143.0, 140.8 (d, *J* = 5.8 Hz), 133.6, 128.1, 123.9 (d, *J* = 3.4 Hz), 115.2 (d, *J* = 22.7 Hz), 114.6, 112.4, 109.4, 107.2 (d, *J* = 21.0 Hz), 71.1, 55.3 (d, *J* = 1.7 Hz), 30.4. **IR (ATR)**: ν = 3030, 1578, 1494, 1483, 126, 1038, 809, 745 cm^{-1}. **HRMS m/z (ESI)** calcd for $C_{22}H_{18}Br_2F_2NO$ $(M + H)^+$: 507.9718; found: 507.9716.

N,N-bis(benzo[d][1,3]dioxol-5-ylmethyl)naphthalen-1-amine (**21**)

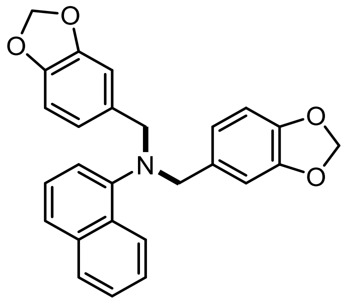

According to the general procedure in 0.2 mmol scale using 1.0 equiv. nitrobenzene with reaction time of 24 h at 65 °C; purified by flash chromatography (eluent: PE / EA = 5:1), 67.4 mg, 82% yield, white solid; m. p. = 86 – 87 °C; R_f = 0.4 (PE / EA = 5:1); **^{1}H NMR** (400 MHz, Chloroform-*d*) δ 8.48 (dd, *J* = 8.4, 1.3 Hz,1H), 7.91 – 7.81 (m, 1H), 7.61 – 7.45 (m, 3H), 7.32 (t, *J* = 7.8 Hz, 1H), 6.96 – 6.86 (m, 1H), 6.79 – 6.77 (m, 2H), 6.75 – 6.59 (m, 4H), 5.92 (s, 4H), 4.19 (s, 4H). **^{13}C NMR** (101 MHz, $CDCl_3$) δ = 147.5, 147.3, 146.5, 134.9, 132.0, 129.6, 128.4, 125.8, 125.5, 125.4, 123.7, 123.6, 121.8, 118.5, 108.9, 107.8, 100.8, 56.6. **IR (ATR)**: ν = 2987, 1687, 1501, 1487, 1038, 930, 808, 775 cm^{-1}. **HRMS m/z (ESI)** calcd for $C_{26}H_{22}NO_4$ (M + H)$^+$: 412.1543; found: 412.1532.

N,N-bis(benzo[d][1,3]dioxol-5-ylmethyl)benzo[d][1,3]dioxol-5-amine (**22**)

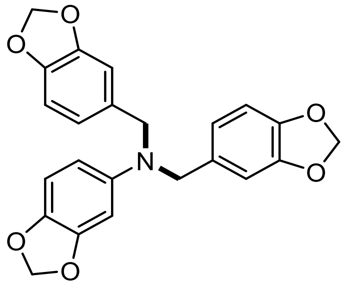

According to the general procedure in 0.2 mmol scale using 1.0 equiv. nitrobenzene with reaction time of 24 h at 65 °C; purified by flash chromatography (eluent: PE / EA = 20:1), 71.3 mg, 88% yield, yellow oil; R_f = 0.8 (PE / EA = 20:1). **^{1}H NMR** (400 MHz, Chloroform-*d*) δ 6.81 – 6.53 (m, 7H), 6.38 (s, 1H), 6.16 (dd, *J* = 8.6, 2.5 Hz, 1H), 5.93 (s, 4H), 5.84 (s, 1H), 4.40 (s, 4H). **^{13}C NMR** (126 MHz, $CDCl_3$) δ = 132.5, 122.4, 122.2, 119.9, 109.7, 108.4, 108.3, 107.4, 105.9, 100.9, 100.6, 96.9, 66.6, 55.1, 40.9. **IR (ATR)**: ν = 2987, 2920, 1503, 1489, 1243, 1039, 932, 810 cm^{-1}. **HRMS m/z (ESI)** calcd for $C_{23}H_{20}NO_6$ (M + H)$^+$: 406.1285; found: 406.1267.

N,N-dibenzyl-4-methoxyaniline (**23**)

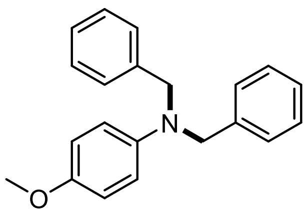

According to the general procedure in 0.2 mmol scale using 1.0 equiv. nitrobenzene with reaction time of 24 h at 65 °C; purified by flash chromatography (eluent: PE / EA = 50:1), 55.3 mg, 91% yield, white solid, m. p. = 81 – 82 °C; R_f = 0.6 (PE / EA = 50:1). **^{1}H NMR** (400 MHz, Chloroform-*d*) δ 7.42 – 7.28 (m, 10H), 7.05 (d, *J* = 8.2 Hz, 2H), 6.74 (d, *J* = 8.6 Hz, 2H), 4.69 (s, 4H), 2.30 (s, 3H).**^{13}C NMR** (100 MHz, $CDCl_3$) δ = 147.0, 138.8, 129.7, 128.5, 126.8, 126.7, 125.8, 112.6, 54.4, 20.2. **IR (ATR)**: ν = 3196, 3062, 3029, 2922, 2852, 1511, 814, 735 cm^{-1}. **HRMS m/z (ESI)** calcd for $C_{21}H_{22}NO$ (M + H)$^+$: 304.1696; found: 304.1689.

N,N-dibenzyl-4-(difluoromethoxy)aniline (**24**)

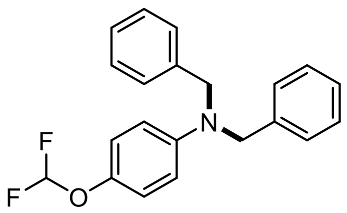

According to the general procedure in 0.2 mmol scale using 1.0 equiv. nitrobenzene with reaction time of 24 h at 65 °C; purified by flash chromatography (eluent: PE / EA = 50:1), 59.5 mg, 88% yield, yellow oil; R_f = 0.7 (PE / EA = 50:1).**^{1}H NMR** (500 MHz, Chloroform-*d*) δ 7.26 – 7.20 (m, 4H), 7.18 – 7.10 (m, 6H), 6.88 – 6.78 (m, 2H), 6.59 – 6.51 (m, 2H), 6.24 (t, *J* = 75 Hz, 1H), 4.53 (s, 4H). **^{13}C NMR** (126 MHz, $CDCl_3$) δ = 147.0, 142.0, 142.0, 141.9, 138.2, 128.7, 127.0, 126.5, 121.2, 118.6, 116.51 (t, *J* = 259.56 Hz), 114.5, 113.1, 54.7. **IR (ATR)**: ν = 3061, 3028, 2923, 2858, 1494, 1221, 1118, 815, 729 cm^{-1}. **HRMS m/z (ESI)** calcd for $C_{21}H_{20}F_2N$ (M + H)$^+$: 340.1507; found: 340.1497.

(4-(dibenzylamino)phenyl)methanol (**25**)

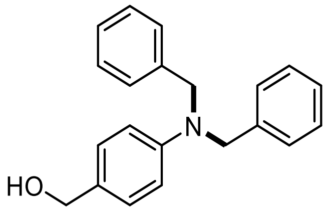

According to the general procedure in 0.2 mmol scale using 1.0 equiv. nitrobenzene with reaction time of 24 h at 65 °C; purified by flash chromatography (eluent: PE / EA = 1:1), 40.0 mg, 66% yield, yellow oil; R_f = 0.2 (PE / EA = 1:1). **^{1}H NMR** (400 MHz, Chloroform-*d*) δ 7.34 – 7.29 (m, 4H), 7.27 – 7.21 (m, 6H), 7.19 – 7.14 (m, 2H), 6.75 – 6.67 (m, 2H), 4.66 (s, 4H), 4.53 (s, 2H). **^{13}C NMR** (101 MHz, $CDCl_3$) δ = 148.8, 138.4, 129.0, 128.8, 128.6, 126.9, 126.6, 112.4, 65.3, 54.3. **IR (ATR)**: ν = 3357, 3060, 3026, 2921, 2864, 1186, 803, 730 cm^{-1}. **HRMS m/z (ESI)** calcd for $C_{21}H_{22}NO$ (M + H)$^+$: 304.1696; found: 304.1689.

2-(4-(bis(4-(methylthio)benzyl)amino)phenyl)ethan-1-ol (**26**)

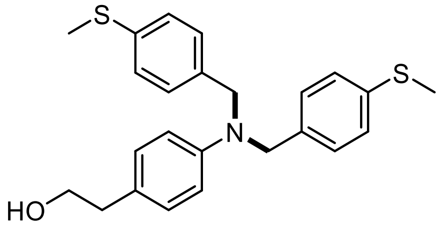

According to the general procedure in 0.2 mmol scale using 1.0 equiv. nitrobenzene with reaction time of 24 h at 65 °C; purified by flash chromatography (eluent: PE / EA = 1:1), 59.7mg, 73% yield, colorless oil; R_f = 0.4 (PE / EA = 1:1). **^{1}H NMR** (400 MHz, Chloroform-*d*) δ 7.24 – 7.17 (m, 4H), 7.16 – 7.10 (m, 4H), 7.08 – 6.93 (m, 2H), 6.74 – 6.60 (m, 2H), 4.54 (s, 4H), 3.76 (t, *J* = 6.5 Hz, 2H), 2.72 (t, *J* = 6.5 Hz, 2H), 2.44 (s, 2H). **^{13}C NMR** (101 MHz, $CDCl_3$) δ = 147.6, 136.7, 135.5, 129.7, 127.2, 127.0, 126.4, 112.8, 63.7, 53.9, 38.0, 16.0. **IR (ATR)**: ν = 3342, 3073, 2971, 2917, 1044, 828, 800 cm^{-1}. **HRMS m/z (ESI)** calcd

for $C_{24}H_{28}NOS_2$ (M + H)$^+$: 410.1607; found: 410.1597.

2-(4-(bis(3,4-dimethoxybenzyl)amino)phenyl)ethan-1-ol (**27**)

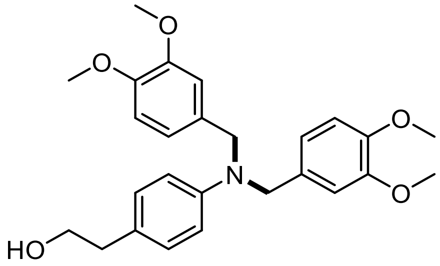

According to the general procedure in 0.2 mmol scale using 1.0 equiv. nitrobenzene with reaction time of 24 h at 65 °C; purified by flash chromatography (eluent: PE / EA = 1:1), 67.5 mg, 77% yield, colorless oil; R_f = 0.3 (PE / EA = 1:1); **^{1}H NMR** (400 MHz, Chloroform-*d*) δ 7.08 – 7.00 (m, 2H), 6.85 – 6.70 (m, 8H), 4.53 (s, 4H), 3.86 (s, 6H), 3.84 – 3.72 (m, 8H), 2.76 (t, *J* = 6.5 Hz, 2H). **^{13}C NMR** (101 MHz, $CDCl_3$) δ = 149.1, 148.2, 147.9, 131.1, 129.7, 126.3, 118.8, 113.2, 111.2, 110.1, 63.8, 55.9, 55.8, 54.1, 38.1. **IR (ATR)**: ν = 3519, 2931, 2835, 1253, 1232, 1026, 857, 807 cm^{-1}. **HRMS m/z (ESI)** calcd for $C_{26}H_{32}NO_5$ (M + H)$^+$: 438.2275; found: 438.2264.

ethyl 2-ethoxy-4-(((4-ethoxy-3-(ethoxycarbonyl)benzyl)(4-(2-hydroxyethyl)phenyl)amino)methyl)benzoate (**28**)

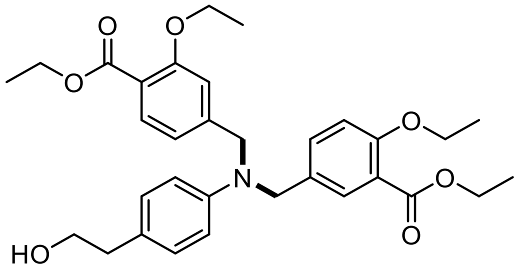

According to the general procedure in 0.2 mmol scale using 1.0 equiv. nitrobenzene with reaction time of 24 h at 65 °C; purified by flash chromatography (eluent: PE / EA = 3:1), 76.9 mg, 70% yield, yellow oil; R_f = 0.5 (PE / EA = 3:1).**^{1}H NMR** (500 MHz, Chloroform-*d*) δ 7.78 – 7.61 (m, 2H), 7.02 (d, *J* = 7.3 Hz, 2H), 6.84 (d, *J* = 8.1 Hz, 2H), 6.80 (s, 2H), 6.69 – 6.62 (m, 2H), 4.57 (s, 4H), 4.33 (q, *J* = 6.0, 4.7 Hz, 4H), 4.00 (q, *J* = 6.9 Hz, 4H), 3.78 (t, *J* = 6.6 Hz, 2H), 2.74 (t, *J* = 6.6 Hz, 2H), 1.45 – 1.31 (m, 12H). **^{13}C NMR** (126 MHz, $CDCl_3$) δ = 166.3, 158.8, 147.4, 144.6, 129.8, 127.2, 119.5, 118.3, 113.3, 113.2, 111.5, 64.5, 63.8, 60.7, 54.8, 38.0, 14.6, 14.2. **HRMS m/z (ESI)** calcd for $C_{32}H_{40}NO_7$ (M + H)$^+$: 550.2799; found: 550.2793.

N,N-dibenzyl-3-bromoaniline (**29**)

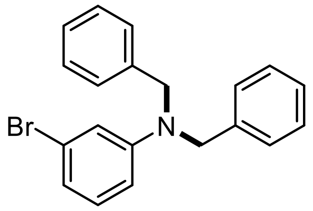

According to the general procedure in 0.2 mmol scale using 1.0 equiv. nitrobenzene with reaction time of 24 h at 65 °C; purified by flash chromatography (eluent: PE / EA = 50:1), 58.7 mg, 83% yield, yellow solid, m. p. = 102 – 103 °C; R_f = 0.6 (PE / EA = 50:1). **^{1}H NMR** (400 MHz, Chloroform-*d*) δ 7.36 – 7.16 (m, 10H), 7.00 – 6.95 (m, 1H), 6.90 – 6.86 (m, 1H), 6.83 – 6.76 (m, 1H), 6.64 – 6.58 (m, 1H), 4.60 (s, 4H). **^{13}C NMR** (101 MHz, $CDCl_3$) δ = 150.4, 137.7, 130.4, 128.7, 127.1, 126.5, 123.5, 119.6, 115.0, 111.0, 54.0. **IR (ATR)**: ν = 2984, 2982, 1181, 758, 741 cm^{-1}. **HRMS m/z (ESI)** calcd for $C_{20}H_{19}BrN$ $(M + H)^+$: 352.0695; found: 352.0687.

N,N-dibenzyl-4-chloroaniline (**30**)

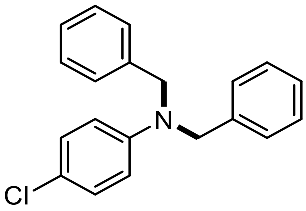

According to the general procedure in 0.2 mmol scale using 1.0 equiv. nitrobenzene with reaction time of 24 h at 65 °C; purified by flash chromatography (eluent: PE / EA = 50:1), 45.5 mg, 74% yield, white solid, m. p. = 104 – 105 °C; R_f = 0.8 (PE / EA = 50:1). **^{1}H NMR** (400 MHz, Chloroform-*d*) δ 7.31 – 7.09 (m, 10H), 7.07 – 6.94 (m, 2H), 6.66 – 6.50 (m, 2H), 4.54 (s, 4H). **^{13}C NMR** (101 MHz, $CDCl_3$) δ = 147.6, 138.0, 129.0, 128.7, 127.0, 126.5, 121.5, 113.6, 54.5. **IR (ATR)**: ν = 3064, 3030, 2925, 2854, 1234, 1177, 859, 816, 787 cm^{-1}. **HRMS m/z (ESI)** calcd for $C_{20}H_{19}ClN$ $(M + H)^+$: 308.1201; found: 308.1196.

N,N-dibenzyl-4-iodoaniline (**31**)

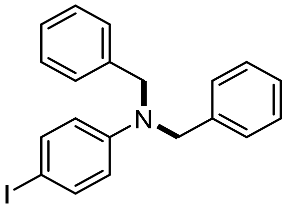

According to the general procedure in 0.2 mmol scale using 1.0 equiv. nitrobenzene with reaction time of 24 h at 65 °C; According to the general procedure in 0.2 mmol scale using 1.0 equiv. nitrobenzene with reaction time of 24 h; purified by flash chromatography (eluent: PE / EA = 50:1), 64.0 mg, 80% yield, white solid, m. p. = 105 – 107 °C; R_f = 0.7 (PE / EA = 50:1). **^{1}H NMR** (400 MHz, Chloroform-*d*) δ 7.41 – 7.36 (m, 2H), 7.35 – 7.18 (m, 10H), 6.53 – 6.47 (m, 2H), 4.62 (s, 4H). **^{13}C NMR** (101 MHz, $CDCl_3$) δ = 148.6, 137.8, 137.7, 128.7, 127.1, 126.5, 114.8, 54.3. **IR (ATR)**: ν = 3057, 3022, 2918, 1237, 801, 729 cm^{-1}. **HRMS m/z (ESI)** calcd for $C_{20}H_{19}IN$ $(M + H)^+$: 400.0557; found: 400.0548.

N,N-bis(4-(trifluoromethyl)benzyl)aniline (**32**)

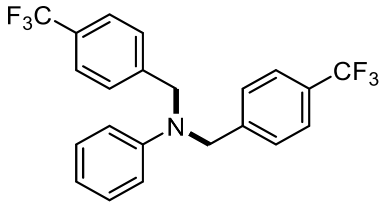

According to the general procedure in 0.2 mmol scale using 1.0 equiv. nitrobenzene with reaction time of 24 h at 65 °C; purified by flash chromatography (eluent: PE / EA = 50:1), 40.9 mg, 50% yield, yellow oil; R_f = 0.6 (PE / EA = 50:1). **^{1}H NMR** (400 MHz, Chloroform-*d*) δ 7.59 (d, *J* = 8.1 Hz, 4H), 7.37 (d, *J* = 8.0 Hz, 4H), 7.24 – 7.15 (m, 2H), 6.81 – 6.75 (m, 1H), 6.73 – 6.67 (m, 2H), 4.70 (s, 4H). **^{13}C NMR** (101 MHz, $CDCl_3$) δ = 148.4, 142.5, 129.5, 126.9, 125.7, 125.7, 125.7, 125.6, 117.8, 112.7, 54.2. **IR (ATR)**: ν = 2987, 2971, 2901, 1324, 825, 749 cm^{-1}. **HRMS m/z (ESI)** calcd for $C_{22}H_{18}F_6N$ $(M + H)^+$: 410.1338; found: 410.1328.

N,N-bis(4-fluorobenzyl)aniline (**33**)

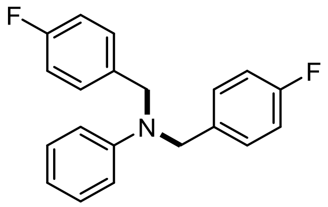

According to the general procedure in 0.2 mmol scale using 1.0 equiv. nitrobenzene with reaction time of 24 h at 65 °C; purified by flash chromatography (eluent: PE / EA = 50:1), 44.5 mg, 72% yield, yellow oil; R_f = 0.8 (PE / EA = 50:1). **^{1}H NMR** (500 MHz, Chloroform-*d*) δ 7.23 – 7.17 (m, 6H), 7.05 – 6.99 (m, 4H), 6.79 – 6.70 (m, 3H), 4.60 (s, 4H). **^{13}C NMR** (126 MHz, Chloroform-*d*) δ 161.9 (d, *J* = 245.0 Hz), 148.8, 133.9 (d, *J* = 3.1 Hz), 129.3, 128.2 (d, *J* = 7.8 Hz), 117.2, 115.5 (d, *J* = 21.4 Hz), 112.7, 53.5. **IR (ATR)**: ν = 2988, 1598, 1504, 1220, 1154, 821, 748 cm^{-1}. **HRMS m/z (ESI)** calcd for $C_{20}H_{18}F_2N$ $(M + H)^+$: 310.1402; found: 310.1394.

N,N-bis(2-iodobenzyl)-4-methylaniline (**34**)

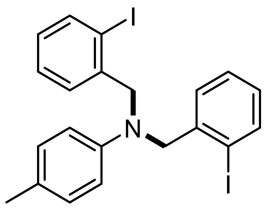

According to the general procedure in 0.2 mmol scale using 1.0 equiv. nitrobenzene with reaction time of 24 h at 65 °C; purified by flash chromatography (eluent: PE / EA = 50:1), 80.8 mg, 75% yield, white solid, m. p. = 148 – 149 °C; R_f = 0.8 (PE / EA = 50:1). **^{1}H NMR** (400 MHz, Chloroform-*d*) δ 7.90 – 7.81 (m, 2H), 7.33 – 7.24 (m, 2H), 7.24 – 7.16 (m, 2H), 7.04 – 6.90 (m, 4H), 6.48 – 6.39 (m, 2H), 4.52 (s, 4H), 2.21 (s, 3H). **^{13}C NMR** (101 MHz, $CDCl_3$) δ = 145.6, 139.6, 139.1, 129.8, 128.8, 128.5, 127.5, 126.3, 112.1, 97.6, 60.6, 20.2.

IR (ATR): ν = 2990, 1517, 1434, 1229, 1010, 801, 744, 511 cm^{-1}. **HRMS m/z (ESI)** calcd for $C_{20}H_{18}F_2N$ (M + H)$^+$: 539.9680; found: 539.9676.

4,4'-((p-tolylazanediyl)bis(methylene))dibenzonitrile (**35**)

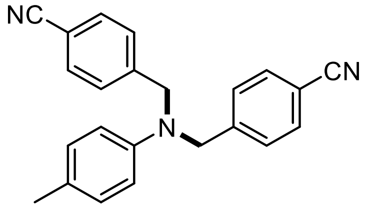

According to the general procedure in 0.2 mmol scale using 1.0 equiv. nitrobenzene with reaction time of 24 h at 65 °C; purified by flash chromatography (eluent: PE / EA = 1:1), 37.1 mg, 55% yield, white solid, m. p. = 90 – 92 °C; R_f = 0.2 (PE / EA = 1:1). **^{1}H NMR** (500 MHz, Chloroform-*d*) δ 7.37 – 7.32 (m, 4H), 7.31 – 7.25 (m, 4H), 6.95 – 6.86 (m, 2H), 6.77 – 6.68 (m, 2H), 4.65 (s, 4H), 2.26 (s, 3H). **^{13}C NMR** (126 MHz, $CDCl_3$) δ = 170.2, 147.1, 141.6, 138.3, 128.6, 126.9, 126.6, 121.9, 112.9, 54.6, 21.1. **IR (ATR)**: ν = cm^{-1}. **HRMS m/z (ESI)** calcd for $C_{23}H_{20}N_3$ (M + H)$^+$: 338.1652; found: 338.1376.

2-(4-(dibenzylamino)phenyl)acetonitrile (**36**)

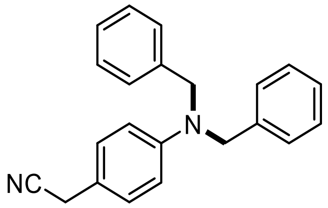

According to the general procedure in 0.2 mmol scale using 1.0 equiv. nitrobenzene with reaction time of 24 h at 65 °C; purified by flash chromatography (eluent: PE / EA = 15:1), 55.5 mg, 89% yield, brown solid, m. p. = 75 – 76 °C; R_f = 0.3 (PE / EA = 15:1). **^{1}H NMR** (400 MHz, Chloroform-*d*) δ 7.35 – 7.28 (m, 4H), 7.28 – 7.16 (m, 6H), 7.10 – 7.02 (m, 2H), 6.74 – 6.65 (m, 2H), 4.64 (s, 4H), 3.57 (s, 2H). **^{13}C NMR** (101 MHz, $CDCl_3$) δ = 148.7, 138.1, 128.8, 128.7, 127.0, 126.5, 118.5, 117.3, 112.7, 54.3, 22.6. **IR (ATR)**: ν = 3084, 3060, 3027, 2925, 2852, 2251, 1295, 801, 744 cm^{-1}. **HRMS m/z (ESI)** calcd for $C_{22}H_{21}N_2$ (M + H)$^+$: 313.1699; found: 313.1690.

4-(dibenzylamino)phenyl acetate (**37**)

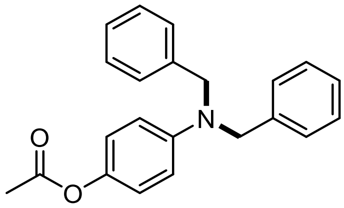

According to the general procedure in 0.2 mmol scale using 1.0 equiv. nitrobenzene with reaction time of 24 h at 65 °C; purified by flash chromatography (eluent: PE / EA = 5:1), 46.5 mg, 70% yield, colorless oil; R_f = 0.5 (PE / EA = 5:1). **^{1}H NMR** (500 MHz, Chloroform-

d) δ 7.36 – 7.29 (m, 4H), 7.27 – 7.21 (m, 6H), 6.90 – 6.83 (m, 2H), 6.70 – 6.64 (m, 2H), 4.62 (s, 4H), 2.23 (s, 3H). **^{13}C NMR** (126 MHz, $CDCl_3$) δ = 170.2, 147.1, 141.5, 138.3, 128.6, 126.9, 126.6, 121.9, 112.8, 54.5, 21.1. **IR (ATR)**: ν = 2987, 2972, 2901, 1753, 1200, 1066, 829, 755 cm^{-1}. **IR (ATR)**: ν = 2987, 2922, 1755, 1508, 1355, 1052, 739, 670 cm^{-1}. **HRMS m/z (ESI)** calcd for $C_{22}H_{22}NO_2$ $(M + H)^+$: 332.1645; found: 332.1639.

6-(dibenzylamino)isobenzofuran-1(3H)-one (**38**)

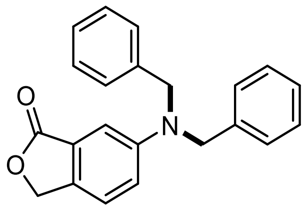

According to the general procedure in 0.2 mmol scale using 1.0 equiv. nitrobenzene with reaction time of 24 h at 65 °C; purified by flash chromatography (eluent: PE / EA = 5:1), 40.1 mg, 61% yield, yellow solid, m. p. = 94 – 95 °C; R_f = 0.2 (PE / EA = 5:1). **^{1}H NMR** (500 MHz, Chloroform-*d*) δ 7.35 – 7.30 (m, 4H), 7.29 – 7.24 (m, 2H), 7.23 – 7.17 (m, 6H), 7.02 (dd, *J* = 8.5, 2.6 Hz, 1H), 5.18 (s, 1H), 4.71 (s, 2H). **^{13}C NMR** (126 MHz, $CDCl_3$) δ = 171.8, 149.9, 137.3, 134.4, 128.8, 127.2, 126.8, 126.4, 122.6, 119.0, 107.2, 69.5, 54.5. **HRMS m/z (ESI)** calcd for $C_{22}H_{20}NO_2$ $(M + H)^+$: 330.1489; found: 330.1481.

methyl 2-(4-(dibenzylamino)phenyl)acetate (**39**)

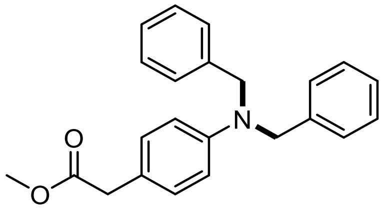

According to the general procedure in 0.2 mmol scale using 1.0 equiv. nitrobenzene with reaction time of 24 h at 65 °C; purified by flash chromatography (eluent: PE / EA = 20:1), 59.6 mg, 86% yield, white solid, m. p. = 93 – 95 °C; R_f = 0.5 (PE / EA = 10:1). **^{1}H NMR** (500 MHz, Chloroform-*d*) δ 7.39 – 7.33 (m, 4H), 7.32 – 7.24 (m, 6H), 7.13 – 7.08 (m, 2H), 6.76 – 6.70 (m, 2H), 4.68 (s, 4H), 3.71 (s, 3H), 3.54 (s, 2H). **^{13}C NMR** (126 MHz, $CDCl_3$) δ = 172.7, 148.2, 138.5, 130.0, 128.6, 126.9, 126.6, 121.8, 112.5, 54.2, 51.9, 40.1. **IR (ATR)**: ν = 3060, 3026, 2948, 2922, 1733, 1227, 1152, 806, 733 cm^{-1}. **HRMS m/z (ESI)** calcd for $C_{23}H_{24}NO_2$ $(M + H)^+$: 346.1802; found: 346.1795.

1-(3-(dibenzylamino)phenyl)ethan-1-one (**40**)

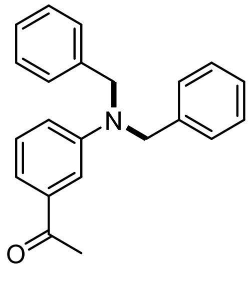

According to the general procedure in 0.2 mmol scale using 1.0 equiv. nitrobenzene with reaction time of 24 h at 65 °C; purified by flash chromatography (eluent: PE / EA = 6:1), 50.4 mg, 80% yield, white solid, m. p. = 90 – 91 °C; R_f = 0.8 (PE / EA = 3:1). **^{1}H NMR** (500 MHz, Chloroform-*d*) δ 7.37 (dd, *J* = 2.9, 1.5 Hz, 1H), 7.34 – 7.29 (m, 4H), 7.27 – 7.18 (m, 8H), 6.90 (ddd, *J* = 8.1, 2.7, 1.1 Hz, 1H), 4.69 (s, 4H), 2.48 (s, 3H). **^{13}C NMR** (126 MHz, $CDCl_3$) δ = 198.6, 149.2, 138.1, 138.0, 129.3, 128.7, 127.1, 126.6, 117.1, 117.0, 111.5, 54.3, 26.7. **IR (ATR)**: ν = 3061, 3027, 3000, 2921, 2854, 1681, 1264, 775, 734 cm^{-1}. **HRMS m/z (ESI)** calcd for $C_{22}H_{22}NO$ (M + H)$^+$: 316.1696; found: 316.1690.

N-(4-(bis(2,5-dimethylbenzyl)amino)phenyl)acetamide (**41**)

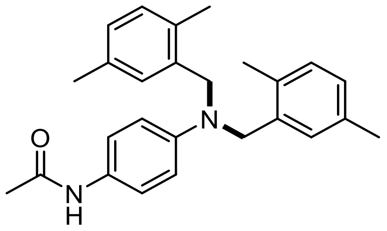

According to the general procedure in 0.2 mmol scale using 1.0 equiv. nitrobenzene with reaction time of 24 h at 65 °C; purified by flash chromatography (eluent: PE / EA = 1:1), 60.3 mg, 78% yield, white solid, m. p. = 156 – 157 °C; R_f = 0.4 (PE / EA = 1:1). **^{1}H NMR** (400 MHz, Chloroform-*d*) δ 7.25 – 7.17 (m, 2H), 7.07 (d, *J* = 7.7 Hz, 2H), 7.02 – 6.93 (m, 4H), 6.54 (d, *J* = 8.4 Hz, 2H), 4.52 (s, 4H), 2.26 (s, 6H), 2.22 (s, 6H), 2.10 (s, 3H). **^{13}C NMR** (101 MHz, $CDCl_3$) δ = 168.2, 146.5, 135.6, 135.4, 132.3, 130.3, 127.8, 127.3, 126.3, 122.5, 112.1, 52.4, 24.1, 21.3, 18.4. **IR (ATR)**: ν = 2985, 2920, 1658, 1600, 1517, 1239, 811, 780 cm^{-1}. **HRMS m/z (ESI)** calcd for $C_{26}H_{31}N_2O$ (M + H)$^+$: 387.2431; found: 387.2428.

N,N-dibenzyl-1H-indol-5-amine (**42**)

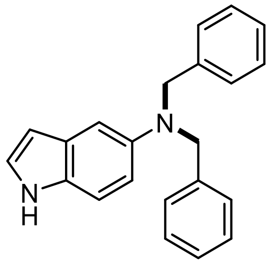

According to the general procedure in 0.2 mmol scale using 1.0 equiv. nitrobenzene with reaction time of 24 h at 65 °C; purified by flash chromatography (eluent: PE / EA = 10:1), 52.9 mg, 85% yield, white solid, m. p. = 120 – 122 °C; R_f = 0.4 (PE / EA = 5:1). **^{1}H NMR** (400 MHz, Chloroform-*d*) δ 7.87 (s, 1H), 7.34 – 7.16 (m, 11H), 7.09 (t, *J* = 2.8 Hz, 1H), 6.98 (s, 1H), 6.82 (dd, *J* = 8.9, 2.4 Hz, 1H), 6.34 (ddt, *J* = 3.0, 1.9, 0.9 Hz, 1H), 4.60 (s, 4H). **^{13}C NMR** (101 MHz, $CDCl_3$) δ = 144.1, 139.4, 129.9, 128.7, 128.4, 127.1, 126.6, 124.5, 112.0, 111.3, 104.3, 102.0, 55.5. **IR (ATR)**: ν = 3345, 2988, 1493, 1451, 1230, 1076, 731, 697 cm^{-1}. **HRMS m/z (ESI)** calcd for $C_{22}H_{21}N_2$ (M + H)$^+$: 313.1699; found: 313.1693.

N,N-dibenzyl-1-methyl-1H-indol-5-amine (**43**)

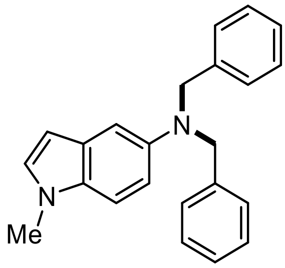

According to the general procedure in 0.2 mmol scale using 1.0 equiv. nitrobenzene with reaction time of 24 h at 65 °C; purified by flash chromatography (eluent: PE / EA = 5:1), 58.7 mg, 90% yield, white solid, m. p. = 125 – 127 °C; R_f = 0.6 (PE / EA = 5:1); **^{1}H NMR** (400 MHz, Chloroform-*d*) δ 7.33 – 7.24 (m, 10H), 7.14 (d, *J* = 8.9 Hz, 1H), 6.97 (d, *J* = 2.4 Hz, 1H), 6.93 (d, *J* = 3.0 Hz, 1H), 6.86 (dd, *J* = 8.9, 2.5 Hz, 1H), 6.26 (dd, *J* = 3.0, 0.9 Hz, 1H), 4.60 (s, 4H), 3.71 (s, 3H). **^{13}C NMR** (101 MHz, $CDCl_3$) δ = 143.7, 139.5, 131.2, 129.2, 129.0, 128.4, 127.2, 126.6, 111.7, 109.5, 104.7, 99.9, 55.6, 32.8. **IR (ATR)**: ν = 2987, 2971, 1406, 1393, 1250, 1066, 850, 690 cm^{-1}. **HRMS m/z (ESI)** calcd for $C_{23}H_{23}N_2$ $(M + H)^+$: 327.1856; found: 327.1851.

N,N-dibenzyl-4-morpholinoaniline (**44**)

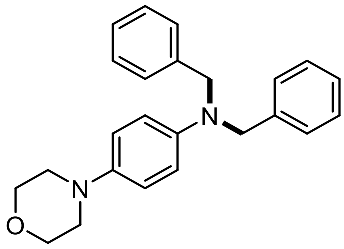

According to the general procedure in 0.2 mmol scale using 1.0 equiv. nitrobenzene with reaction time of 24 h at 65 °C; purified by flash chromatography (eluent: PE / EA = 6:1), 53.4 mg, 75% yield, yellow solid, m. p. = xxx – xxx °C; R_f = 0.6 (PE / EA = 3:1). **^{1}H NMR** (500 MHz, Chloroform-*d*) δ 7.36 – 7.28 (m, 4H), 7.26 – 7.21 (m, 6H), 6.80 (d, *J* = 8.5 Hz, 2H), 6.71 (d, *J* = 8.5 Hz, 2H), 4.57 (s, 4H), 3.82 (t, *J* = 4.3 Hz, 4H), 3.00 (t, *J* = 4.7 Hz, 4H). **^{13}C NMR** (126 MHz, $CDCl_3$) δ = 143.9, 142.8, 138.9, 128.5, 126.8, 126.8, 117.9, 114.0, 67.1, 54.7, 50.9. **IR (ATR)**: ν = 3083, 3026, 2961, 2890, 2856, 2826, 1199, 1175, 850, 730 cm^{-1}. **HRMS m/z (ESI)** calcd for $C_{24}H_{27}N_2O$ $(M + H)^+$: 359.2118; found: 359.2108.

N,N-dibenzyl-4-(4,4,5,5-tetramethyl-1,3,2-dioxaborolan-2-yl)aniline (**45**)

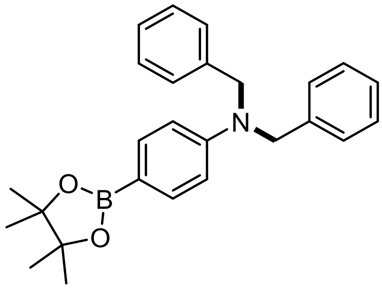

According to the general procedure in 0.2 mmol scale using 1.0 equiv. nitrobenzene with reaction time of 24 h at 65 °C; purified by flash chromatography (eluent: PE / EA = 5:1),

57.0 mg, 71% yield, white solid, m. p. = 79 – 80 °C; R_f = 0.5 (PE / EA = 5:1); **^{1}H NMR** (500 MHz, Chloroform-*d*) δ 7.68 (d, *J* = 8.5 Hz, 2H), 7.40 – 7.31 (m, 4H), 7.32 – 7.23 (m, 6H), 6.78 (d, *J* = 8.3 Hz, 2H), 4.71 (s, 4H), 1.34 (s, 12H). **^{13}C NMR** (126 MHz, $CDCl_3$) δ = 151.4, 138.0, 136.3, 128.6, 126.9, 126.6, 111.3, 83.1, 53.6, 24.8. **IR (ATR)**: ν = 2974, 2925, 1603, 1356, 1094, 1047, 880, 729 cm^{-1}. **HRMS m/z (ESI)** calcd for $C_{26}H_{31}BNO_2$ (M + H)$^+$: 400.2442; found: 400.2433.

N,N-dibenzyl-3-ethynylaniline (**46**)

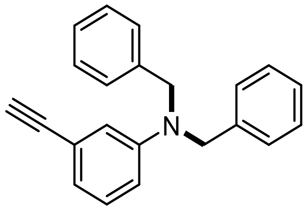

According to the general procedure in 0.2 mmol scale using 1.0 equiv. nitrobenzene with reaction time of 24 h at 65 °C; purified by flash chromatography (eluent: PE / EA = 50:1), 37.2 mg, 63% yield, white solid, m. p. = 75 – 77 °C; R_f = 0.6 (PE / EA = 50:1). **^{1}H NMR** (500 MHz, Chloroform-*d*) δ 7.36 – 7.30 (m, 4H), 7.28 – 7.18 (m, 6H), 7.09 (dd, *J* = 8.4, 7.5 Hz, 1H), 6.89 (dd, *J* = 2.8, 1.3 Hz, 1H), 6.84 (dt, *J* = 7.5, 1.2 Hz, 1H), 6.71 (ddd, *J* = 8.5, 2.8, 0.9 Hz, 1H), 4.63 (s, 4H), 2.94 (s, 1H). **^{13}C NMR** (126 MHz, $CDCl_3$) δ = 149.0, 138.0, 129.2, 128.7, 128.6, 127.0, 126.6, 122.7, 120.7, 115.6, 113.2, 84.4, 76.1, 53.9. **IR (ATR)**: ν = 3287, 3083, 3061, 3027, 2922, 2853, 2204, 1327, 774, 732 cm^{-1}. **HRMS m/z (ESI)** calcd for $C_{22}H_{20}N$ (M + H)$^+$: 298.1590; found: 298.1585.

N,N-dibenzyl-2-methoxypyridin-3-amine (**47**)

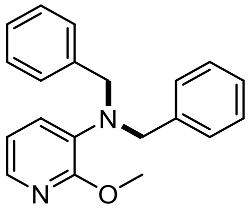

According to the general procedure in 0.2 mmol scale using 1.0 equiv. nitrobenzene with reaction time of 24 h at 65 °C; purified by flash chromatography (eluent: PE / EA = 10:1), 44.2 mg, 73% yield, colorless oil; R_f = 0.8 (PE / EA = 5:1). **^{1}H NMR** (400 MHz, Chloroform-*d*) δ 7.72 (dd, *J* = 4.9, 1.6 Hz, 1H), 7.29 – 7.17 (m, 10H), 6.90 (dd, *J* = 7.6, 1.6 Hz, 1H), 6.66 (dd, *J* = 7.7, 4.9 Hz, 1H), 4.27 (s, 4H), 4.05 (s, 3H). **^{13}C NMR** (101 MHz, $CDCl_3$) δ = 157.3, 138.3, 138.3, 134.3, 128.3, 128.2, 127.3, 126.9, 116.7, 55.0, 53.2. **HRMS m/z (ESI)** calcd for $C_{20}H_{21}N_2O$ (M + H)$^+$: 305.1648; found: 305.1642.

N,N-bis(4-(benzyloxy)benzyl)-2-methoxypyridin-3-amine (**48**)

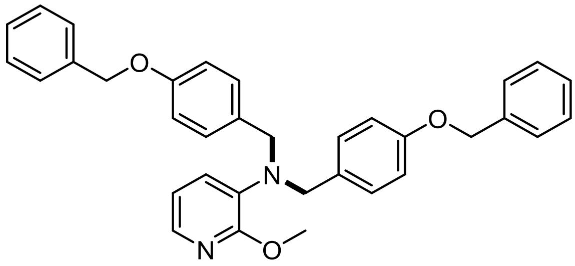

According to the general procedure in 0.2 mmol scale using 1.0 equiv. nitrobenzene with reaction time of 24 h at 65 °C; purified by flash chromatography (eluent: PE / EA = 5:1), 84.7 mg, 82% yield, white solid, m. p. = 82 – 85 °C; R_f = 0.5 (PE / EA = 30:1). **^{1}H NMR** (400 MHz, Chloroform-*d*) δ 7.76 (dd, *J* = 4.9, 1.6 Hz, 1H), 7.56 – 7.29 (m, 10H), 7.29 – 7.11 (m, 4H), 7.00 – 6.84 (m, 5H), 6.70 (dd, *J* = 7.6, 4.9 Hz, 1H), 5.04 (s, 4H), 4.21 (s, 4H), 4.08 (s, 3H). **^{13}C NMR** (101 MHz, $CDCl_3$) δ = 157.8, 157.4, 138.2, 137.0, 134.3, 130.6, 129.6, 128.5, 127.9, 127.5, 127.4, 116.7, 114.5, 70.0, 54.1, 53.2. **IR (ATR)**: ν = 2987, 2920, 1581, 1509, 1240, 1015, 737, 696 cm^{-1}. **HRMS m/z (ESI)** calcd for $C_{34}H_{33}N_2O_3$ $(M + H)^+$: 517.2486; found: 517.2479.

N-benzyl-N-(4-methoxybenzyl)aniline (**49**)

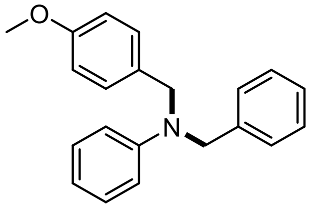

According to the general procedure in 0.2 mmol scale using 1.0 equiv. nitrobenzene with reaction time of 24 h at 65 °C; purified by flash chromatography (eluent: PE / EA = 30:1), 27.4 mg, 45% yield, colorless oil; R_f = 0.6 (PE / EA = 15:1). **^{1}H NMR** (400 MHz, Chloroform-*d*) δ 7.34 – 7.28 (m, 0H), 7.26 – 7.21 (m, 1H), 7.19 – 7.11 (m, 1H), 6.87 – 6.67 (m, 2H), 4.64 (s, 1H), 4.61 (s, 1H), 3.75 (s, 1H). **^{13}C NMR** (101 MHz, $CDCl_3$) δ = 159.9, 129.6, 129.3, 129.2, 128.6, 126.9, 126.7, 118.9, 116.8, 112.5, 112.4, 112.1, 55.1, 54.3. **IR (ATR)**: ν = 3030, 2920, 1598, 1505, 1259, 1046, 748, 692 cm^{-1}. **HRMS m/z (ESI)** calcd for $C_{21}H_{22}NO$ $(M + H)^+$: 304.1696; found: 304.1690.

N,N-bis(4-methoxybenzyl)aniline (**49a**)

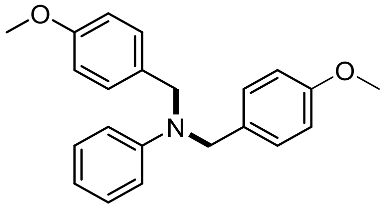

According to the general procedure in 0.2 mmol scale using 1.0 equiv. nitrobenzene with reaction time of 24 h at 65 °C; purified by flash chromatography (eluent: PE / EA = 15:1), 20.6 mg, 31% yield, colorless oil; R_f = 0.4 (PE / EA = 15:1). **^{1}H NMR** (400 MHz, Chloroform-*d*) δ 7.24 – 7.16 (m, 6H), 6.93 – 6.86 (m, 4H), 6.82 – 6.77 (m, 2H), 6.76 – 6.70 (m, 1H),

4.59 (s, 4H), 3.82 (s, 6H). **^{13}C NMR** (101 MHz, $CDCl_3$) δ = 158.5, 149.2, 130.5, 129.1, 127.8, 116.6, 114.0, 112.6, 55.2, 53.4. **IR (ATR)**: ν = 2931, 2834, 1599, 1507, 1244, 1033, 817, 748 cm^{-1}. **HRMS m/z (ESI)** calcd for $C_{21}H_{24}NO_2$ (M + H)$^+$: 334.1802; found: 334.1794.

N,N-dibenzylaniline (**49b**)

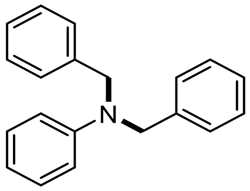

According to the general procedure in 0.2 mmol scale using 1.0 equiv. nitrobenzene with reaction time of 24 h at 65 °C; purified by flash chromatography (eluent: PE / EA = 50:1), 10.9 mg, 20% yield, white solid; R_f = 0.9 (PE / EA = 15:1). Other characterization data refer to product **4**.

N-(3,4-dimethoxybenzyl)-N-(4-fluorobenzyl)aniline (**50**)

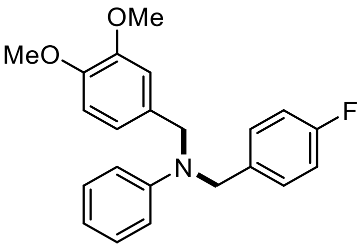

According to the general procedure in 0.2 mmol scale using 1.0 equiv. nitrobenzene with reaction time of 24 h at 65 °C; purified by flash chromatography (eluent: PE / EA = 10:1), 34.4 mg, 49% yield, colorless oil; R_f = 0.5 (PE / EA = 10:1). **^{1}H NMR** (400 MHz, Chloroform-*d*) δ 7.25 – 7.14 (m, 4H), 7.05 – 6.93 (m, 2H), 6.85 – 6.65 (m, 6H), 4.56 (s, 2H), 4.55 (s, 2H), 3.85 (d, *J* = 1.4 Hz, 3H), 3.79 (d, *J* = 1.4 Hz, 3H). **^{13}C NMR** (101 MHz, Chloroform-*d*) δ 161.8 (d, *J* = 244.6 Hz), 149.2, 149.1, 148.0, 134.2 (d, *J* = 3.1 Hz), 130.8, 129.2, 128.2 (d, *J* = 8.0 Hz), 118.8, 117.0, 115.4 (d, *J* = 21.4 Hz), 112.8, 111.2, 110.0, 55.9, 54.0, 53.4, 29.7. **IR (ATR)**: ν = 3062, 1580, 1502, 1240, 820, 743 cm^{-1}. **HRMS m/z (ESI)** calcd for $C_{22}H_{23}FNO_2$ (M + H)$^+$: 352.1707; found: 352.1702.

N,N-bis(3,4-dimethoxybenzyl)aniline (**50a**)

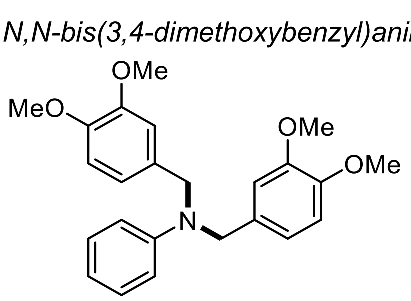

According to the general procedure in 0.2 mmol scale using 1.0 equiv. nitrobenzene with reaction time of 24 h at 65 °C; purified by flash chromatography (eluent: PE / EA = 10:1), 12.6 mg, 16% yield, colorless oil; R_f = 0.4 (PE / EA = 10:1). **^{1}H NMR** (400 MHz, Chloroform-*d*) δ 7.23 – 7.16 (m, 2H), 6.85 – 6.68 (m, 9H), 4.55 (s, 4H), 3.86 (s, 6H), 3.80 (s, 6H). **^{13}C**

NMR (101 MHz, $CDCl_3$) δ = 149.5, 149.2, 147.9, 131.1, 129.1, 118.8, 116.9, 112.9, 111.2, 110.1, 55.9, 55.8, 53.9. **IR (ATR)**: ν = 2926, 1596, 1428, 1289, 1232, 1154, 830, 749, 690 cm^{-1}. **HRMS m/z (ESI)** calcd for $C_{24}H_{28}NO_4$ $(M + H)^+$: 394.2013; found: 394.2010.

N,N-bis(4-fluorobenzyl)aniline (**50b**)

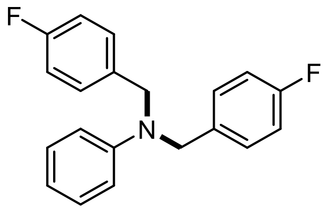

According to the general procedure in 0.2 mmol scale using 1.0 equiv. nitrobenzene with reaction time of 24 h at 65 °C; purified by flash chromatography (eluent: PE / EA = 50:1), 12.4 mg, 20% yield, colorless oil; R_f = 0.9 (PE / EA = 10:1). Other characterization data refer to product **33**.

ethyl 4-(((2,5-dimethylbenzyl)(phenyl)amino)methyl)-2-ethoxybenzoate (**51**)

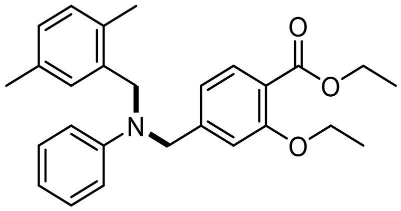

According to the general procedure in 0.2 mmol scale using 1.0 equiv. nitrobenzene with reaction time of 24 h at 65 °C; purified by flash chromatography (eluent: PE / EA = 20:1), 36.7 mg, 44% yield, colorless oil; R_f = 0.5 (PE / EA = 20:1). **^{1}H NMR** (400 MHz, Chloroform-*d*) δ 7.79 – 7.71 (m, 1H), 7.22 – 7.16 (m, 2H), 7.12 – 7.07 (m, 1H), 7.01 (d, *J* = 7.8 Hz, 2H), 6.90 – 6.82 (m, 2H), 6.77 – 6.66 (m, 3H), 4.65 (s, 2H), 4.52 (s, 2H), 4.36 (q, *J* = 7.1 Hz, 2H), 4.02 (q, *J* = 7.0 Hz, 2H), 2.27 (s, 3H), 2.24 (s, 3H), 1.50 – 1.24 (m, 6H). **^{13}C NMR** (101 MHz, $CDCl_3$) δ = 166.3, 158.9, 149.1, 145.0, 135.6, 135.3, 132.4, 131.9, 130.4, 129.2, 127.5, 126.7, 119.3, 118.1, 116.9, 112.4, 111.3, 64.5, 60.6, 54.1, 52.5, 21.2, 18.5, 14.6, 14.3. **IR (ATR)**: ν = 2987, 1724, 1609, 1505, 1253, 1080, 779, 749 cm^{-1}. **HRMS m/z (ESI)** calcd for $C_{27}H_{32}NO_3$ $(M + H)^+$: 418.2377; found: 418.2371.

N,N-bis(2,5-dimethylbenzyl)aniline (**51a, 54a**)

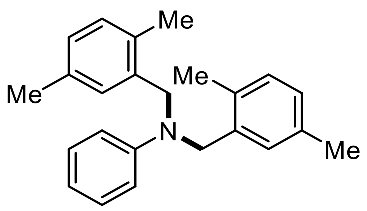

According to the general procedure in 0.2 mmol scale using 1.0 equiv. nitrobenzene with reaction time of 24 h at 65 °C; purified by flash chromatography (eluent: PE / EA = 20:1), 23.1 mg, 35% yield (**51a**) and 13.2 mg, 20% yield (**54a**), white solid, m. p. = 78 – 80 °C; R_f

= 0.9 (PE / EA = 20:1). **^{1}H NMR** (400 MHz, Chloroform-*d*) δ 7.23 – 7.16 (m, 2H), 7.14 – 7.09 (m, 2H), 7.05 – 6.99 (m, 4H), 6.76 – 6.70 (m, 1H), 6.68 – 6.62 (m, 2H), 4.58 (s, 4H), 2.30 (s, 6H), 2.26 (s, 6H). **^{13}C NMR** (101 MHz, $CDCl_3$) δ = 149.1, 135.6, 135.4, 132.3, 130.3, 129.1, 127.3, 126.4, 116.3, 112.1, 52.3, 21.3, 18.5. **IR (ATR)**: ν = 2919, 2860, 1597, 1505, 1264, 808, 732, 530, 438 cm^{-1}. **HRMS m/z (ESI)** calcd for $C_{24}H_{28}N$ $(M + H)^+$: 330.2216; found: 330.2202.

diethyl 4,4'-((phenylazanediyl)bis(methylene))bis(2-ethoxybenzoate) (**51b, 52b**)

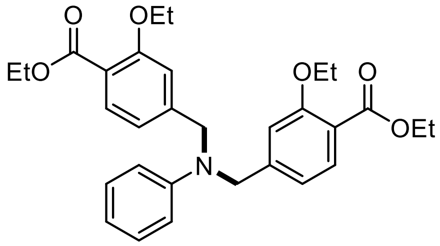

According to the general procedure in 0.2 mmol scale using 1.0 equiv. nitrobenzene with reaction time of 24 h at 65 °C; purified by flash chromatography (eluent: PE / EA = 20:1), 10.1 mg, 10% yield (**51b**) and 12.1 mg, 12% yield (**52b**), colorless oil; R_f = 0.2 (PE / EA = 20:1). **^{1}H NMR** (400 MHz, Chloroform-*d*) δ 7.76 (d, *J* = 7.9 Hz, 2H), 7.24 – 7.15 (m, 2H), 6.91 – 6.82 (m, 4H), 6.81 – 6.71 (m, 3H), 4.63 (s, 4H), 4.37 (q, *J* = 7.1 Hz, 4H), 4.02 (q, *J* = 7.0 Hz, 4H), 1.45 – 1.37 (m, 3H). **^{13}C NMR** (101 MHz, $CDCl_3$) δ = 166.2, 158.8, 147.0, 144.4, 131.9, 129.2, 120.2, 119.5, 118.3, 113.0, 111.5, 64.5, 60.6, 54.7, 37.5, 14.6, 14.2. **IR (ATR)**: ν = 3040, 2980, 2928, 1728, 1112, 1044, 823, 670 cm^{-1}. **HRMS m/z (ESI)** calcd for $C_{30}H_{36}NO_6$ $(M + H)^+$: 506.2537; found: 506.2521.

ethyl 2-ethoxy-4-(((4-(methylthio)benzyl)(phenyl)amino)methyl)benzoate (**52**)

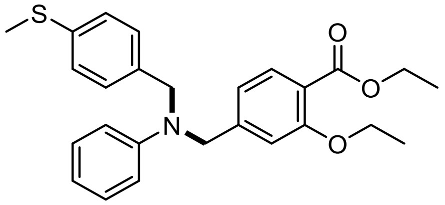

According to the general procedure in 0.2 mmol scale using 1.0 equiv. nitrobenzene with reaction time of 24 h at 65 °C; purified by flash chromatography (eluent: PE / EA = 10:1), 41.8 mg, 48% yield, colorless oil; R_f = 0.5 (PE / EA = 10:1). **^{1}H NMR** (400 MHz, Chloroform-*d*) δ 7.83 – 7.69 (m, 1H), 7.29 – 7.12 (m, 6H), 6.90 – 6.77 (m, 2H), 6.77 – 6.63 (m, 3H), 4.58 (d, *J* = 3.9 Hz, 4H), 4.34 (q, *J* = 7.1 Hz, 2H), 3.99 (q, *J* = 7.0 Hz, 2H), 2.46 (s, 3H), 1.45 – 1.26 (m, 6H). **^{13}C NMR** (101 MHz, $CDCl_3$) δ = 166.3, 158.9, 148.9, 144.8, 136.9, 135.3, 131.9, 129.2, 127.4, 127.0, 119.4, 118.2, 117.3, 112.8, 111.4, 64.5, 60.6, 54.4, 54.1, 16.0, 14.6, 14.3. **IR (ATR)**: ν = 2987, 1724, 1599, 1504, 1286, 1235, 1081, 749 cm^{-1}. **HRMS m/z (ESI)** calcd for $C_{26}H_{30}NO_3S$ $(M + H)^+$: 436.1941; found: 436.1935.

N,N-bis(4-(methylthio)benzyl)aniline (**52a, 54b**)

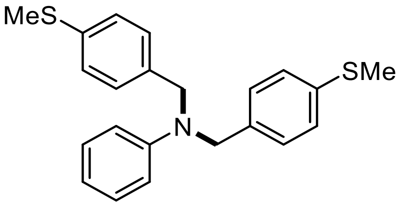

According to the general procedure in 0.2 mmol scale using 1.0 equiv. nitrobenzene with reaction time of 24 h at 65 °C; purified by flash chromatography (eluent: PE / EA = 10:1), 21.2 mg, 29% yield (**52a**) and 19.7 mg, 27% (**54b**), yellow oil; R_f = 0.8 (PE / EA = 10:1). Other characterization data refer to product **15.**

N-(4-(benzyloxy)benzyl)-N-(4-chlorobenzyl)aniline (**53**)

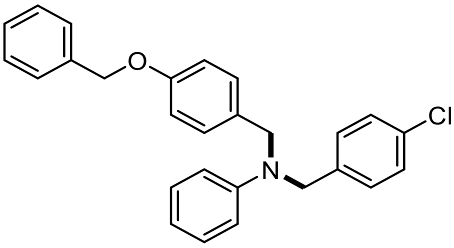

According to the general procedure in 0.2 mmol scale using 1.0 equiv. nitrobenzene with reaction time of 24 h at 65 °C; purified by flash chromatography (eluent: PE / EA = 30:1), 35.5 mg, 43% yield, colorless oil; R_f = 0.5 (PE / EA = 30:1). **^{1}H NMR** (400 MHz, Chloroform-*d*) δ 7.45 – 7.22 (m, 8H), 7.19 – 7.11 (m, 6H), 6.94 – 6.89 (m, 2H), 6.76 – 6.70 (m, 2H), 5.03 (s, 2H), 4.55 (s, 4H). **^{13}C NMR** (101 MHz, $CDCl_3$) δ = 157.9, 148.8, 137.1, 137.0, 132.5, 130.3, 129.2, 128.7, 128.6, 128.1, 128.0, 127.9, 127.4, 117.1, 115.0, 112.8, 70.0, 53.8, 53.6. **IR (ATR)**: ν = 3030, 2987, 1600, 1506, 1233, 989, 808, 747, 694 cm^{-1}. **HRMS m/z (ESI)** calcd for $C_{27}H_{25}ClNO$ $(M + H)^+$: 414.1619; found: 414.1614.

N,N-bis(4-(benzyloxy)benzyl)aniline (**53a**)

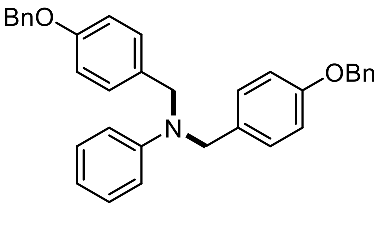

According to the general procedure in 0.2 mmol scale using 1.0 equiv. nitrobenzene with reaction time of 24 h at 65 °C; purified by flash chromatography (eluent: PE / EA = 30:1), 23.5 mg, 25% yield, colorless oil; R_f = 0.3 (PE / EA = 30:1). **^{1}H NMR** (400 MHz, Chloroform-*d*) δ 7.49 – 7.30 (m, 10H), 7.24 – 7.13 (m, 6H), 7.01 – 6.91 (m, 4H), 6.85 – 6.66 (m, 3H), 5.06 (s, 4H), 4.58 (s, 4H). **^{13}C NMR** (101 MHz, $CDCl_3$) δ = 157.8, 149.2, 137.1, 130.8, 129.2, 128.6, 127.9, 127.9, 127.5, 116.6, 115.0, 112.6, 70.1, 53.4. **IR (ATR)**: ν = 2922, 2852, 1599, 1507, 1237, 811, 694 cm^{-1}. **HRMS m/z (ESI)** calcd for $C_{34}H_{32}NO_2$ $(M + H)^+$: 486.2428; found: 486.2413.

N,N-bis(4-chlorobenzyl)aniline (**53b**)

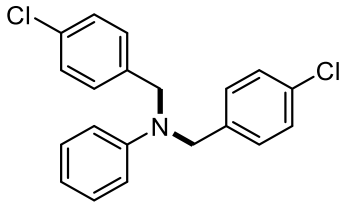

According to the general procedure in 0.2 mmol scale using 1.0 equiv. nitrobenzene with reaction time of 24 h at 65 °C; purified by flash chromatography (eluent: PE / EA = 30:1), 13.6 mg, 20% yield, colorless solid; R_f = 0.9 (PE / EA = 30:1). **^{1}H NMR** (400 MHz, Chloroform-*d*) δ 7.36 – 7.28 (m, 4H), 7.26 – 7.16 (m, 6H), 6.82 – 6.70 (m, 3H), 4.60 (s, 4H). **^{13}C NMR** (101 MHz, $CDCl_3$) δ = 148.6, 136.8, 132.7, 129.3, 128.8, 128.1, 117.4, 112.8, 53.8. **IR (ATR)**: ν = 2924, 1597, 1503, 1236, 1169, 826, 745 cm^{-1}. **HRMS m/z (ESI)** calcd for $C_{20}H_{18}Cl_2N$ $(M + H)^+$: 342.0811; found: 342.0797.

N-(2,5-dimethylbenzyl)-N-(4-(methylthio)benzyl)aniline (**54**)

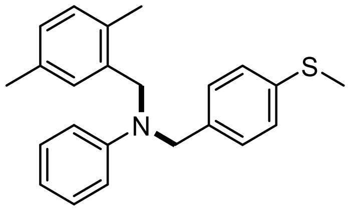

According to the general procedure in 0.2 mmol scale using 1.0 equiv. nitrobenzene with reaction time of 24 h at 65 °C; purified by flash chromatography (eluent: PE / EA = 30:1), 27.9 mg, 40% yield, colorless oil; R_f = 0.2 (PE / EA = 40:1). ^{1}H NMR (400 MHz, Chloroform-*d*) δ 7.24 – 7.20 (m, 2H), 7.19 – 7.13 (m, 4H), 7.09 – 7.05 (m, 1H), 7.00 – 6.96 (m, 2H), 6.70 – 6.65 (m, 3H), 4.61 (s, 2H), 4.50 (s, 2H), 2.46 (s, 3H), 2.25 (s, 3H), 2.21 (s, 3H). ^{13}C NMR (101 MHz, $CDCl_3$) δ = 149.1, 136.6, 135.6, 132.3, 130.3, 129.2, 129.2, 128.2, 127.4, 127.2, 127.1, 126.5, 116.5, 112.2, 53.4, 52.3, 21.3, 18.5, 16.1. **IR (ATR)**: ν = 2987, 2885, 1598, 1504, 1350, 1233, 958, 747 cm^{-1}. **HRMS m/z (ESI)** calcd for $C_{23}H_{26}NS$ $(M + H)^+$: 348.1780; found: 348.1774.

N-(3,5-dimethoxybenzyl)-N-(1-phenylethyl)aniline (**55**)

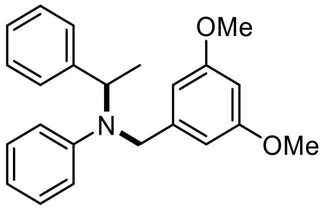

According to the general procedure in 0.2 mmol scale using 1.0 equiv. nitrobenzene with reaction time of 24 h at 65 °C; purified by flash chromatography (eluent: PE / EA = 30:1), 36.1 mg, 52% yield, colorless oil; R_f = 0.4 (PE / EA = 30:1). **^{1}H NMR** (500 MHz, Chloroform-*d*) δ 7.33 – 7.28 (m, 4H), 7.24 – 7.19 (m, 1H), 7.17 – 7.11 (m, 2H), 6.76 (d, *J* = 8.2 Hz, 2H), 6.69 (t, *J* = 7.2 Hz, 1H), 6.41 (d, *J* = 2.3 Hz, 2H), 6.31 – 6.27 (m, 1H), 5.24 (q, *J* = 7.0 Hz, 1H), 4.47 – 4.31 (m, 2H), 3.70 (s, 6H), 1.60 (d, *J* = 7.0 Hz, 3H). **^{13}C NMR** (126 MHz, $CDCl_3$)

δ = 160.8, 149.2, 143.0, 142.8, 128.9, 128.5, 126.9, 126.8, 117.2, 114.2, 104.4, 98.3, 57.0, 55.2, 50.6, 18.6. **IR (ATR)**: ν = 2985, 1596, 1503, 1460, 1203, 1154, 749, 698 cm^{-1}. **HRMS m/z (ESI)** calcd for $C_{23}H_{26}NO_2$ $(M + H)^+$: 348.1958; found: 348.1950.

N-(1-phenylethyl)aniline (**55a, 56a, 57a**)

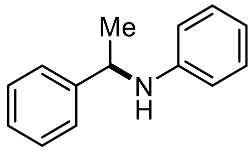

According to the general procedure in 0.2 mmol scale using 1.0 equiv. nitrobenzene with reaction time of 24 h at 65 °C; purified by flash chromatography (eluent: PE / EA = 30:1), 11.0 mg, 28% yield (**55a**); 9.9 mg, 25% yield (**56a**); 5.9 mg, 15% (**57a**), yellow oil; R_f = 0.8 (PE / EA = 30:1). **^{1}H NMR** (400 MHz, Chloroform-*d*) δ 7.41 – 7.30 (m, 4H), 7.26 – 7.21 (m, 1H), 7.16 – 7.03 (m, 2H), 6.74 – 6.61 (m, 1H), 6.57 – 6.44 (m, 2H), 4.50 (q, *J* = 6.7 Hz, 1H), 4.04 (br, 1H), 1.53 (d, *J* = 6.7 Hz, 3H). **^{13}C NMR** (101 MHz, $CDCl_3$) δ 147.2, 145.2, 129.1, 128.6, 126.8, 125.8, 117.2, 113.3, 53.4, 25.0. **IR (ATR)**: ν = 3350, 2960, 1580, 1452, 695 cm^{-1}. **HRMS m/z (ESI)** calcd for $C_{14}H_{16}N$ $(M + H)^+$: 198.1277; found: 198.1265.

N,N-bis(3,5-dimethoxybenzyl)aniline (**55b**)

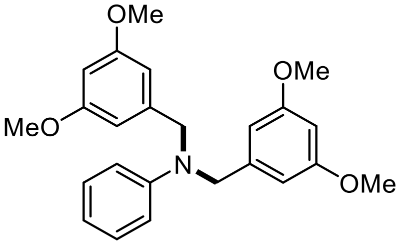

According to the general procedure in 0.2 mmol scale using 1.0 equiv. nitrobenzene with reaction time of 24 h at 65 °C; purified by flash chromatography (eluent: PE / EA = 30:1), 9.4 mg, 12% yield, colorless oil; R_f = 0.2 (PE / EA = 30:1). **^{1}H NMR** (400 MHz, Chloroform-*d*) δ 7.23 – 7.12 (m, 2H), 6.77 – 6.65 (m, 3H), 6.47 – 6.38 (m, 4H), 6.38 – 6.31 (m, 2H), 4.57 (s, 4H), 3.74 (s, 12H). **^{13}C NMR** (101 MHz, $CDCl_3$) δ = 161.1, 149.2, 141.5, 129.1, 116.9, 112.6, 104.6, 98.6, 55.3, 54.6. **IR (ATR)**: ν = 3000, 2920, 2840, 1590, 1427, 1288, 1231, 1150, 810, 680 cm^{-1}. **HRMS m/z (ESI)** calcd for $C_{24}H_{28}NO_4$ $(M + H)^+$: 394.2013; found: 394.1996.

4-((phenyl(1-phenylethyl)amino)methyl)benzonitrile (**56**)

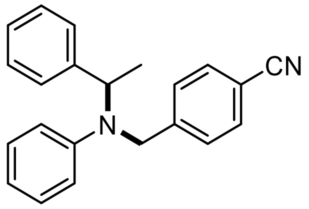

According to the general procedure in 0.2 mmol scale using 1.0 equiv. nitrobenzene with reaction time of 24 h at 65 °C; purified by flash chromatography (eluent: PE / EA = 30:1), 26.2 mg, 42% yield, colorless oil; R_f = 0.4 (PE / EA = 30:1). **^{1}H NMR** (500 MHz, Chloroform-

d) δ 7.61 – 7.53 (m, 2H), 7.37 (d, *J* = 2.9 Hz, 6H), 7.29 (q, *J* = 3.2 Hz, 1H), 7.26 – 7.19 (m, 2H), 6.79 (dd, *J* = 7.7, 5.1 Hz, 3H), 5.34 (q, *J* = 6.9 Hz, 1H), 4.62 – 4.36 (m, 2H), 1.62 (d, *J* = 6.9 Hz, 3H). **^{13}C NMR** (126 MHz, $CDCl_3$) δ = 148.6, 145.9, 142.0, 132.1, 129.2, 128.5, 127.2, 127.1, 127.0, 118.9, 118.0, 114.4, 110.3, 57.2, 49.9, 17.9. **IR (ATR)**: ν = 3032, 2985, 2228, 1598, 1502, 750, 698, 546 cm^{-1}. **HRMS m/z (ESI)** calcd for $C_{22}H_{21}N_2$ $(M + H)^+$: 313.1699; found: 313.1693.

N-(2-iodobenzyl)-4-methyl-N-(1-phenylpropyl)aniline (**57**)

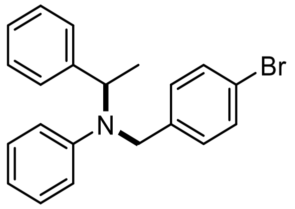

According to the general procedure in 0.2 mmol scale using 1.0 equiv. nitrobenzene with reaction time of 24 h at 65 °C; purified by flash chromatography (eluent: PE / EA = 50:1), 42.4 mg, 58% yield, colorless oil; R_f = 0.7 (PE / EA = 50:1). **^{1}H NMR** (500 MHz, Chloroform-*d*) δ 7.53 – 7.39 (m, 6H), 7.38 – 7.31 (m, 1H), 7.30 – 7.17 (m, 4H), 6.92 – 6.81 (m, 3H), 5.45 – 5.33 (m, 1H), 4.54 – 4.42 (m, 2H), 1.68 (d, *J* = 7.0 Hz, 3H). **^{13}C NMR** (126 MHz, $CDCl_3$) δ = 148.9, 142.4, 139.1, 131.3, 129.1, 128.5, 128.2, 126.9, 126.9, 120.0, 117.5, 114.3, 57.1, 49.6, 18.3. **IR (ATR)**: ν = 3032, 2980, 1596, 1501, 1251, 1027, 748, 697 cm^{-1}. **HRMS m/z (ESI)** calcd for $C_{21}H_{21}BrN$ $(M + H)^+$: 366.0852; found: 366.0845.

N,N-bis(4-bromobenzyl)aniline (**57b**)

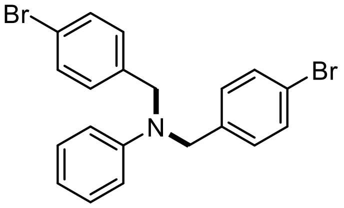

According to the general procedure in 0.2 mmol scale using 1.0 equiv. nitrobenzene with reaction time of 24 h at 65 °C; purified by flash chromatography (eluent: PE / EA = 50:1), 18.9 mg, 22% yield, colorless solid; R_f = 0.9 (PE / EA = 50:1). **^{1}H NMR** (400 MHz, Chloroform-*d*) δ 7.53 – 7.40 (m, 4H), 7.24 – 7.09 (m, 6H), 6.81 – 6.69 (m, 3H), 4.57 (s, 4H). **^{13}C NMR** (101 MHz, $CDCl_3$) δ = 148.5, 137.3, 131.7, 129.3, 128.5, 120.8, 117.5, 112.8, 53.9. **IR (ATR)**: ν = 3041, 2923, 2854, 1598, 1503, 1484, 1009, 804, 692 cm^{-1}. **HRMS m/z (ESI)** calcd for $C_{20}H_{18}Br_2N$ $(M + H)^+$: 429.9801; found: 429.9786.

N-benzyl-N-(1-phenylpropyl)-2,3-dihydrobenzofuran-5-amine (**58**)

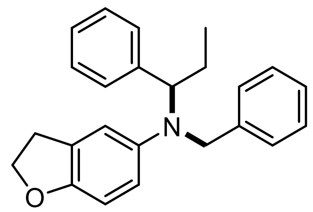

According to the general procedure in 0.2 mmol scale using 1.0 equiv. nitrobenzene with reaction time of 24 h at 65 °C; purified by flash chromatography (eluent: PE / EA = 30:1), 26.8 mg, 39% yield, colorless oil; R_f = 0.5 (PE / EA = 15:1). **^{1}H NMR** (500 MHz, Chloroform-*d*) δ 7.33 – 7.27 (m, 3H), 7.26 – 7.21 (m, 3H), 7.21 – 7.18 (m, 3H), 7.17 – 7.07 (m, 1H), 6.77 (s, 1H), 6.67 – 6.54 (m, 2H), 4.47 (t, *J* = 8.7 Hz, 3H), 4.09 (dd, *J* = 107.9, 15.7 Hz, 2H), 3.09 (t, *J* = 8.6 Hz, 2H), 2.04 – 1.93 (m, 1H), 1.92 – 1.82 (m, 1H), 0.97 (t, *J* = 7.3 Hz, 3H). **^{13}C NMR** (126 MHz, Chloroform-*d*) δ 161.5 (d, *J* = 243.9 Hz), 147.3, 138.6, 137.5, 135.7 (d, *J* = 2.9 Hz), 129.7, 129.2, 127.9 (d, *J* = 7.7 Hz), 127.3, 126.7, 126.3, 126.0, 115.1 (d, *J* = 21.3 Hz), 114.0, 59.1, 50.5, 29.5, 28.8, 22.5, 20.2. **IR (ATR)**: ν = 2959, 2924, 2852, 1492, 1222, 985, 803, 699 cm^{-1}. **HRMS m/z (ESI)** calcd for $C_{24}H_{26}NO$ (M + H)$^+$: 344.2009; found: 344.2000.

N-(1-phenylpropyl)-2,3-dihydrobenzofuran-5-amine (**58a**)

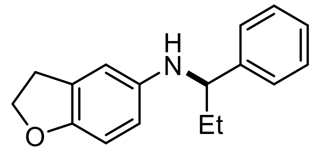

According to the general procedure in 0.2 mmol scale using 1.0 equiv. nitrobenzene with reaction time of 24 h at 65 °C; purified by flash chromatography (eluent: PE / EA = 30:1), 12.7 mg, 25% yield, colorless oil; R_f = 0.3 (PE / EA = 15:1). **^{1}H NMR** (400 MHz, Chloroform-*d*) δ 7.37 – 7.28 (m, 4H), 7.25 – 7.20 (m, 1H), 6.56 – 6.52 (m, 1H), 6.46 – 6.43 (m, 1H), 6.34 – 6.27 (m, 1H), 4.44 (t, *J* = 8.6 Hz, 2H), 4.14 (t, *J* = 6.7 Hz, 1H), 3.78 (br, 1H), 3.11 – 3.02 (m, 2H), 1.90 – 1.72 (m, 2H), 0.95 (t, *J* = 7.4 Hz, 3H). **^{13}C NMR** (101 MHz, $CDCl_3$) δ = 152.2, 144.3, 141.9, 128.4, 127.5, 126.8, 126.5, 112.9, 110.7, 109.2, 70.8, 60.8, 31.7, 30.4, 10.8. **IR (ATR)**: ν = 3345, 3024, 2961, 2925, 1489, 1200, 983, 802, 700 cm^{-1}. **HRMS m/z (ESI)** calcd for $C_{17}H_{20}NO$ (M + H)$^+$: 254.1539; found: 254.1526.

N,N-dibenzyl-2,3-dihydrobenzofuran-5-amine (**58b**)

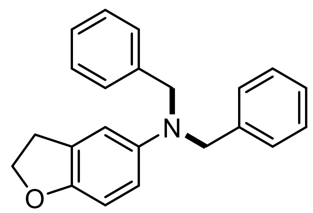

According to the general procedure in 0.2 mmol scale using 1.0 equiv. nitrobenzene with reaction time of 24 h at 65 °C; purified by flash chromatography (eluent: PE / EA = 30:1), 12.6 mg, 20% yield, colorless oil; R_f = 0.6 (PE / EA = 15:1). **^{1}H NMR** (400 MHz, Chloroform-*d*) δ 7.37 – 7.28 (m, 4H), 7.25 – 7.20 (m, 1H), 6.56 – 6.52 (m, 1H), 6.46 – 6.43 (m, 1H),

6.34 – 6.27 (m, 1H), 4.44 (t, *J* = 8.6 Hz, 2H), 4.14 (t, *J* = 6.7 Hz, 1H), 3.78 (br, 1H), 3.11 – 3.02 (m, 2H), 1.90 – 1.72 (m, 2H), 0.95 (t, *J* = 7.4 Hz, 3H). **^{13}C NMR** (101 MHz, $CDCl_3$) δ = 152.2, 144.3, 141.9, 128.4, 127.5, 126.8, 126.5, 112.9, 110.7, 109.2, 70.8, 60.8, 31.7, 30.4, 10.8. **IR (ATR)**: ν = 3060, 3027, 2966, 1492, 1205, 1047, 195, 694 cm^{-1}. **HRMS m/z (ESI)** calcd for $C_{22}H_{22}NO$ (M + H)$^+$: 316.1696; found: 316.1684.

N-(4-fluorobenzyl)-N-(p-tolyl)-1,2,3,4-tetrahydronaphthalen-1-amine (**59**)

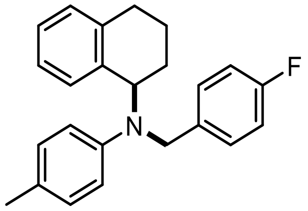

According to the general procedure in 0.2 mmol scale using 1.0 equiv. nitrobenzene with reaction time of 24 h at 65 °C; purified by flash chromatography (eluent: PE / EA = 50:1), 24.2 mg, 35% yield, colorless oil; R_f = 0.7 (PE / EA = 50:1). **^{1}H NMR** (500 MHz, Chloroform-*d*) δ 7.24 – 7.18 (m, 2H), 7.19 – 7.05 (m, 4H), 7.02 – 6.96 (m, 2H), 6.98 – 6.91 (m, 2H), 6.73 – 6.69 (m, 2H), 5.26 – 5.16 (m, 1H), 4.31 – 4.17 (m, 2H), 2.88 – 2.75 (m, 2H), 2.24 (s, 3H), 2.18 – 2.10 (m, 1H), 2.03 – 1.94 (m, 1H), 1.89 – 1.76 (m, 2H). **^{13}C NMR** (126 MHz, $CDCl_3$) δ = 147.3, 138.6, 137.5, 135.7, 129.7, 129.2, 128.0, 127.9, 127.3, 126.7, 126.3, 126.0, 115.1, 115.0, 114.0, 100.0, 59.1, 50.5, 29.5, 28.8, 22.5, 20.2. **IR (ATR)**: ν = 3018, 2986, 2858, 1509, 1343, 1222, 821, 778 cm^{-1}. **HRMS m/z (ESI)** calcd for $C_{24}H_{25}FN$ (M + H)$^+$: 346.1966; found: 346.1958.

N-(p-tolyl)-1,2,3,4-tetrahydronaphthalen-1-amine (**59a**)

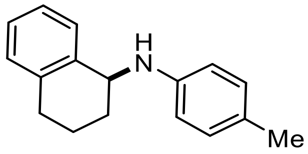

According to the general procedure in 0.2 mmol scale using 1.0 equiv. nitrobenzene with reaction time of 24 h at 65 °C; purified by flash chromatography (eluent: PE / EA = 50:1), 7.1 mg, 15% yield, colorless oil; R_f = 0.5 (PE / EA = 50:1). **^{1}H NMR** (500 MHz, Chloroform-*d*) δ 7.45 – 7.39 (m, 1H), 7.23 – 7.15 (m, 2H), 7.15 – 7.11 (m, 1H), 7.07 – 7.00 (m, 2H), 6.67 – 6.58 (m, 2H), 4.61 (t, *J* = 5.1 Hz, 1H), 2.91 – 2.71 (m, 2H), 2.28 (s, 3H), 2.05 – 1.92 (m, 2H), 1.91 – 1.77 (m, 2H). **^{13}C NMR** (126 MHz, $CDCl_3$) δ = 145.2, 138.4, 137.6, 129.8, 129.2, 129.0, 127.0, 126.3, 126.0, 113.0, 51.3, 29.3, 28.7, 20.3, 19.4. **IR (ATR)**: ν = 3485, 3040, 2980, 1488, 1200, 980, 802, 710 cm^{-1}. **HRMS m/z (ESI)** calcd for $C_{17}H_{20}N$ (M + H)$^+$: 238.1590; found: 238.1588.

N,N-bis(4-fluorobenzyl)-4-methylaniline (**59b**)

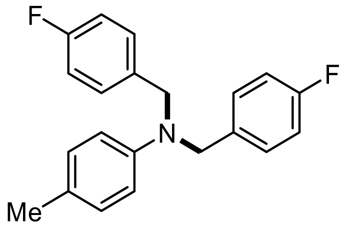

According to the general procedure in 0.2 mmol scale using 1.0 equiv. nitrobenzene with reaction time of 24 h at 65 °C; purified by flash chromatography (eluent: PE / EA = 50:1), 12.9 mg, 20% yield, colorless oil; R_f = 0.9 (PE / EA = 50:1). Other characterization data refer to product **8**.

2-(4-((4-fluorobenzyl)(1-(4-isobutylphenyl)ethyl)amino)phenyl)ethan-1-ol (**60**)

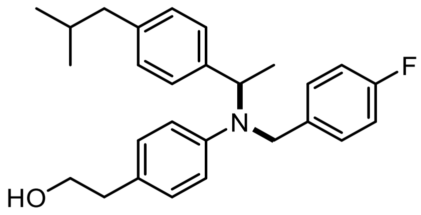

According to the general procedure in 0.2 mmol scale using 1.0 equiv. nitrobenzene with reaction time of 24 h at 65 °C; purified by flash chromatography (eluent: PE / EA = 5:1), 30.8 mg, 38% yield, colorless oil; R_f = 0.4 (PE / EA = 1:1). **^{1}H NMR** (500 MHz, Chloroform-*d*) δ 7.22 (d, *J* = 7.9 Hz, 2H), 7.16 (dd, *J* = 8.5, 5.6 Hz, 2H), 7.08 (d, *J* = 8.1 Hz, 2H), 7.02 (d, *J* = 8.6 Hz, 2H), 6.92 (t, *J* = 8.7 Hz, 2H), 6.74 – 6.69 (m, 2H), 5.21 (q, *J* = 6.9 Hz, 1H), 4.42 – 4.29 (m, 2H), 3.79 (t, *J* = 6.5 Hz, 2H), 2.75 (t, *J* = 6.5 Hz, 2H), 2.44 (d, *J* = 7.2 Hz, 2H), 1.89 – 1.77 (m, 1H), 1.56 (d, *J* = 6.9 Hz, 3H), 0.88 (d, *J* = 6.6 Hz, 6H). **^{13}C NMR** (126 MHz, Chloroform-*d*) δ 161.5 (d, *J* = 243.9 Hz), 147.8, 140.4, 139.7, 135.7 (d, *J* = 3.0 Hz), 129.6, 129.2, 127.9 (d, *J* = 7.9 Hz), 126.8, 126.7, 115.0 (d, *J* = 21.3 Hz), 114.5, 63.8, 56.9, 49.5, 45.0, 38.1, 30.2, 22.3, 18.1. **IR (ATR)**: ν = 3340, 2954, 2925, 1614, 1501, 1220, 1046, 808 cm^{-1}. **HRMS m/z (ESI)** calcd for $C_{27}H_{33}FNO$ $(M + H)^+$: 406.2541; found: 406.2538.

2-(4-((1-(4-isobutylphenyl)ethyl)amino)phenyl)ethan-1-ol (**60a**)

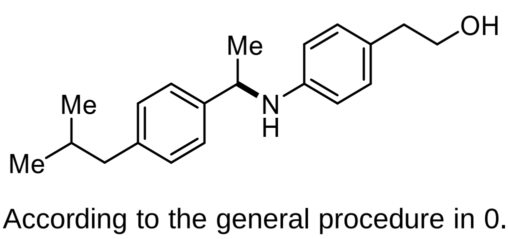

According to the general procedure in 0.2 mmol scale using 1.0 equiv. nitrobenzene with reaction time of 24 h at 65 °C; purified by flash chromatography (eluent: PE / EA = 5:1), 6.0 mg, 10% yield, colorless oil; R_f = 0.2 (PE / EA = 1:1). **^{1}H NMR** (400 MHz, Chloroform-*d*) δ 7.18 (d, *J* = 5.1 Hz, 2H), 7.01 (d, *J* = 7.7 Hz, 2H), 6.89 (d, *J* = 8.1 Hz, 2H), 6.53 (m, 2H), 4.37 (q, *J* = 6.7 Hz, 1H), 3.68 (t, *J* = 6.5 Hz, 2H), 2.65 (t, *J* = 6.5 Hz, 2H), 2.36 (d, *J* = 7.2 Hz, 2H), 1.82 – 1.70 (m, 1H), 1.19 (s, 3H), 0.81 (d, *J* = 6.6 Hz, 6H). **^{13}C NMR** (101 MHz, $CDCl_3$) δ = 129.7, 129.3, 77.3, 77.0, 76.7, 63.8, 45.1, 38.3, 30.2, 22.4, 22.4. The low yield

and concentration of the sample lead to poor peak. **IR (ATR)**: ν = 3356, 3258, 2954, 2922, 2852, 1517, 1316, 1010, 769, 670 cm^{-1}. **HRMS m/z (ESI)** calcd for $C_{20}H_{28}NO$ $(M + H)^+$: 298.2165; found: 298.2151.

2-(4-(bis(4-fluorobenzyl)amino)phenyl)ethan-1-ol (**60b**)

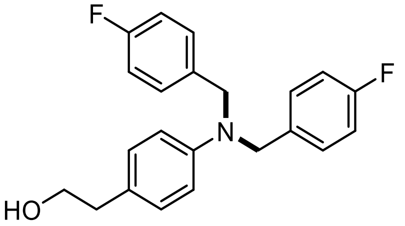

According to the general procedure in 0.2 mmol scale using 1.0 equiv. nitrobenzene with reaction time of 24 h at 65 °C; purified by flash chromatography (eluent: PE / EA = 5:1), 10.6 mg, 15% yield, colorless oil; R_f = 0.5 (PE / EA = 1:1). **^{1}H NMR** (400 MHz, Chloroform-*d*) δ 7.25 – 7.16 (m, 4H), 7.09 – 6.96 (m, 6H), 6.73 – 6.67 (m, 2H), 4.57 (s, 4H), 3.80 (t, *J* = 6.5 Hz, 2H), 2.76 (t, *J* = 6.5 Hz, 2H). **^{13}C NMR** (101 MHz, $CDCl_3$) δ = 163.1, 160.7, 147.5, 134.0, 129.8, 128.3, 128.2, 126.8, 115.6, 115.3, 113.0, 63.8, 53.7, 38.1. **IR (ATR)**: ν = 3352, 2928, 2860, 1602, 1505, 1216, 1043, 808, 527 cm^{-1}. **HRMS m/z (ESI)** calcd for $C_{22}H_{22}F_2NO$ $(M + H)^+$: 354.1664; found: 354.1652.

N-(2-iodobenzyl)-N-(1-(4-isobutylphenyl)ethyl)-2,3-dihydrobenzofuran-5-amine (**61**)

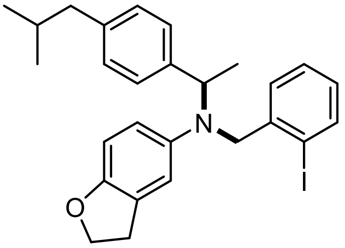

According to the general procedure in 0.2 mmol scale using 1.0 equiv. nitrobenzene with reaction time of 24 h at 65 °C; purified by flash chromatography (eluent: PE / EA = 20:1), 60.3 mg, 59% yield, colorless oil; R_f = 0.5 (PE / EA = 15:1). **^{1}H NMR** (400 MHz, Chloroform-*d*) δ 7.76 (dd, *J* = 7.9, 1.2 Hz, 1H), 7.32 (dd, *J* = 7.8, 1.7 Hz, 1H), 7.28 – 7.18 (m, 3H), 7.08 (d, *J* = 8.1 Hz, 2H), 6.88 (td, *J* = 7.6, 1.8 Hz, 1H), 6.67 – 6.62 (m, 1H), 6.60 (d, *J* = 8.6 Hz, 1H), 6.48 (dd, *J* = 8.7, 2.7 Hz, 1H), 4.98 (q, *J* = 6.9 Hz, 1H), 4.47 (t, *J* = 8.6 Hz, 2H), 4.30 (d, *J* = 17.4 Hz, 1H), 4.16 (d, *J* = 17.4 Hz, 1H), 3.09 (t, *J* = 8.6 Hz, 2H), 2.44 (d, *J* = 7.2 Hz, 2H), 1.93 – 1.76 (m, 1H), 1.51 (d, *J* = 6.9 Hz, 3H), 0.88 (d, *J* = 6.6 Hz, 6H). **^{13}C NMR** (126 MHz, $CDCl_3$) δ = 153.0, 143.3, 141.2, 140.2, 140.1, 138.9, 129.1, 129.1, 128.2, 128.0, 127.4, 126.8, 115.9, 113.5, 109.0, 97.8, 58.9, 57.0, 45.0, 30.4, 30.2, 22.4, 18.3. **IR (ATR)**: ν = 3030, 2988, 2850, 1495, 1437, 1222, 11011, 749 cm^{-1}. **HRMS m/z (ESI)** calcd for $C_{27}H_{31}INO$ $(M + H)^+$: 512.1445; found: 512.1441.

N-(1-(4-isobutylphenyl)ethyl)-2,3-dihydrobenzofuran-5-amine (**61a**)

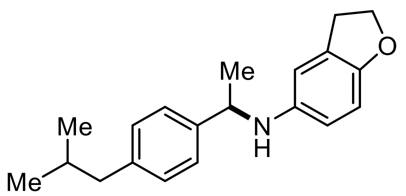

According to the general procedure in 0.2 mmol scale using 1.0 equiv. nitrobenzene with reaction time of 24 h at 65 °C; purified by flash chromatography (eluent: PE / EA = 20:1), 8.3 mg, 14% yield, colorless oil; R_f = 0.3 (PE / EA = 15:1). **^{1}H NMR** (400 MHz, Chloroform-*d*) δ 7.29 (d, *J* = 8.0 Hz, 2H), 7.11 (d, *J* = 8.0 Hz, 2H), 6.58 (d, *J* = 8.4 Hz, 1H), 6.50 (s, 1H), 6.41 – 6.32 (m, 1H), 4.47 (t, *J* = 8.6 Hz, 2H), 4.40 (q, *J* = 6.7 Hz, 1H), 3.09 (t, *J* = 8.5 Hz, 2H), 2.47 (d, *J* = 7.2 Hz, 2H), 1.95 – 1.80 (m, 1H), 1.53 (d, *J* = 6.7 Hz, 3H), 0.92 (d, *J* = 6.6 Hz, 6H). **^{13}C NMR** (101 MHz, $CDCl_3$) δ = 152.6, 142.3, 140.2, 129.3, 127.5, 125.7, 113.5, 111.8, 111.3, 109.2, 70.8, 54.7, 45.1, 30.3, 30.2, 24.7, 22.4, 22.4. **IR (ATR)**: ν = 3350, 2953, 2922, 2866, 1489, 1202, 984, 797, 549 cm^{-1}. **HRMS m/z (ESI)** calcd for $C_{20}H_{26}NO$ (M + H)$^+$: 296.2009; found: 296.1992.

N-benzyl-N-(2-iodobenzyl)-2,3-dihydrobenzofuran-5-amine (**61b**)

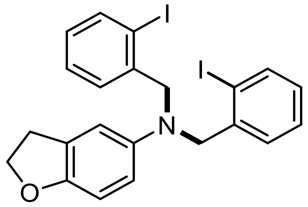

According to the general procedure in 0.2 mmol scale using 1.0 equiv. nitrobenzene with reaction time of 24 h at 65 °C; purified by flash chromatography (eluent: PE / EA = 20:1), 13.6 mg, 12% yield, colorless oil; R_f = 0.6 (PE / EA = 15:1). **^{1}H NMR** (400 MHz, Chloroform-*d*) δ 7.80 – 7.75 (m, 2H), 7.26 – 7.21 (m, 2H), 7.20 – 7.15 (m, 2H), 6.95 – 6.85 (m, 2H), 6.56 – 6.48 (m, 1H), 6.39 – 6.34 (m, 1H), 6.25 – 6.18 (m, 1H), 4.46 – 4.29 (m, 6H), 3.00 (t, *J* = 8.5 Hz, 2H). **^{13}C NMR** (101 MHz, $CDCl_3$) δ = 152.3, 142.6, 139.6, 139.4, 128.7, 128.4, 127.9, 127.8, 111.9, 109.7, 109.4, 97.7, 70.9, 61.2, 30.5. **IR (ATR)**: ν = 3056, 2920, 2850, 1494, 1435, 1209, 1044, 746, 429 cm^{-1}. **HRMS m/z (ESI)** calcd for $C_{22}H_{20}I_2NO$ (M + H)$^+$: 567.9629; found: 567.9615.

N-(3,4-dichlorobenzyl)-N-(1-(4-isobutylphenyl)ethyl)-4-methoxyaniline (**62**)

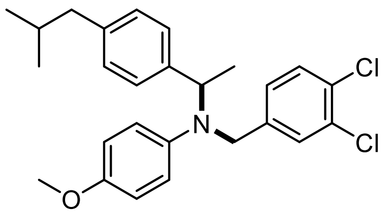

According to the general procedure in 0.2 mmol scale using 1.0 equiv. nitrobenzene with reaction time of 24 h at 65 °C; purified by flash chromatography (eluent: PE / EA = 30:1), 37.1 mg, 42% yield, colorless oil; R_f = 0.6 (PE / EA = 15:1). **^{1}H NMR** (500 MHz, Chloroform-*d*) δ 7.30 – 7.21 (m, 4H), 7.11 – 7.06 (m, 2H), 7.06 – 7.01 (m, 1H), 6.75 (s, 4H), 4.93 (q, *J*

= 6.8 Hz, 1H), 4.27 – 4.05 (m, 2H), 3.73 (s, 3H), 2.44 (d, *J* = 7.2 Hz, 2H), 1.92 – 1.79 (m, 1H), 1.47 (d, *J* = 6.8 Hz, 3H), 0.88 (d, *J* = 6.6 Hz, 6H). **^{13}C NMR** (126 MHz, $CDCl_3$) δ = 153.0, 142.8, 140.8, 140.5, 139.7, 132.1, 130.1, 129.1, 129.0, 127.0, 126.4, 125.5, 118.5, 114.4, 59.1, 55.5, 50.2, 45.0, 30.2, 22.3, 17.7. **IR (ATR)**: ν = 2987, 2885, 1508, 1467, 1241, 1182, 1030, 806 cm^{-1}. **HRMS m/z (ESI)** calcd for $C_{26}H_{30}Cl_2NO$ (M + H)$^+$: 442.1699; found: 442.1693.

N-(1-(4-isobutylphenyl)ethyl)-4-methoxyaniline (**62a**)

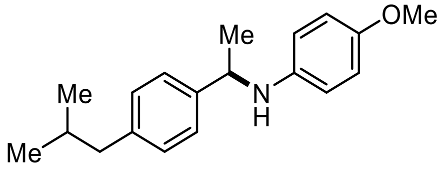

According to the general procedure in 0.2 mmol scale using 1.0 equiv. nitrobenzene with reaction time of 32 h at 65 ^{o}C; purified by flash chromatography (eluent: PE / EA = 30:1), 15.8 mg, 28% yield, colorless oil; R_f = 0.5 (PE / EA = 15:1). **^{1}H NMR** (400 MHz, Chloroform-*d*) δ 7.28 (d, *J* = 7.9 Hz, 2H), 7.11 (d, *J* = 7.8 Hz, 2H), 6.77 – 6.68 (m, 2H), 6.56 – 6.47 (m, 2H), 4.41 (q, *J* = 6.7 Hz, 1H), 3.72 (s, 3H), 2.47 (d, *J* = 7.2 Hz, 2H), 1.95 – 1.78 (m, 1H), 1.51 (d, *J* = 6.7 Hz, 3H), 0.92 (d, *J* = 6.6 Hz, 6H). **^{13}C NMR** (101 MHz, $CDCl_3$) δ = 151.9, 142.5, 141.5, 140.1, 129.2, 125.6, 114.7, 114.6, 55.7, 54.1, 45.0, 30.1, 24.8, 22.4. **IR (ATR)**: ν = 3380, 3051, 2953, 2923, 1510, 1234, 907, 748, 520 cm^{-1}. **HRMS m/z (ESI)** calcd for $C_{19}H_{26}NO$ (M + H)$^+$: 284.2009; found: 284.1993.

N,N-bis(3,4-dichlorobenzyl)-4-methoxyaniline (**62b**)

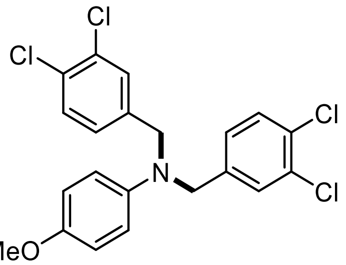

According to the general procedure in 0.2 mmol scale using 1.0 equiv. nitrobenzene with reaction time of 32 h at 65 ^{o}C; purified by flash chromatography (eluent: PE / EA = 30:1), 15.8 mg, 18% yield, colorless oil; R_f = 0.7 (PE / EA = 15:1). **^{1}H NMR** (400 MHz, Chloroform-*d*) δ 7.40 – 7.35 (m, 2H), 7.34 – 7.31 (m, 2H), 7.10 – 7.04 (m, 2H), 6.82 – 6.75 (m, 2H), 6.72 – 6.64 (m, 2H), 4.44 (s, 4H), 3.74 (s, 3H). **^{13}C NMR** (101 MHz, $CDCl_3$) δ = 152.8, 142.6, 138.9, 132.7, 131.0, 130.6, 129.0, 126.4, 115.7, 114.9, 55.6, 54.8. **IR (ATR)**: ν = 3050, 2994, 2931, 2832, 1511, 1242, 962, 718 cm^{-1}. **HRMS m/z (ESI)** calcd for $C_{21}H_{18}Cl_4NO$ (M + H)$^+$: 440.0137; found: 440.0125.

ethyl-2-ethoxy-4-(((1-(4-isobutylphenyl)ethyl)(p-tolyl)amino)methyl)benzoate (**63**)

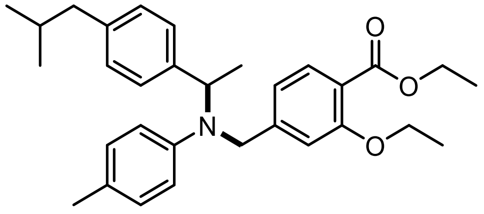

According to the general procedure in 0.2 mmol scale using 1.0 equiv. nitrobenzene with reaction time of 24 h at 65 °C; purified by flash chromatography (eluent: PE / EA = 15:1), 37.9 mg, 40% yield, colorless oil; R_f = 0.6 (PE / EA = 15:1). **^{1}H NMR** (500 MHz, Chloroform-*d*) δ 7.67 (d, *J* = 8.4 Hz, 1H), 7.24 (d, *J* = 8.0 Hz, 2H), 7.08 (d, *J* = 8.2 Hz, 2H), 6.97 (d, *J* = 8.4 Hz, 2H), 6.83 (d, *J* = 6.8 Hz, 2H), 6.68 (d, *J* = 8.6 Hz, 2H), 5.19 (q, *J* = 6.9 Hz, 1H), 4.37 – 4.32 (m, 2H), 4.35 – 4.28 (m, 2H), 3.99 (qd, *J* = 6.9, 3.6 Hz, 2H), 2.44 (d, *J* = 7.1 Hz, 2H), 2.23 (s, 3H), 1.88 – 1.79 (m, 1H), 1.55 (d, *J* = 6.9 Hz, 3H), 1.39 (t, *J* = 6.9 Hz, 3H), 1.36 (t, *J* = 7.1 Hz, 3H), 0.89 (d, *J* = 6.6 Hz, 6H). **^{13}C NMR** (126 MHz, $CDCl_3$) δ = 166.39, 158.63, 146.89, 146.80, 140.37, 139.73, 131.52, 129.55, 129.13, 126.83, 125.87, 118.67, 118.24, 114.81, 111.44, 64.36, 60.53, 57.20, 50.35, 44.96, 30.15, 22.34, 20.22, 17.84, 14.62, 14.27. **IR (ATR)**: ν = 2955, 2925, 1727, 1610, 1517, 1249, 1079, 803 cm^{-1}. **HRMS m/z (ESI)** calcd for $C_{31}H_{40}NO_3$ $(M + H)^+$: 474.3003; found: 473.3000.

N-(1-(4-isobutylphenyl)ethyl)-4-methylaniline (**63a**)

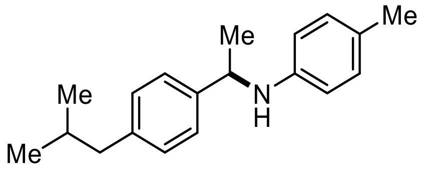

According to the general procedure in 0.2 mmol scale using 1.0 equiv. nitrobenzene with reaction time of 32 h at 65 °C; purified by flash chromatography (eluent: PE / EA = 15:1), 16.0 mg, 30% yield, colorless oil; R_f = 0.4 (PE / EA = 15:1). **^{1}H NMR** (400 MHz, Chloroform-*d*) δ 7.35 – 7.26 (m, 2H), 7.16 – 7.08 (m, 2H), 6.99 – 6.90 (m, 2H), 6.56 – 6.45 (m, 2H), 4.47 (q, *J* = 6.7 Hz, 1H), 2.47 (d, *J* = 7.2 Hz, 2H), 2.23 (s, 3H), 1.87 (dt, *J* = 13.5, 6.7 Hz, 1H), 1.54 (d, *J* = 6.7 Hz, 3H), 0.93 (d, *J* = 7.2 Hz, 6H). **^{13}C NMR** (101 MHz, $CDCl_3$) δ = 144.6, 142.2, 140.2, 129.6, 129.3, 126.7, 125.7, 113.8, 53.8, 45.1, 30.2, 24.6, 22.4, 22.4, 20.3. **IR (ATR)**: ν = 3380, 3016, 2954, 2922, 2867, 1618, 1518, 806, 510 cm^{-1}. **HRMS m/z (ESI)** calcd for $C_{19}H_{26}N$ $(M + H)^+$: 268.2060; found: 268.2047.

diethyl 4,4'-((p-tolylazanediyl)bis(methylene))bis(2-ethoxybenzoate) (**63b**)

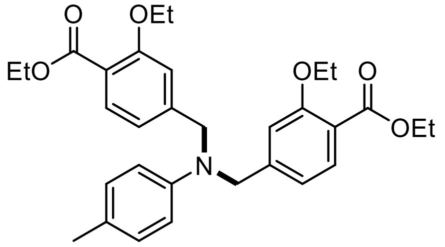

According to the general procedure in 0.2 mmol scale using 1.0 equiv. nitrobenzene with reaction time of 32 h at 65 °C; purified by flash chromatography (eluent: PE / EA = 15:1), 13.5 mg, 13% yield, colorless oil; R_f = 0.2 (PE / EA = 15:1). **^{1}H NMR** (500 MHz, Chloroform-*d*) δ 7.75 – 7.70 (m, 2H), 6.98 (d, *J* = 8.4 Hz, 2H), 6.87 – 6.83 (m, 2H), 6.81 (s, 2H), 6.66 – 6.60 (m, 2H), 4.56 (s, 4H), 4.34 (q, *J* = 7.1 Hz, 4H), 4.00 (q, *J* = 6.9 Hz, 4H), 2.23 (s, 3H), 1.47 – 1.30 (m, 12H). **^{13}C NMR** (101 MHz, $CDCl_3$) δ = 166.3, 158.8, 146.7, 144.8, 131.9, 129.7, 126.9, 119.5, 118.4, 113.3, 111.6, 64.5, 60.7, 54.9, 20.2, 14.6, 14.3. **IR (ATR)**: ν = 2979, 2928, 2348, 1723, 1110, 1040, 823, 670 cm^{-1}. **HRMS m/z (ESI)** calcd for $C_{31}H_{38}NO_6$ $(M + H)^+$: 520.2694; found: 520.2681.

3,4-dimethoxy-N-(p-tolyl)benzamide (**64**)

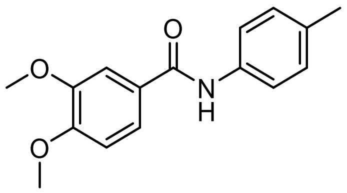

According to the general procedure in 0.2 mmol scale using 1.0 equiv. product **9** and Rose Bengal (5 mol%) with reaction time of 35 h under the O_2 atmosphere; purified by flash chromatography (eluent: PE / EA = 5:1), 48.8 mg, 90% yield, white solid; R_f = 0.2 (PE / EA = 5:1). **^{1}H NMR** (400 MHz, Chloroform-d) δ 8.20 (s, 1H), 7.55 – 7.49 (m, 2H), 7.45 (d, J = 2.1 Hz, 1H), 7.39 (dd, J = 8.3, 2.1 Hz, 1H), 7.11 (d, J = 8.2 Hz, 2H), 6.77 (d, J = 8.4 Hz, 1H), 3.86 (s, 3H), 3.82 (s, 3H), 2.30 (s, 3H). **^{13}C NMR** (101 MHz, CDCl3) δ = 165.3, 151.8, 148.9, 135.5, 133.8, 129.4, 127.5, 120.3, 119.6, 110.6, 110.2, 55.9, 55.8, 20.8.

N-benzyl-N-phenylheptanamide (**65**)

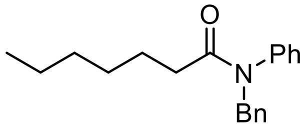

According to the general procedure in 0.2 mmol scale using 1.0 equiv. heptanoic acid (0.2 mmol), product **4** (0.6 mmol), PPh_3 (0.3 mmol) and I_2 (0.3 mmol) at room temperature under N_2 for 3h. Purified by flash chromatography (eluent: PE / EA = 5:1), 46.1 mg, 78% yield; R_f = 0.4 (PE / EA = 5:1). **^{1}H NMR** (400 MHz, Chloroform-*d*) δ 7.37 – 7.14 (m, 8H), 7.00 – 6.91 (m, 2H), 4.88 (s, 2H), 2.07 (t, *J* = 7.5 Hz, 2H), 1.60 (p, *J* = 7.2 Hz, 2H), 1.29 – 1.14 (m, 6H), 0.83 (t, *J* = 7.0 Hz, 3H). **^{13}C NMR** (101 MHz, $CDCl_3$) δ = 172.8, 142.4, 137.6, 129.3, 128.6, 128.3, 128.1, 127.6, 127.1, 52.8, 34.2, 31.4, 28.8, 25.3, 22.3, 13.9.

N-benzyl-N-phenyl-2-naphthamide (**66**)

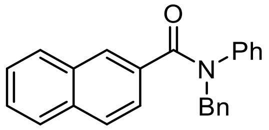

According to the general procedure in 0.2 mmol scale using 1.0 equiv. 2-naphthylacetonitrile, 1.2 equiv. product **9** and 5 mol% $CuCl_2$ with reaction time of 24 h under

the O_2 atmosphere; purified by flash chromatography (eluent: PE / EA = 5:1), 57.3 mg, 85% yield, white solid; R_f = 0.6 (PE / EA = 5:1). ^{1}**H NMR** (400 MHz, Chloroform-*d*) δ 8.13 (d, *J* = 8.3 Hz, 1H), 7.83 – 7.57 (m, 2H), 7.51 – 7.11 (m, 9H), 7.01 – 6.69 (m, 5H), 5.24 (s, 2H). ^{13}**C NMR** (101 MHz, $CDCl_3$) δ = 170.2, 142.3, 137.4, 134.1, 133.1, 130.2, 128.9, 128.6, 128.4, 128.1, 127.4, 127.3, 126.8, 126.7, 125.9, 125.5, 125.1, 124.2, 53.0.

2,4,6-triphenyl-1,3,5-triazine (**67**)

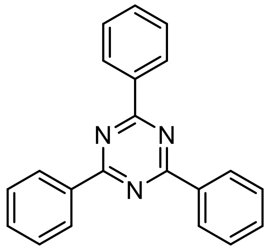

According to the general procedure in 0.2 mmol scale using 1.0 equiv. product **4**, 1.0 equiv. benzamidine hydrochloride, 2.0 equiv. K_3PO_4 and 5 mol% Cu $(OAc)_2$ with reaction time of 15 h under the O_2 atmosphere; purified by flash chromatography (eluent: PE / EA = 5:1), 40.9 mg, 66% yield, white solid; R_f = 0.2 (PE / EA = 5:1). ^{1}**H NMR** (400 MHz, Chloroform-*d*) δ 8.84 – 8.73 (m, 6H), 7.70 – 7.54 (m, 9H). ^{13}**C NMR** (101 MHz, $CDCl_3$) δ = 171.4, 135.6, 132.9, 129.2, 128.7.

N4,N4,N4',N4'-tetrabenzyl-[1,1'-biphenyl]-4,4'-diamine (**68**)

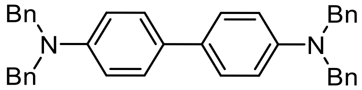

According to the general procedure in 0.2 mmol scale using 1.0 equiv. product **9** and 2.5 equiv. $FeCl_3 \cdot 6H_2O$ with reaction time of 3 h under the Air atmosphere at 85 °C; purified by flash chromatography (eluent: PE / EA = 80:1), 92.5 mg, 85% yield, white solid; R_f = 0.3 (PE / EA = 80:1). 92.5 mg, 85% yield.^{1}**H NMR** (400 MHz, Chloroform-*d*) δ 7.35 – 7.29 (m, 12H), 7.28 – 7.22 (m, 12H), 6.76 (d, *J* = 8.3 Hz, 4H), 4.65 (s, 8H). ^{13}**C NMR** (101 MHz, $CDCl_3$) δ = 147.7, 138.6, 128.6, 127.0, 126.9, 126.7, 112.8, 54.3. Same as the reference data.[1]

N-benzyl-N-phenylbenzamide (**69**)

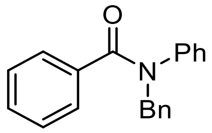

According to the general procedure in 0.2 mmol scale using 1.0 equiv. product **4**, 0.6 mmol TBHP (70% wt in water), 0.06 mmol TBAI and 5 mol% CuSO4·5H2O with reaction time of 24 h at 80 °C; purified by flash chromatography (eluent: PE / EA = 5:1), 33.3 mg, 58% yield; Rf = 0.4 (PE / EA = 5:1). ^{1}**H NMR** (500 MHz, Chloroform-d) δ 7.44 – 7.36 (m, 4H), 7.36 – 7.30 (m, 2H), 7.30 – 7.07 (m, 7H), 6.99 – 6.92 (m, 2H), 5.19 (s, 2H). ^{13}**C NMR** (126 MHz, CDCl3) δ = 170.3, 143.3, 137.4, 135.8, 129.4, 128.8, 128.6, 128.3, 128.2, 127.5, 127.5,

127.2, 126.5, 53.6.

4,4'-methylenebis(N,N-dibenzylaniline) (**70**)

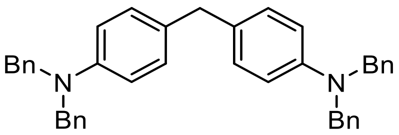

According to the general procedure in 0.2 mmol scale using 1.0 equiv. product **9**, 3.0 equiv. paraformaldehyde and 10 mol% PTSA with reaction time of 10 h under the Air; purified by flash chromatography (eluent: PE / EA = 30:1), 33.5 mg, 60% yield, white solid; R_f = 0.5 (PE / EA = 30:1). **^{1}H NMR** (400 MHz, Chloroform-*d*) δ 7.43 – 7.12 (m, 20H), 7.10 – 6.83 (m, 4H), 6.73 – 6.56 (m, 4H), 4.58 (s, 8H), 3.73 (s, 2H). **^{13}C NMR** (101 MHz, $CDCl_3$) δ = 147.4, 138.8, 130.0, 129.5, 128.5, 126.8, 126.7, 112.5, 54.3, 39.8.

2-(benzyl(phenyl)amino)-2-phenylacetonitrile (**71**)

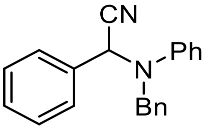

According to the general procedure in 0.2 mmol scale using 1.0 equiv. tertiary amine **4** , trimethylsilyl cyanide (0.4 mmol), iron (II) chloride (10 mol%) and tert-butyl hydroperoxide (0.5 mmol, 5.5M solution in decane) in MeOH (1.0 ml) at room temperature for 50 h; purified by flash chromatography (eluent: PE / EA = 50:1), 25.0 mg, yield 42%; Rf = 0.8 (PE / EA = 50:1). **^{1}H NMR** (400 MHz, Chloroform-d) δ 7.60 – 7.51 (m, 2H), 7.42 – 7.32 (m, 3H), 7.32 – 7.18 (m, 7H), 7.06 – 7.00 (m, 2H), 6.99 – 6.92 (m, 1H), 5.72 (s, 1H), 4.52 – 4.30 (m, 2H). **^{13}C NMR** (101 MHz, CDCl3) δ = 147.3, 137.4, 133.3, 129.2, 129.0, 128.9, 128.5, 127.6, 127.5, 127.3, 122.3, 119.8, 116.6, 58.5, 53.8.

4-((1H-benzo[d][1,2,3]triazol-1-yl)methyl)-N,N-dibenzylaniline (**72**)

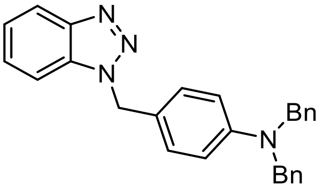

According to the general procedure in 0.2 mmol scale using 1.0 equiv. 1H-benzotriazole, 3.0 equiv. paraformaldehyde, 1.2 equiv. product **9** and 10 mol% PTSA with reaction time of 5 h under the Air; purified by flash chromatography (eluent: PE / EA = 10:1), 64.7 mg, 80% yield, white solid; R_f = 0.5 (PE / EA = 10:1). **^{1}H NMR** (500 MHz, Chloroform-*d*) δ 7.92 – 7.80 (m, 1H), 7.56 – 7.16 (m, 16H), 6.74 – 6.66 (m, 2H), 5.73 (s, 2H), 4.62 (s, 4H). **^{13}C NMR** (126 MHz, $CDCl_3$) δ = 149.2, 144.5, 138.1, 129.8, 128.8, 128.7, 127.0, 126.6, 126.5, 126.1, 122.6, 118.1, 112.5, 60.1, 54.2.

1-(4-(dibenzylamino)phenyl)-N,N-dimethylnaphthalen-2-amine (**73**)

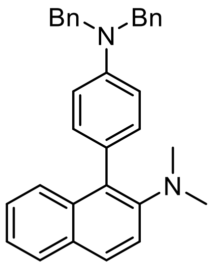

According to the general procedure in 0.2 mmol scale using 1.0 equiv. N,N-dimethylnaphthalen-2-amine, tertiary amine **4** (0.3 mmol), and HFIP (6 ml). Purified by flash chromatography (eluent: PE / EA = 30:1), 72.5 mg, yield 82%; R_f = 0.5 (PE / EA = 30:1). **^{1}H NMR** (400 MHz, Chloroform-d) δ 7.83 – 7.75 (m, 2H), 7.74 – 7.65 (m, 1H), 7.48 – 7.27 (m, 13H), 7.25 – 7.18 (m, 2H), 6.99 – 6.85 (m, 2H), 4.75 (s, 4H), 2.65 (s, 6H). **^{13}C NMR** (126 MHz, $CDCl_3$) δ = 148.0, 138.8, 134.1, 131.8, 128.6, 127.7, 127.6, 126.8, 125.6, 123.4, 119.4, 112.5, 54.2, 43.8.

6-(p-tolyl)-6,7-dihydro-5H-dibenzo[c,e]azepine (**74**)

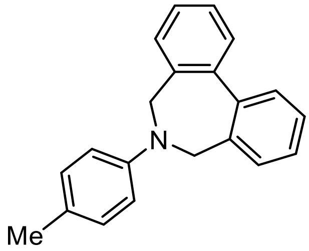

According to the general procedure in 0.2 mmol scale using 1.0 equiv. tertiary amine **34**, Pd $(OAc)_2$ (10 mol%), dppf (the indicated loading) and KOAc (1.0 mmol) in DMF (1 ml) at 100°C for 24 h; purified by flash chromatography (eluent: PE / EA = 50:1), 42.8 mg, yield 75%, white solid; R_f = 0.7 (PE / EA = 50:1). **^{1}H NMR** (500 MHz, Chloroform-*d*) δ 7.62 – 7.56 (m, 2H), 7.51 – 7.44 (m, 2H), 7.41 – 7.35 (m, 4H), 7.18 – 7.11 (m, 2H), 7.00 – 6.94 (m, 2H), 4.14 (s, 4H), 2.33 (s, 3H). **^{13}C NMR** (126 MHz, $CDCl_3$) δ = 147.4, 140.7, 134.8, 129.7, 129.6, 129.6, 128.1, 128.0, 127.6, 115.4. 52.5, 20.4.

2-(benzyl(phenyl)amino)acetonitrile (**75**)

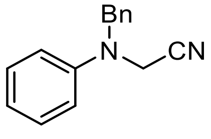

According to the general procedure in 0.2 mmol scale using 1.0 equiv. tertiary amine **4** , trimethylsilyl cyanide (0.4 mmol), iron (II) chloride (10 mol%) and tert-butyl hydroperoxide (0.5 mmol, 5.5M solution in decane) in MeOH (1.0 ml) at room temperature for 50 h; purified by flash chromatography (eluent: PE / EA = 50:1), 23.1 mg, yield 52%; R_f = 0.7 (PE / EA = 50:1). **^{1}H NMR** (400 MHz, Chloroform-*d*) δ 7.45 – 7.30 (m, 7H), 7.04 – 6.94 (m, 3H), 4.53 (s, 2H), 4.08 (s, 2H). **^{13}C NMR** (101 MHz, $CDCl_3$) δ = 147.9, 136.8, 129.5, 128.9, 127.8, 127.6, 120.6, 115.7, 115.6, 55.7, 39.5.

N-benzylaniline (**78**)

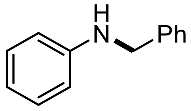

1H NMR (500 MHz, Chloroform-d) δ 7.44 – 7.34 (m, 4H), 7.35 – 7.28 (m, 1H), 7.26 – 7.18 (m, 2H), 6.82 – 6.73 (m, 1H), 6.72 – 6.65 (m, 2H), 4.36 (s, 2H), 4.21 (b, 1H).

N,N-diallyl-4-methoxyaniline (**94**)

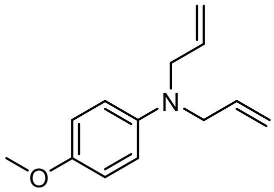

According to the general procedure in 0.2 mmol scale using 1.0 equiv. nitrobenzene with reaction time of 24 h at 65 °C; purified by flash chromatography (eluent: PE / EA = 15:1), 14.2 mg, 35% yield, colorless oil; R_f = 0.7 (PE / EA = 15:1). **^{1}H NMR** (400 MHz, Chloroform-*d*) δ 6.77 – 6.70 (m, 2H), 6.67 – 6.60 (m, 2H), 5.87 – 5.72 (m, 2H), 5.16 – 5.04 (m, 4H), 3.81 – 3.75 (m, 4H), 3.67 (s, 3H). **^{13}C NMR** (101 MHz, $CDCl_3$) δ = 151.6, 143.4, 134.5, 116.2, 114.6, 114.3, 77.3, 77.0, 76.7, 55.8, 53.7. **IR (ATR)**: ν = 3076, 2976, 2923, 1600, 1505, 1212, 1098, 823 cm^{-1}. **HRMS m/z (ESI)** calcd for $C_{13}H_{18}NO$ $(M + H)^+$: 204.1383; found: 204.1379.

N,N-diallyl-2,3-dihydrobenzofuran-5-amine (**95**)

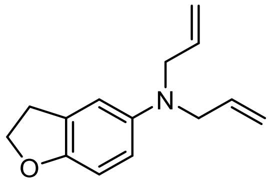

According to the general procedure in 0.2 mmol scale using 1.0 equiv. nitrobenzene with reaction time of 24 h at 65 °C; purified by flash chromatography (eluent: PE / EA = 20:1), 10.3 mg, 24% yield, colorless oil; R_f = 0.6 (PE / EA = 10:1). **^{1}H NMR** (400 MHz, Chloroform-*d*) δ 6.82 – 6.63 (m, 2H), 6.61 – 6.47 (m, 1H), 5.94 – 5.77 (m, 2H), 5.24 – 5.09 (m, 4H), 4.50 (t, *J* = 8.6 Hz, 2H), 3.83 (d, *J* = 5.2 Hz, 4H), 3.15 (t, *J* = 8.6 Hz, 2H). **^{13}C NMR** (101 MHz, $CDCl_3$) δ = 152.2, 134.6, 127.5, 116.2, 113.5, 111.5, 109.1, 96.0, 70.9, 54.1, 30.5. **IR (ATR)**: ν = 3065, 2920, 2930, 1640, 1505, 1200, 1100, 878, 808 cm^{-1}. **HRMS m/z (ESI)** calcd for $C_{14}H_{18}NO$ $(M + H)^+$: 216.1383; found: 216.1379.

7 NMR spectra of products

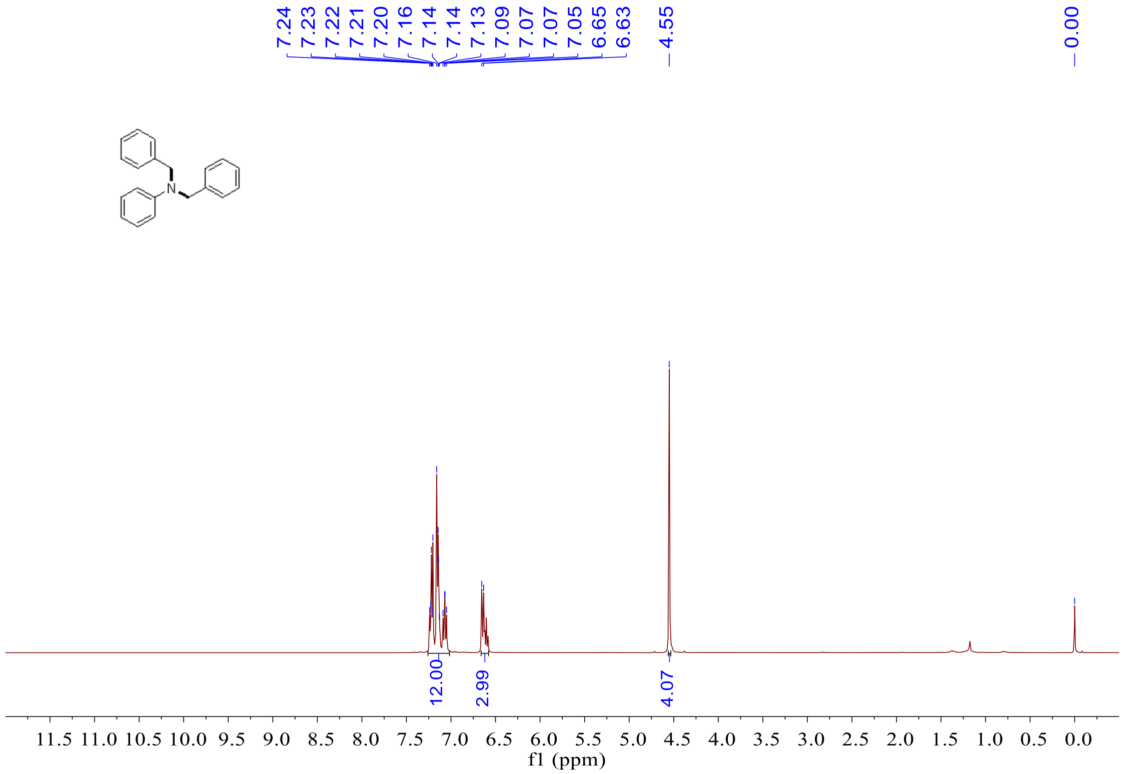

Supplementary Figure 13 ^{1}H NMR spectrum for compound **4**

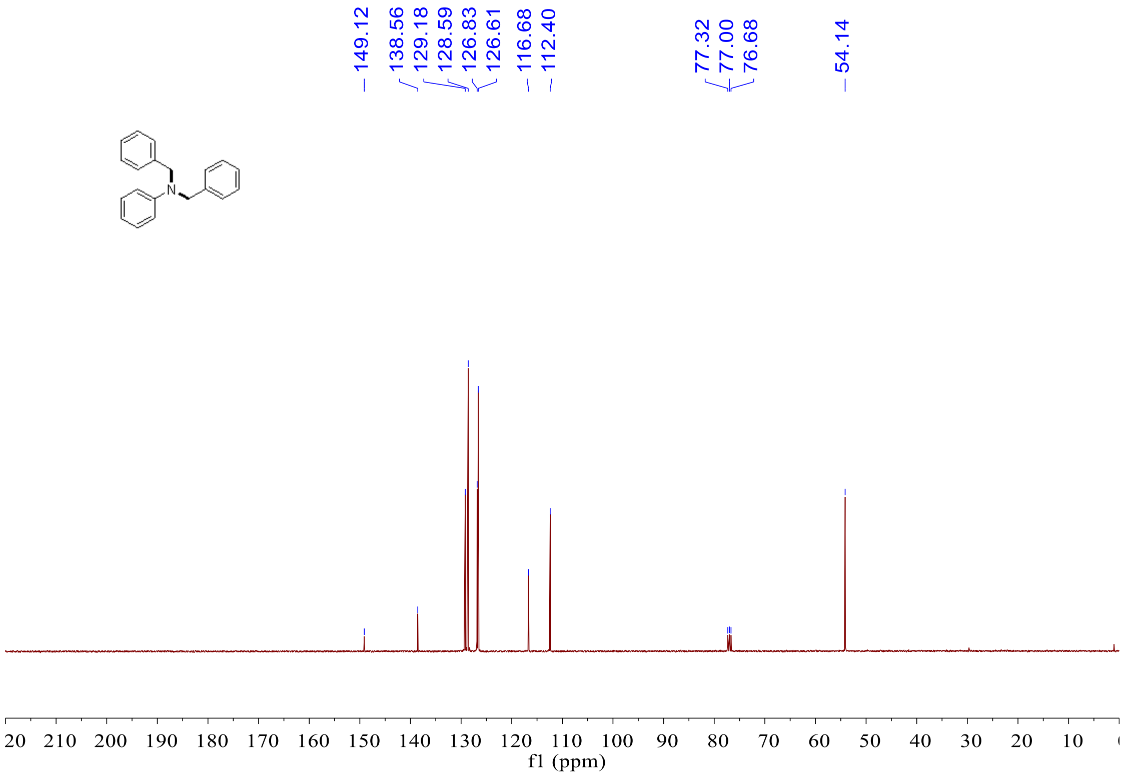

Supplementary Figure 14 ^{13}C NMR spectrum for compound **4**

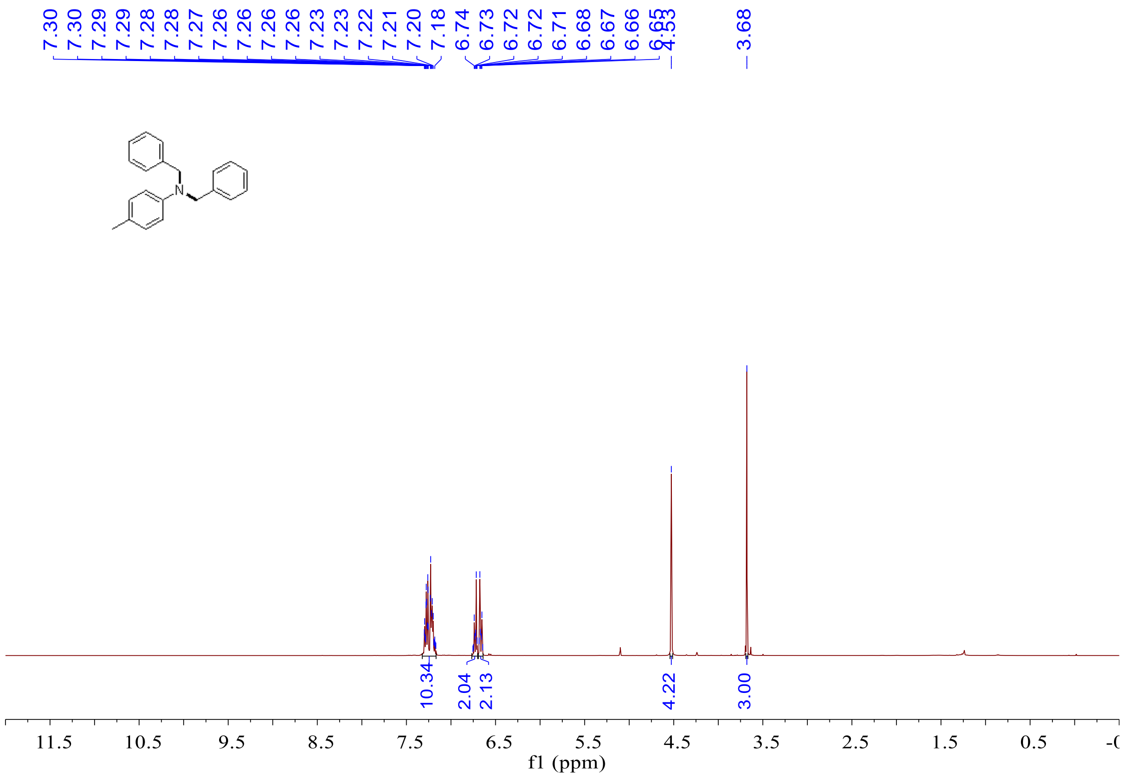

Supplementary Figure 15 ^{1}H NMR spectrum for compound **5**

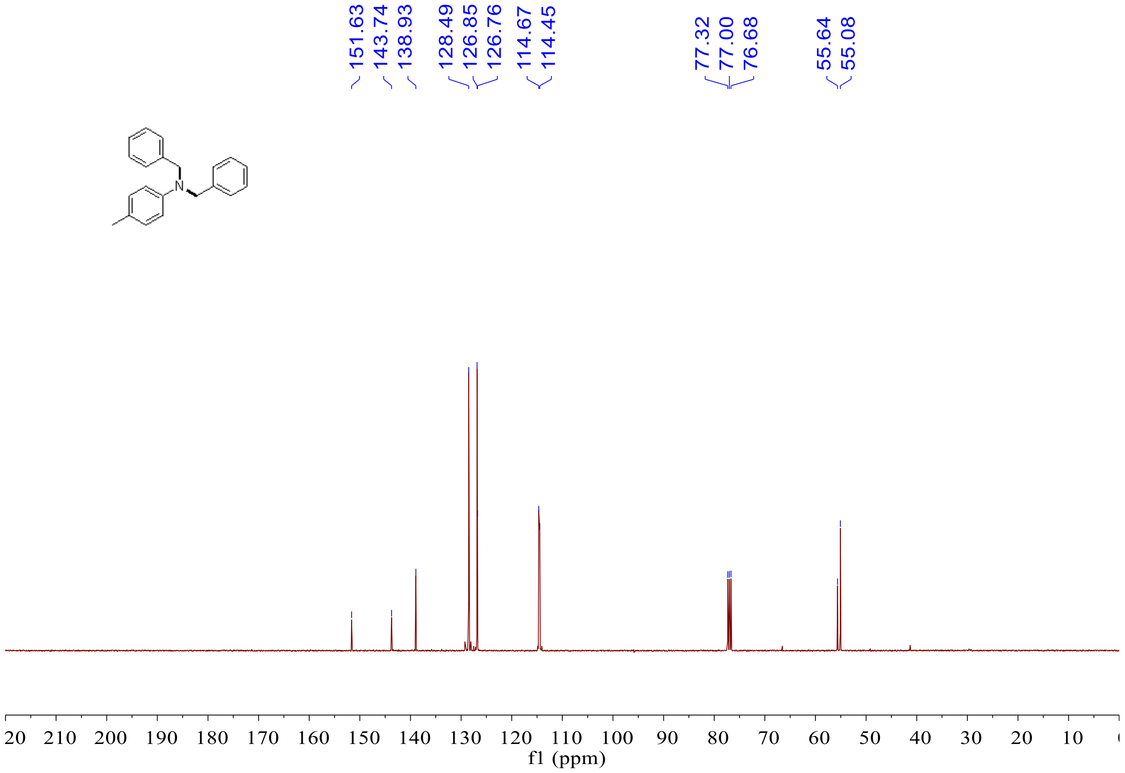

Supplementary Figure 16 ^{13}C NMR spectrum for compound **5**

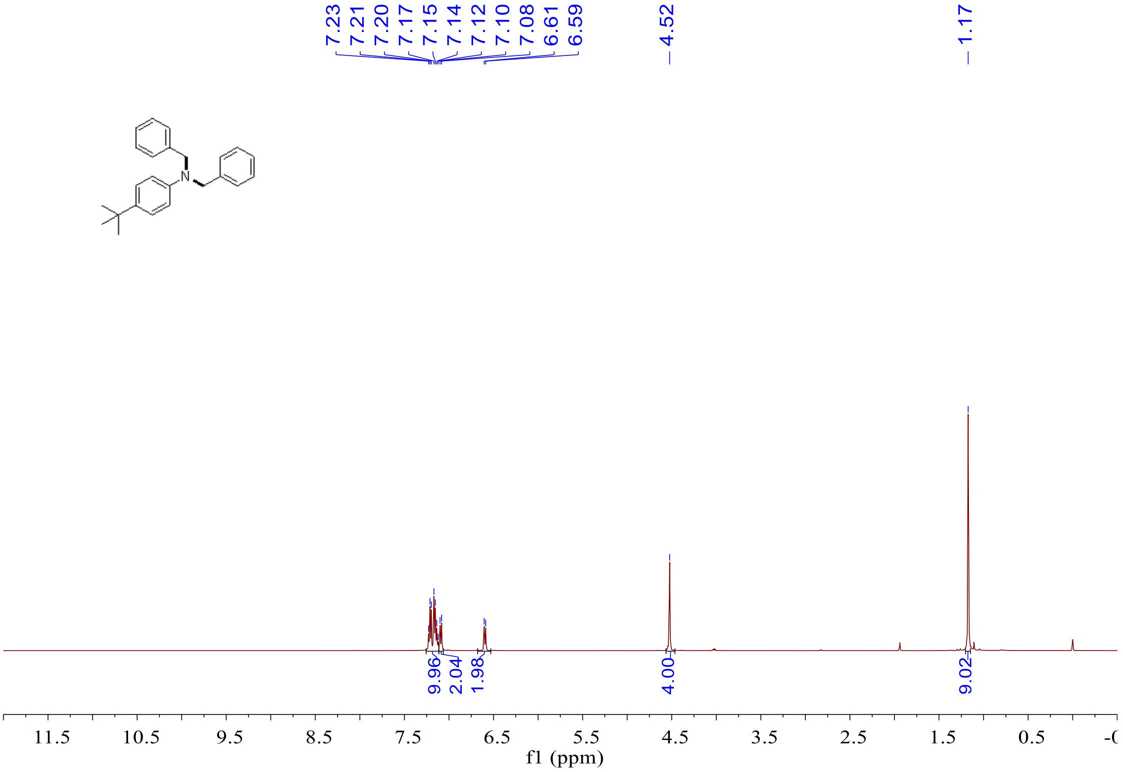

Supplementary Figure 17 ^{1}H NMR spectrum for compound **6**

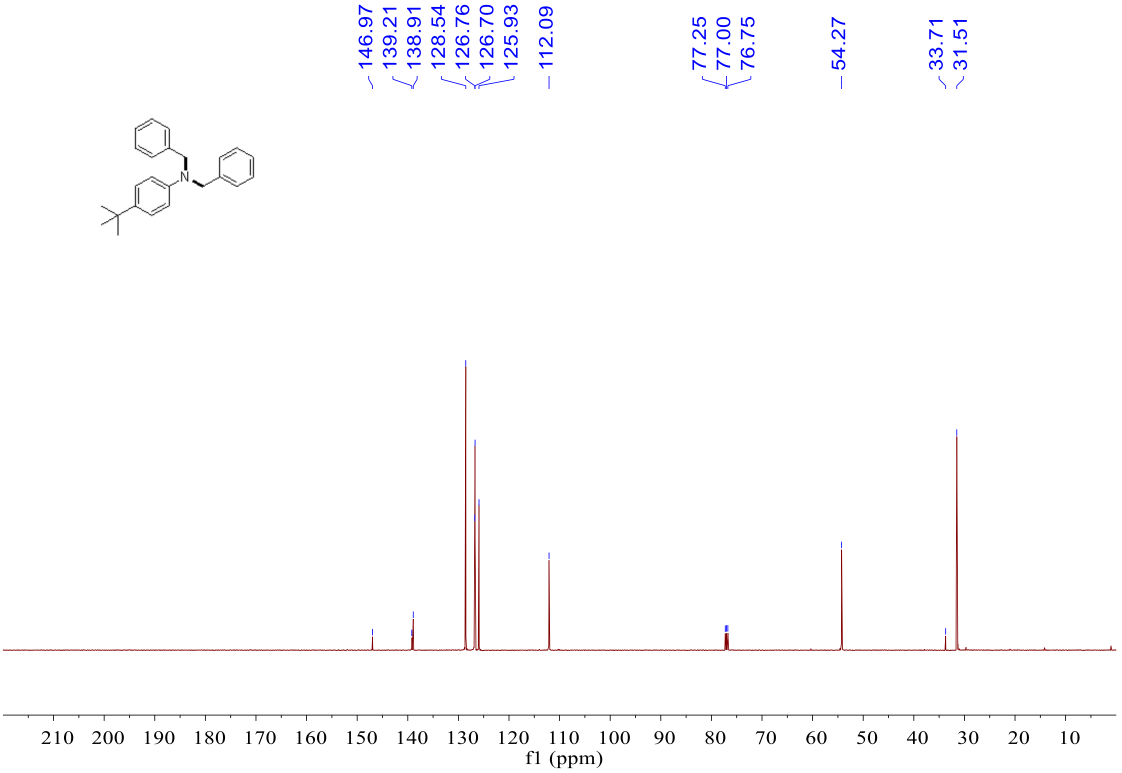

Supplementary Figure 18 ^{13}C NMR spectrum for compound **6**

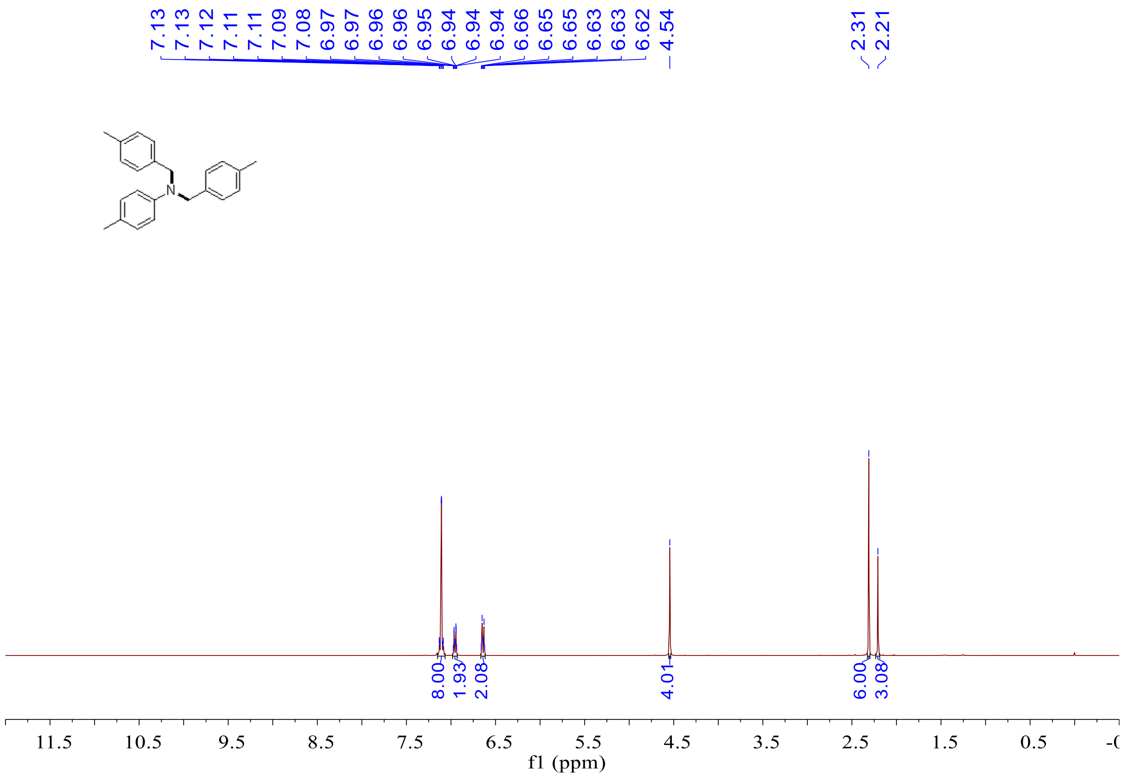

Supplementary Figure 19 ^{1}H NMR spectrum for compound **7**

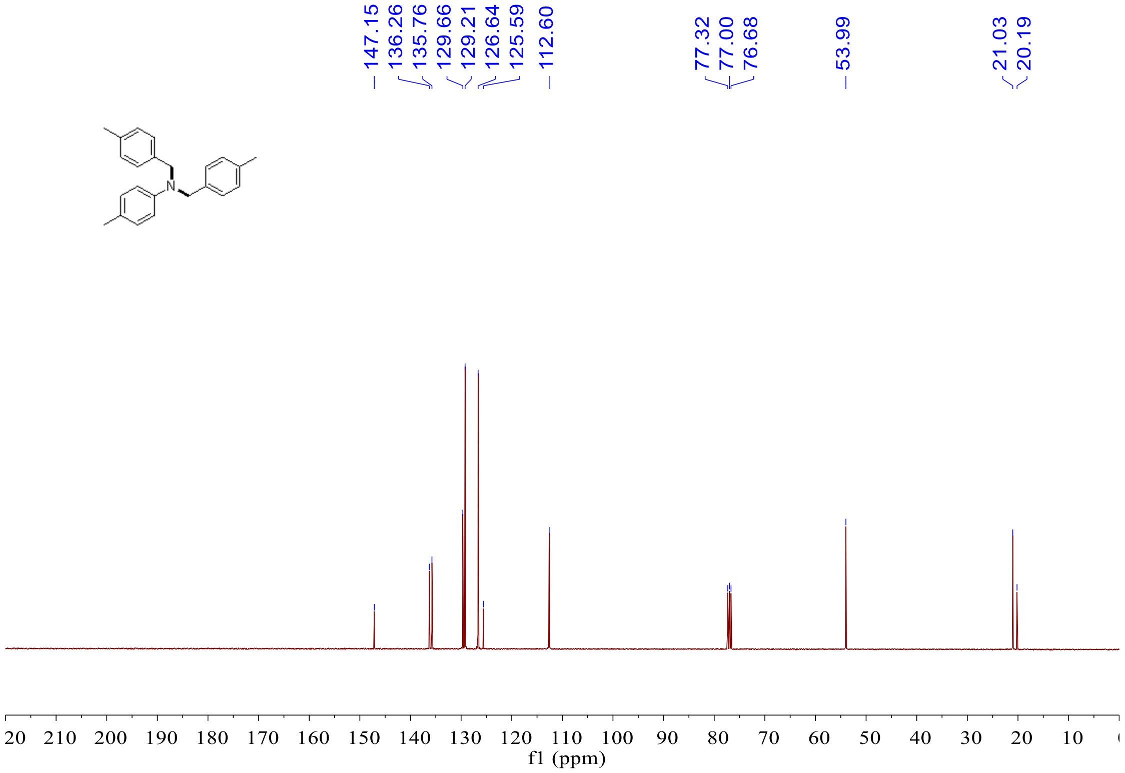

Supplementary Figure 20 ^{13}C NMR spectrum for compound **7**

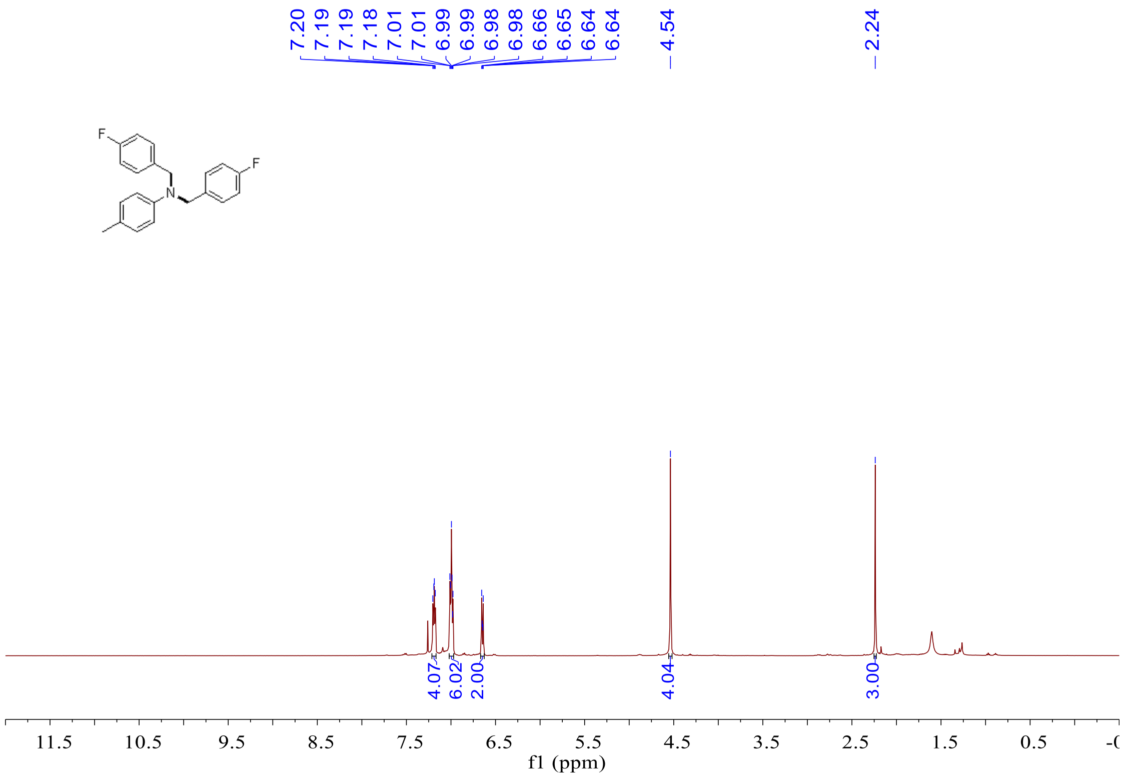

Supplementary Figure 21 ^{1}H NMR spectrum for compound **8**

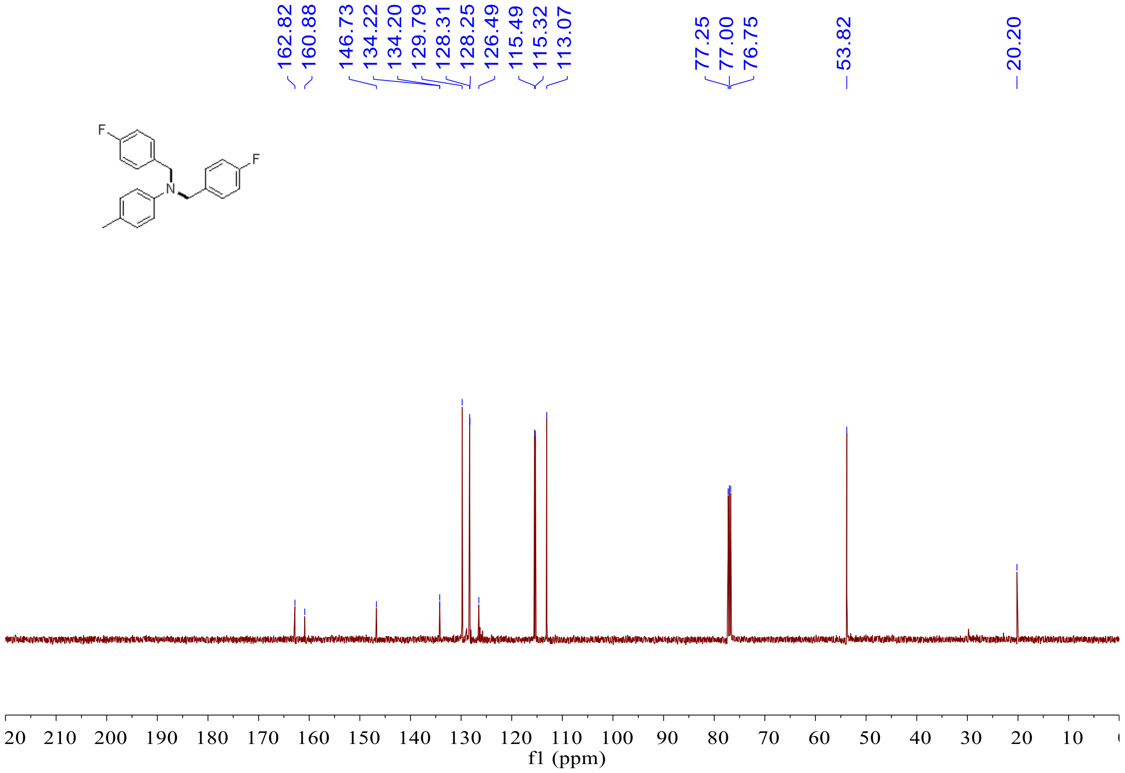

Supplementary Figure 22 ^{13}C NMR spectrum for compound **8**

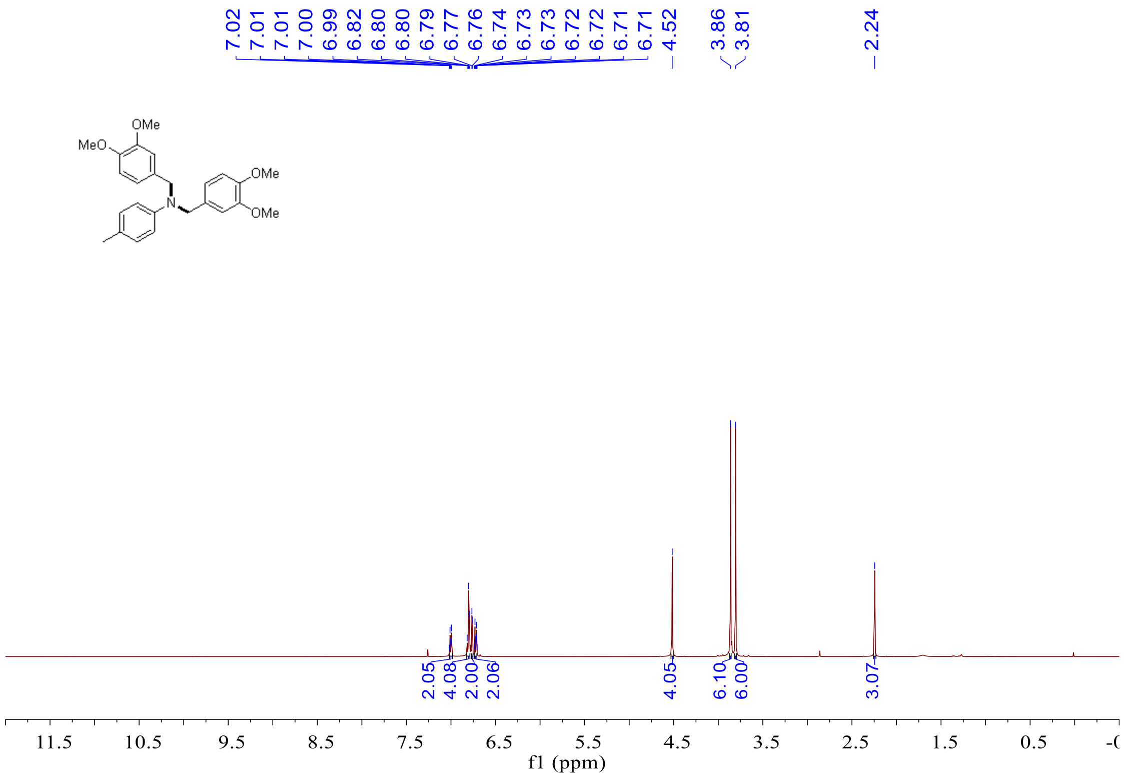

Supplementary Figure 23 ^{1}H NMR spectrum for compound **9**

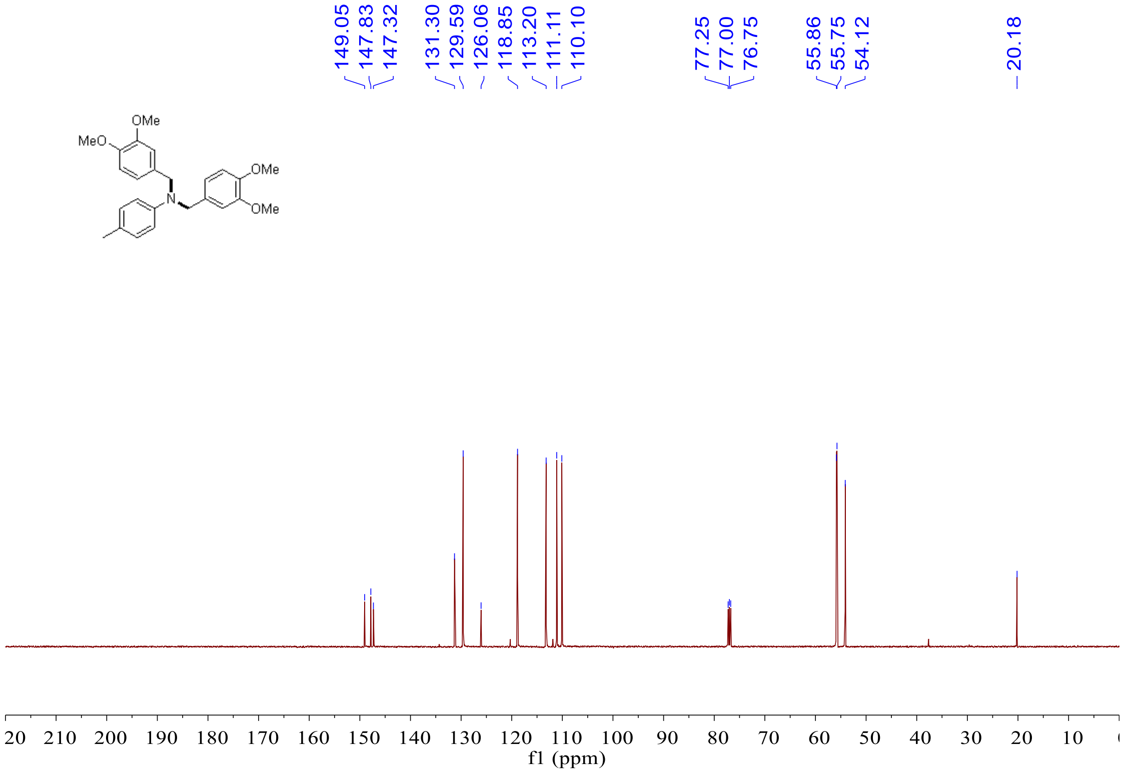

Supplementary Figure 24 ^{13}C NMR spectrum for compound **9**

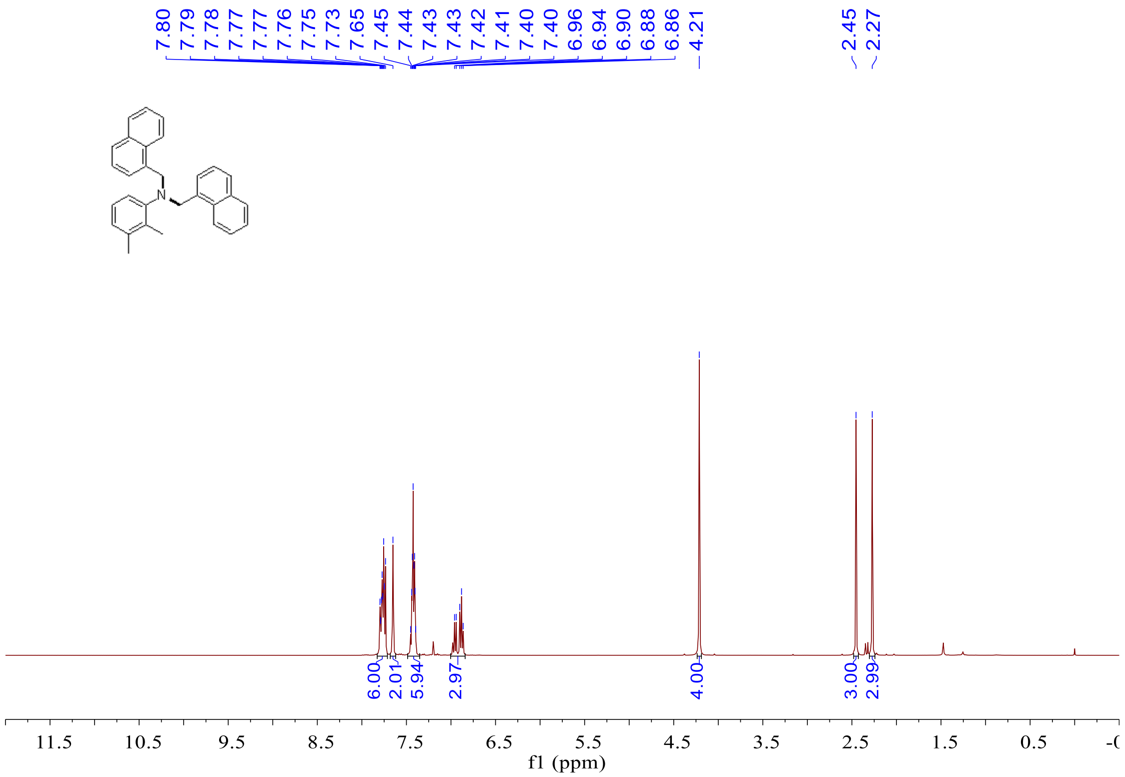

Supplementary Figure 25 ^{1}H NMR spectrum for compound **10**

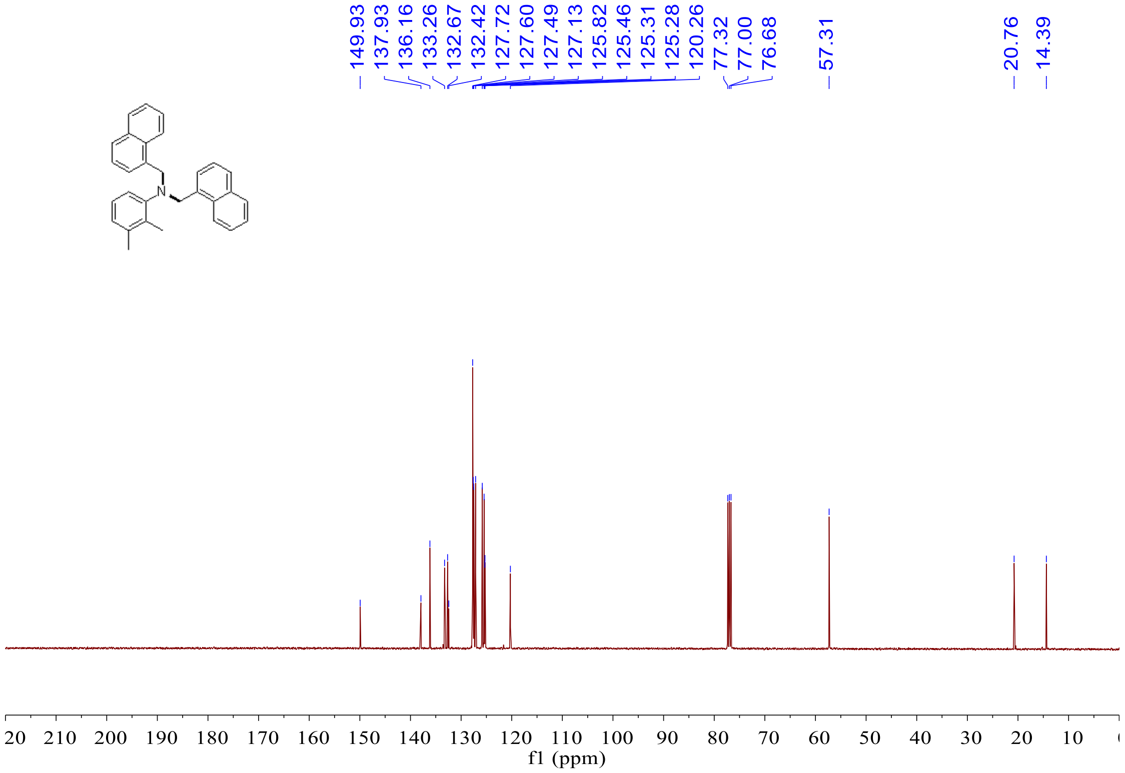

Supplementary Figure 26 ^{13}C NMR spectrum for compound **10**

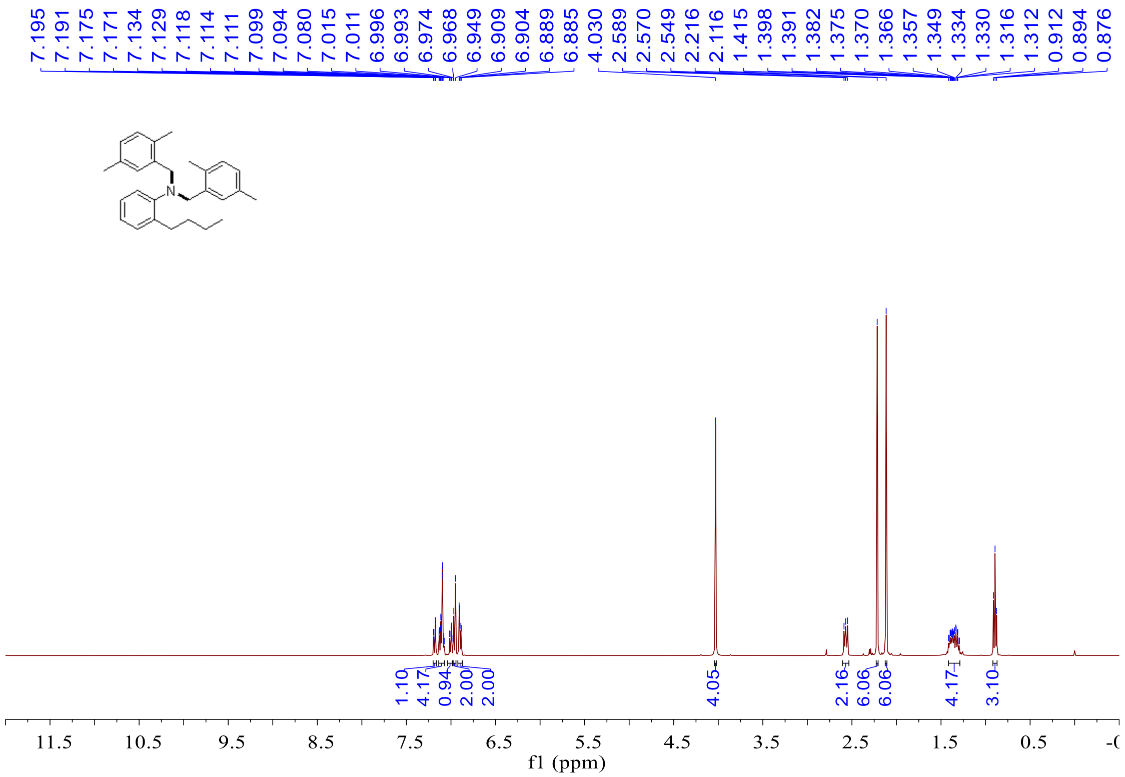

Supplementary Figure 27 ^{1}H NMR spectrum for compound **11**

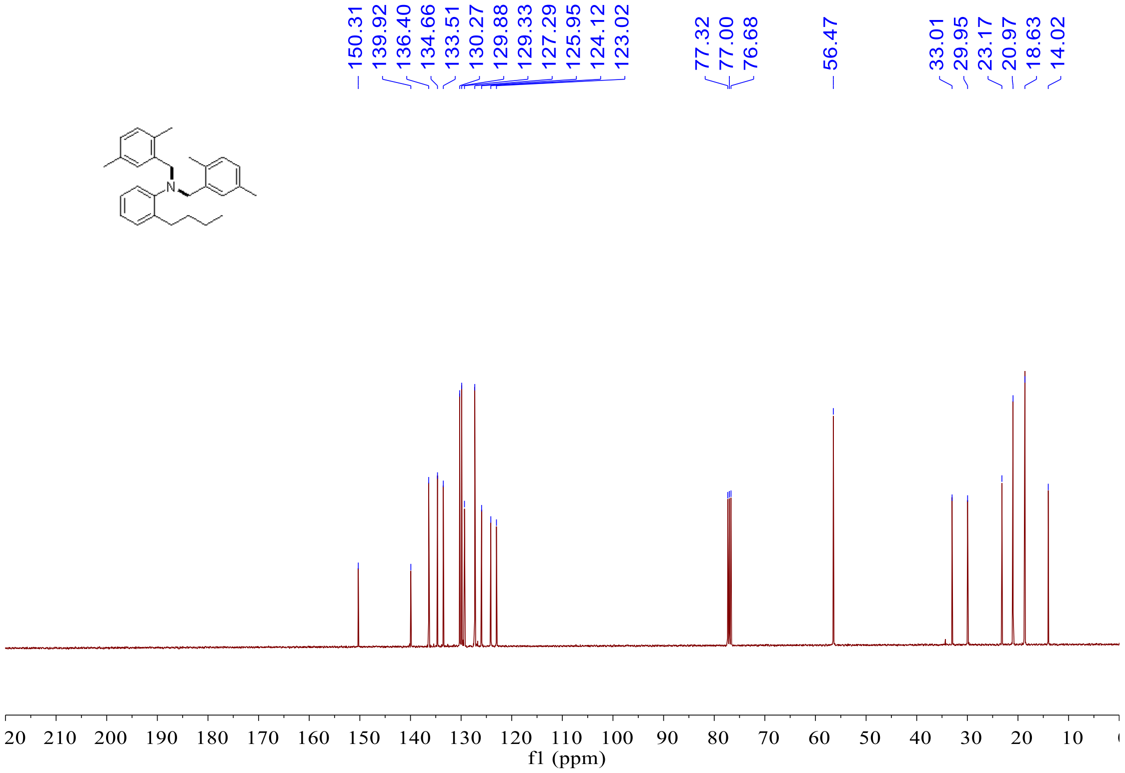

Supplementary Figure 28 ^{13}C NMR spectrum for compound **11**

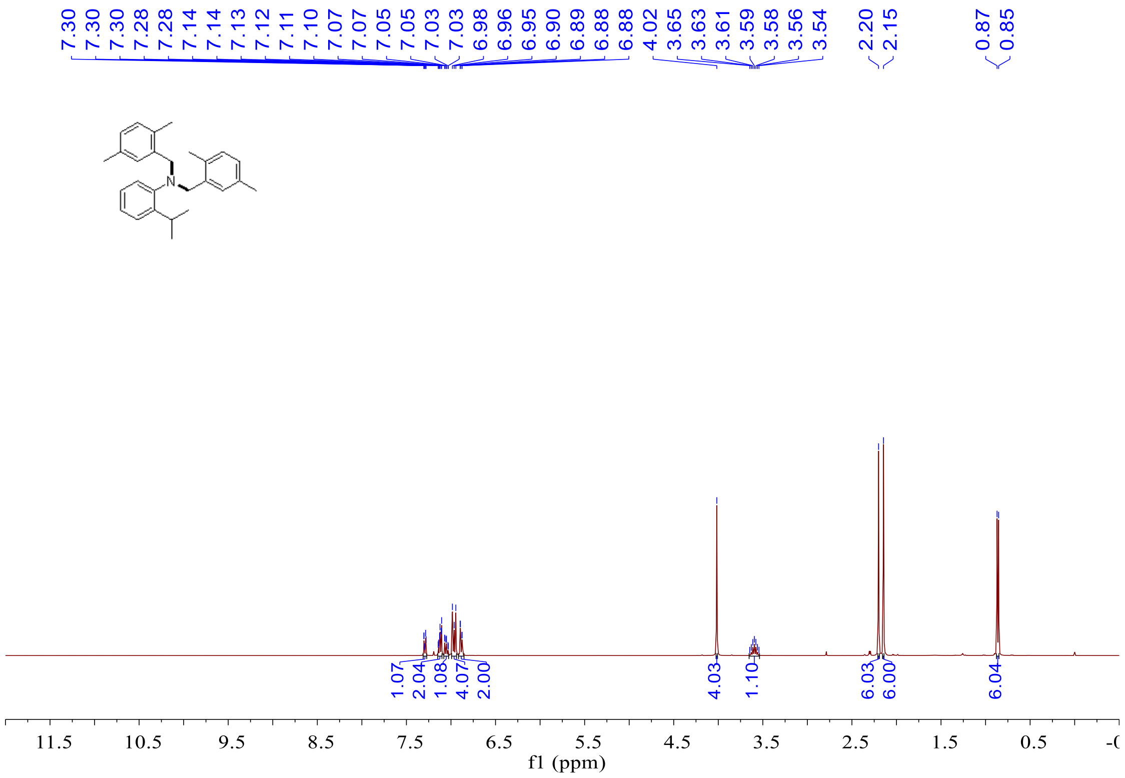

Supplementary Figure 29 ^{1}H NMR spectrum for compound **12**

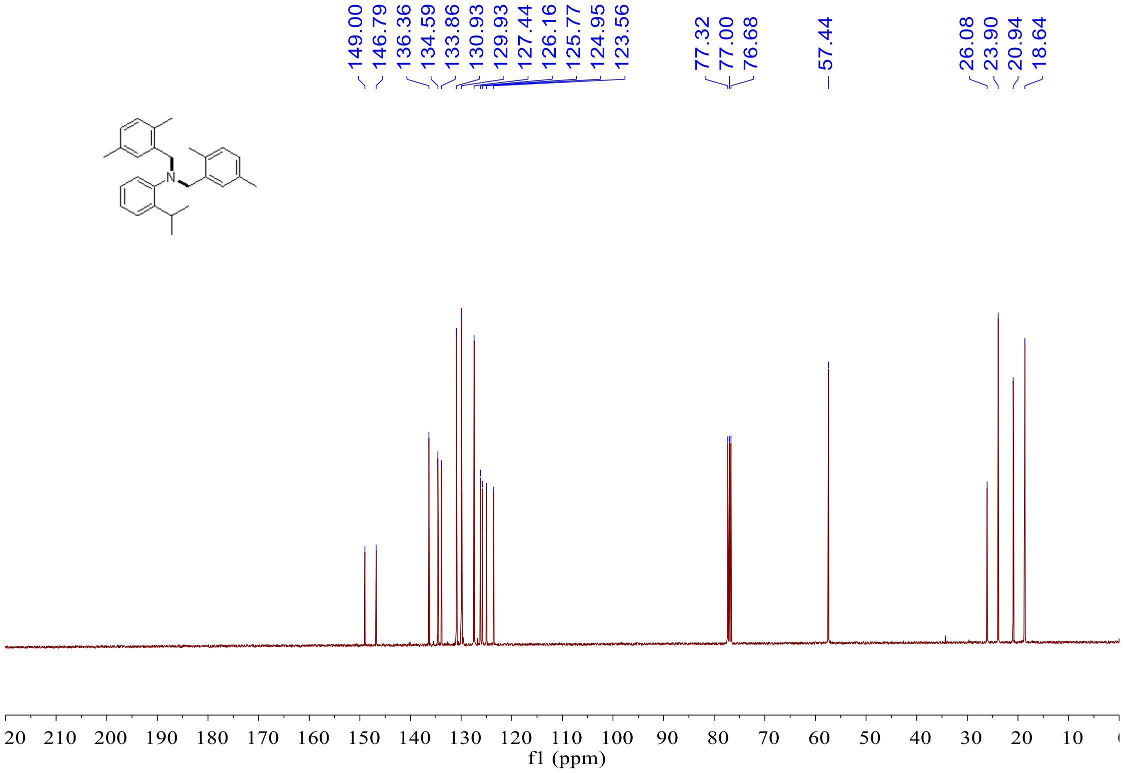

Supplementary Figure 30 ^{13}C NMR spectrum for compound **12**

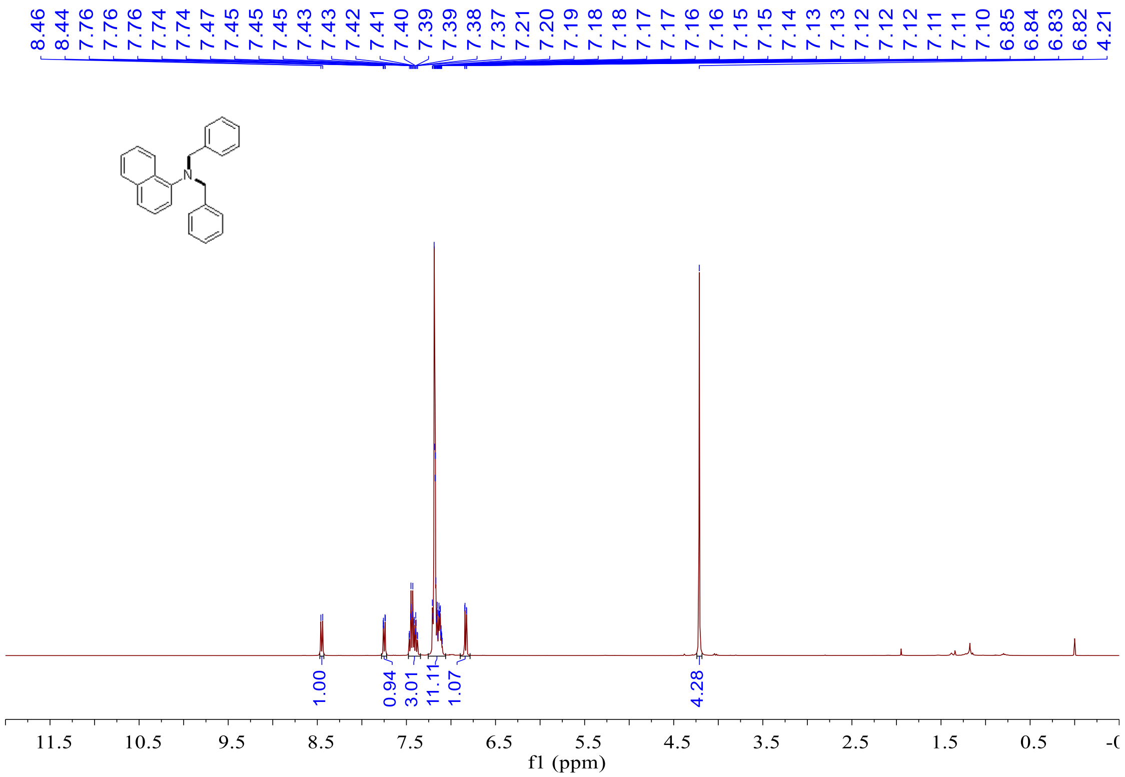

Supplementary Figure 31 ^{1}H NMR spectrum for compound **13**

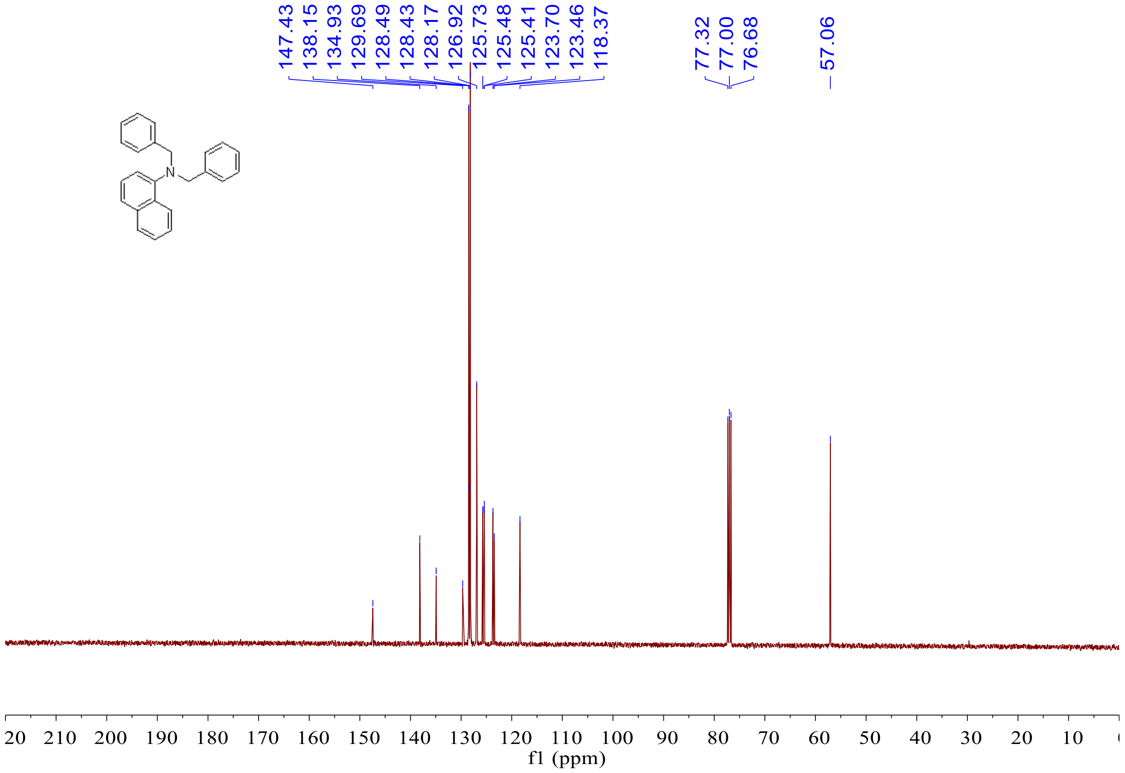

Supplementary Figure 32 ^{13}C NMR spectrum for compound **13**

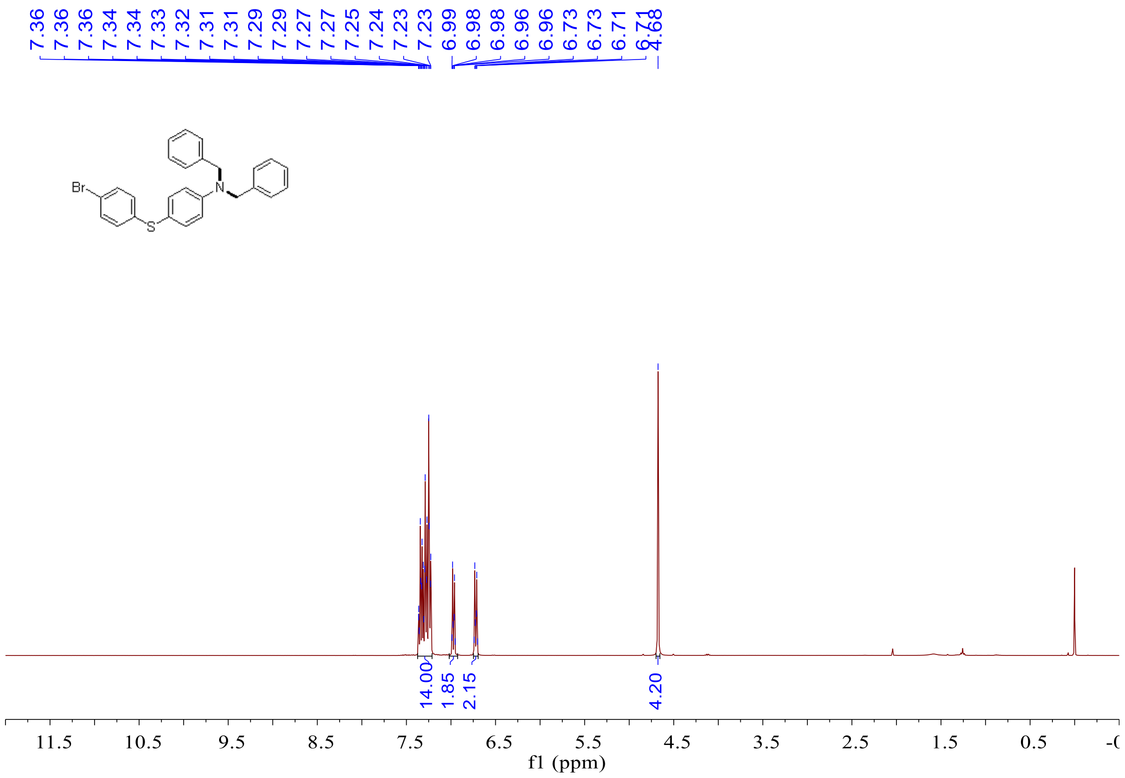

Supplementary Figure 33 ^{1}H NMR spectrum for compound **14**

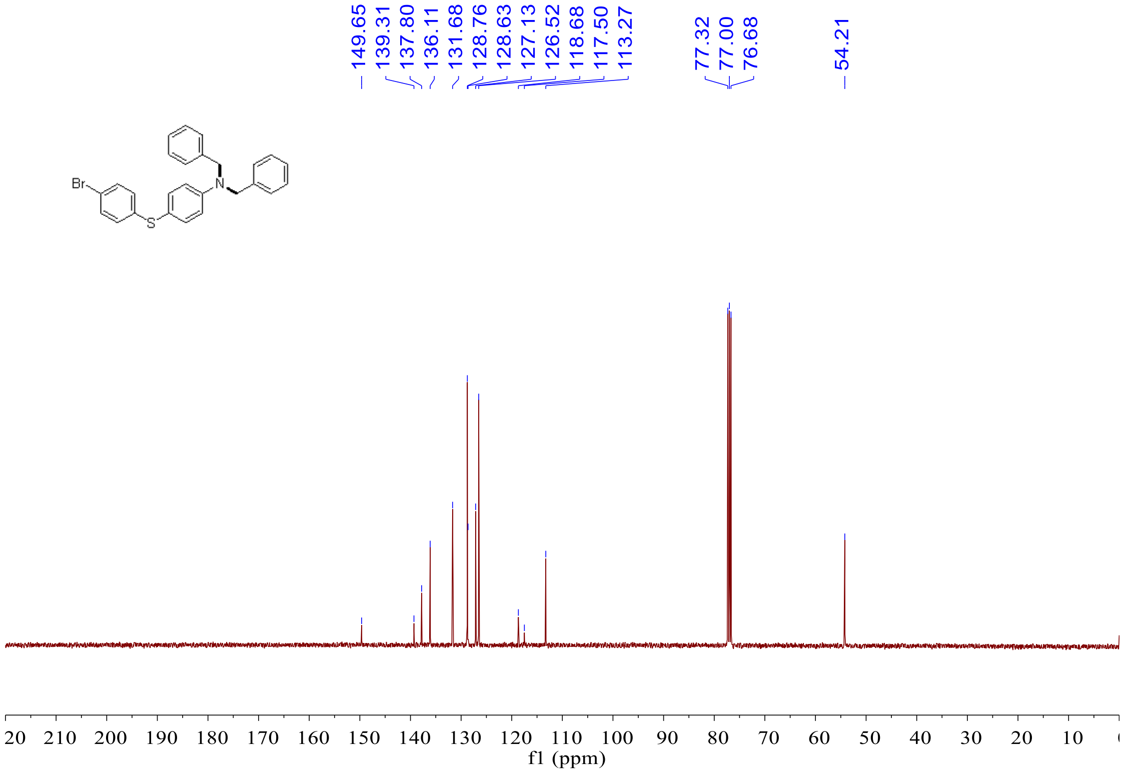

Supplementary Figure 34 ^{13}C NMR spectrum for compound **14**

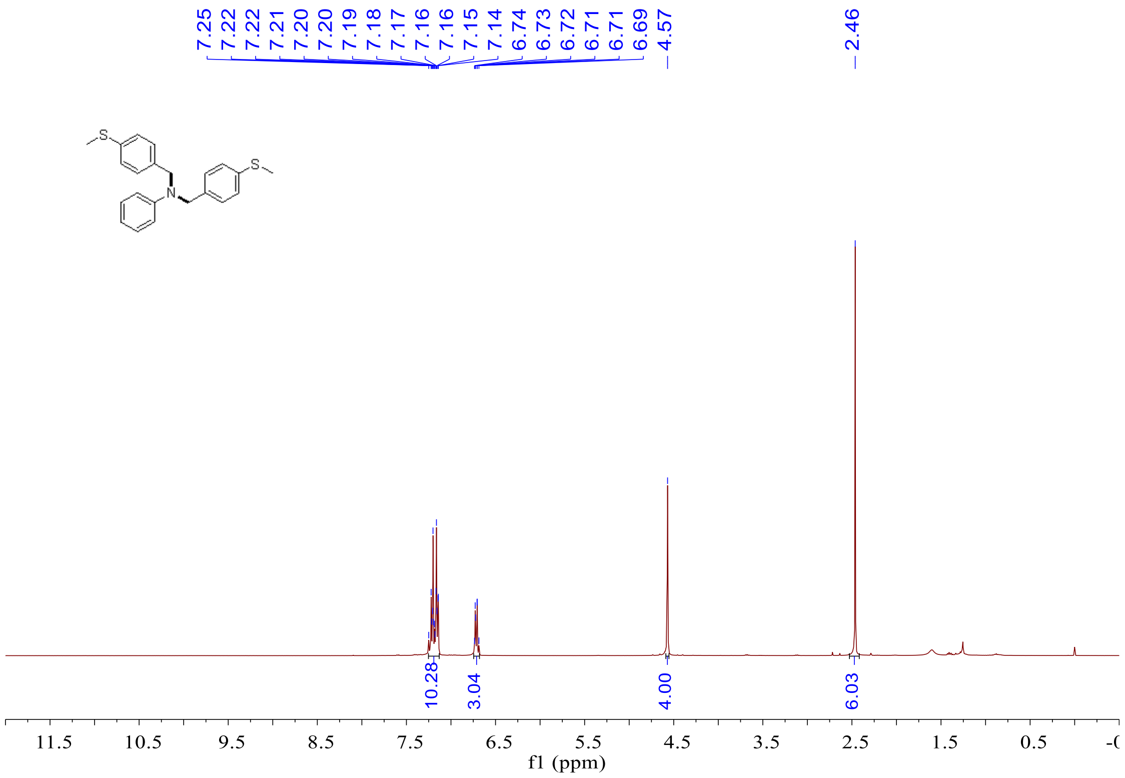

Supplementary Figure 35 ^{1}H NMR spectrum for compound **15**

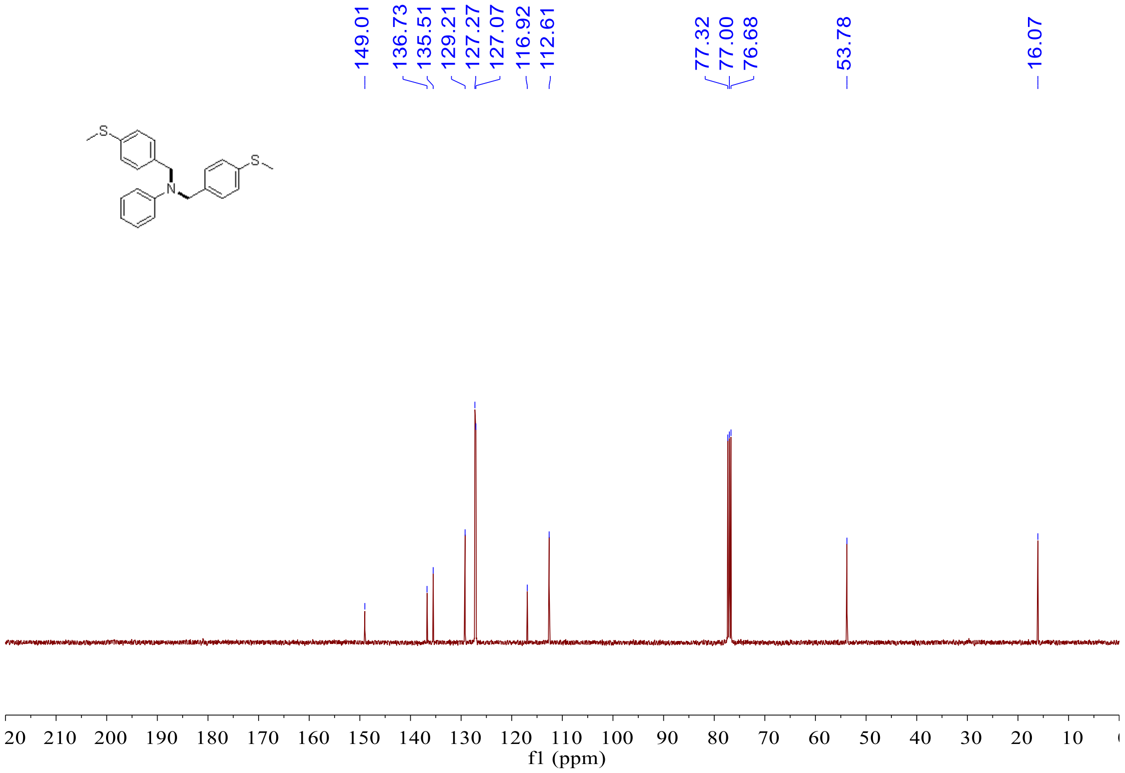

Supplementary Figure 36 ^{13}C NMR spectrum for compound **15**

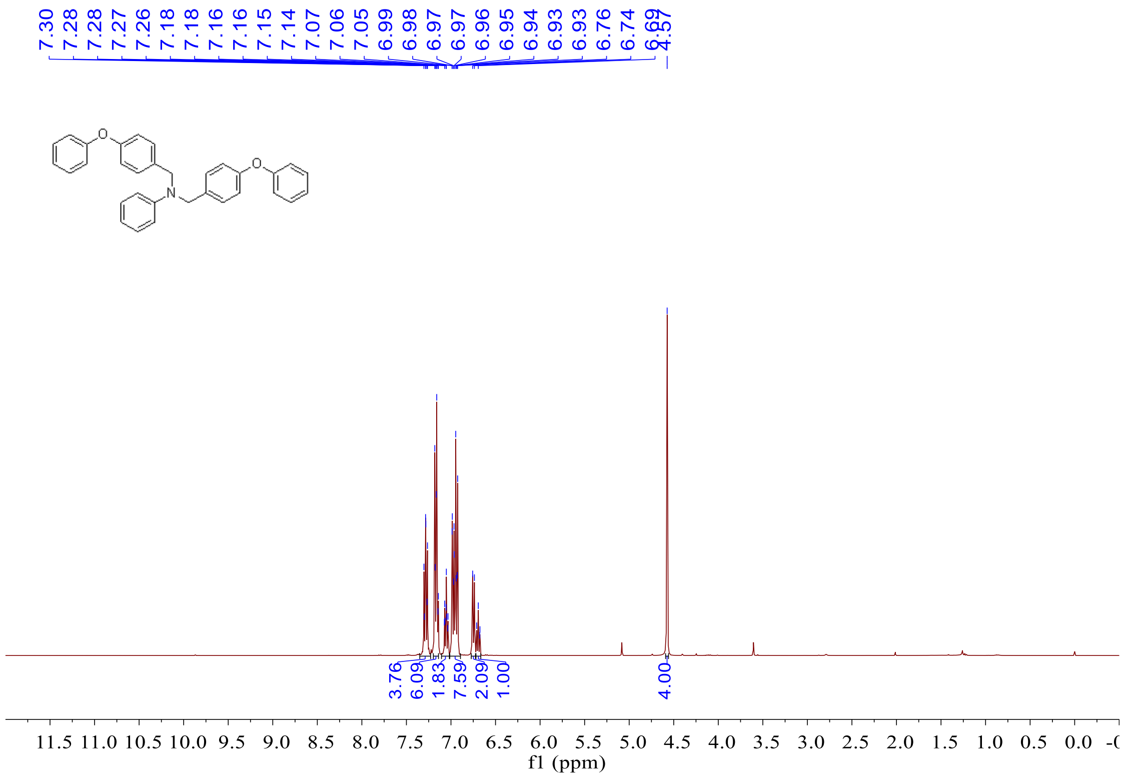

Supplementary Figure 37 ^1H NMR spectrum for compound **16**

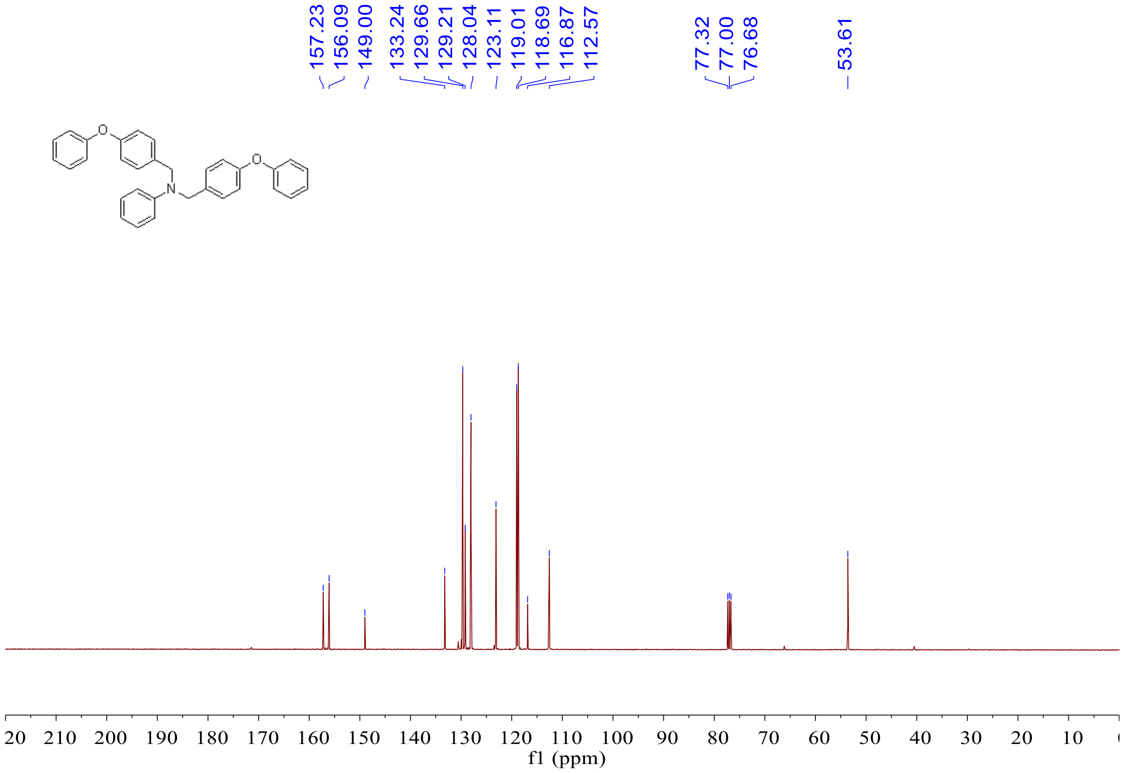

Supplementary Figure 38 ^{13}C NMR spectrum for compound 1**6**

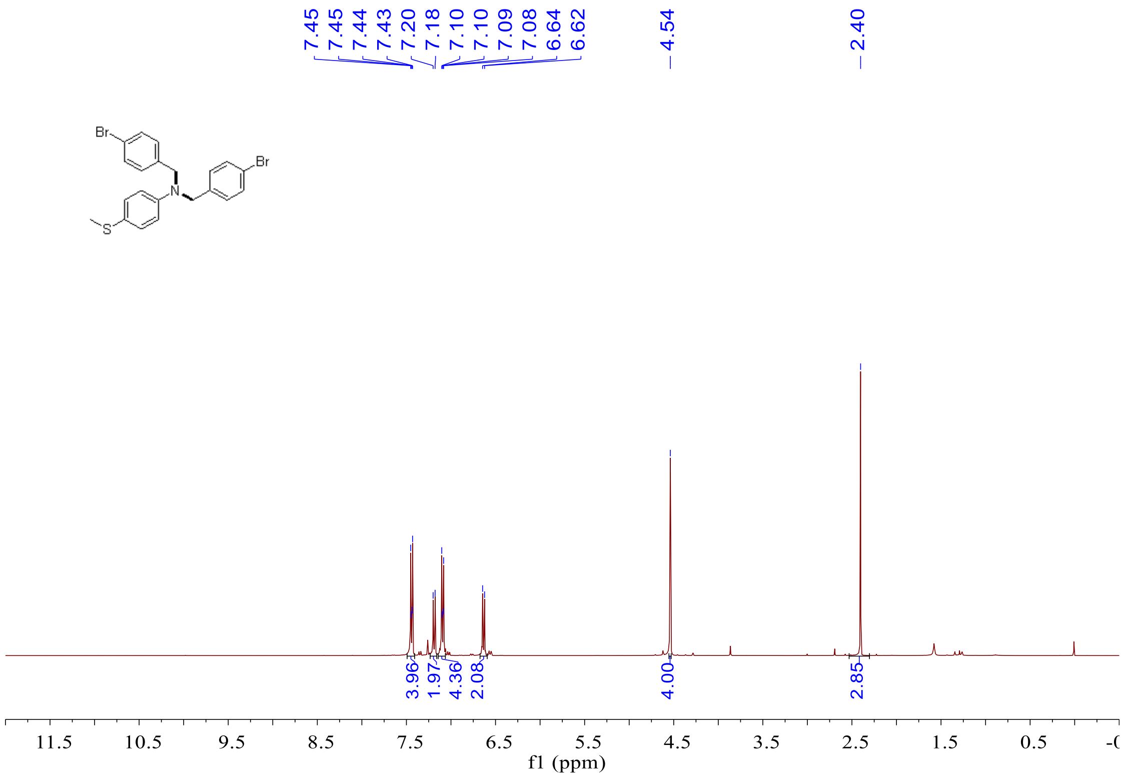

Supplementary Figure 39 ^{1}H NMR spectrum for compound **17**

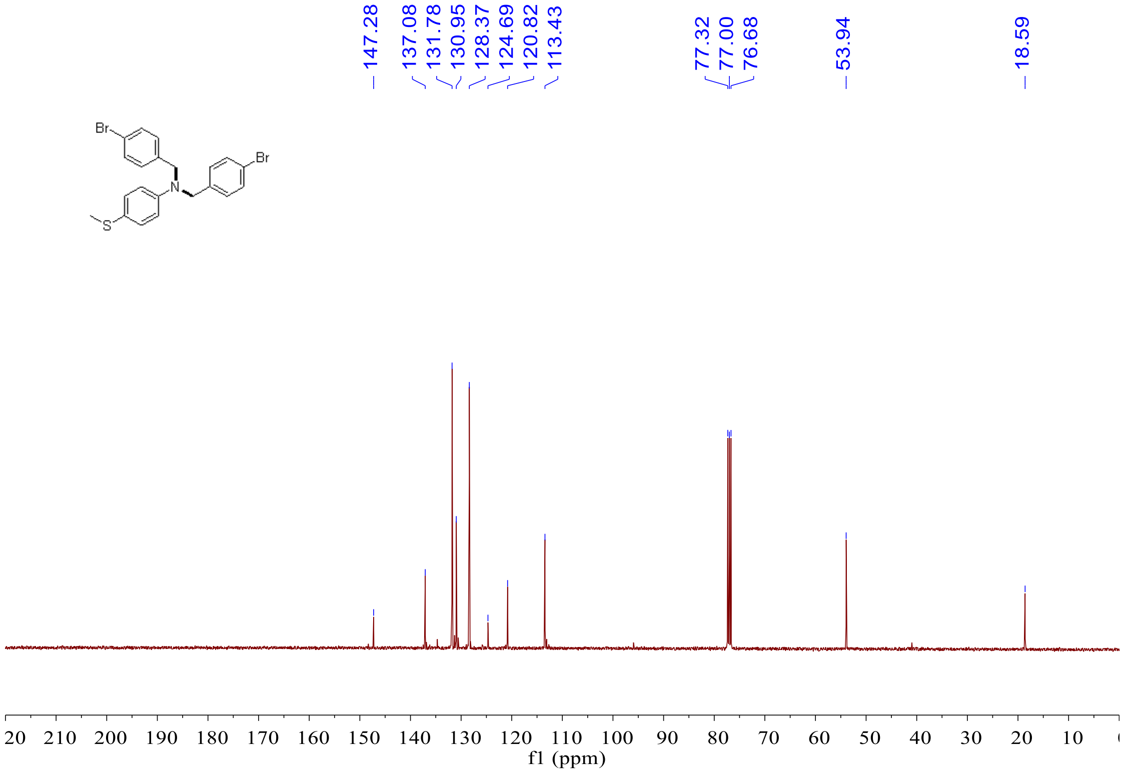

Supplementary Figure 40 ^{13}C NMR spectrum for compound **17**

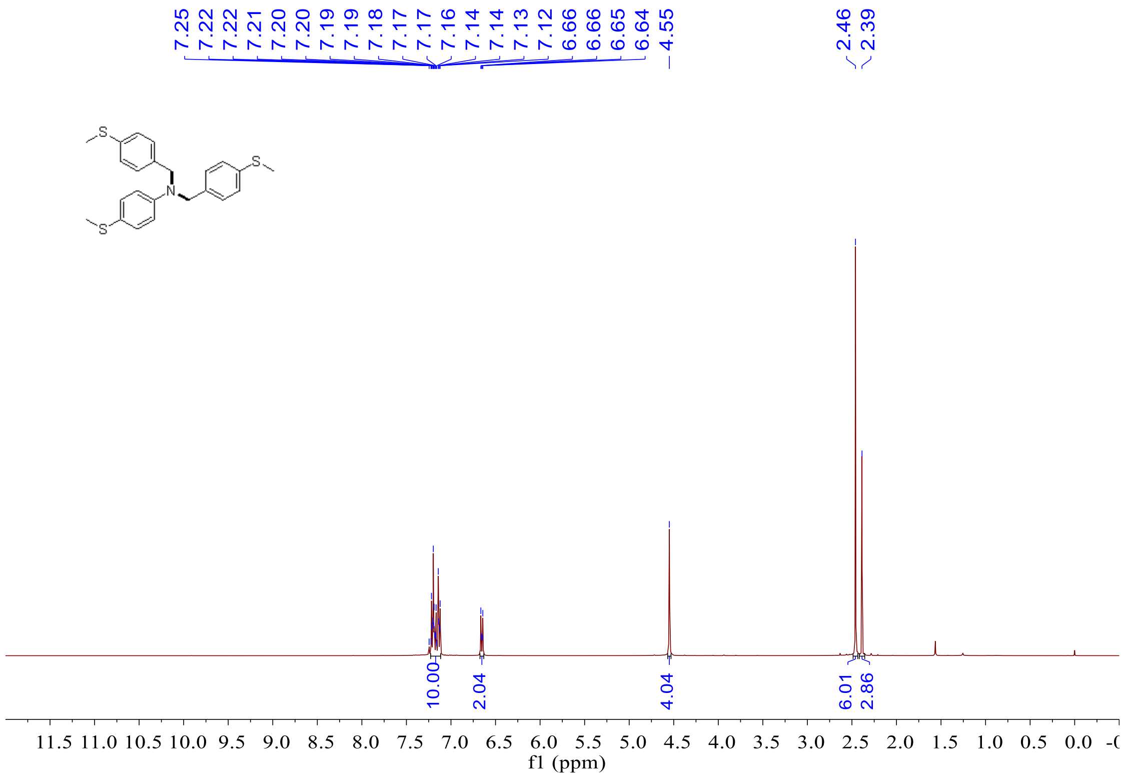

Supplementary Figure 41 ^{1}H NMR spectrum for compound **18**

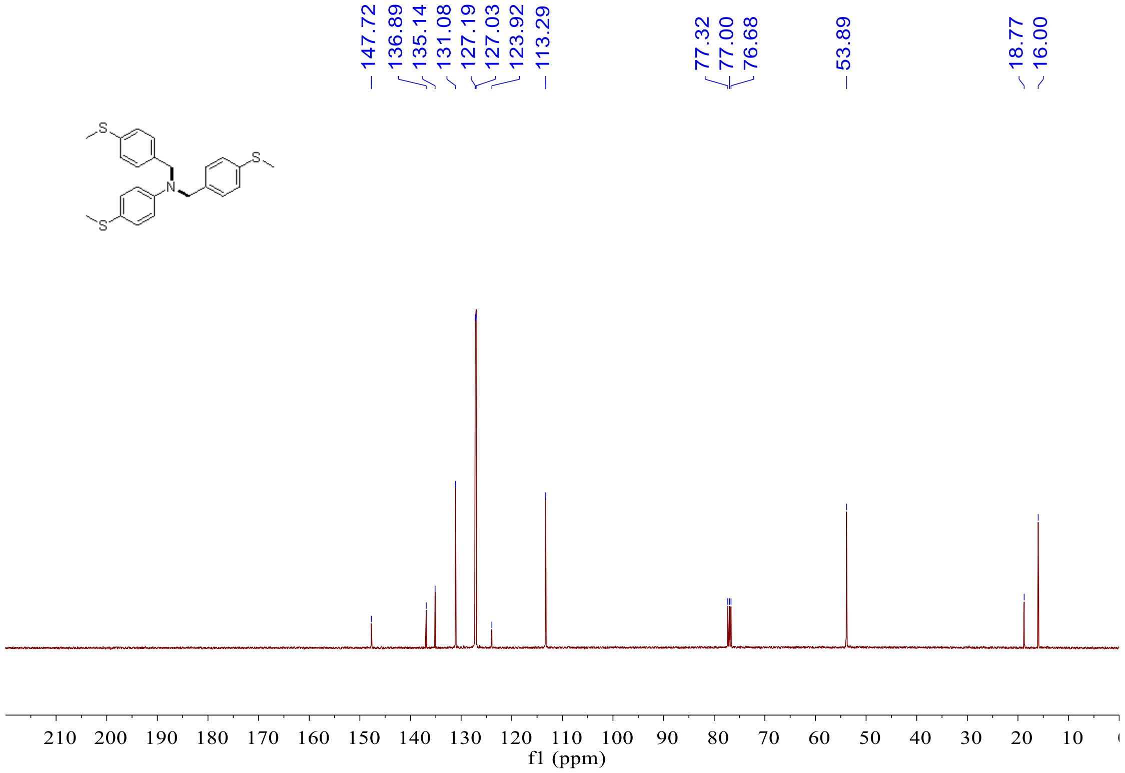

Supplementary Figure 42 ^{13}C NMR spectrum for compound **18**

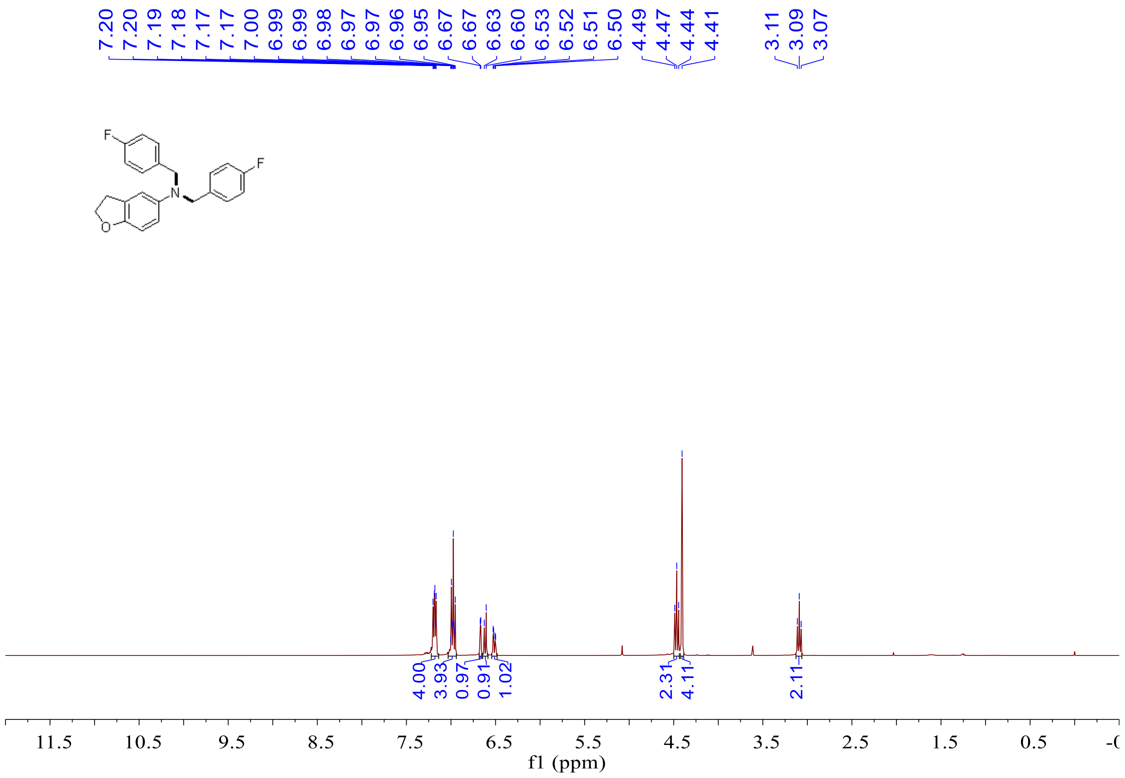

Supplementary Figure 43 ^{1}H NMR spectrum for compound **19**

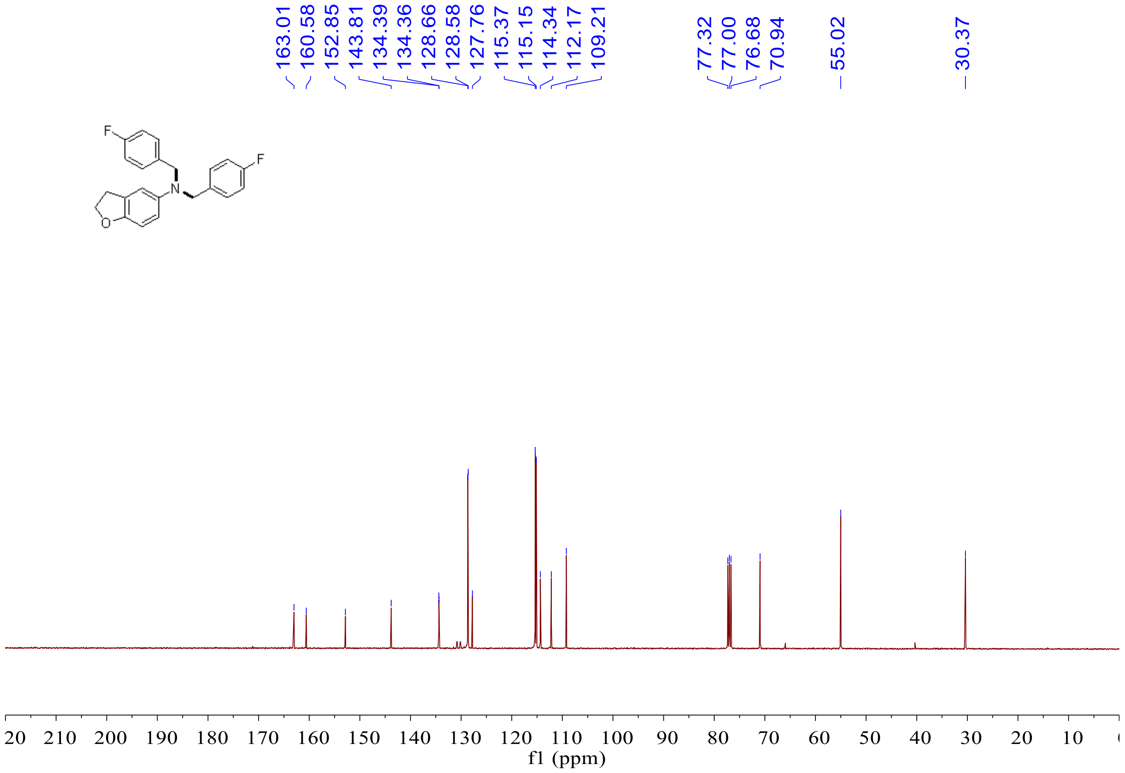

Supplementary Figure 44 ^{13}C NMR spectrum for compound **19**

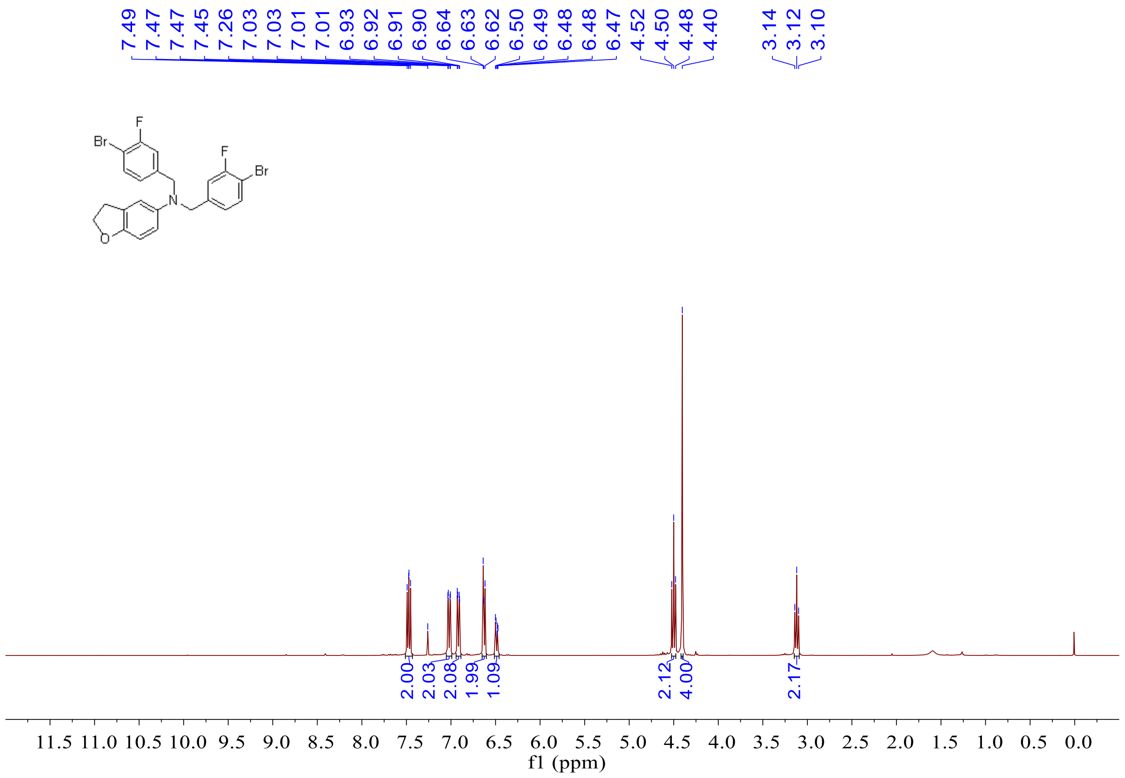

Supplementary Figure 45 ^{1}H NMR spectrum for compound **20**

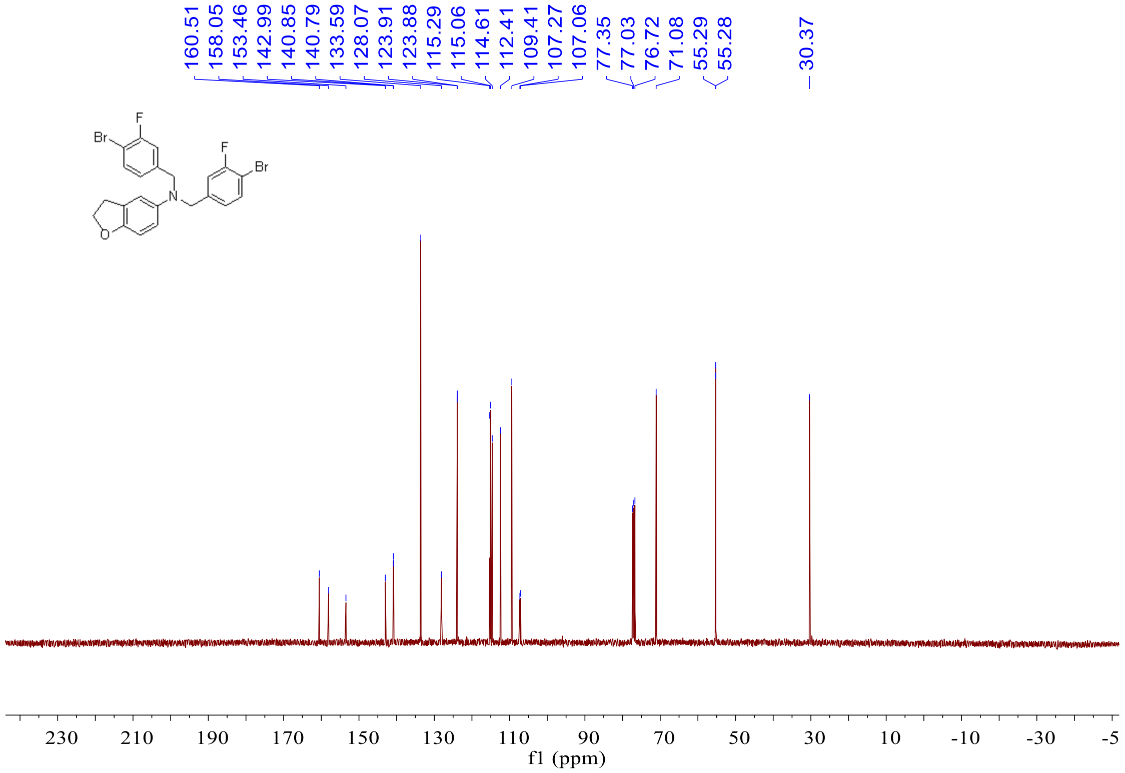

Supplementary Figure 46 ^{13}C NMR spectrum for compound 2**0**

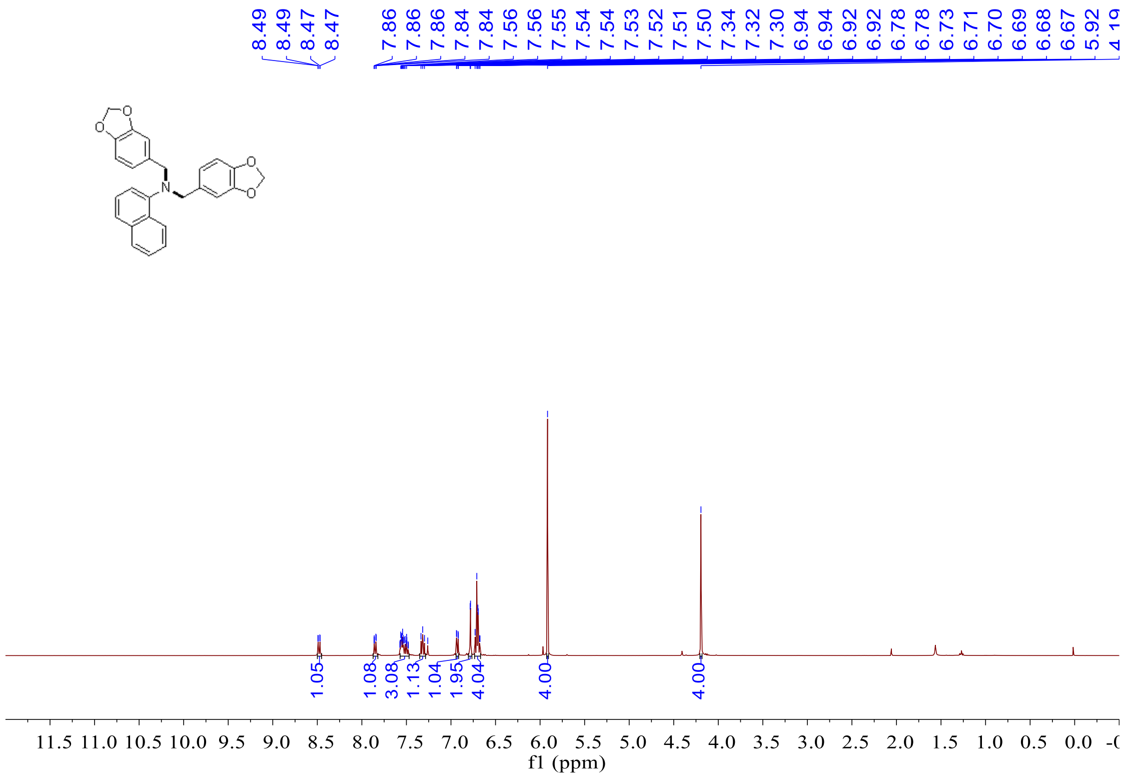

Supplementary Figure 47 ^{1}H NMR spectrum for compound **21**

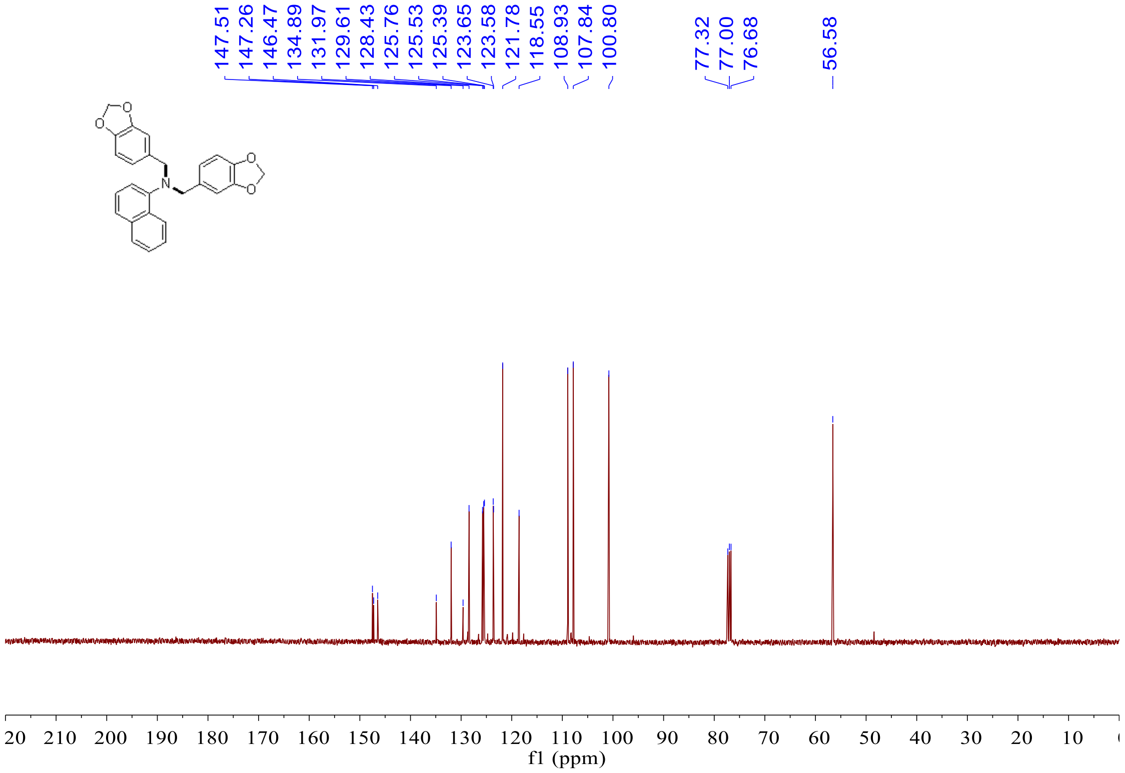

Supplementary Figure 48 ^{13}C NMR spectrum for compound **21**

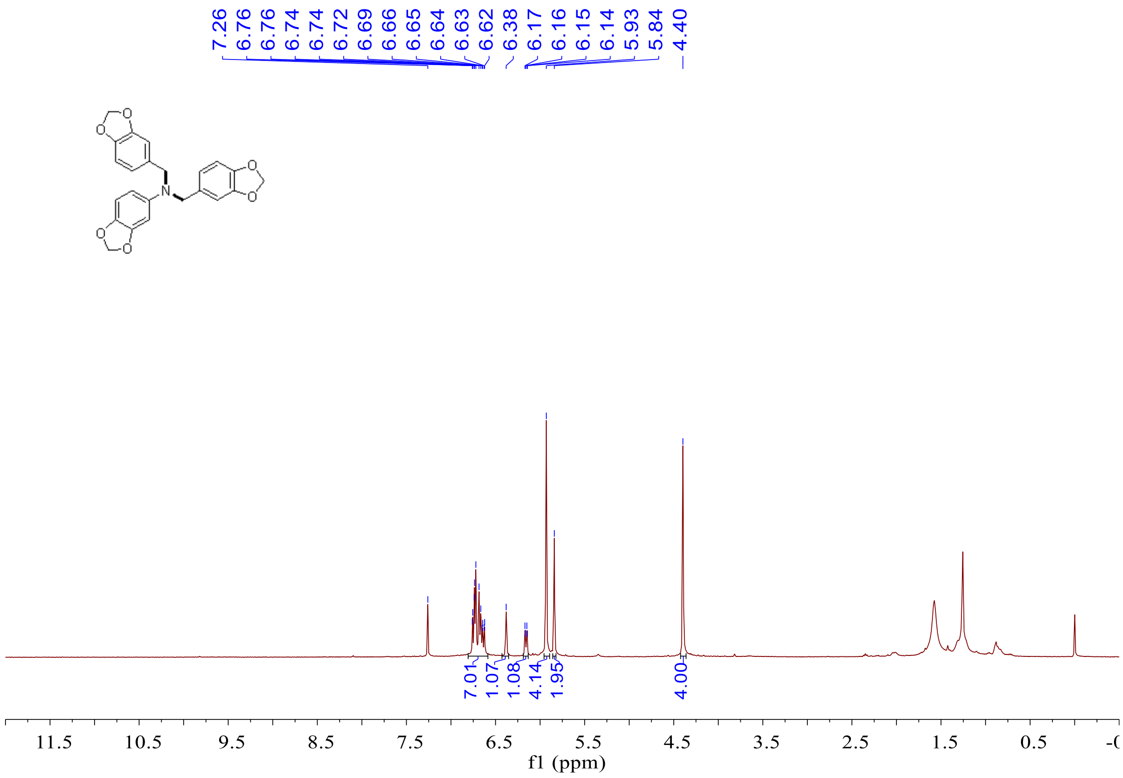

Supplementary Figure 49 ^{1}H NMR spectrum for compound **22**

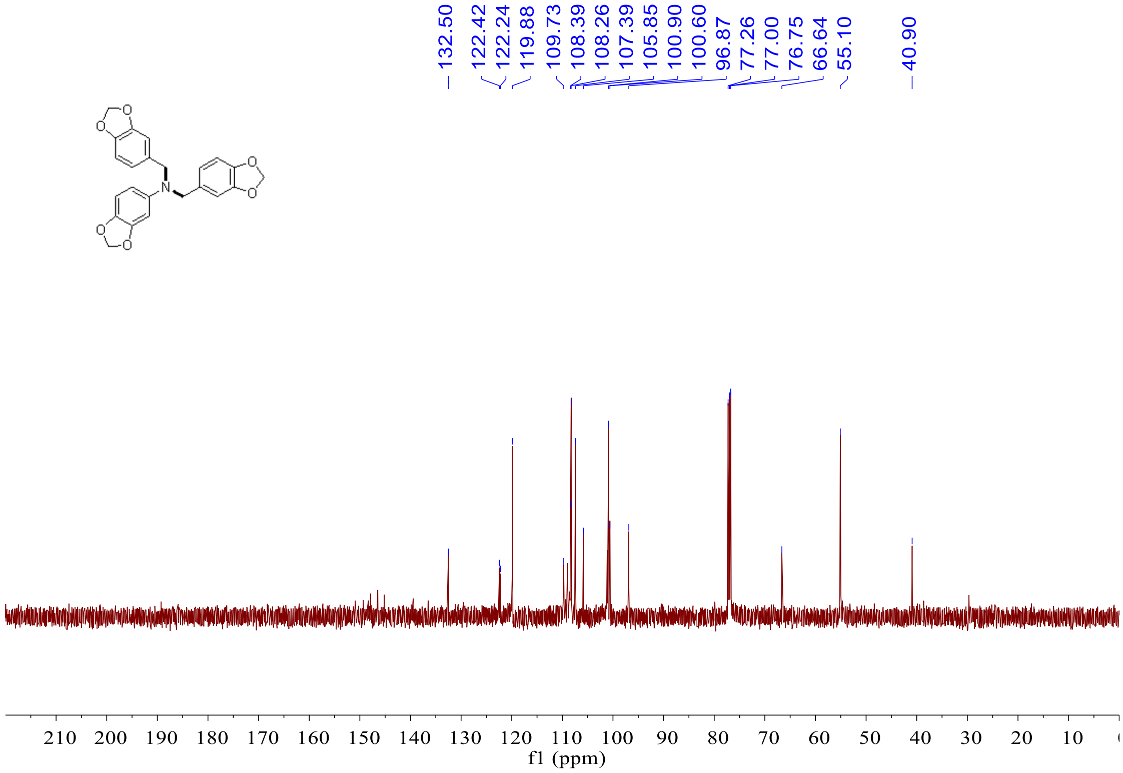

Supplementary Figure 50 ^{13}C NMR spectrum for compound **22**

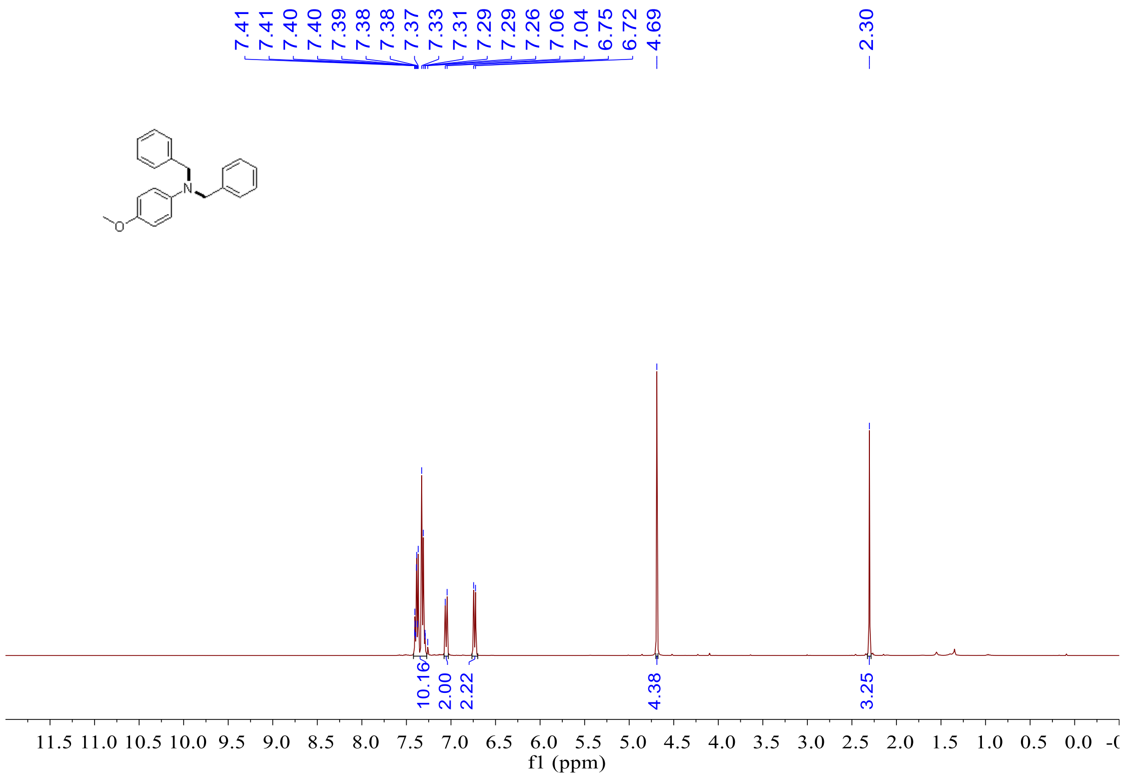

Supplementary Figure 51 ^{1}H NMR spectrum for compound **23**

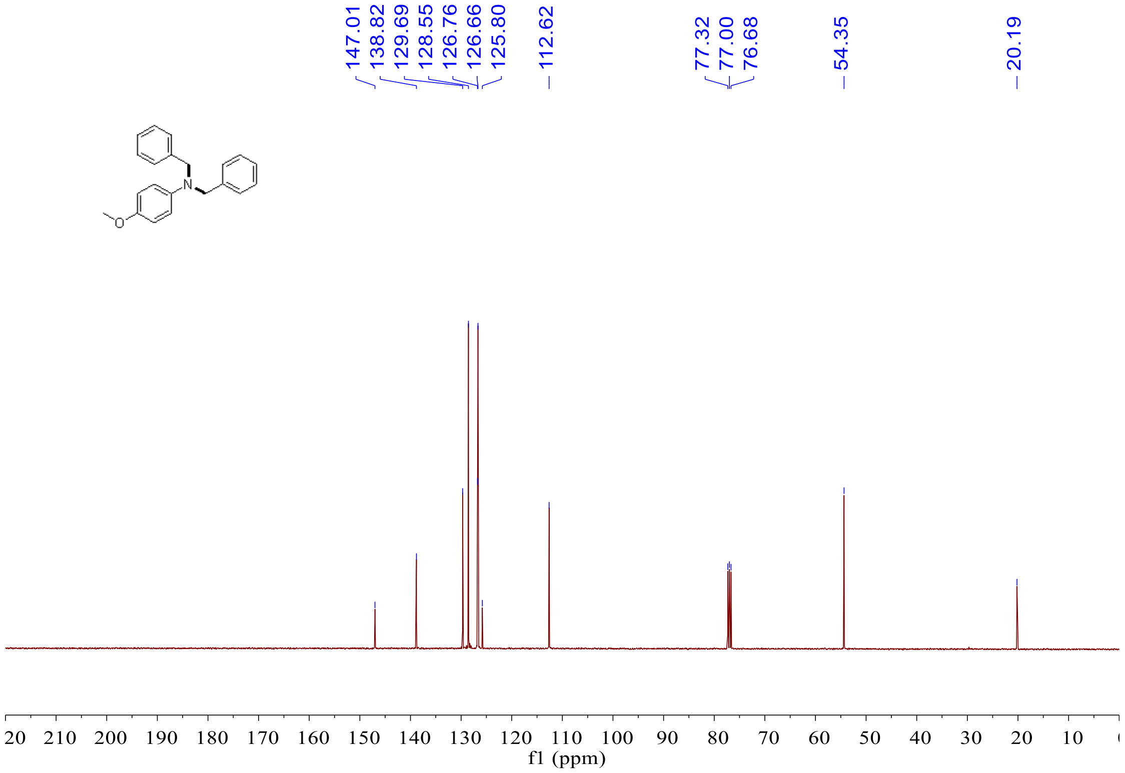

Supplementary Figure 52 ^{13}C NMR spectrum for compound **23**

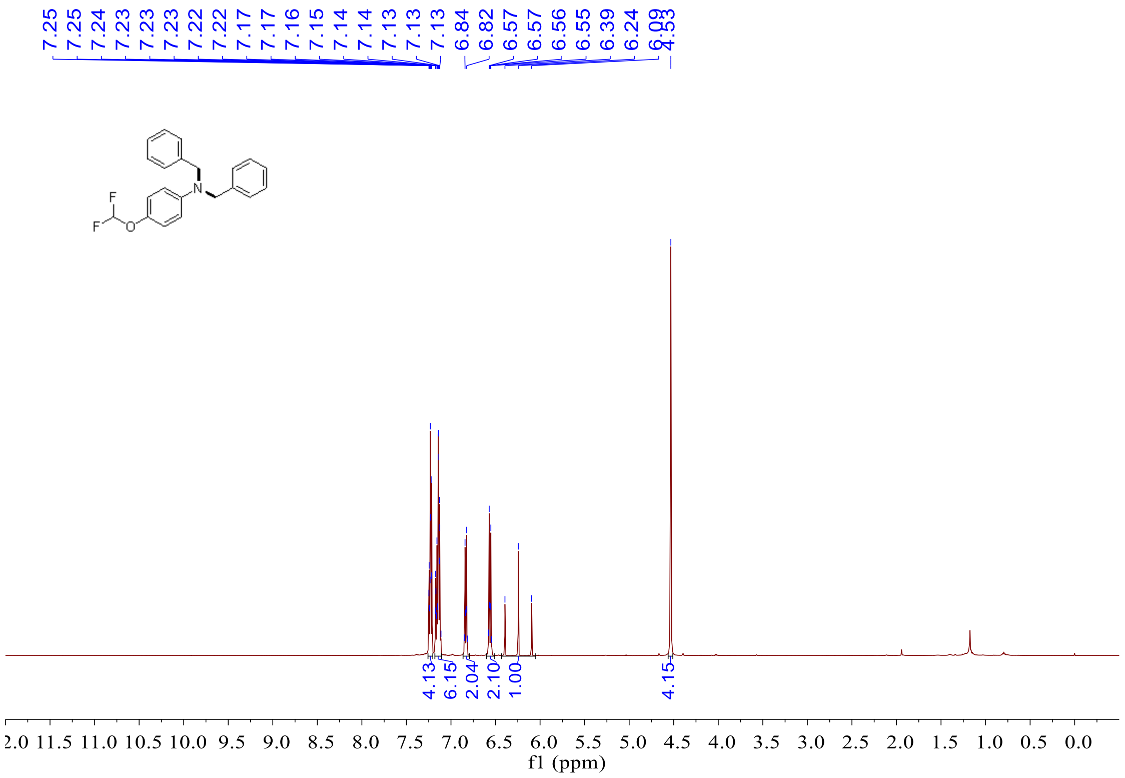

Supplementary Figure 53 ^{1}H NMR spectrum for compound **24**

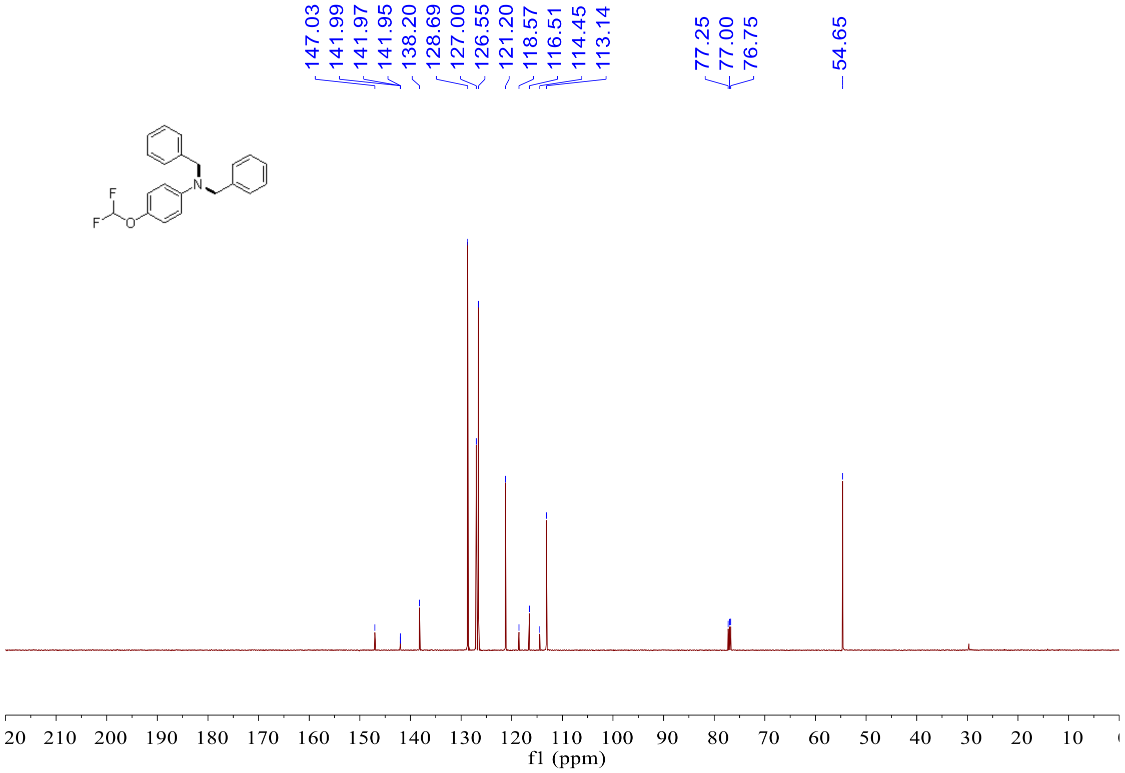

Supplementary Figure 54 ^{13}C NMR spectrum for compound **24**

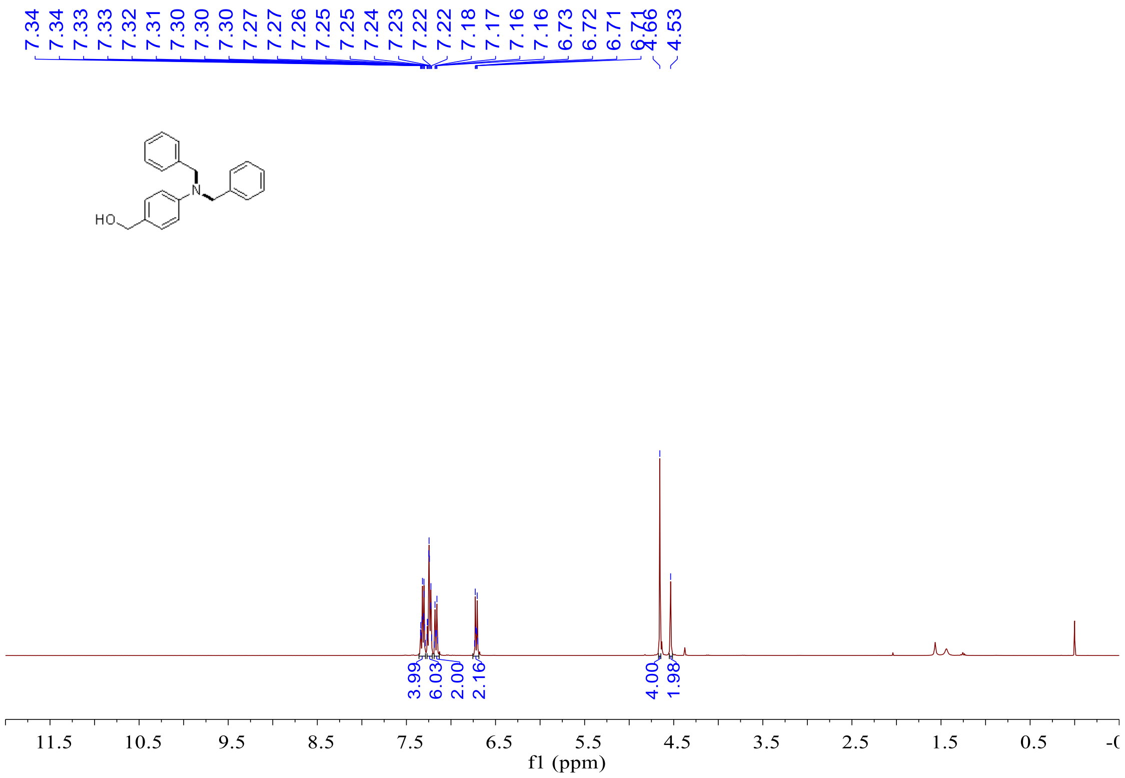

Supplementary Figure 55 ^{1}H NMR spectrum for compound **25**

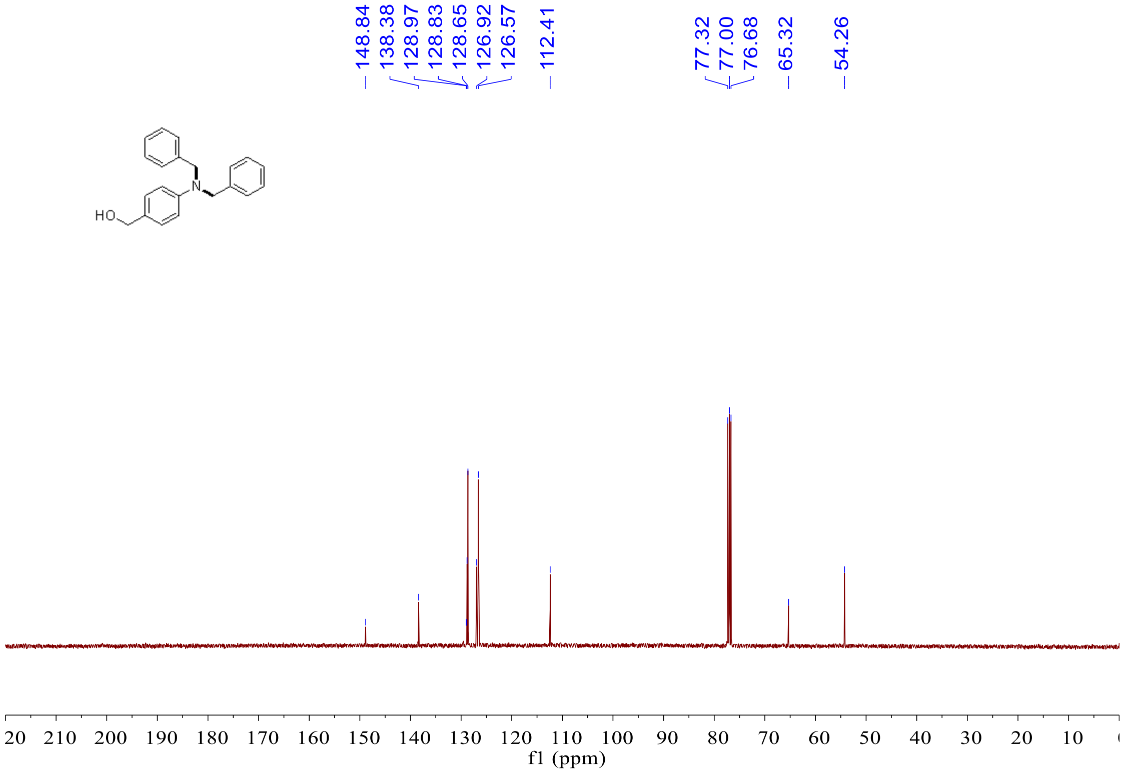

Supplementary Figure 56 ^{13}C NMR spectrum for compound **25**

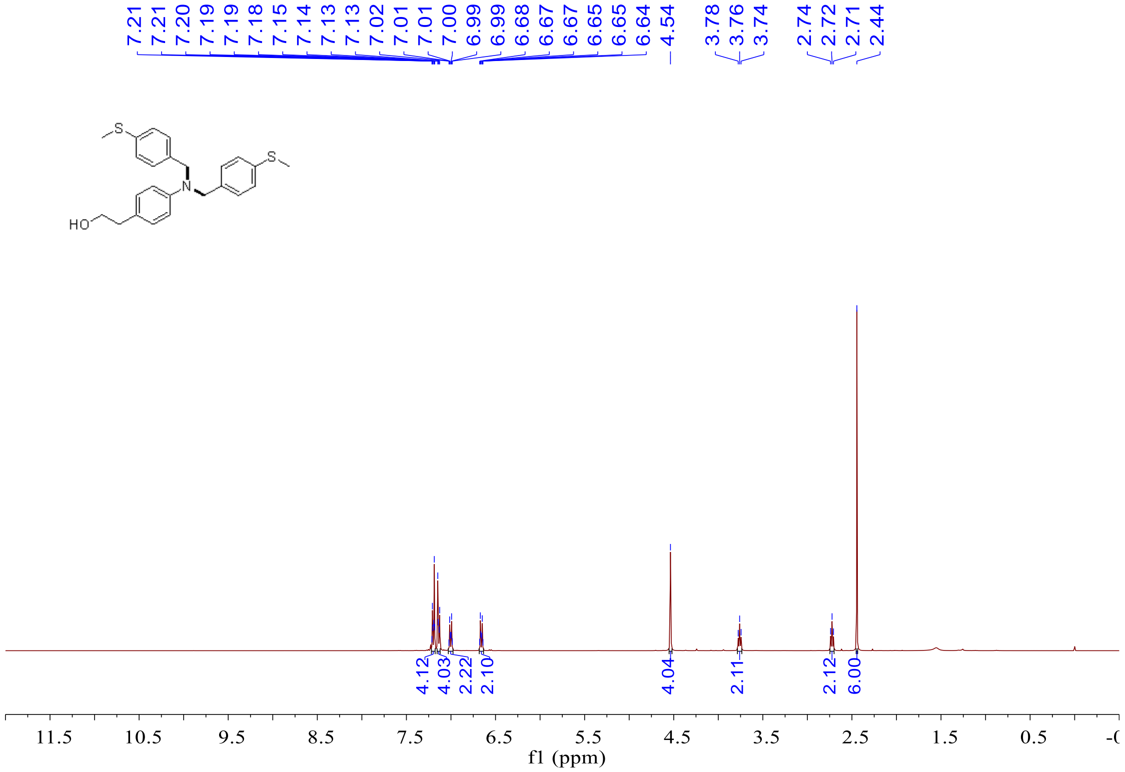

Supplementary Figure 57 ^{1}H NMR spectrum for compound **26**

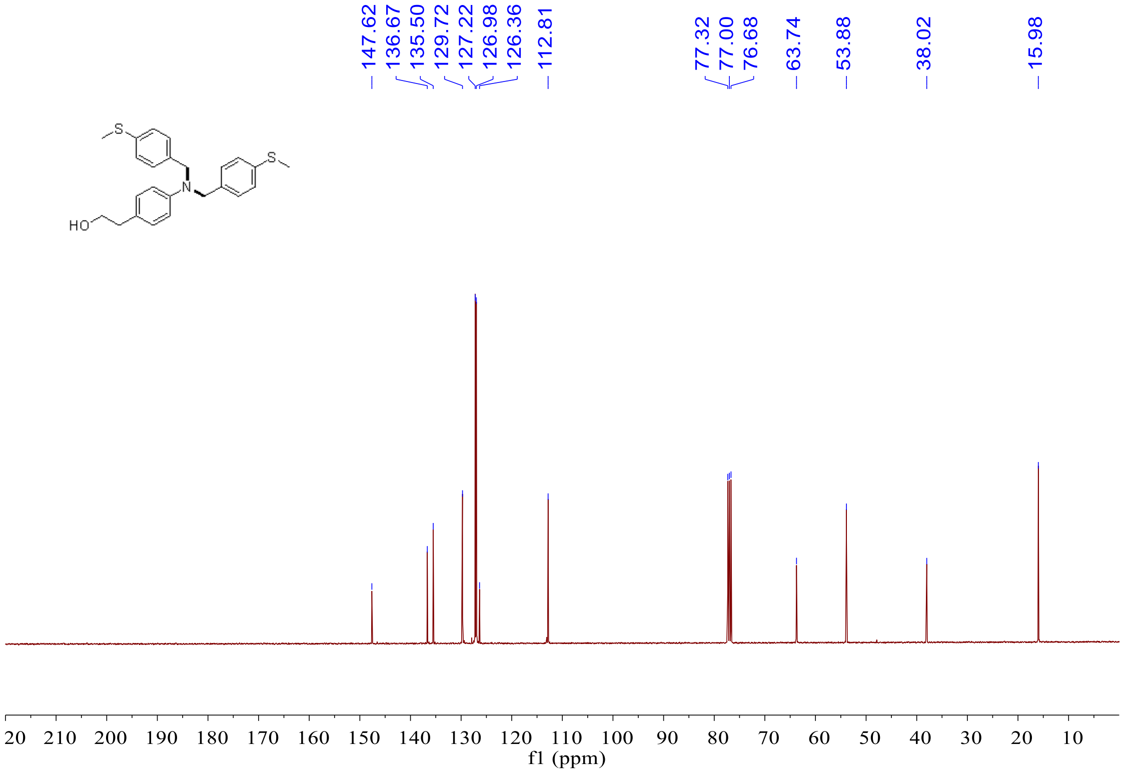

Supplementary Figure 58 ^{13}C NMR spectrum for compound **26**

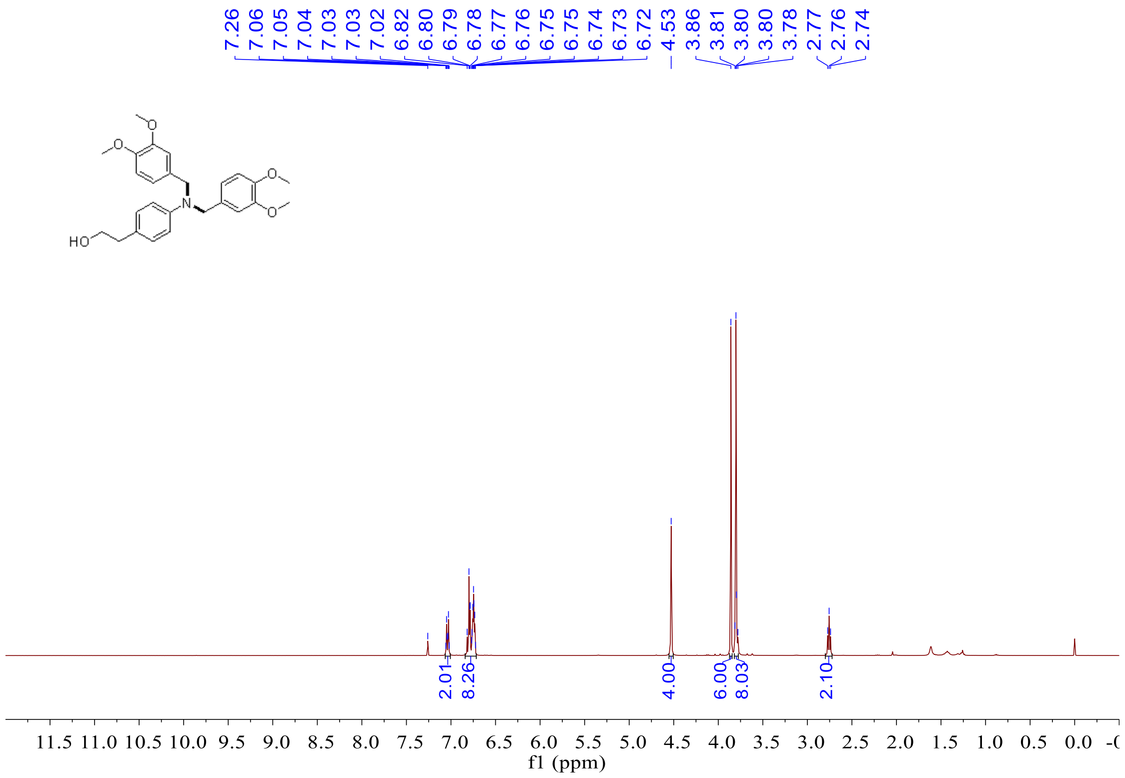

Supplementary Figure 59 ^{1}H NMR spectrum for compound **27**

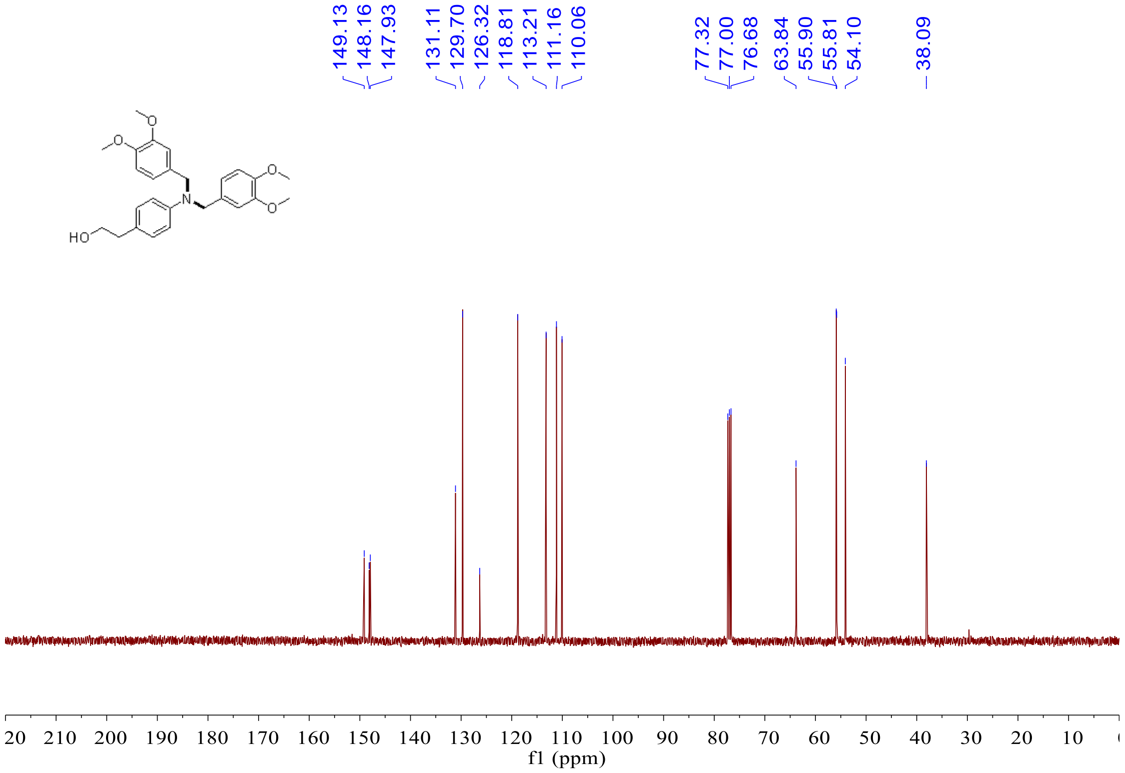

Supplementary Figure 60 ^{13}C NMR spectrum for compound **27**

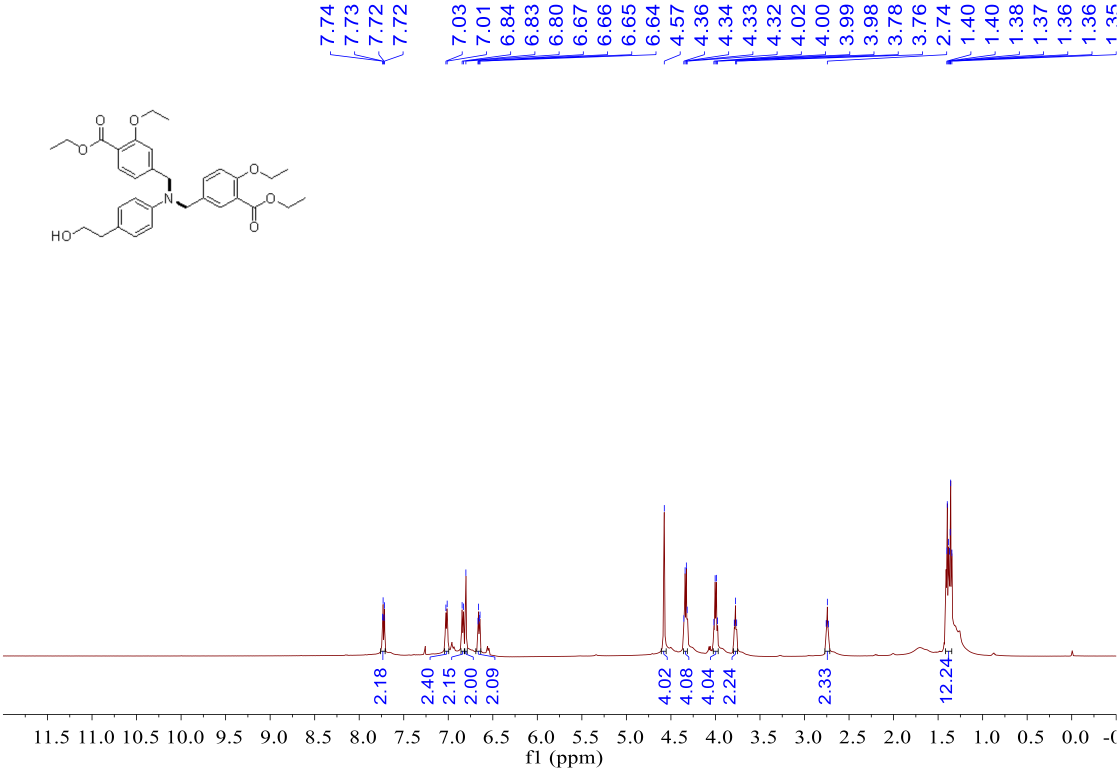

Supplementary Figure 61 ^{1}H NMR spectrum for compound **28**

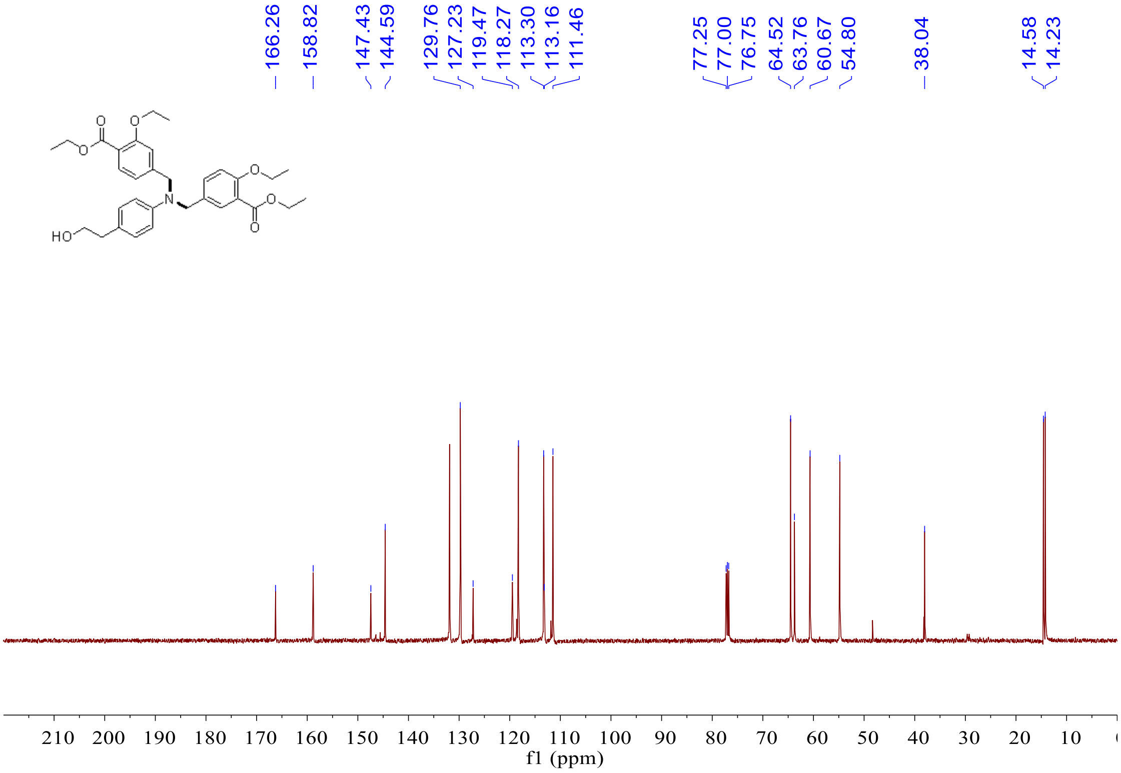

Supplementary Figure 62 ^{13}C NMR spectrum for compound **28**

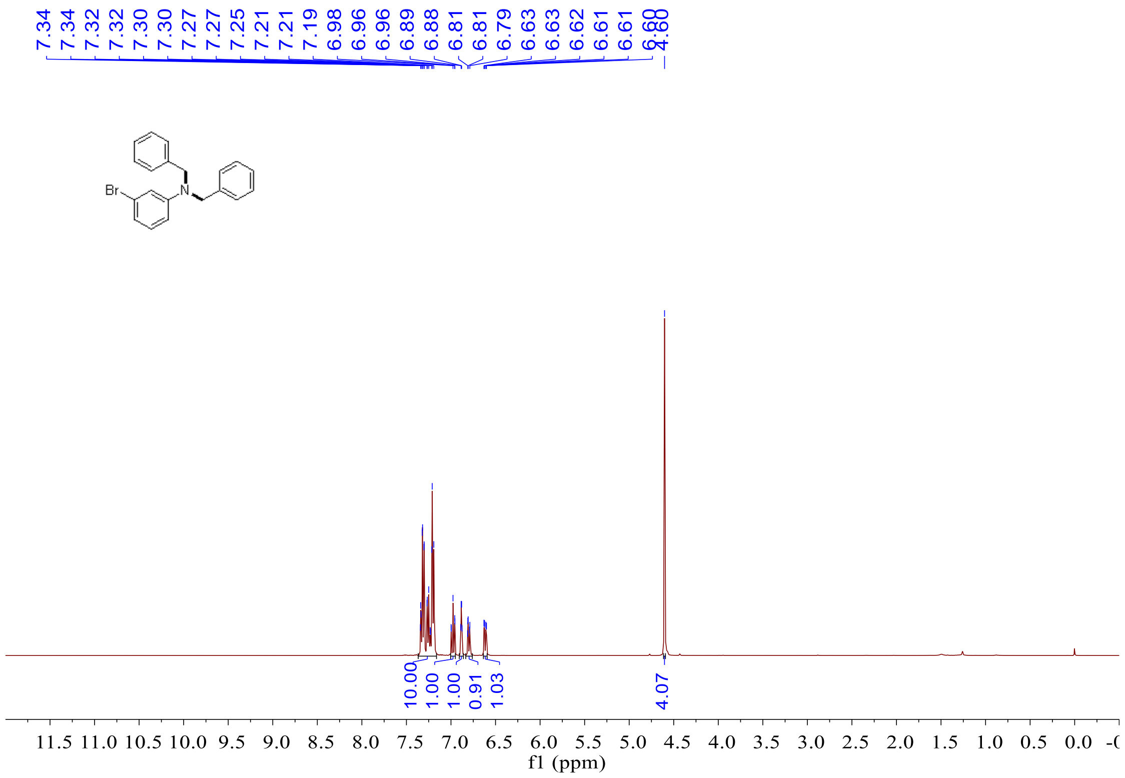

Supplementary Figure 63 ^{1}H NMR spectrum for compound **29**

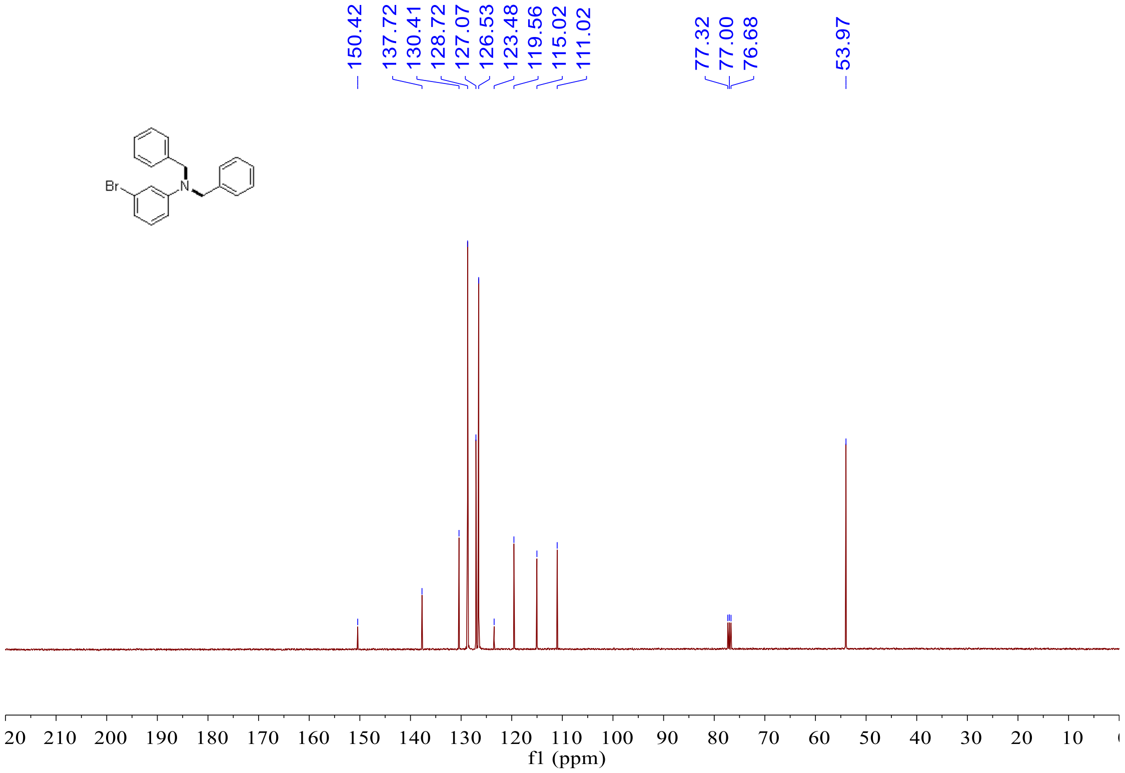

Supplementary Figure 64 ^{13}C NMR spectrum for compound **29**

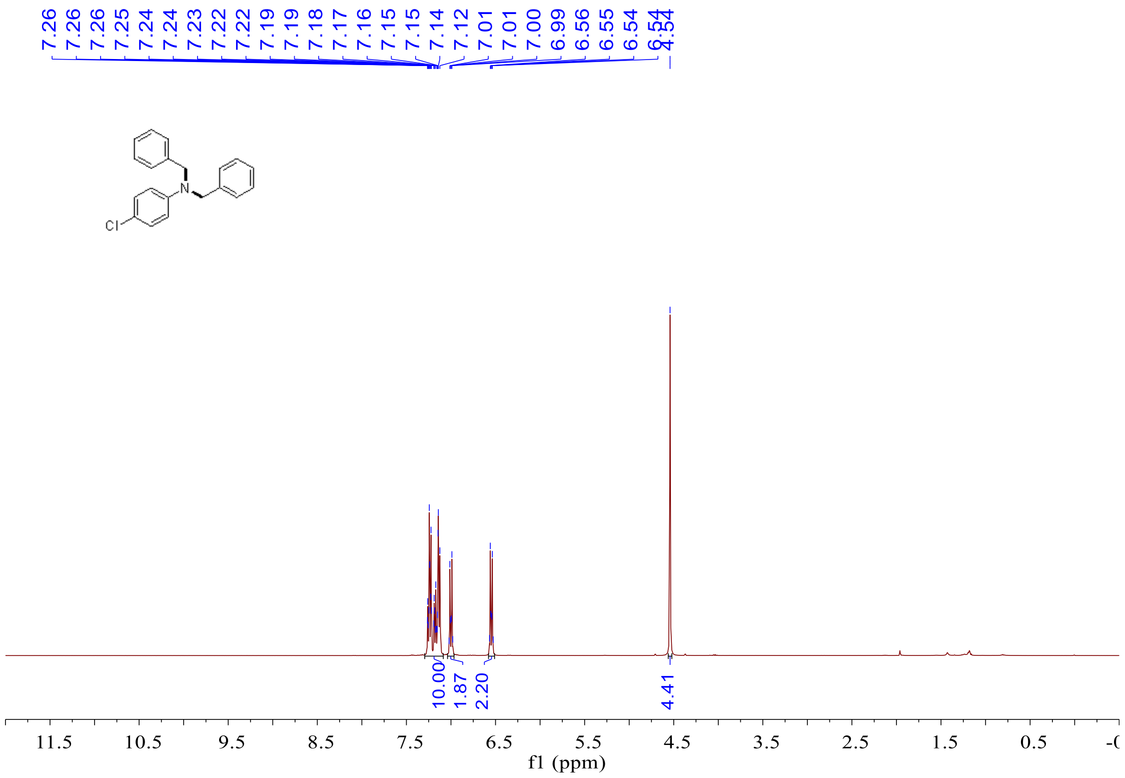

Supplementary Figure 65 ^{1}H NMR spectrum for compound **30**

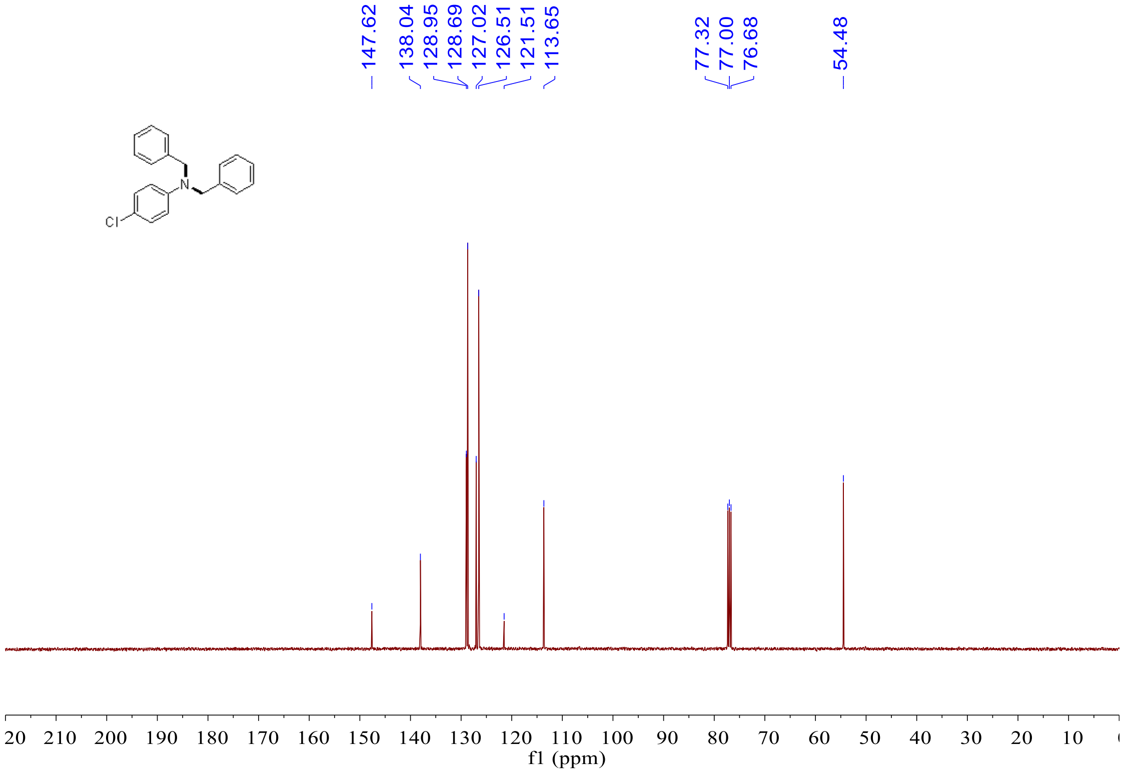

Supplementary Figure 66 ^{13}C NMR spectrum for compound **30**

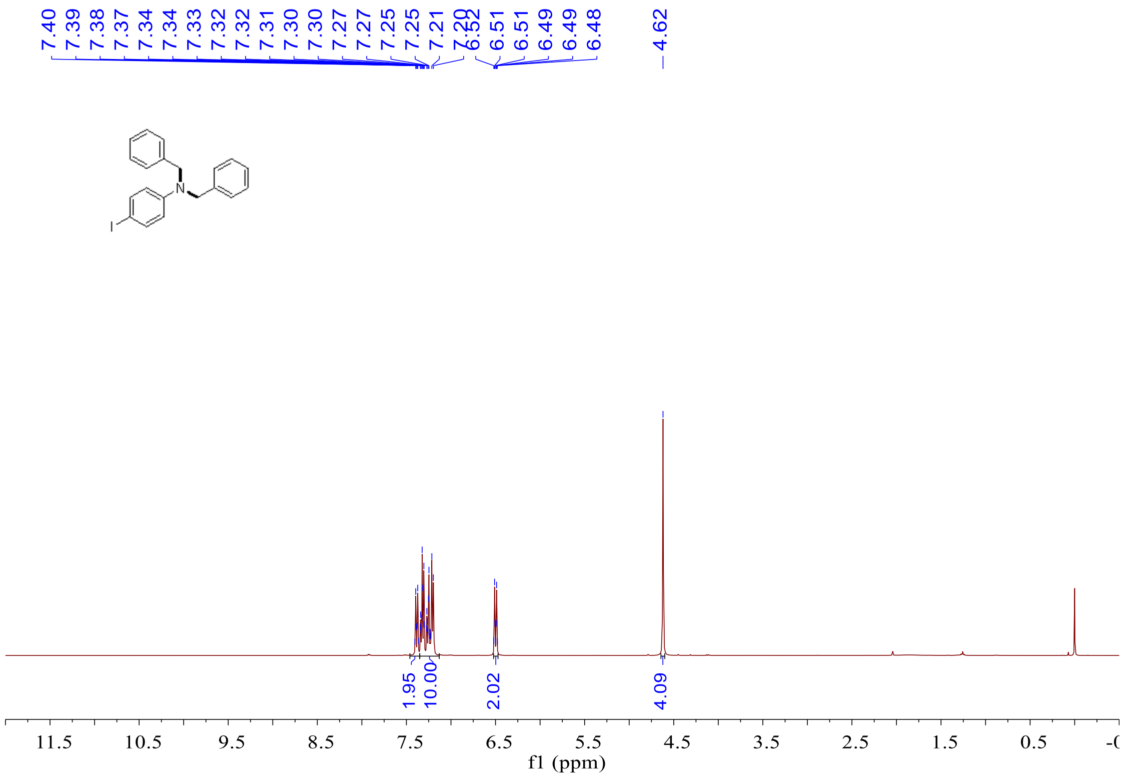

Supplementary Figure 67 ^{1}H NMR spectrum for compound **31**

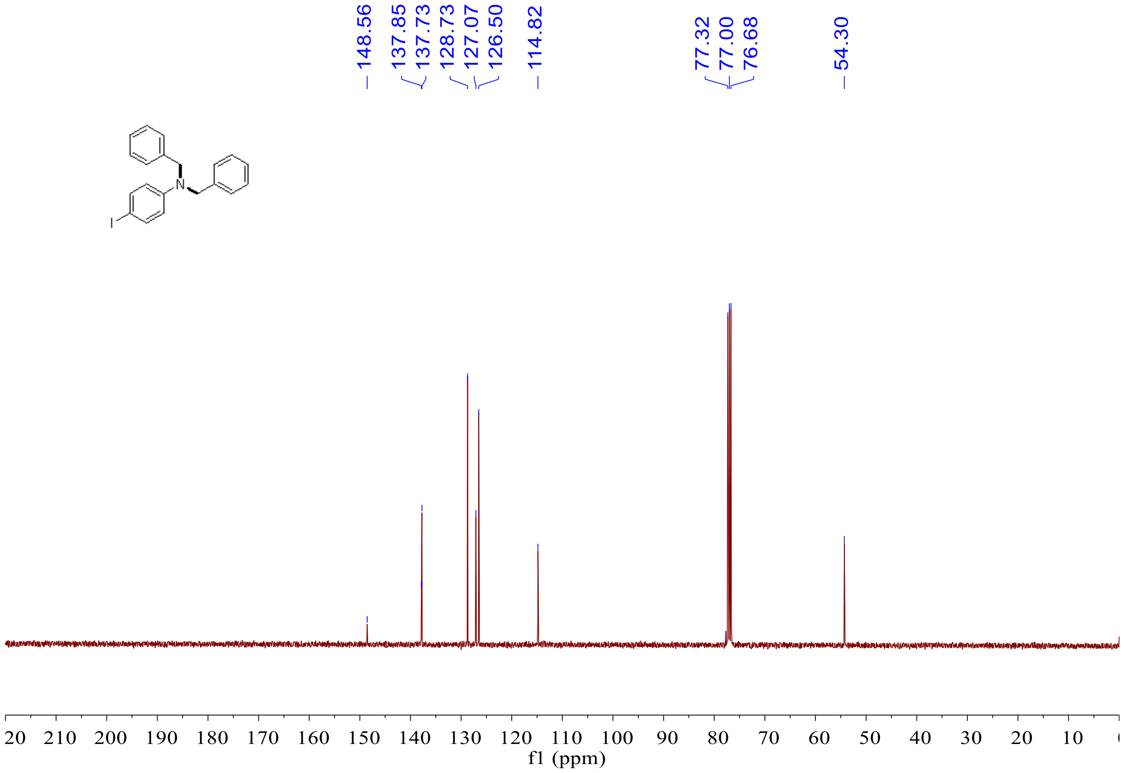

Supplementary Figure 68 ^{13}C NMR spectrum for compound **31**

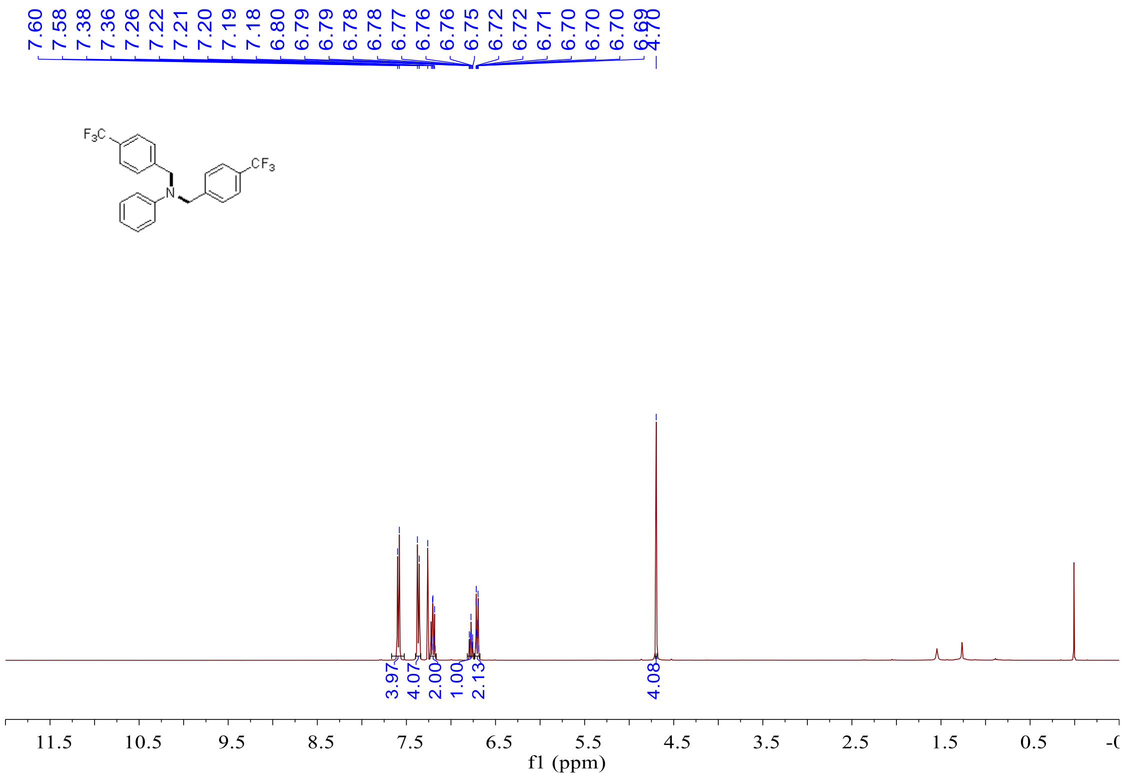

Supplementary Figure 69 ^{1}H NMR spectrum for compound **32**

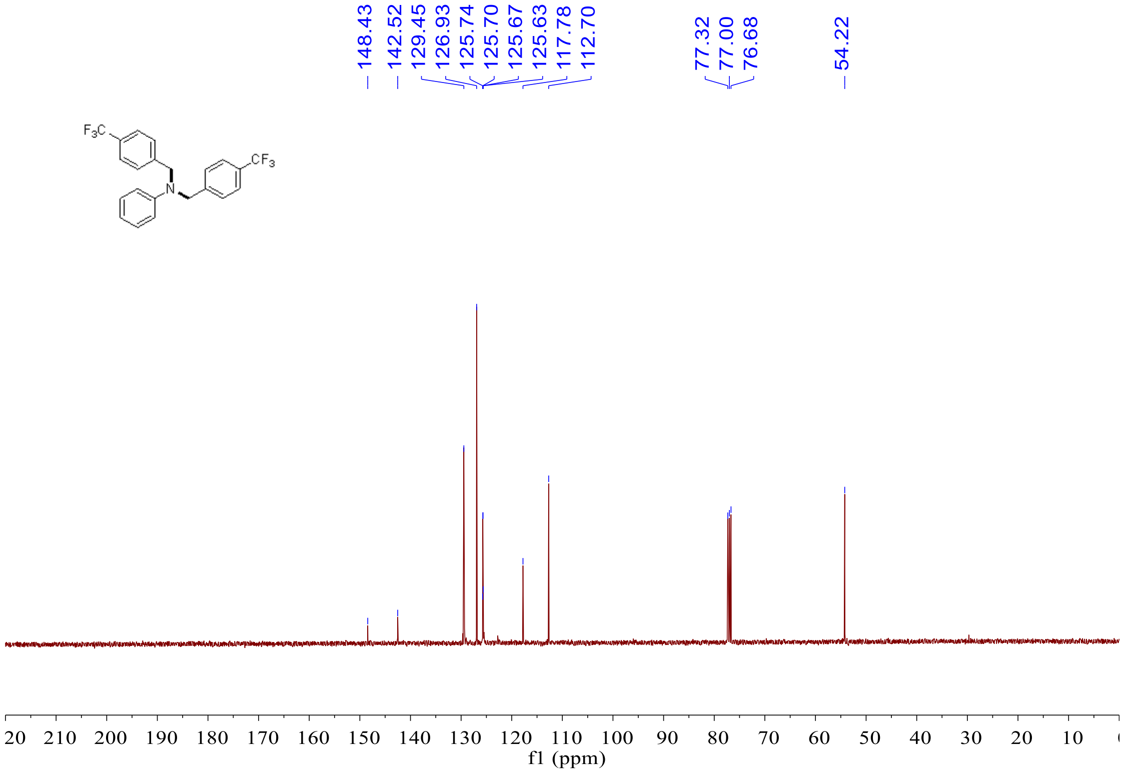

Supplementary Figure 70 ^{13}C NMR spectrum for compound **32**

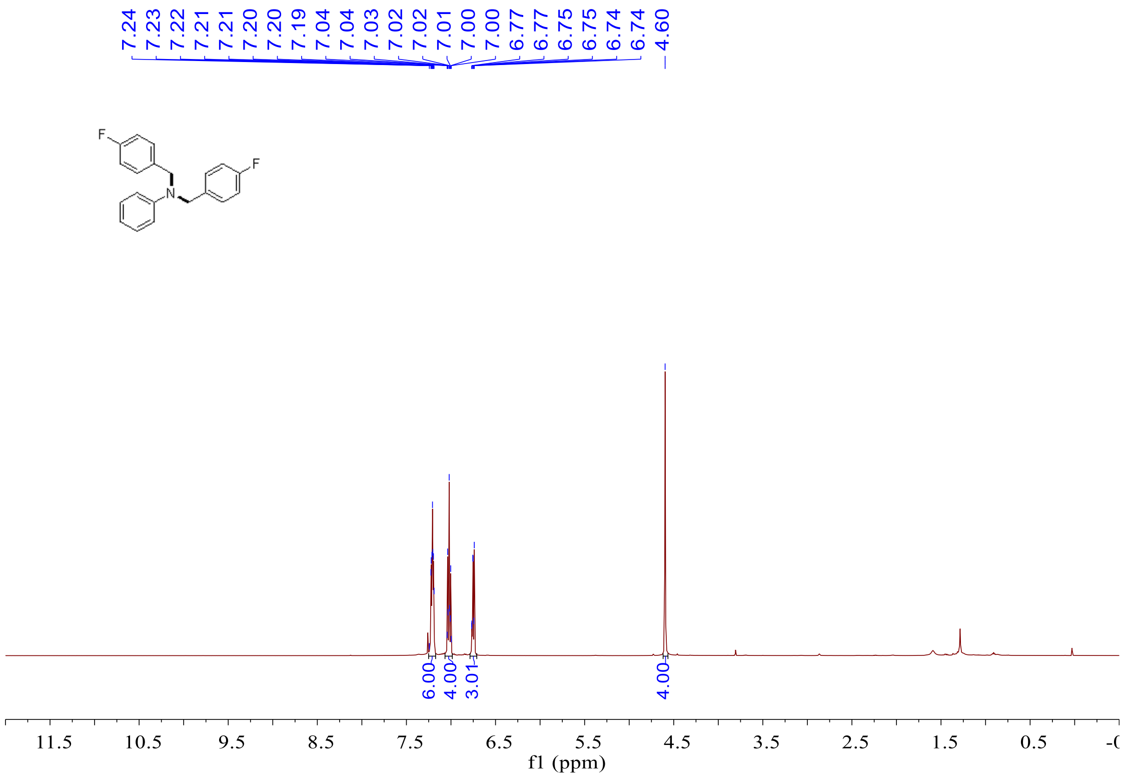

Supplementary Figure 71 ^{1}H NMR spectrum for compound **33**

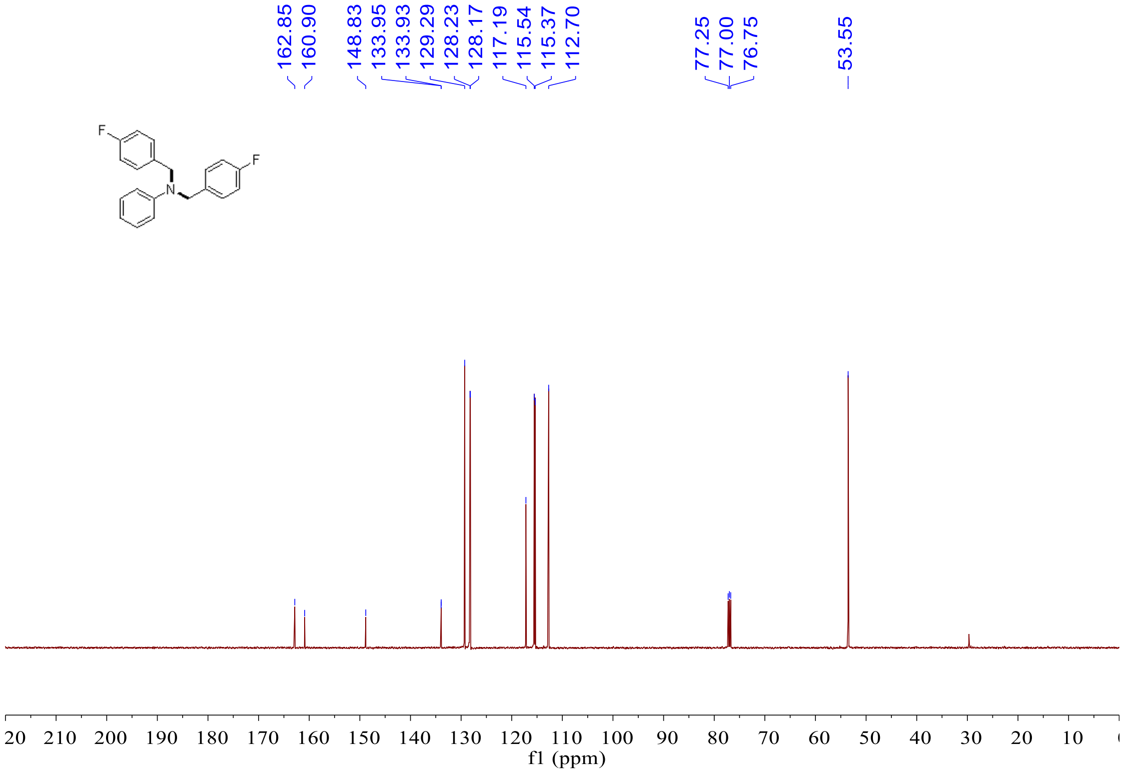

Supplementary Figure 72 ^{13}C NMR spectrum for compound **33**

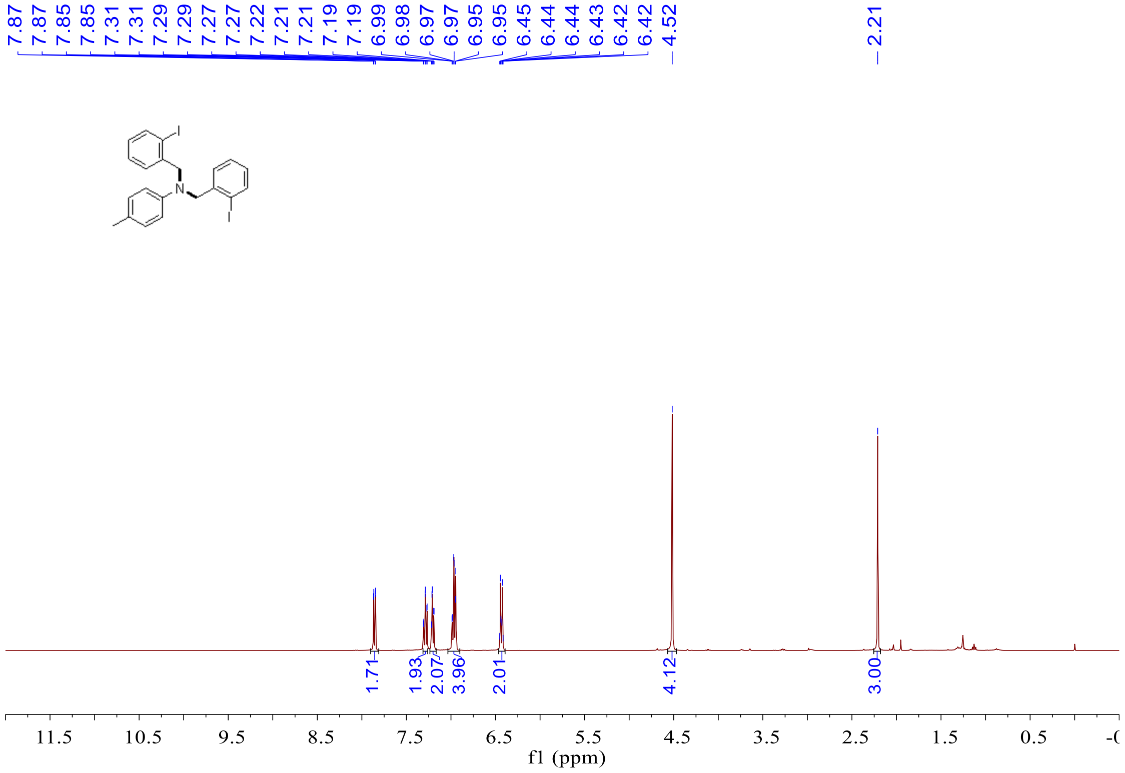

Supplementary Figure 73 ^{1}H NMR spectrum for compound **34**

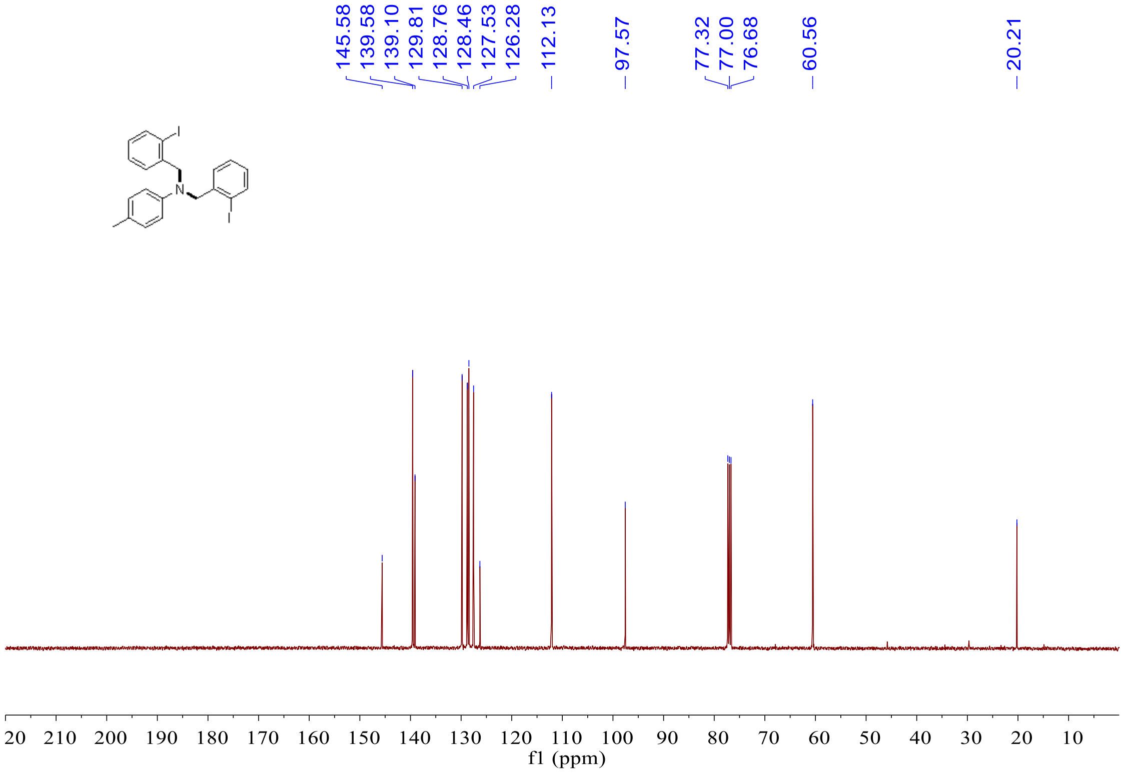

Supplementary Figure 74 ^{13}C NMR spectrum for compound **34**

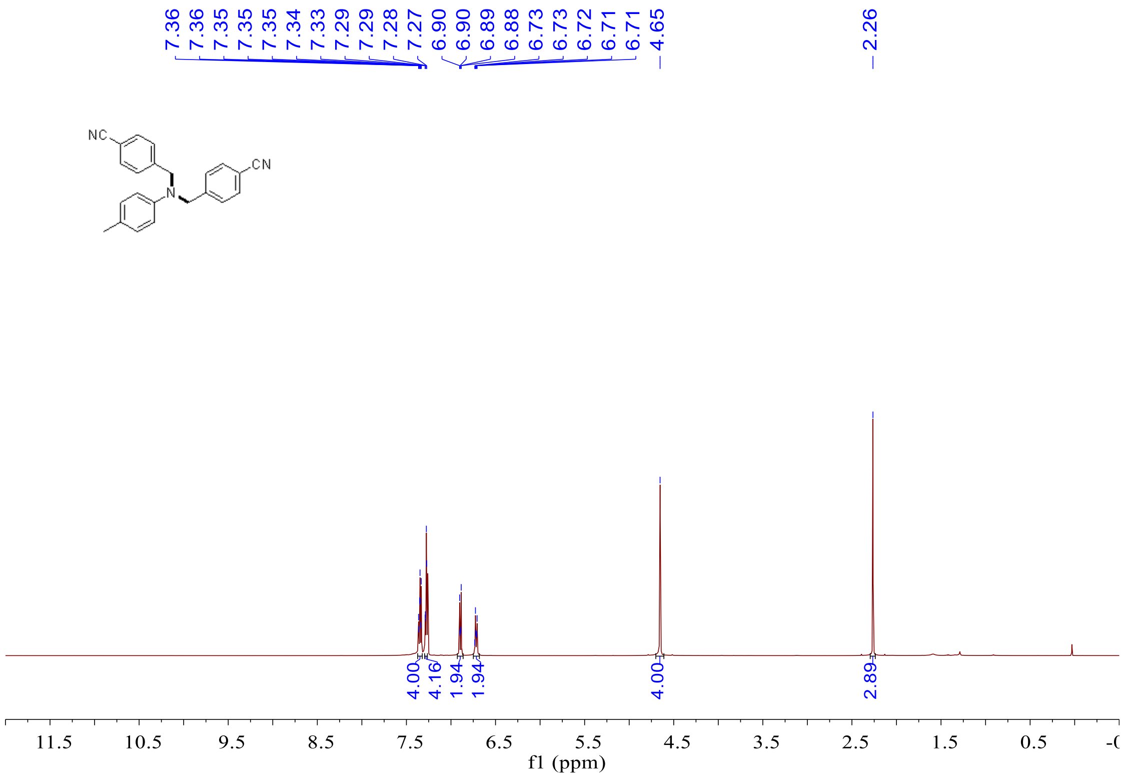

Supplementary Figure 75 ^{1}H NMR spectrum for compound **35**

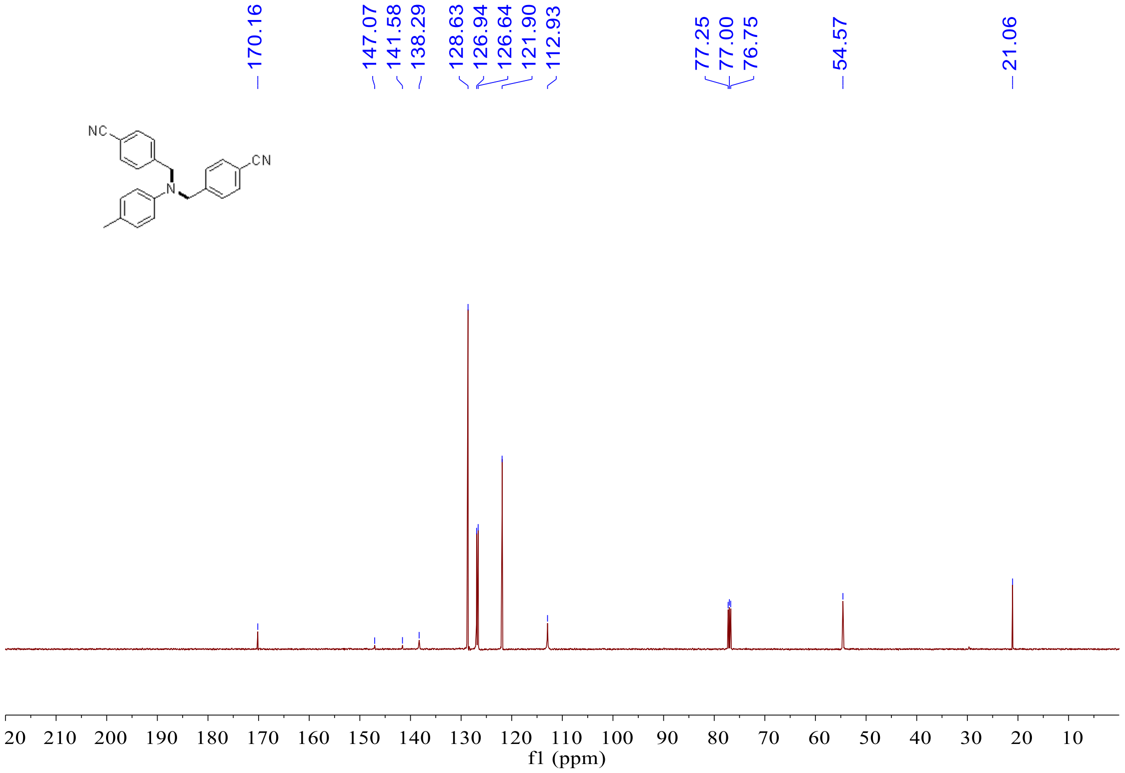

Supplementary Figure 76 ^{13}C NMR spectrum for compound **35**

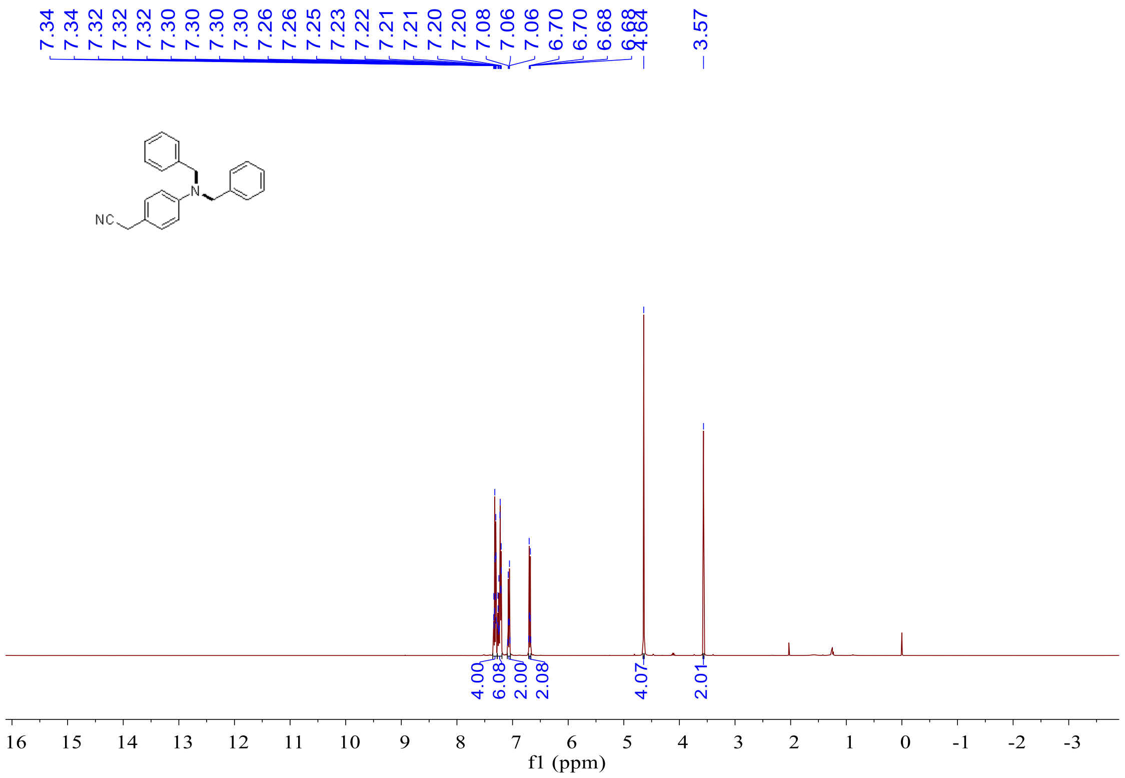

Supplementary Figure 77 ^{1}H NMR spectrum for compound **36**

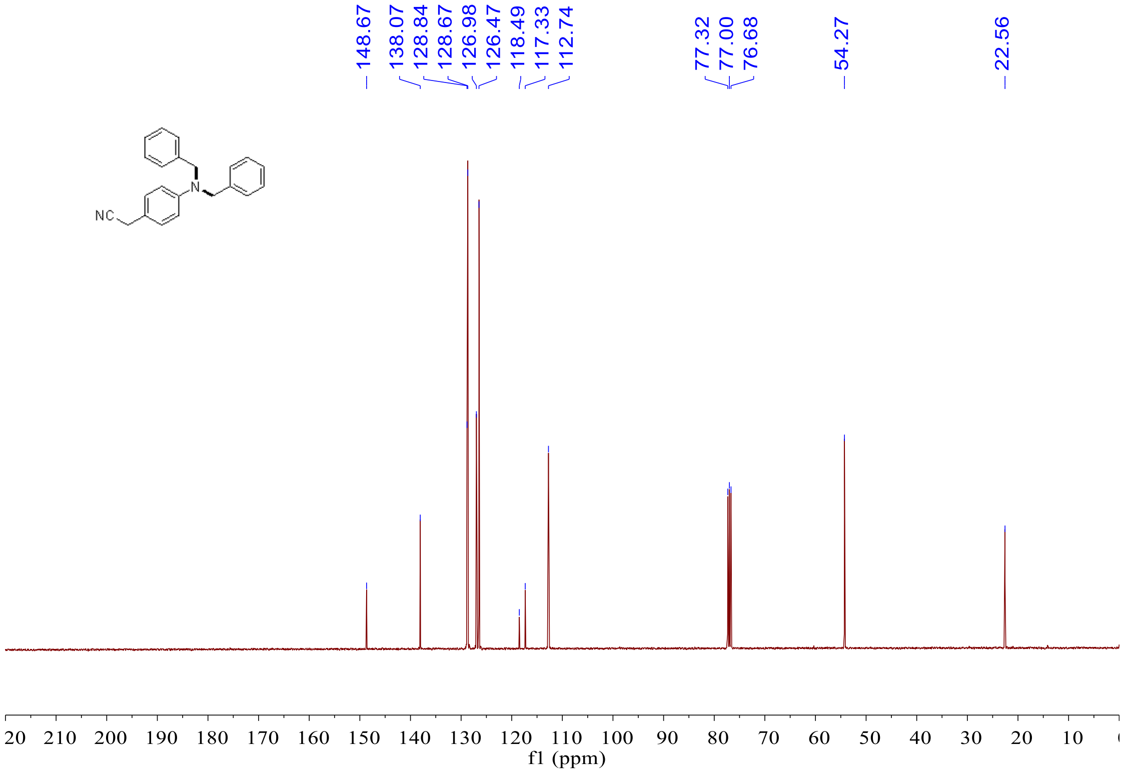

Supplementary Figure 78 ^{13}C NMR spectrum for compound **36**

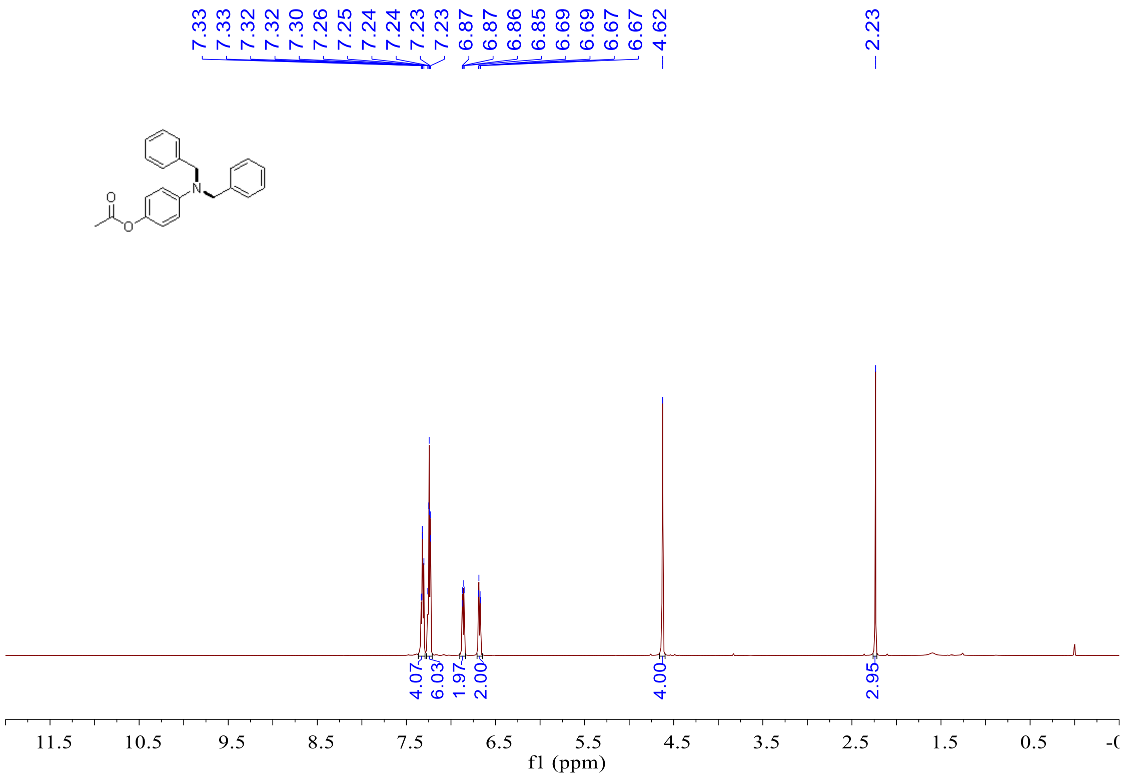

Supplementary Figure 79 ^{1}H NMR spectrum for compound **37**

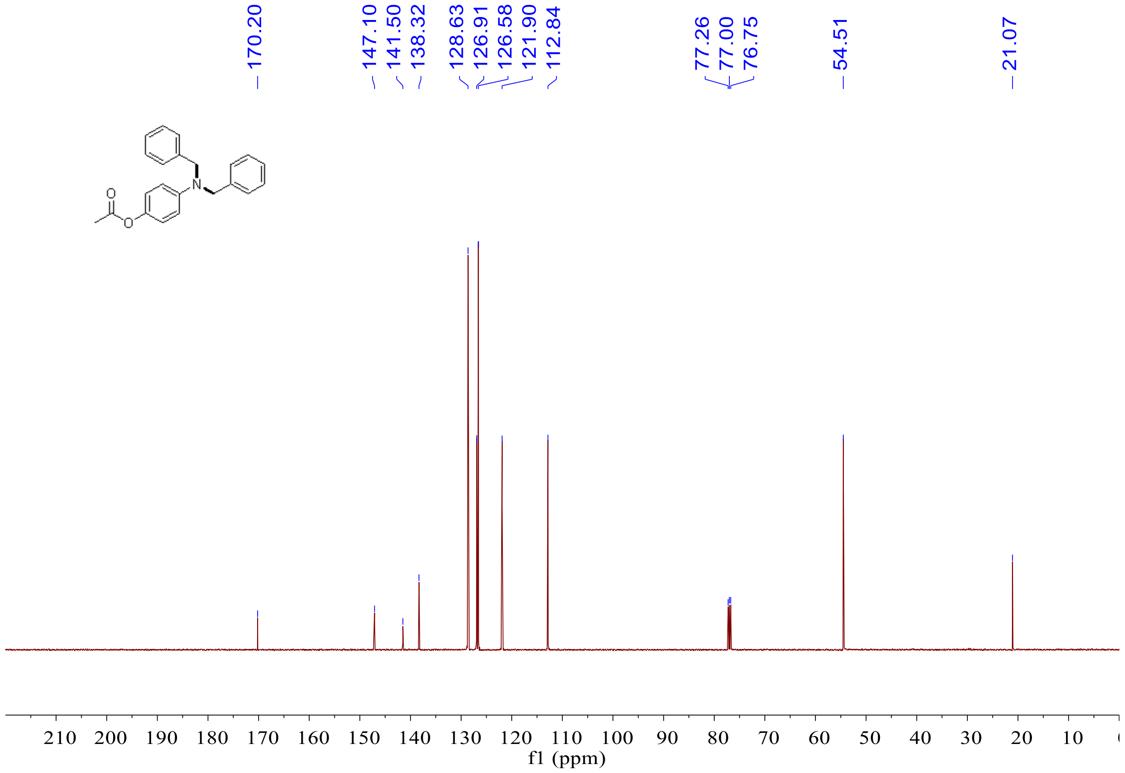

Supplementary Figure 80 ^{13}C NMR spectrum for compound **37**

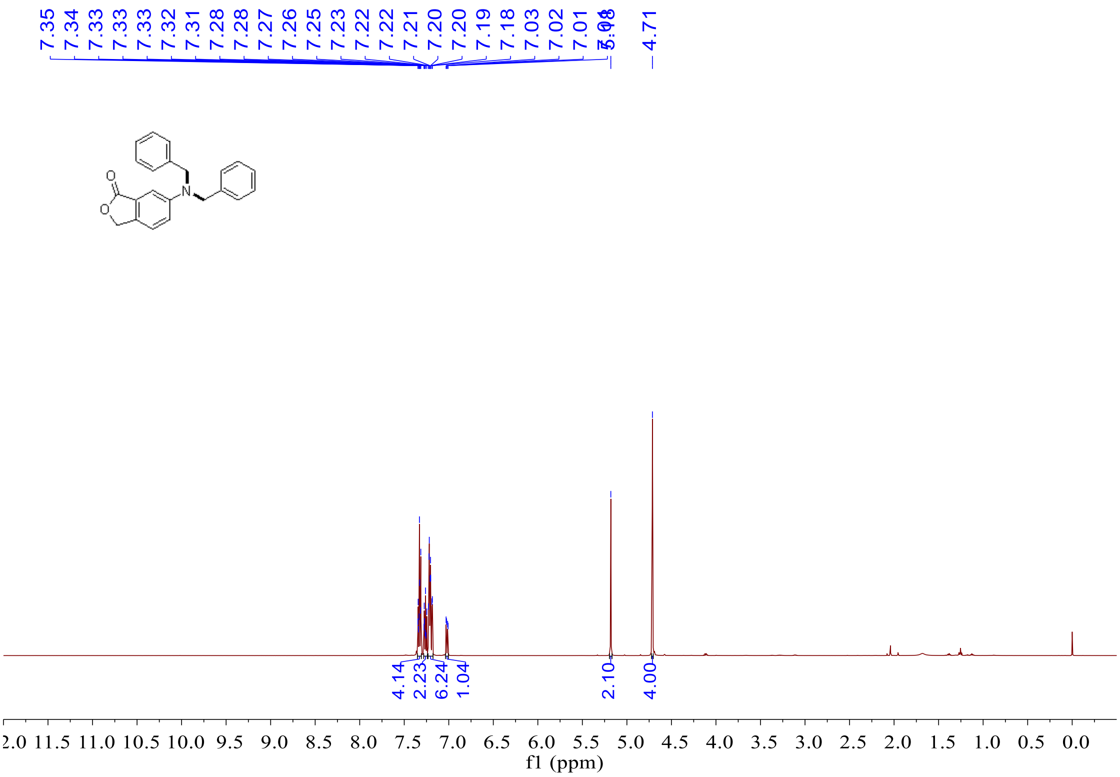

Supplementary Figure 81 ^{1}H NMR spectrum for compound **38**

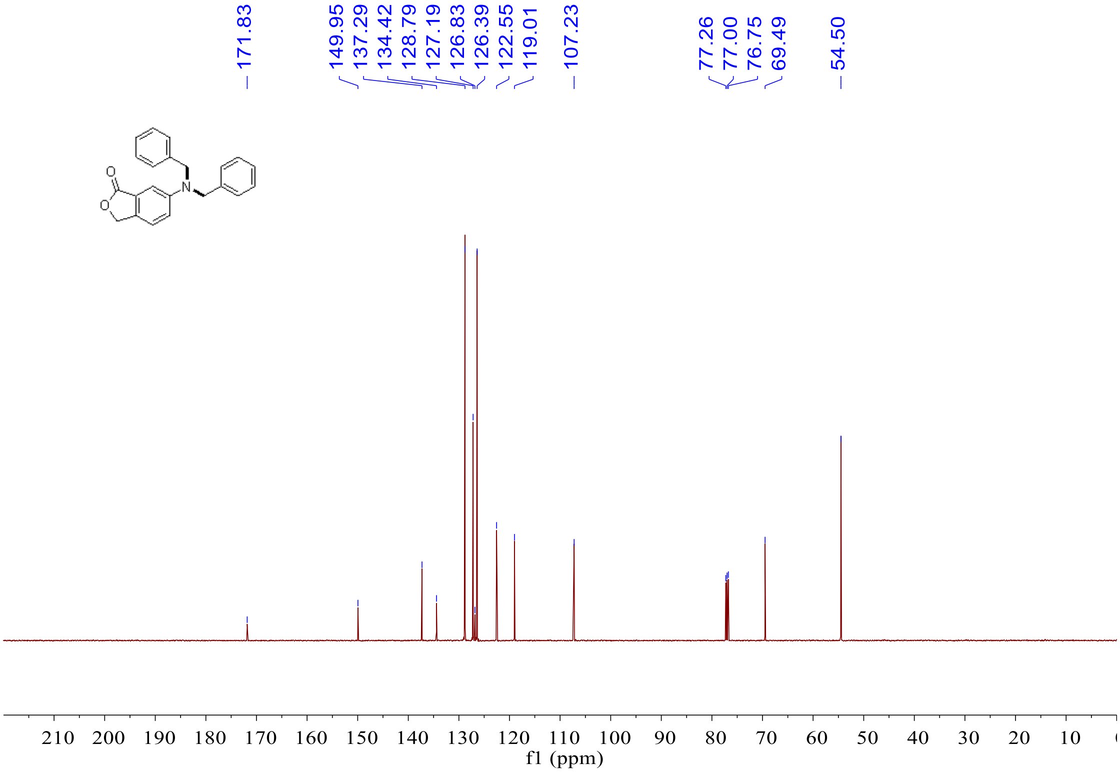

Supplementary Figure 82 ^{13}C NMR spectrum for compound **38**

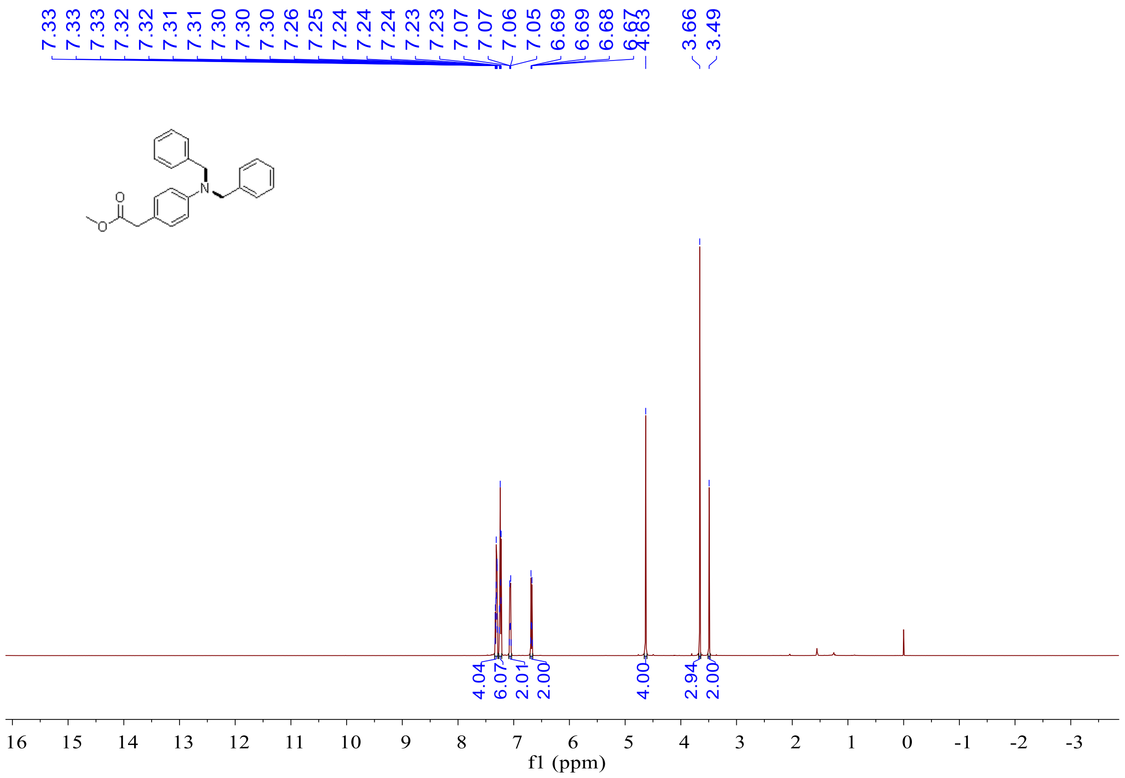

Supplementary Figure 83 ^{1}H NMR spectrum for compound **39**

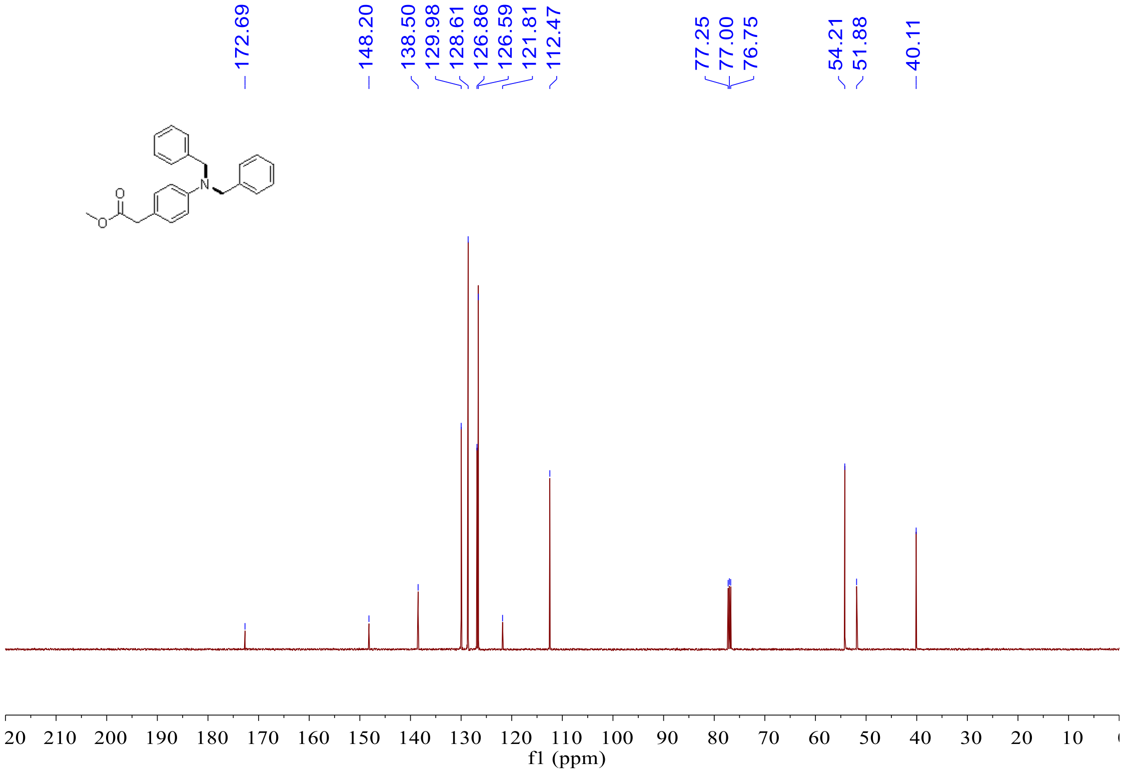

Supplementary Figure 84 ^{13}C NMR spectrum for compound **39**

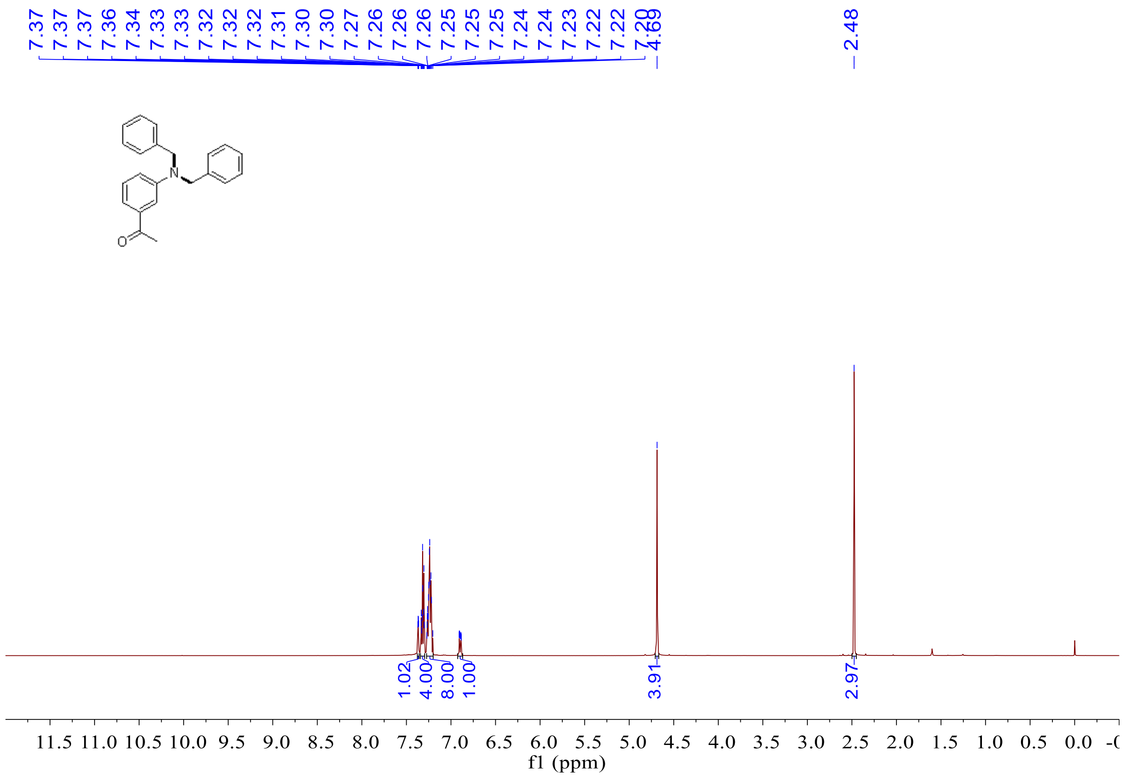

Supplementary Figure 85 ^{1}H NMR spectrum for compound **40**

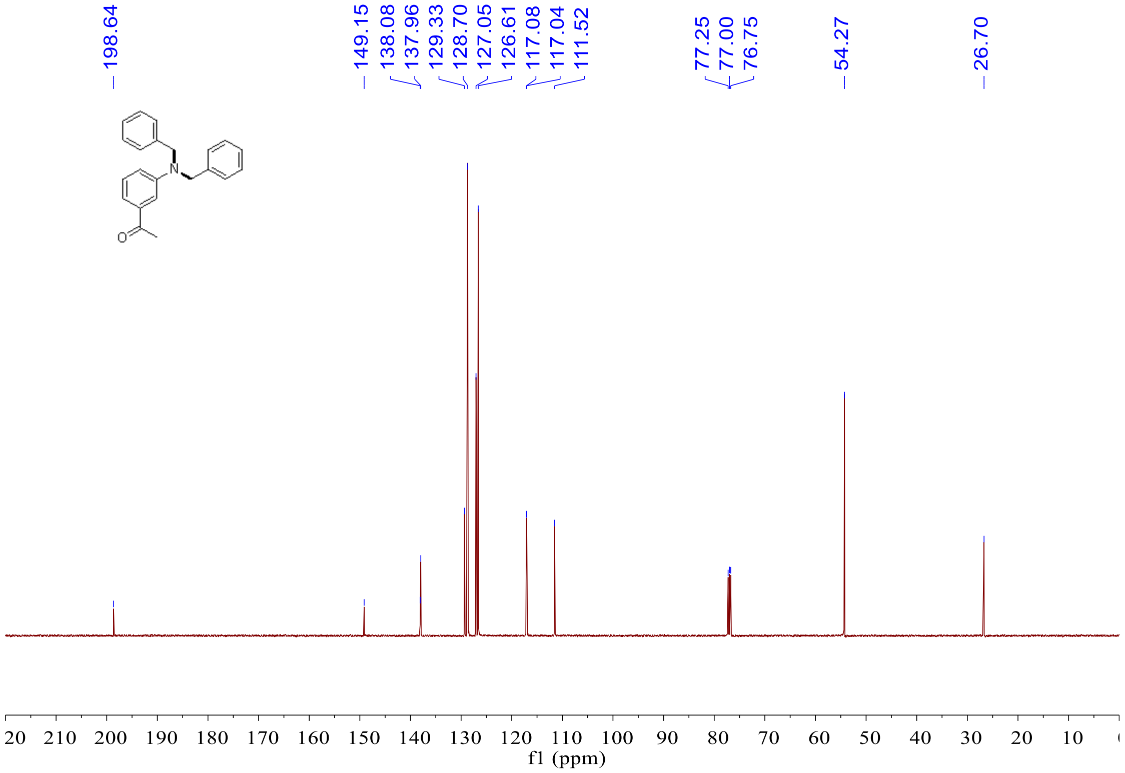

Supplementary Figure 86 ^{13}C NMR spectrum for compound **40**

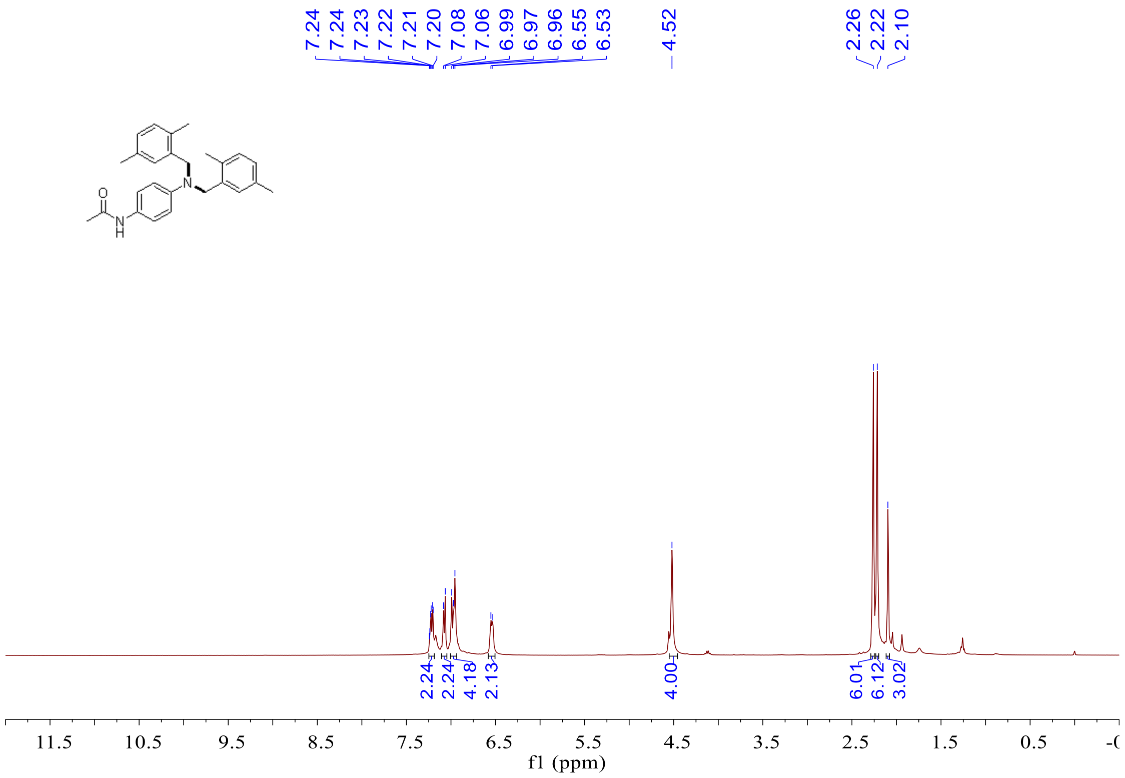

Supplementary Figure 87 ^{1}H NMR spectrum for compound **41**

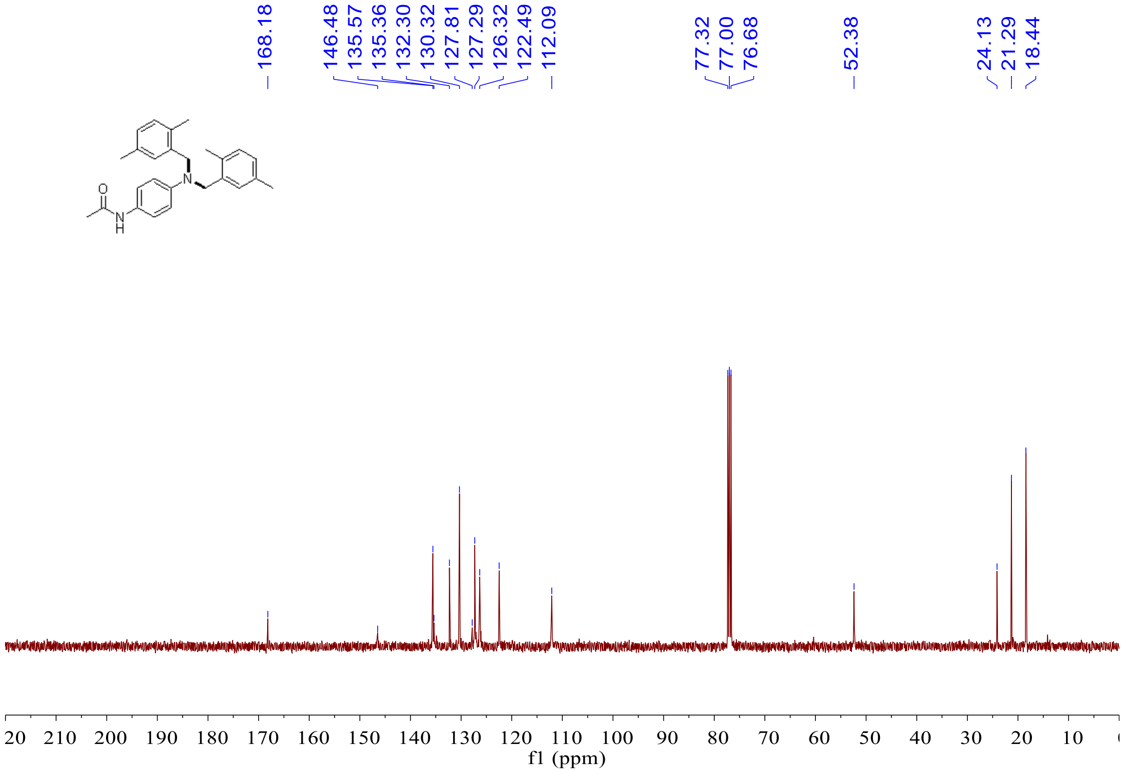

Supplementary Figure 88 ^{13}C NMR spectrum for compound **41**

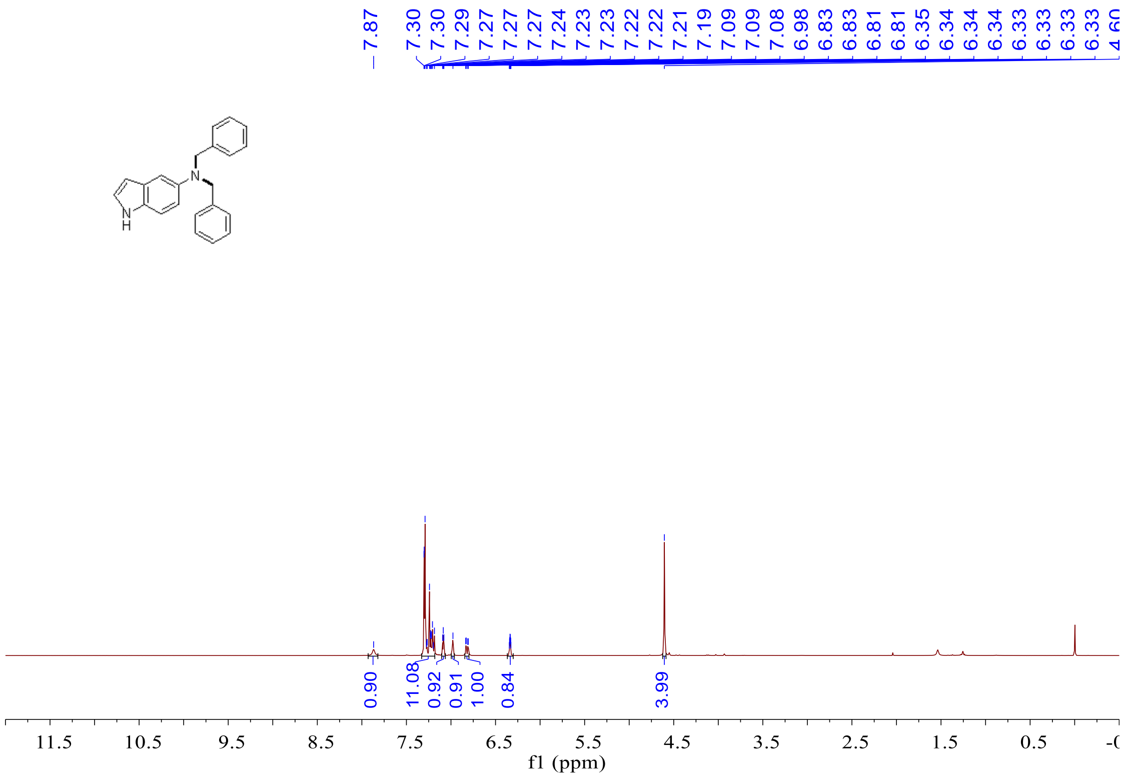

Supplementary Figure 89 ^{1}H NMR spectrum for compound **42**

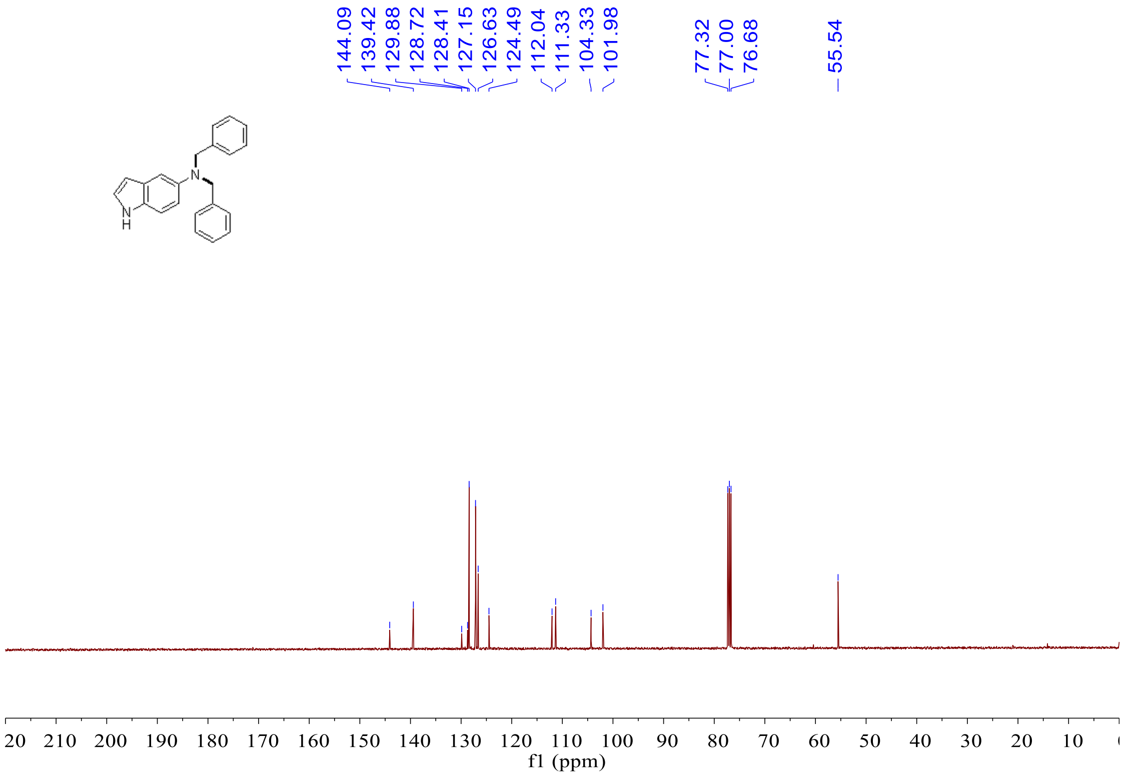

Supplementary Figure 90 ^{13}C NMR spectrum for compound **42**

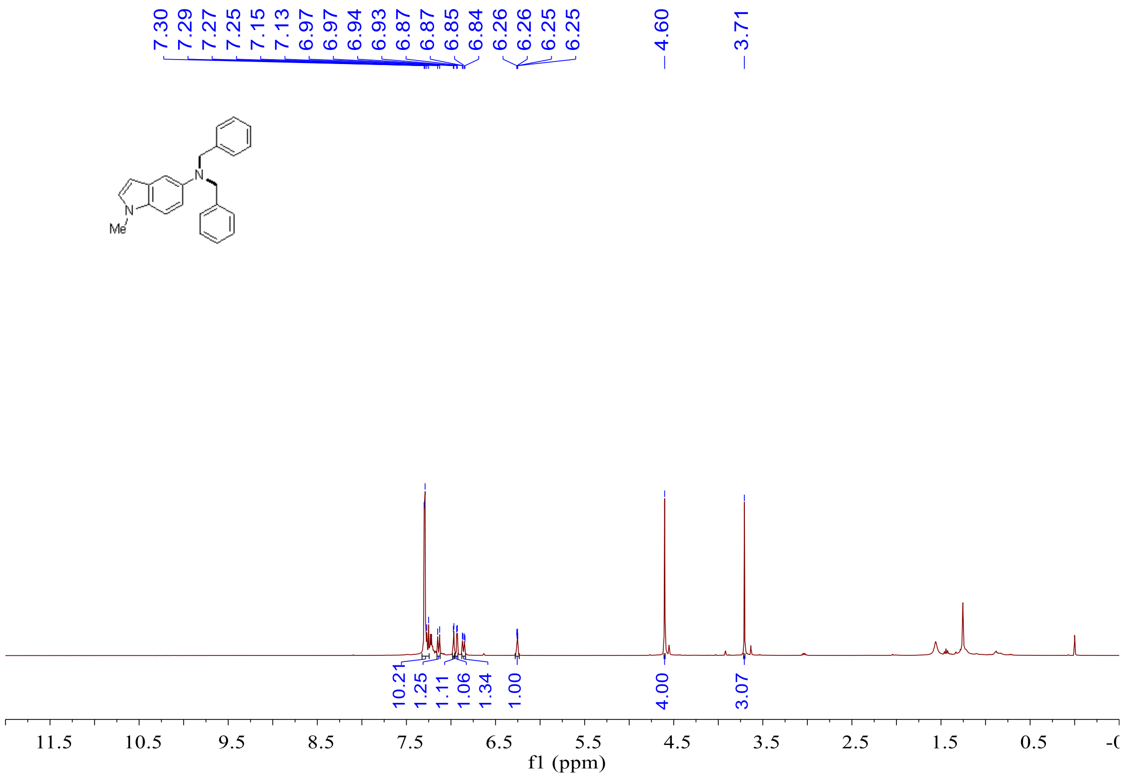

Supplementary Figure 91 ^{1}H NMR spectrum for compound **43**

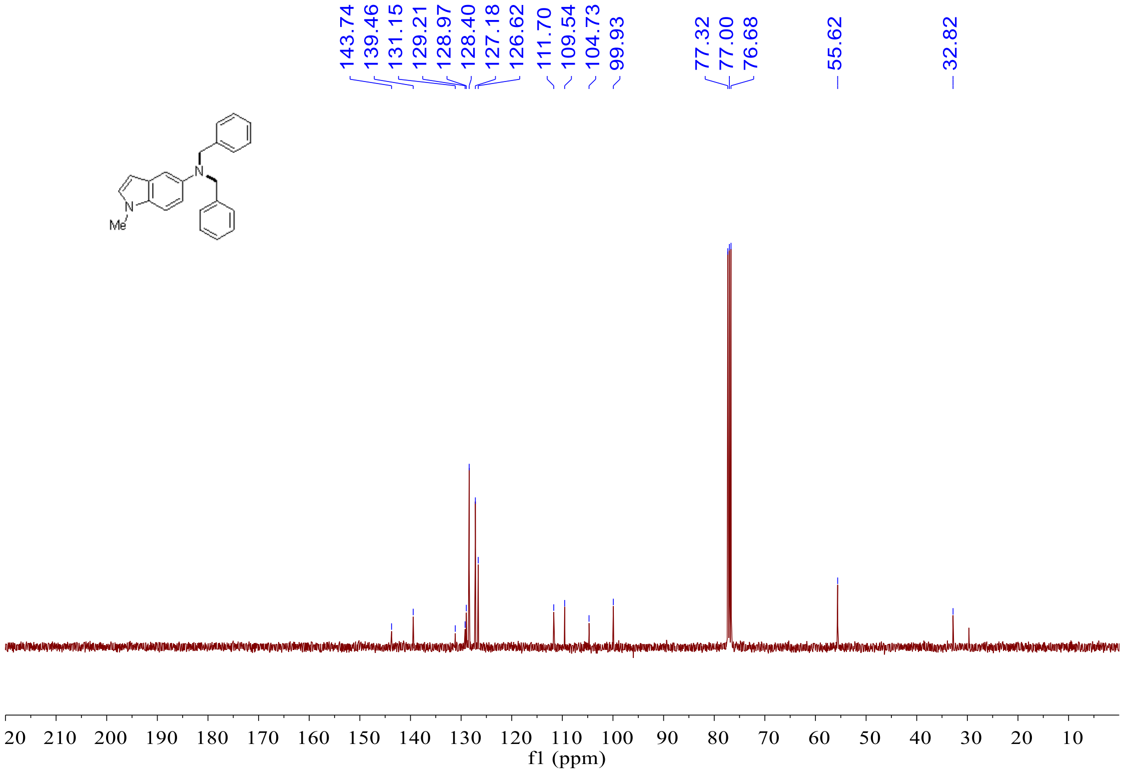

Supplementary Figure 92 ^{13}C NMR spectrum for compound **43**

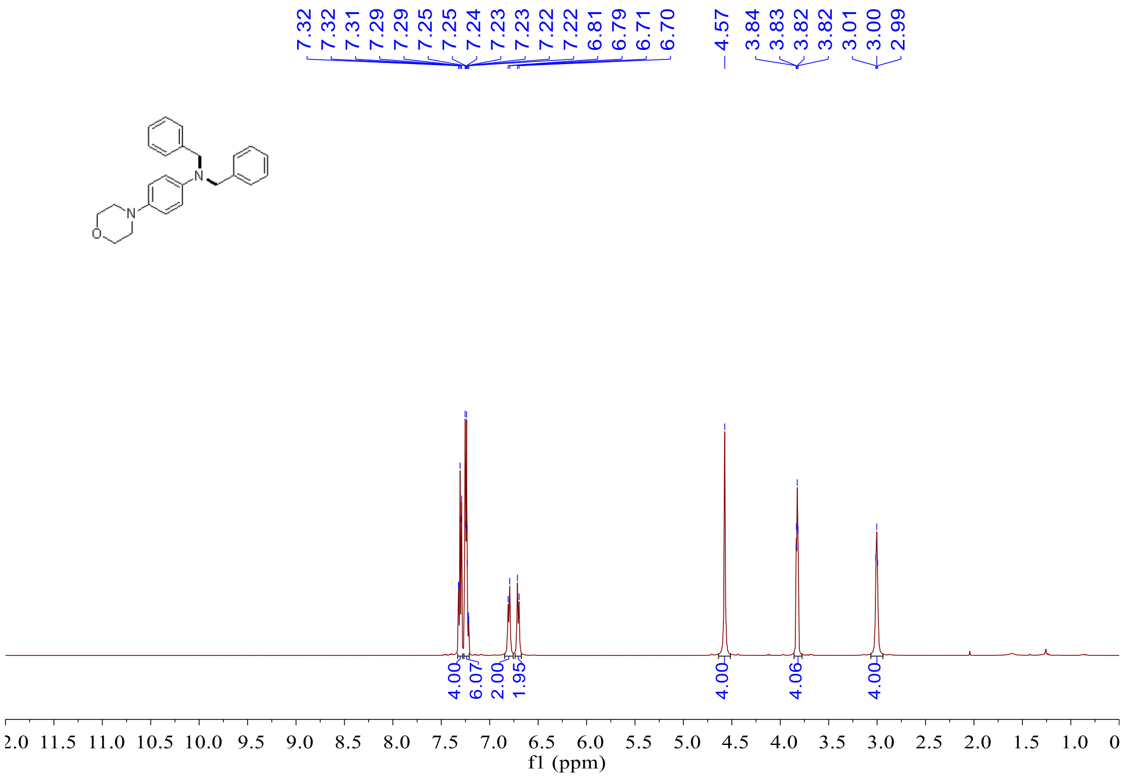

Supplementary Figure 93 ^{1}H NMR spectrum for compound **44**

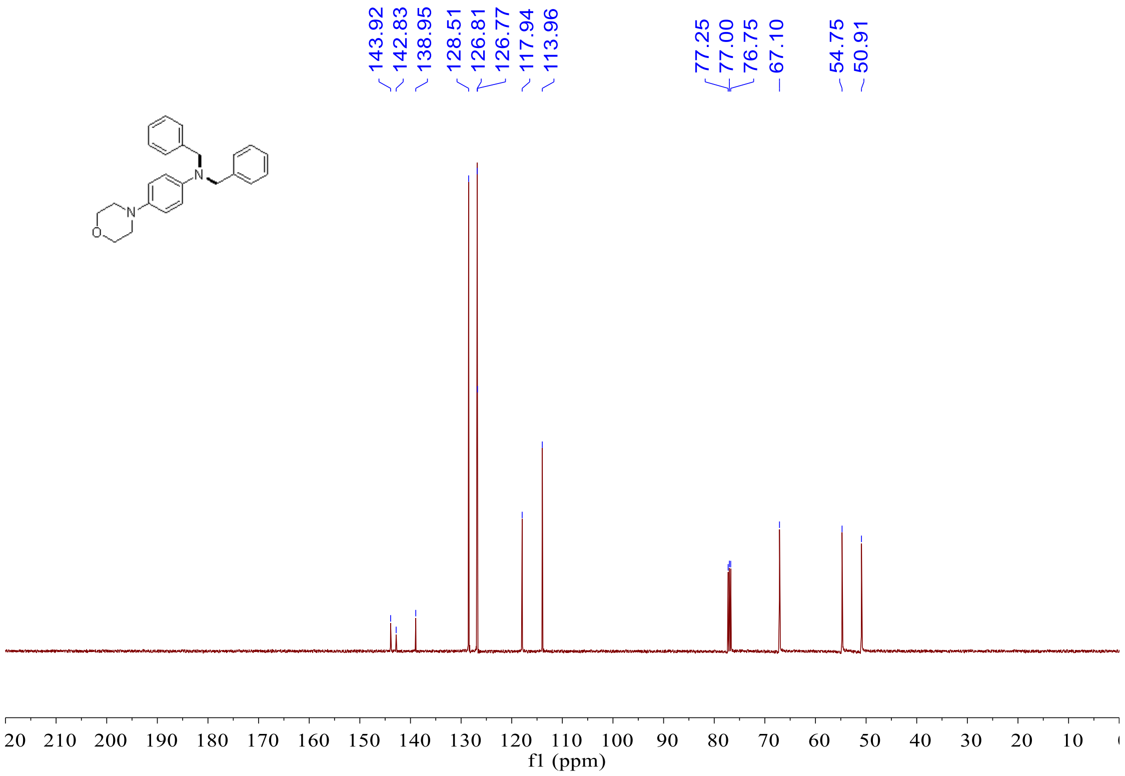

Supplementary Figure 94 ^{13}C NMR spectrum for compound **44**

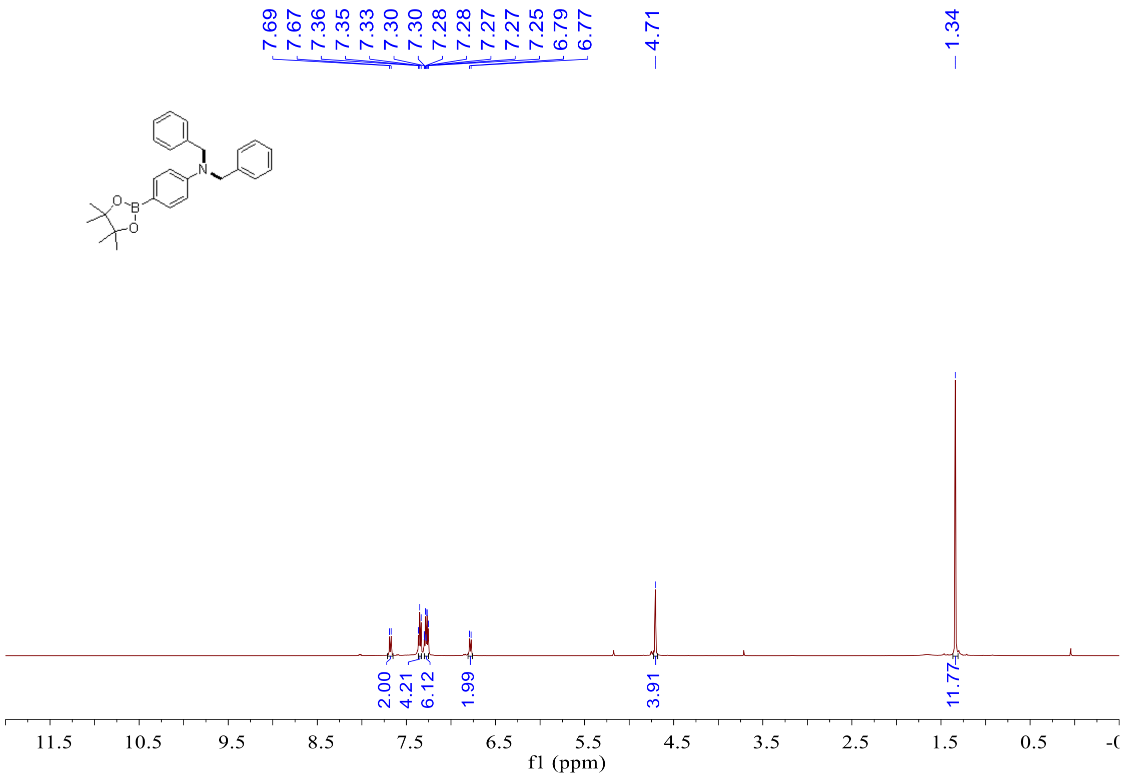

Supplementary Figure 95 ^{1}H NMR spectrum for compound **45**

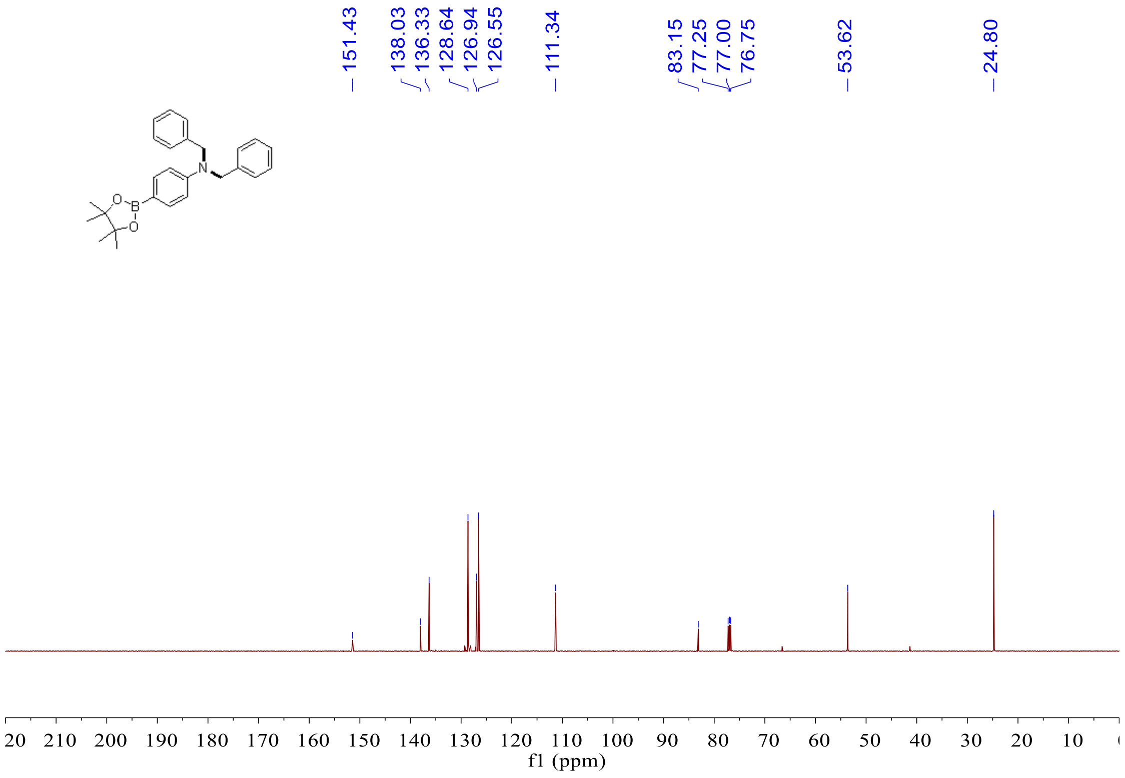

Supplementary Figure 96 ^{13}C NMR spectrum for compound **45**

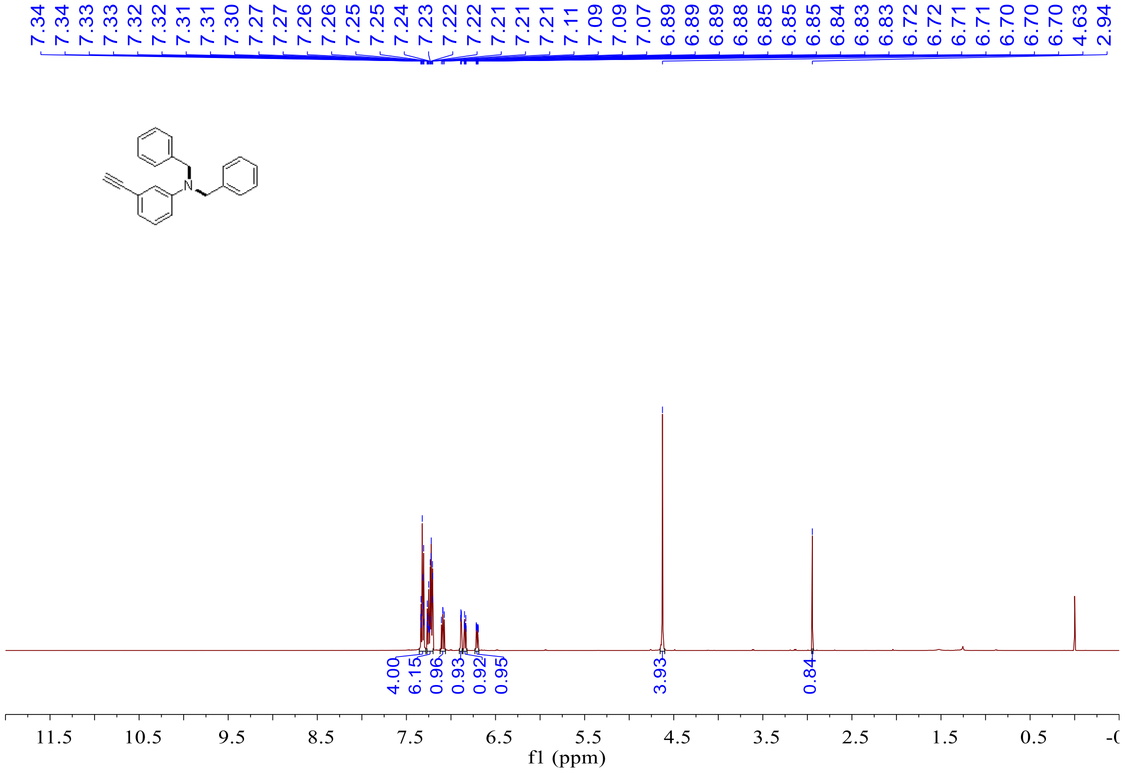

Supplementary Figure 97 ^{1}H NMR spectrum for compound **46**

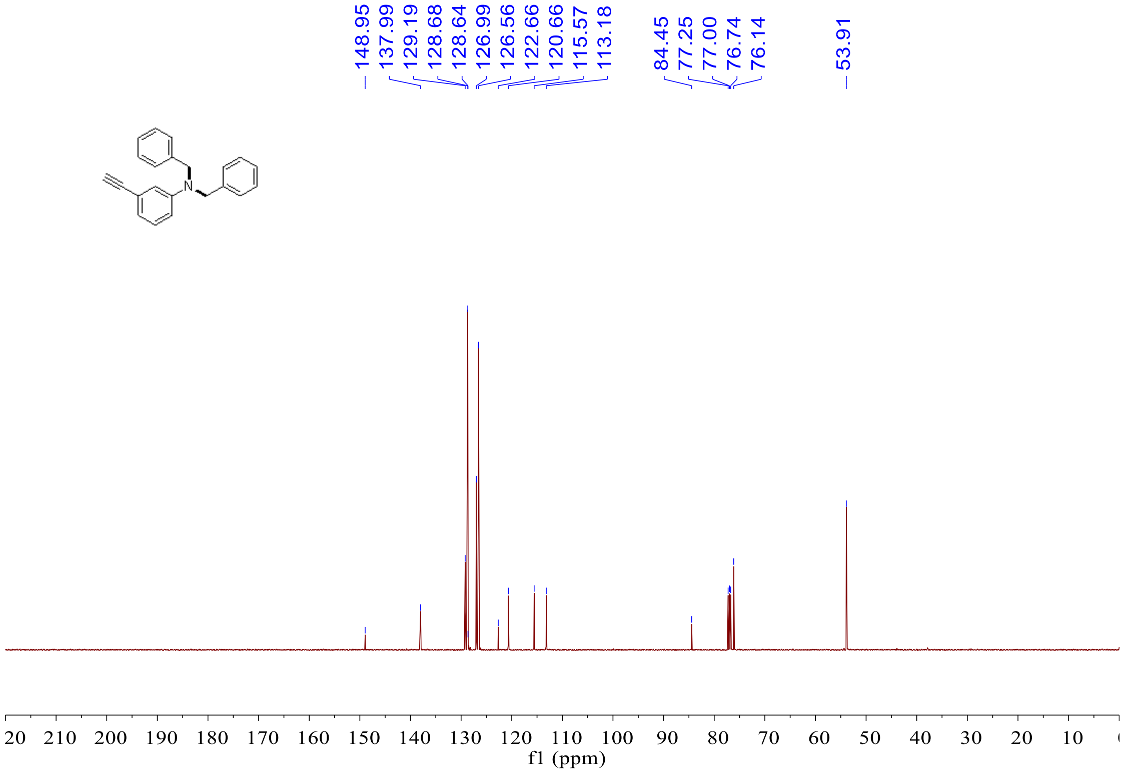

Supplementary Figure 98 ^{13}C NMR spectrum for compound **46**

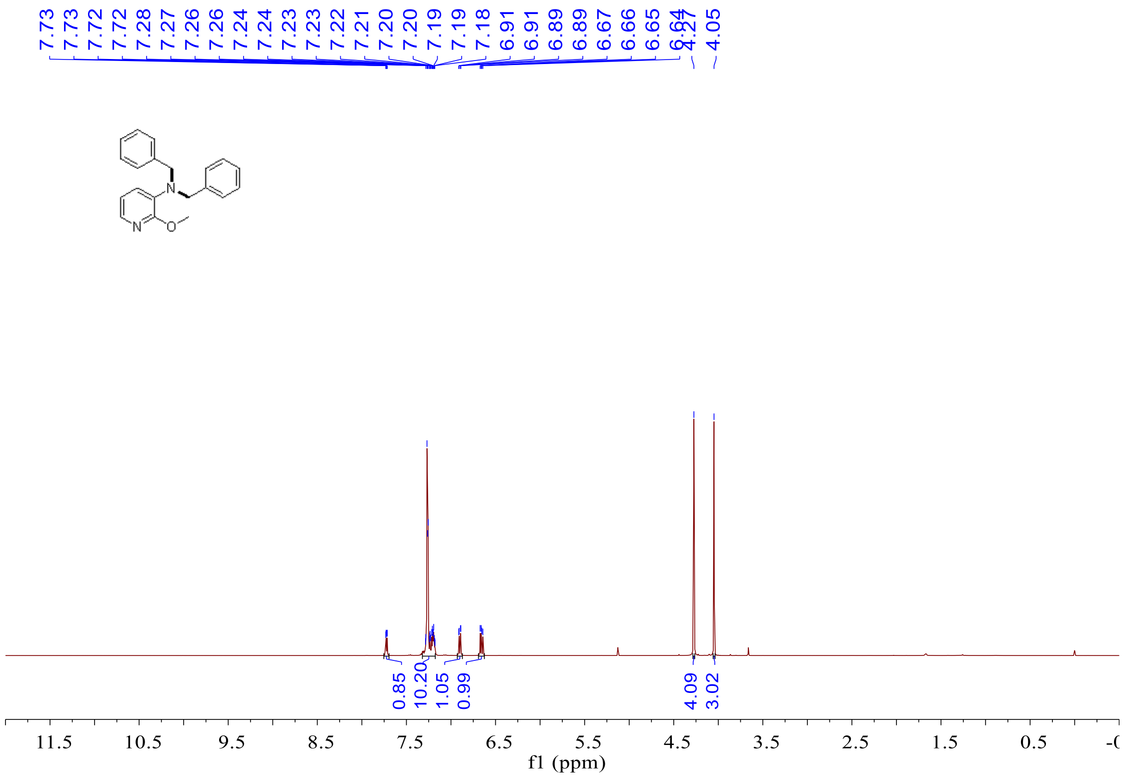

Supplementary Figure 99 ^{1}H NMR spectrum for compound **47**

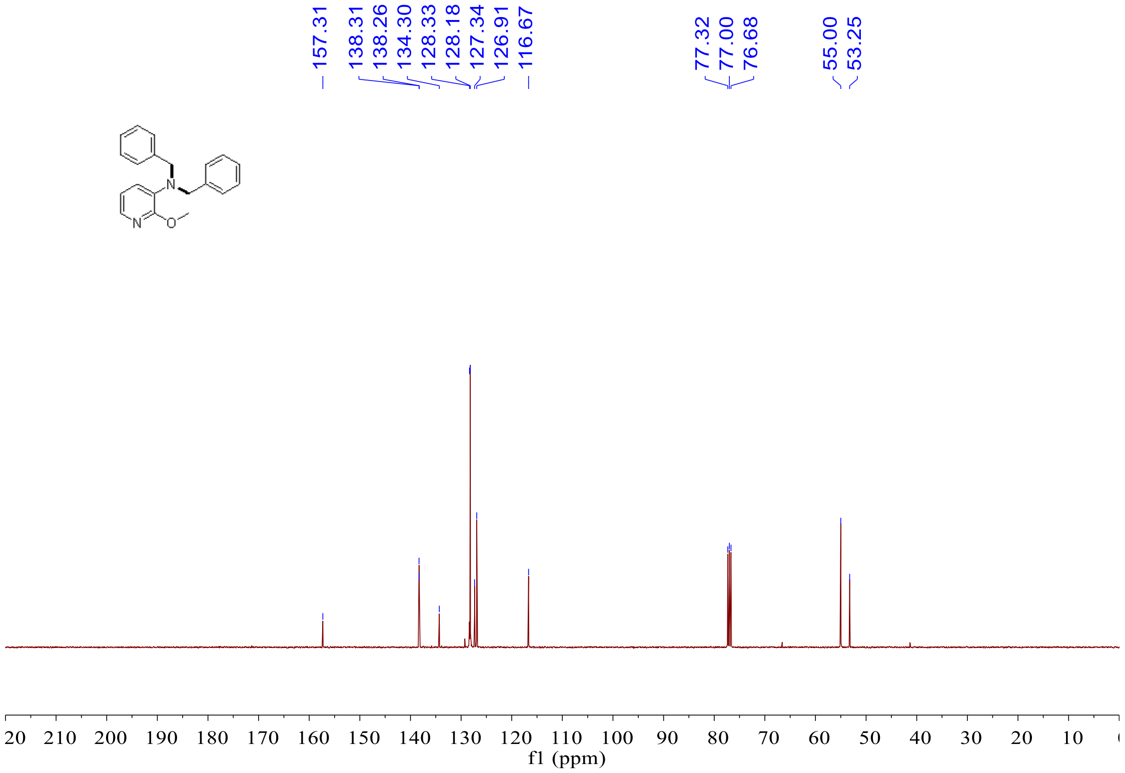

Supplementary Figure 100 ^{13}C NMR spectrum for compound **47**

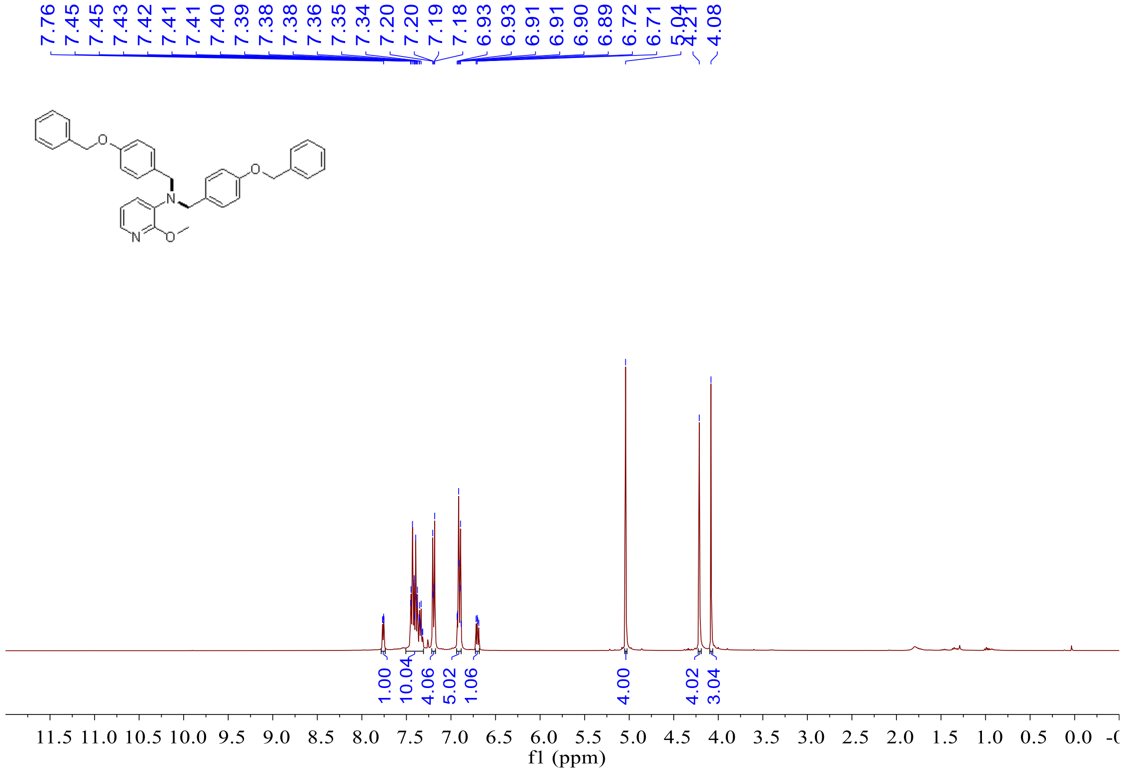

Supplementary Figure 101 ^{1}H NMR spectrum for compound **48**

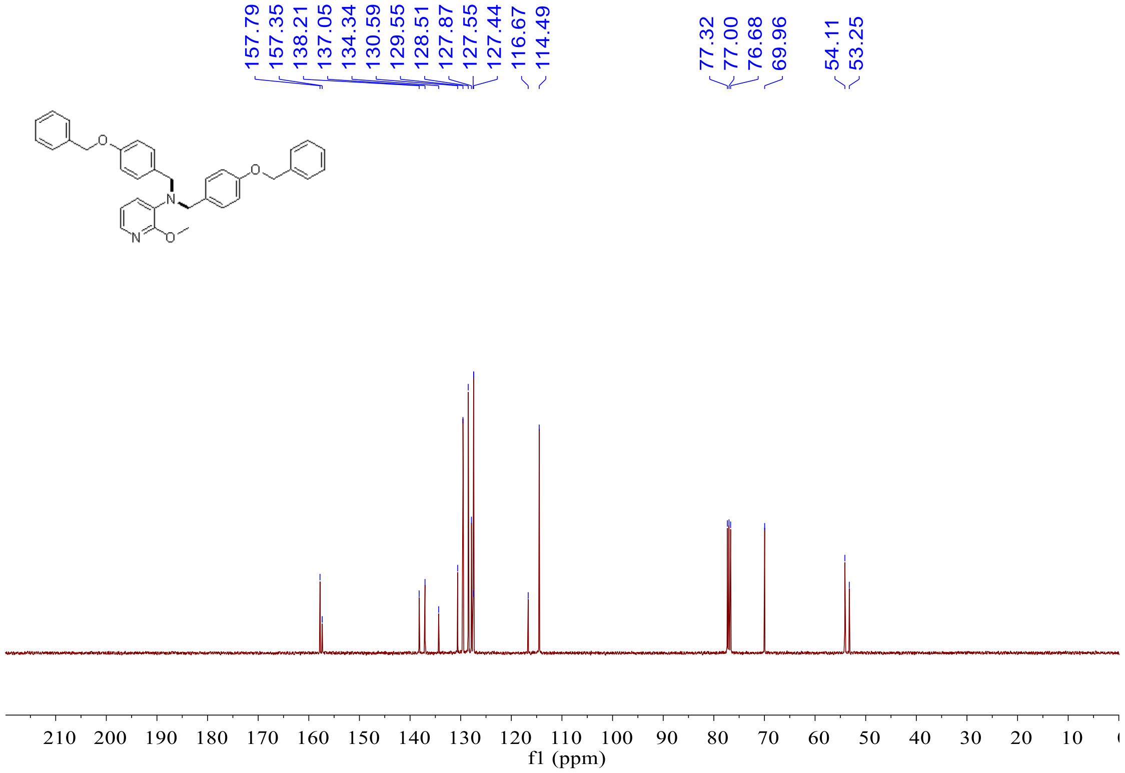

Supplementary Figure 102 ^{13}C NMR spectrum for compound **48**

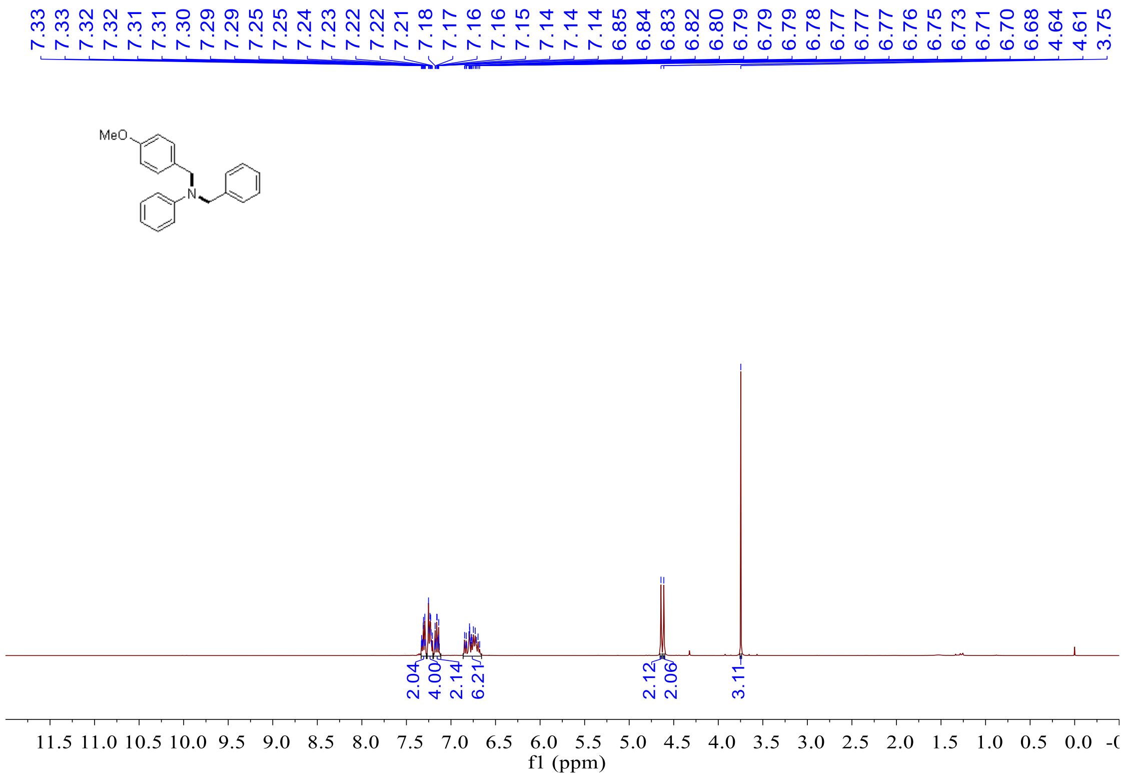

Supplementary Figure 103 ^{1}H NMR spectrum for compound **49**

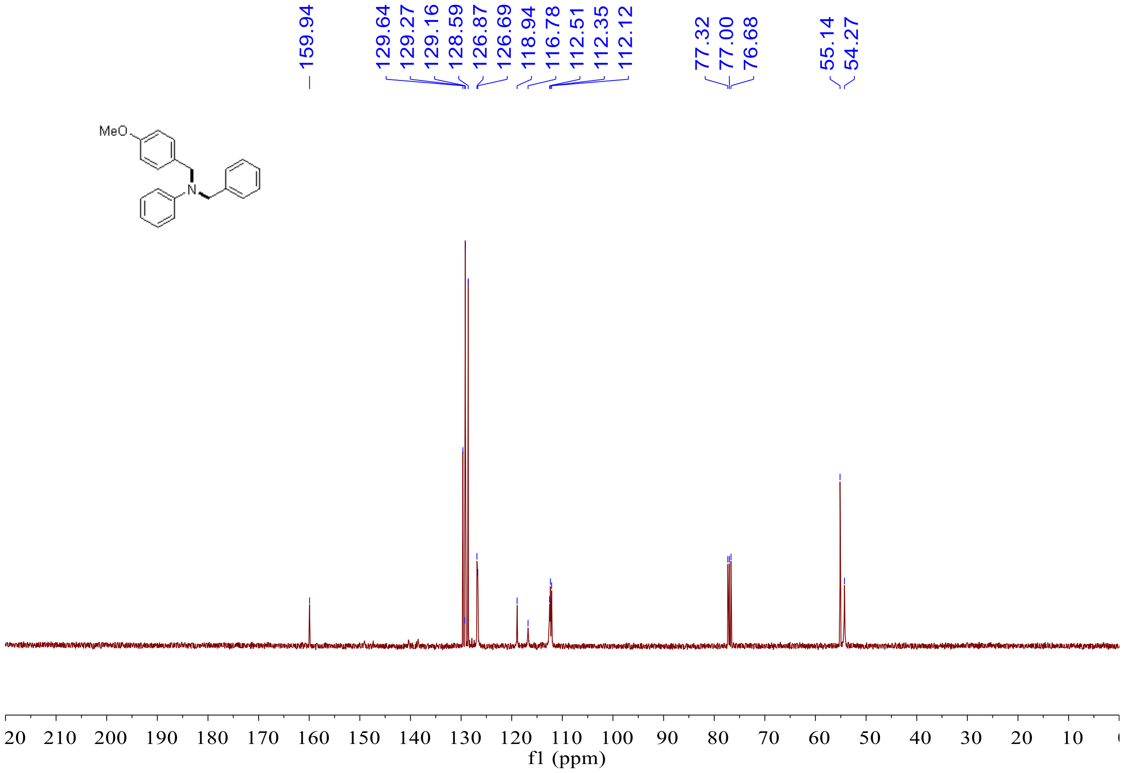

Supplementary Figure 104 ^{13}C NMR spectrum for compound **49**

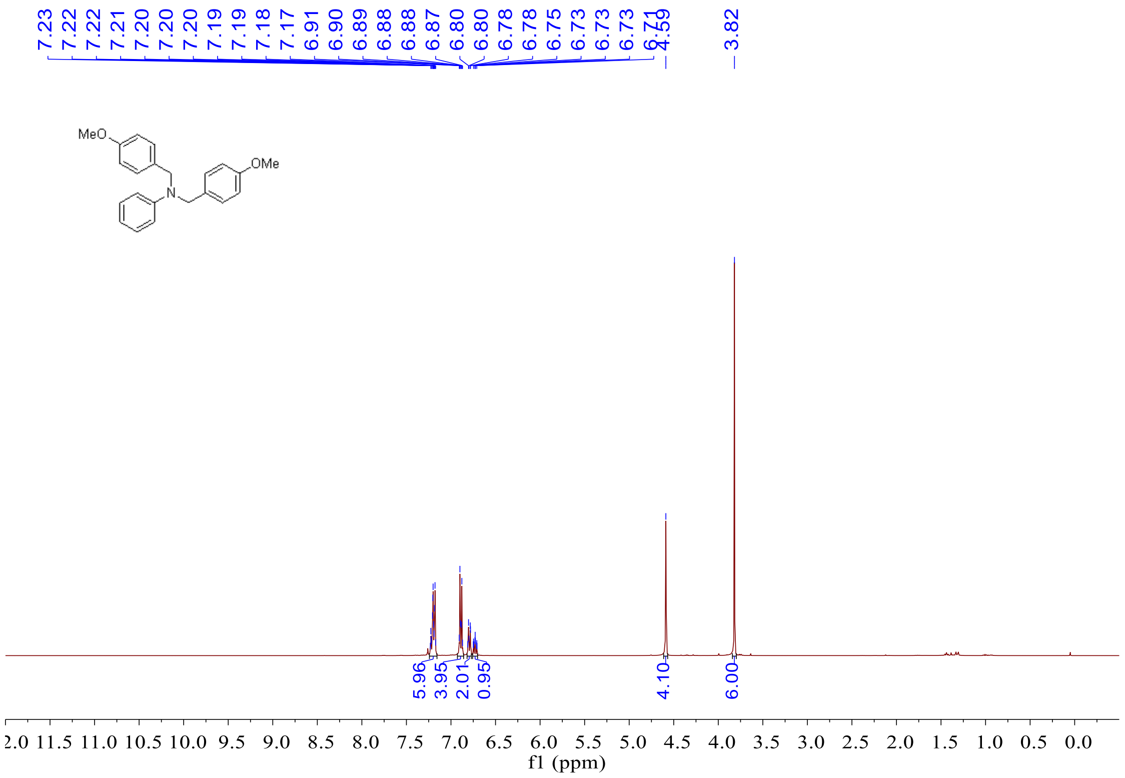

Supplementary Figure 105 ^{1}H NMR spectrum for compound **49a**

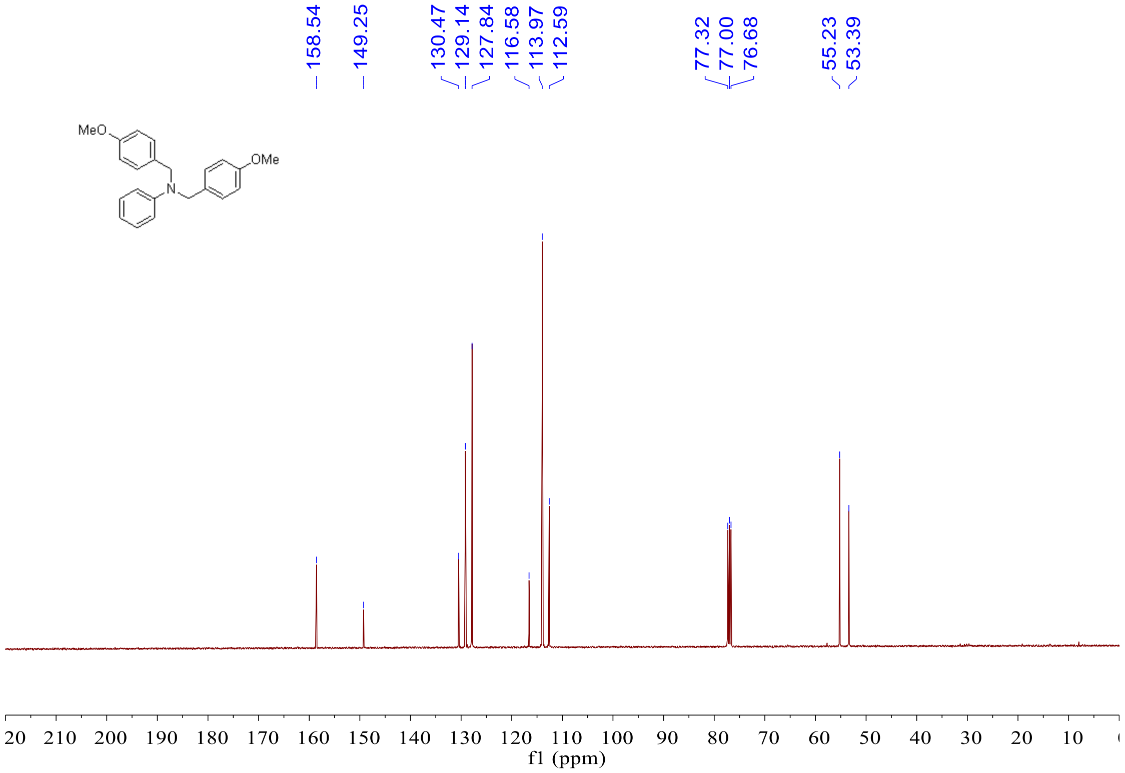

Supplementary Figure 106 ^{13}C NMR spectrum for compound **49a**

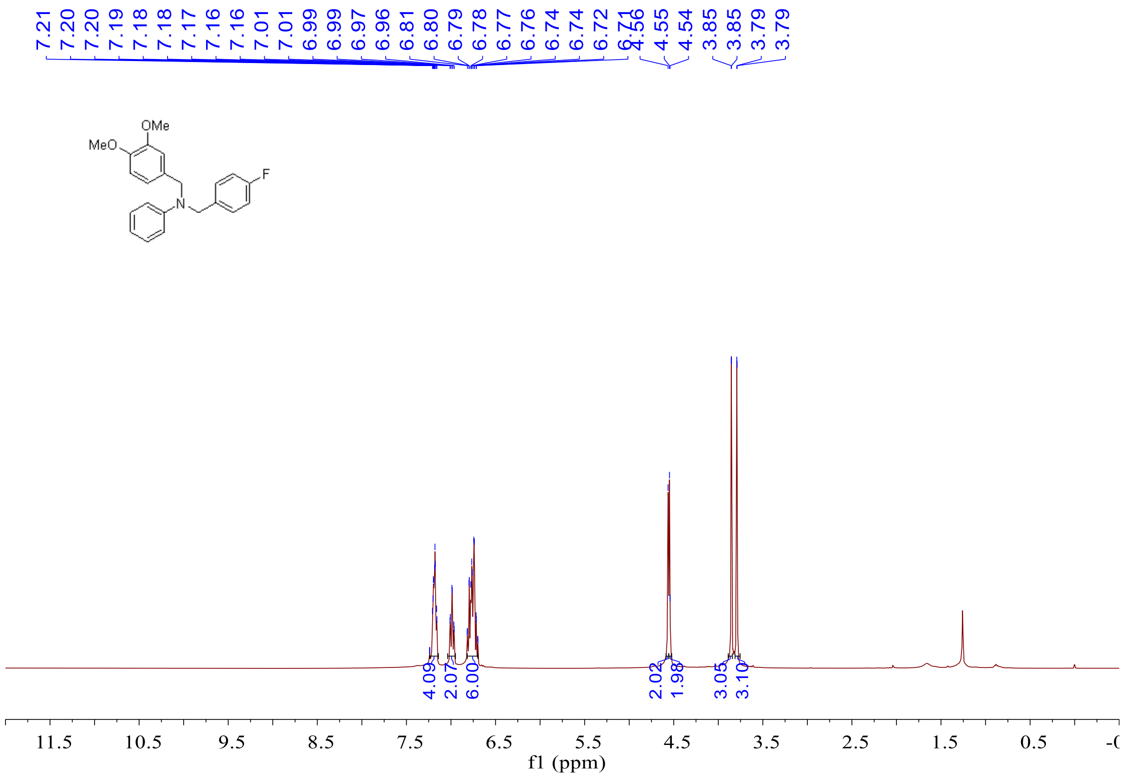

Supplementary Figure 107 ^{1}H NMR spectrum for compound **50**

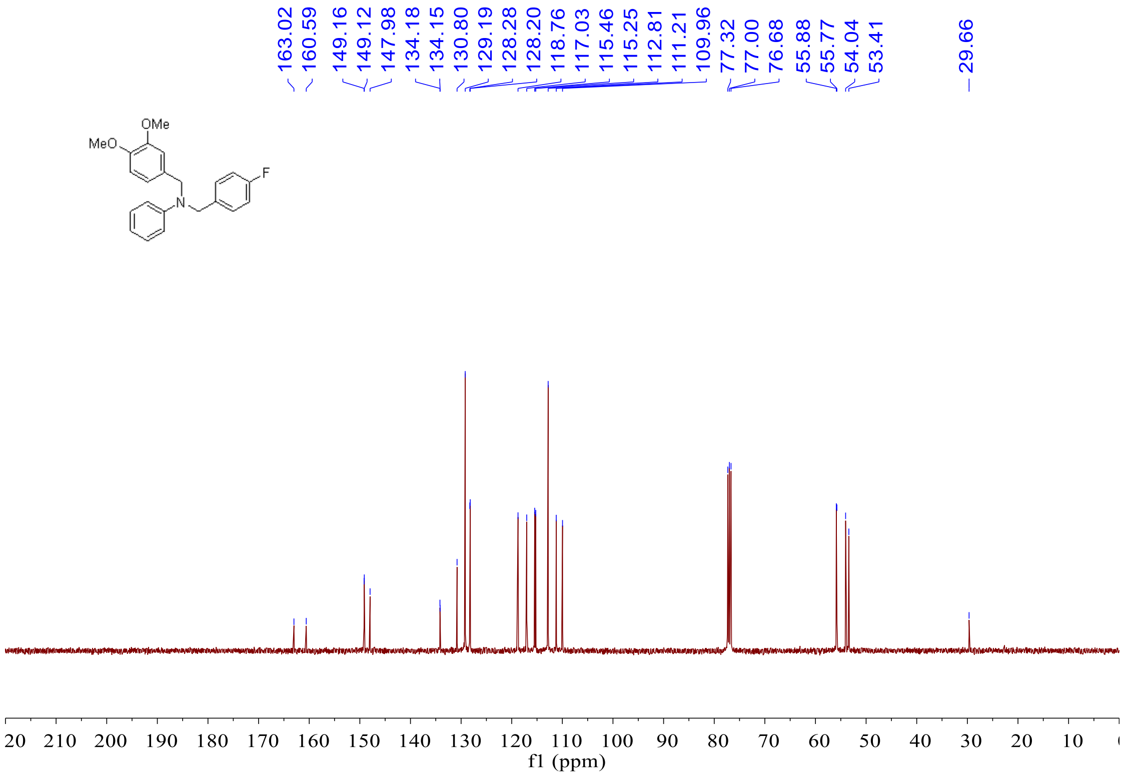

Supplementary Figure 108 ^{13}C NMR spectrum for compound **50**

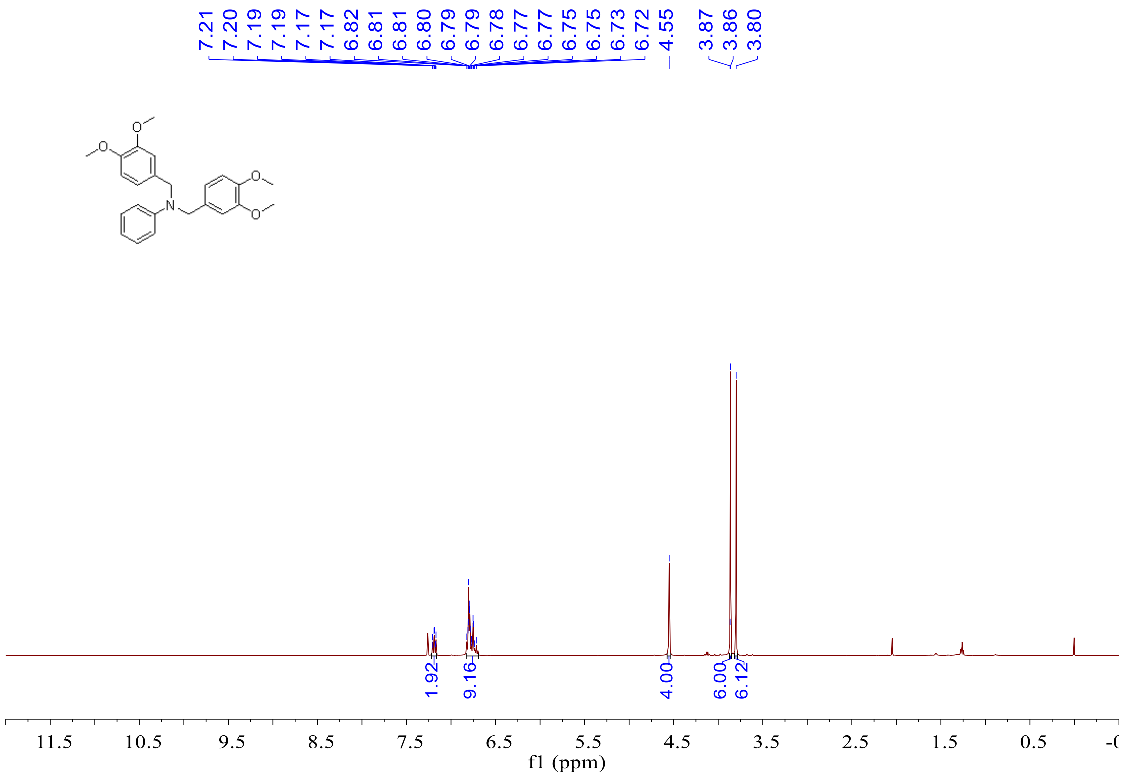

Supplementary Figure 109 ^{1}H NMR spectrum for compound **50a**

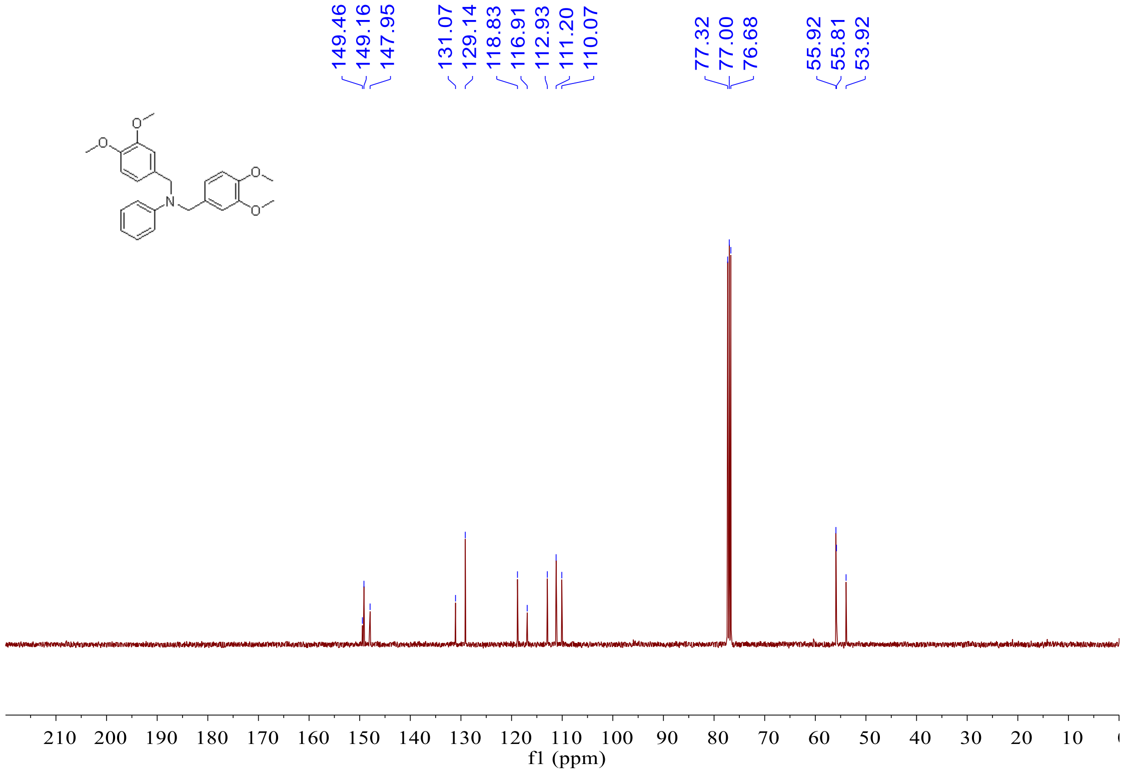

Supplementary Figure 110 ^{13}C NMR spectrum for compound **50a**

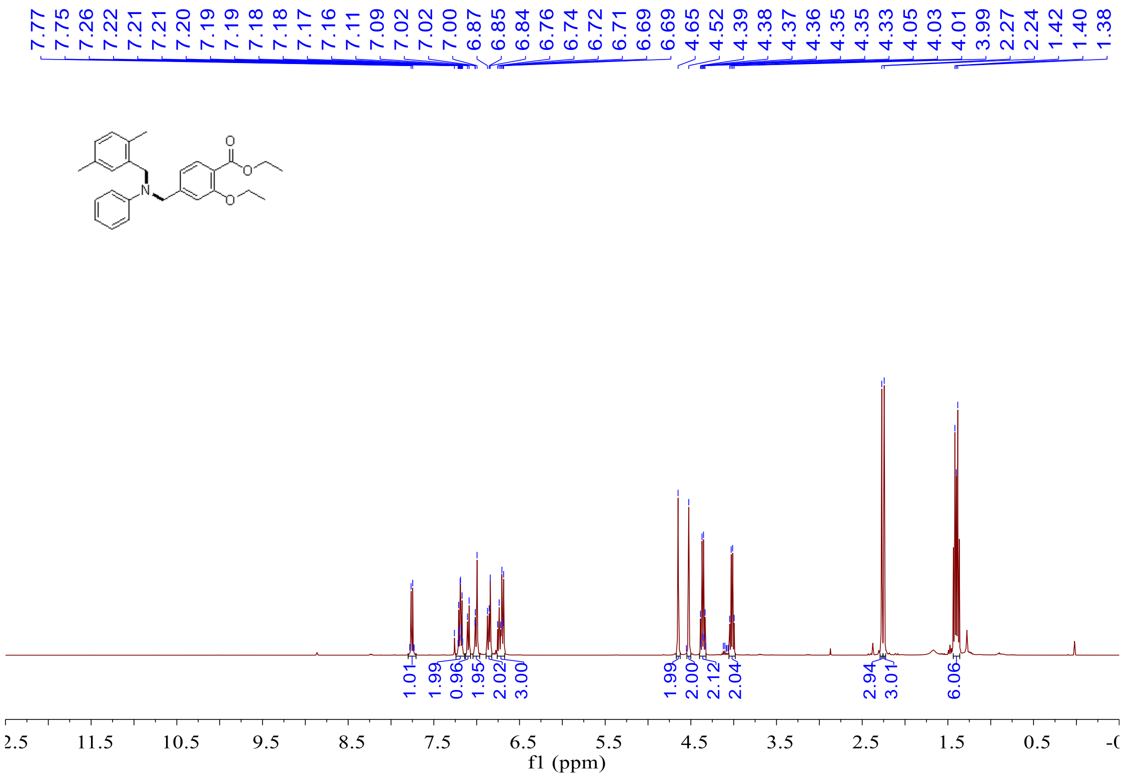

Supplementary Figure 111 ^{1}H NMR spectrum for compound **51**

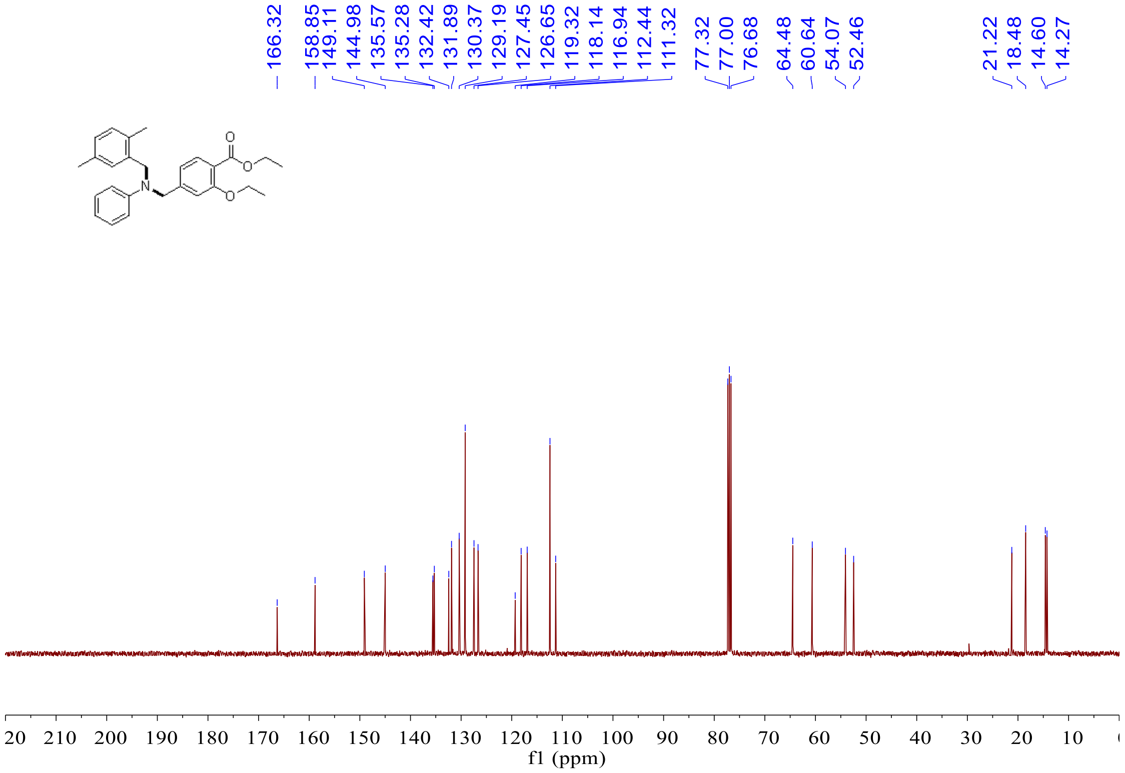

Supplementary Figure 112 ^{13}C NMR spectrum for compound **51**

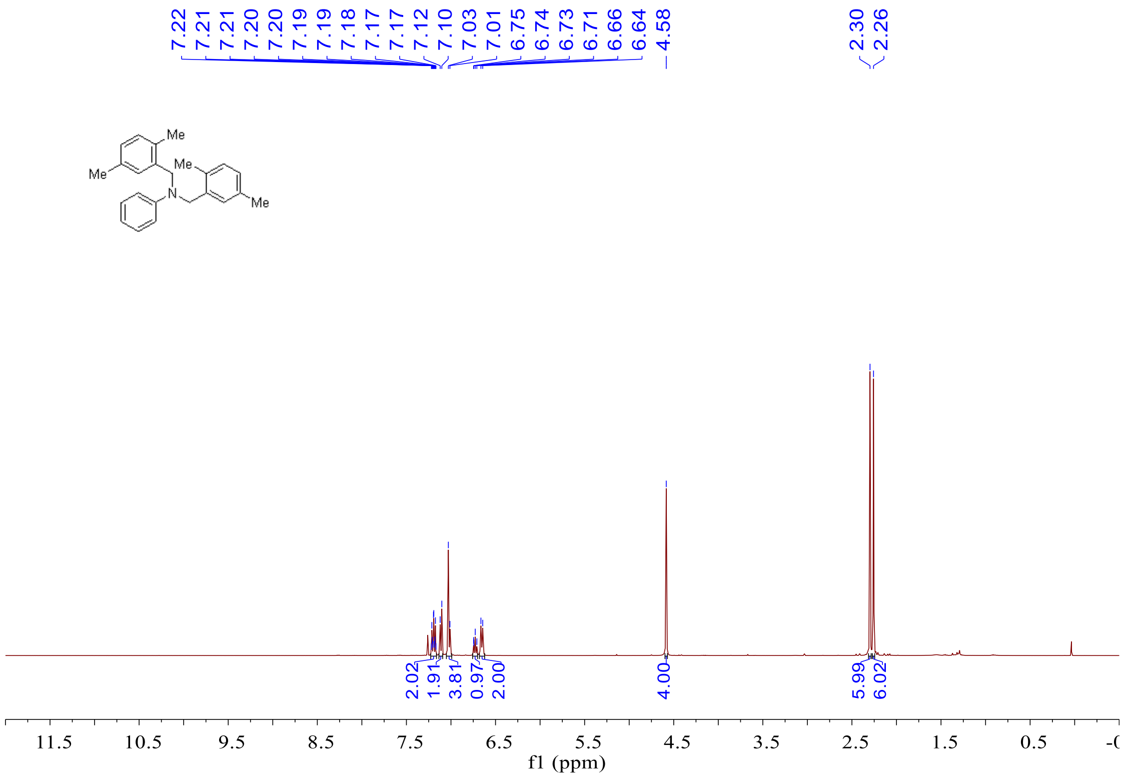

Supplementary Figure 113 ^{1}H NMR spectrum for compound **51a**

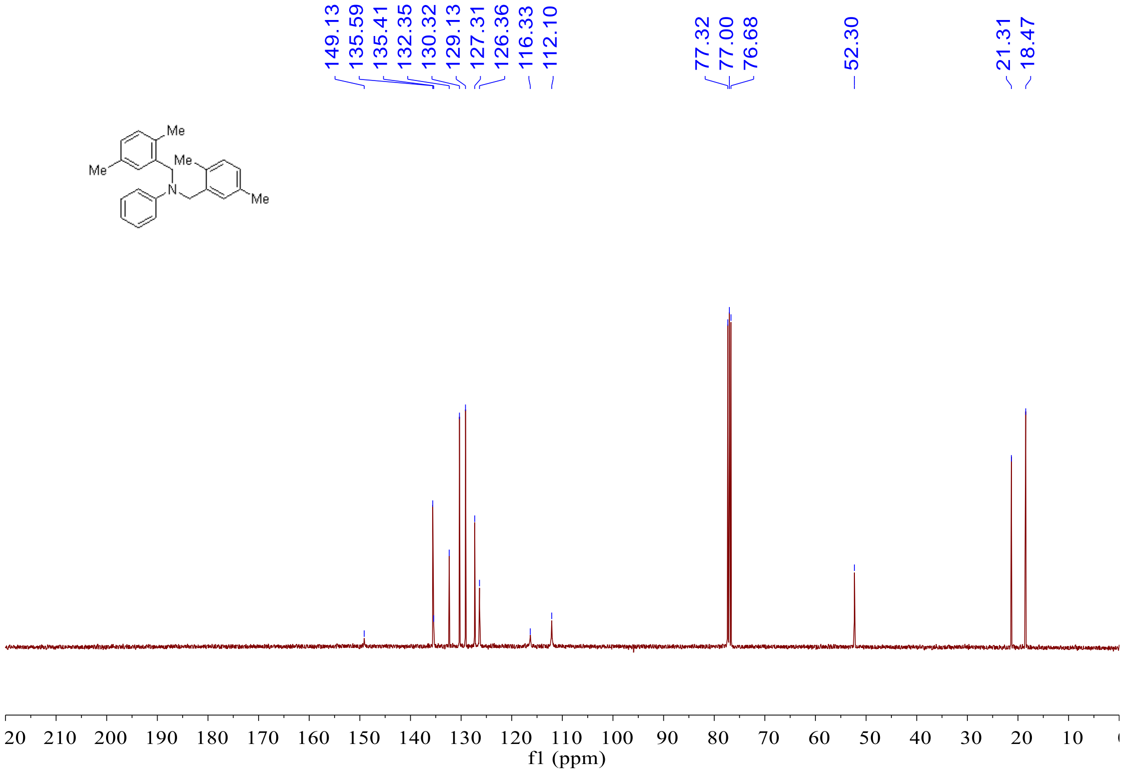

Supplementary Figure 114 ^{13}C NMR spectrum for compound **51a**

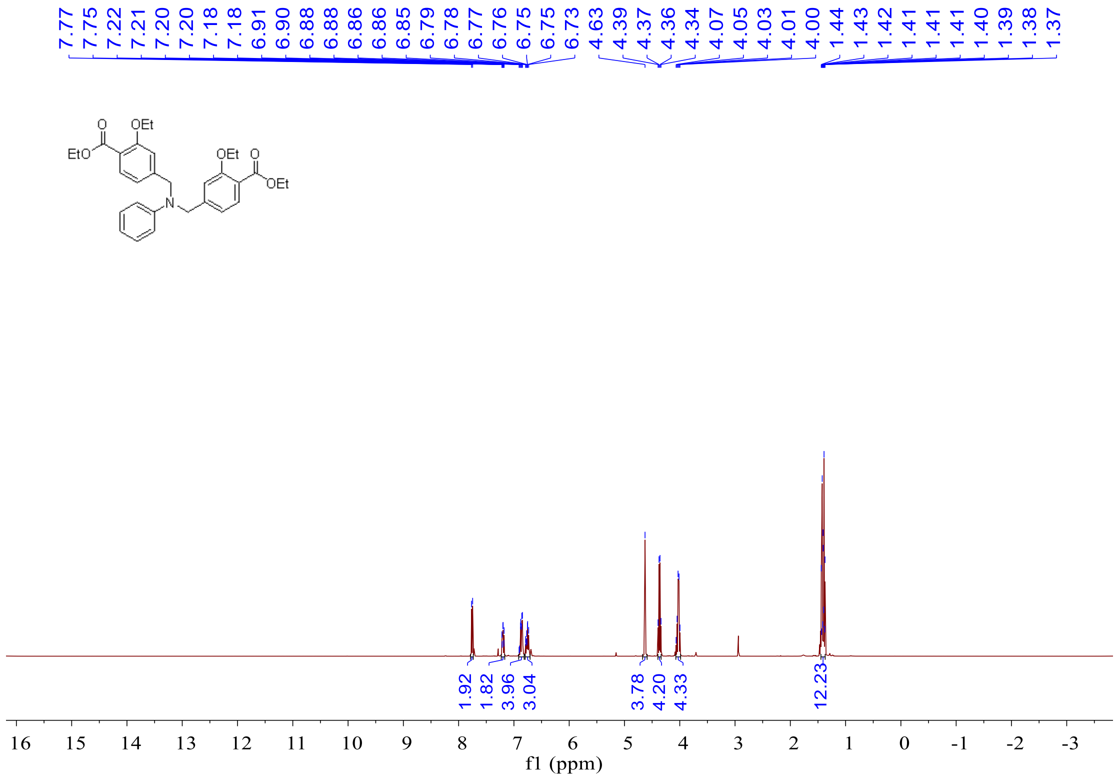

Supplementary Figure 115 ^{1}H NMR spectrum for compound **51b**

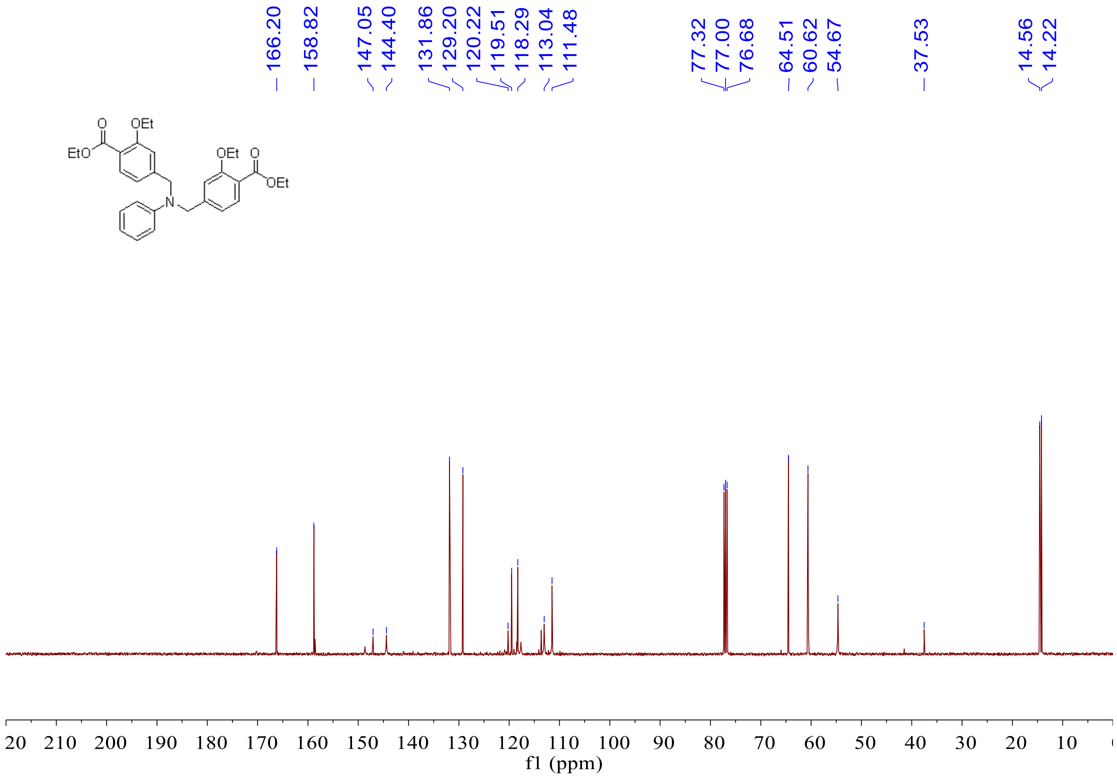

Supplementary Figure 116 ^{13}C NMR spectrum for compound **51b**

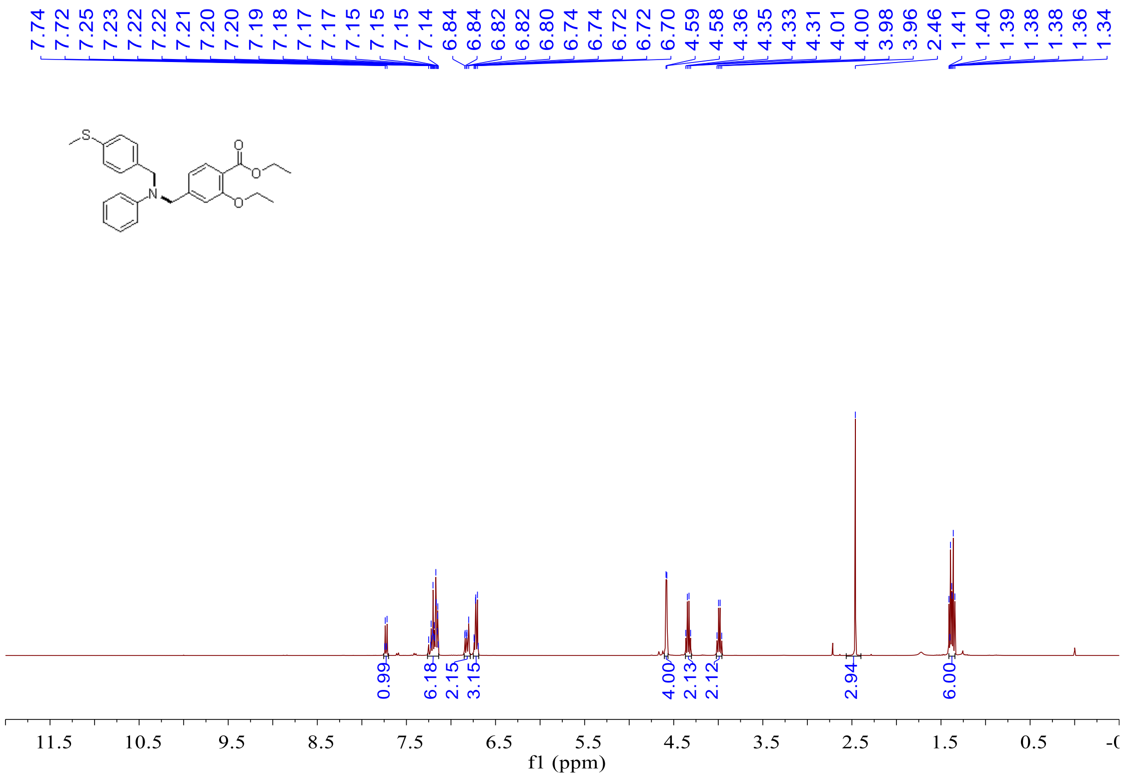

Supplementary Figure 117 ^{1}H NMR spectrum for compound **52**

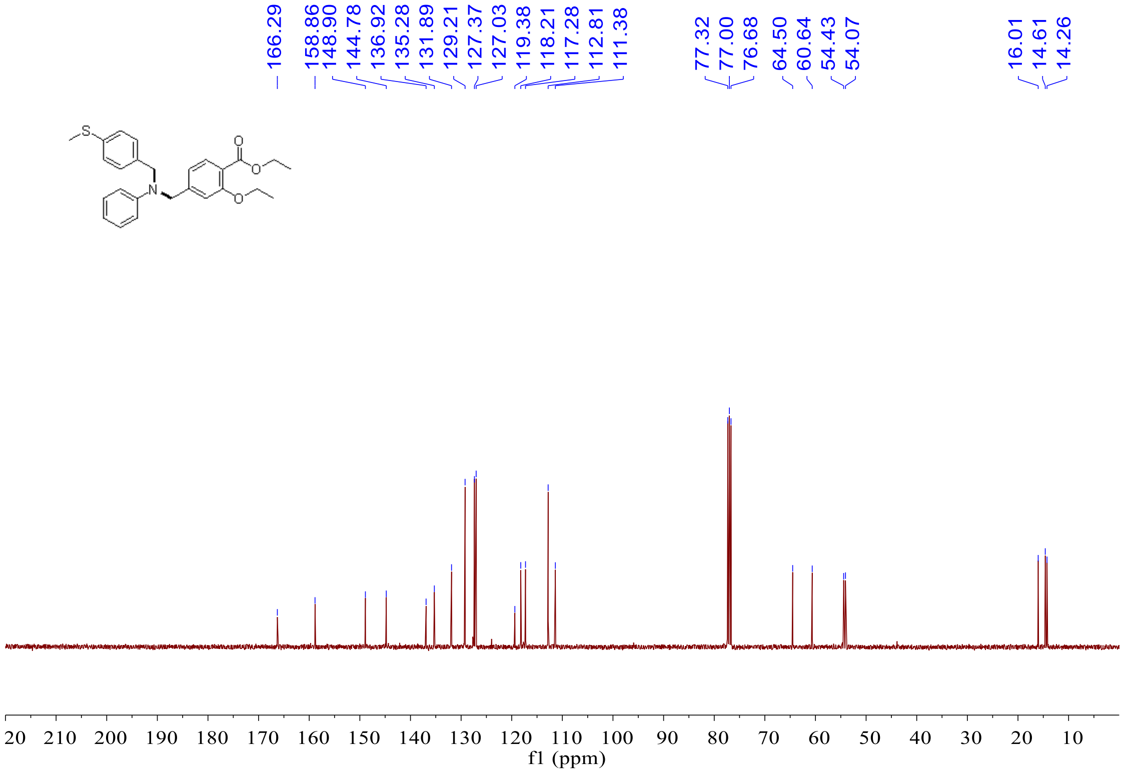

Supplementary Figure 118 ^{13}C NMR spectrum for compound **52**

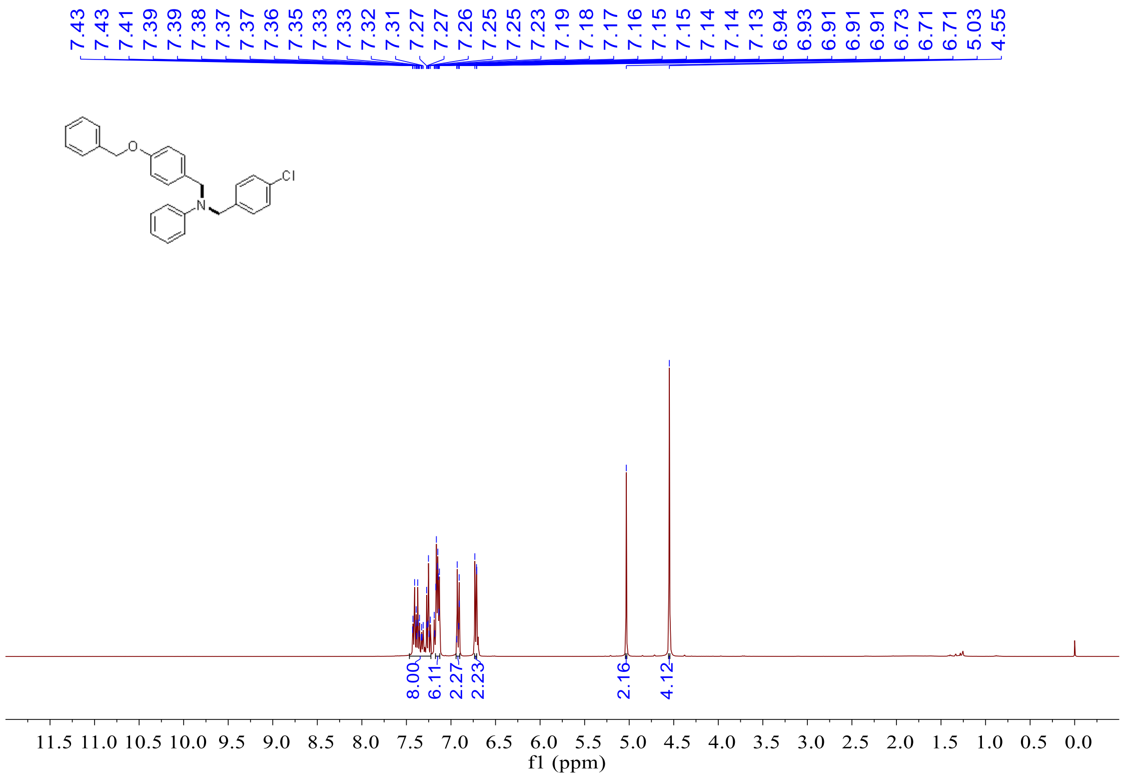

Supplementary Figure 119 ^{1}H NMR spectrum for compound **53**

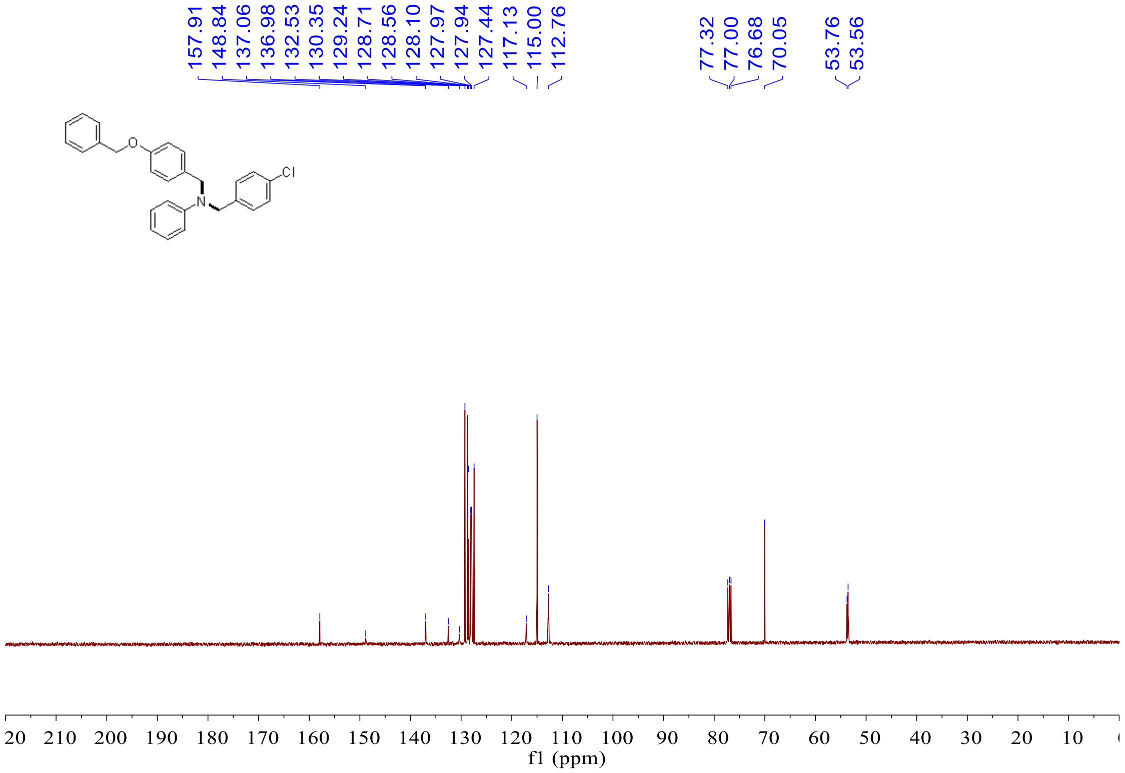

Supplementary Figure 120 ^{13}C NMR spectrum for compound **53**

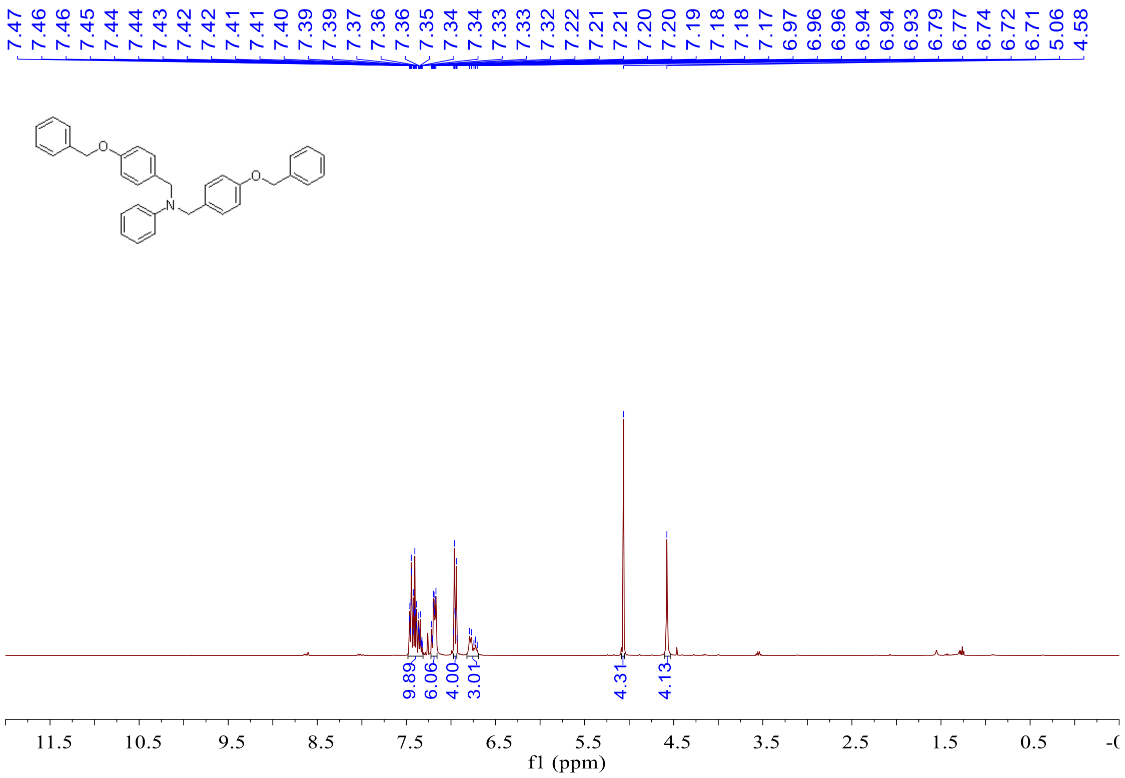

Supplementary Figure 121 ^{1}H NMR spectrum for compound **53a**

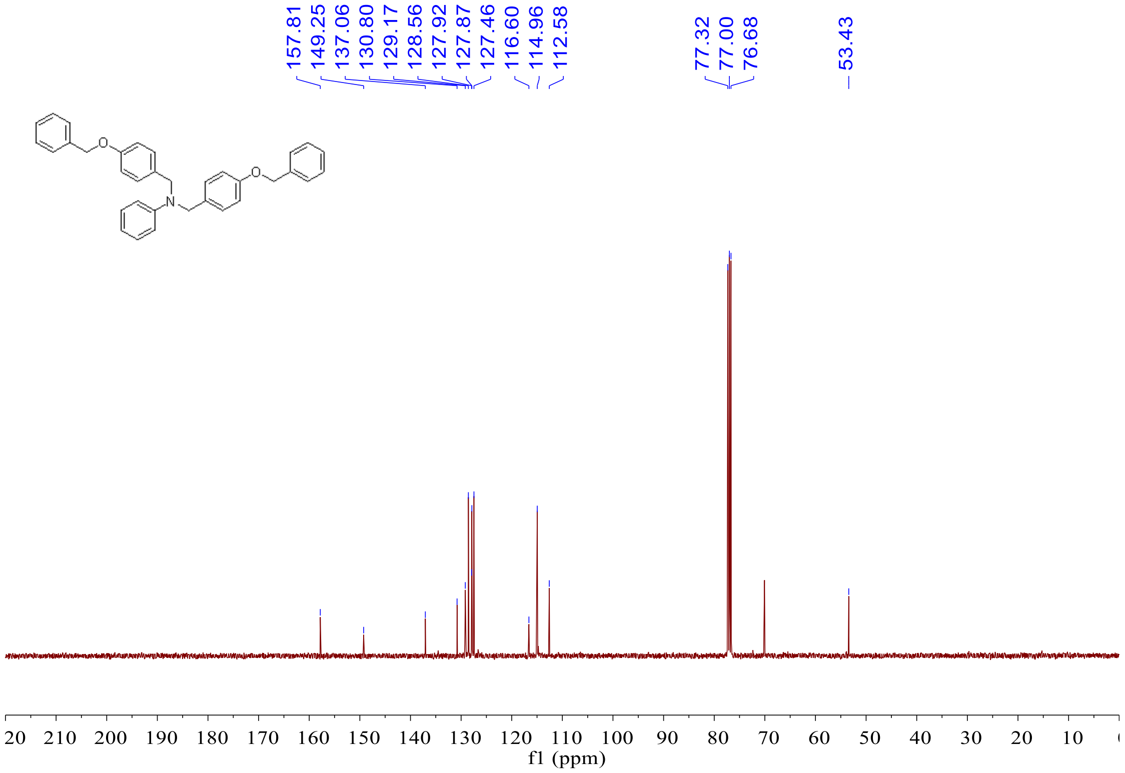

Supplementary Figure 122 ^{13}C NMR spectrum for compound **53a**

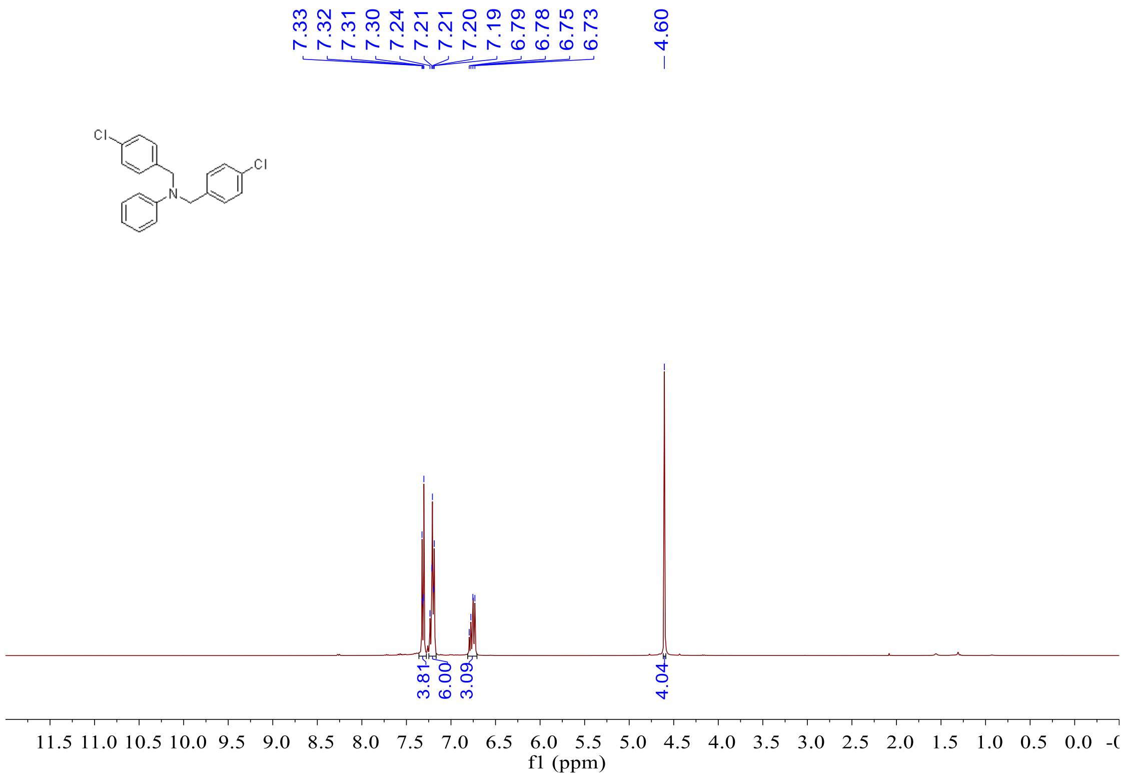

Supplementary Figure 123 ^{1}H NMR spectrum for compound **53b**

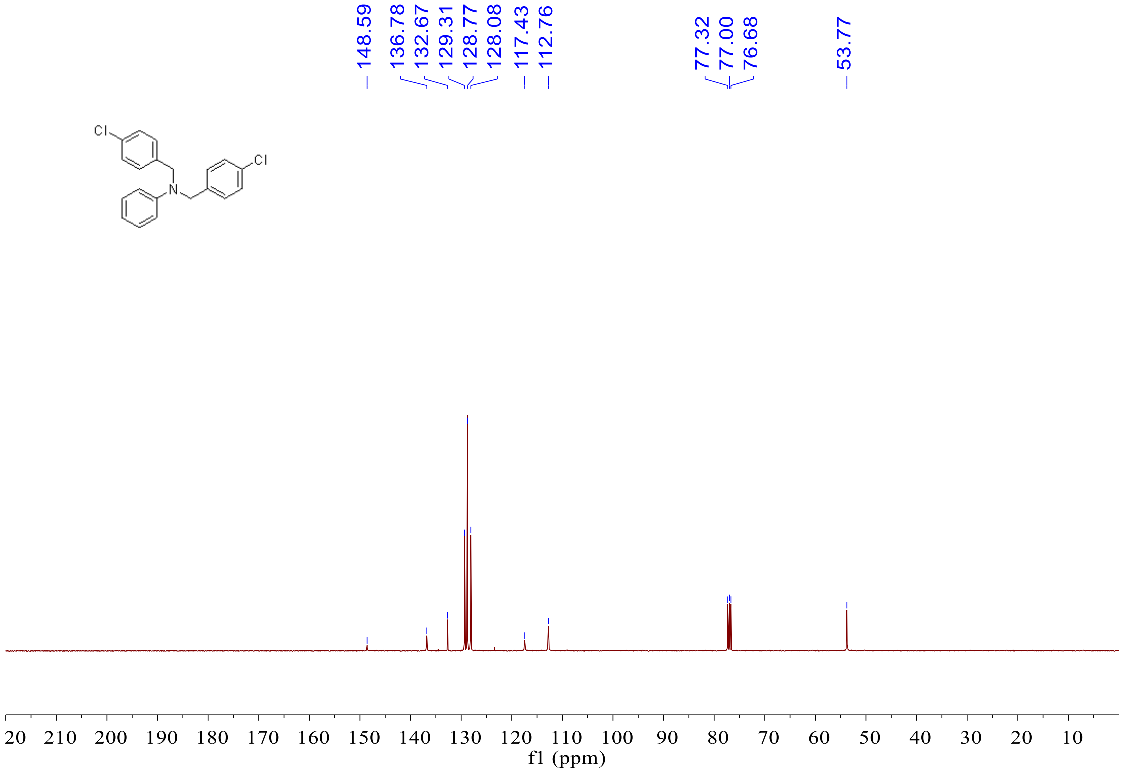

Supplementary Figure 124 ^{13}C NMR spectrum for compound **53b**

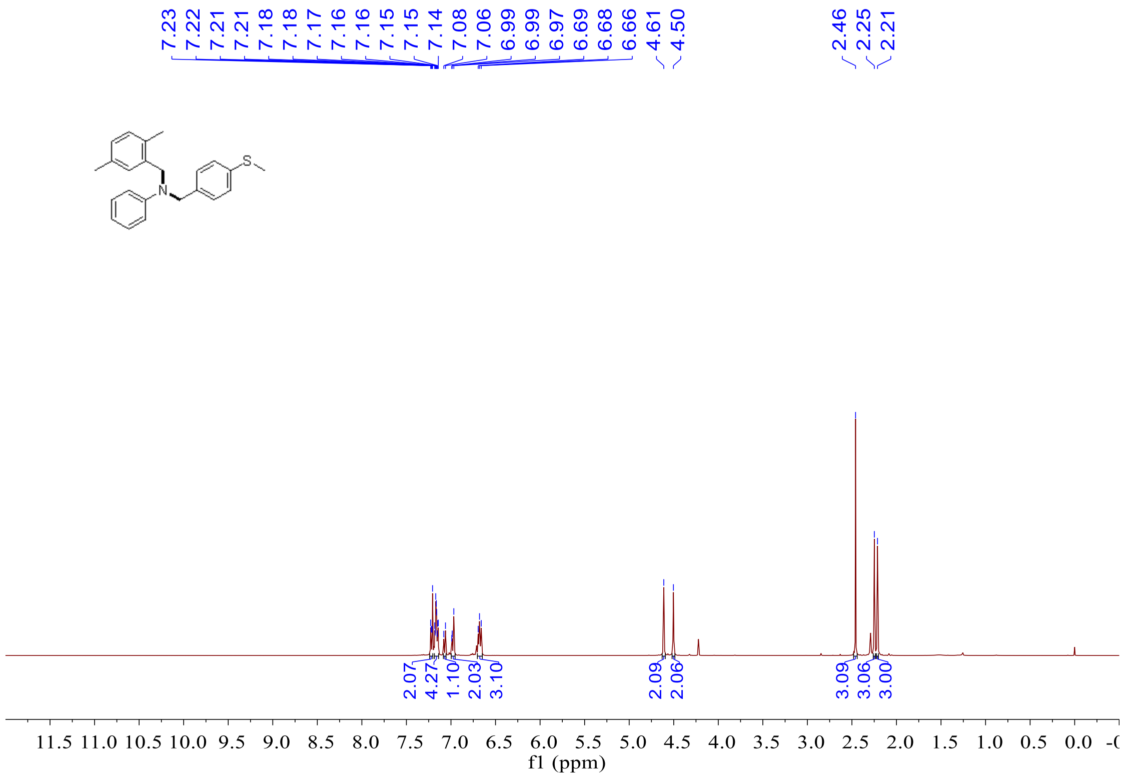

Supplementary Figure 125 ^{1}H NMR spectrum for compound **54**

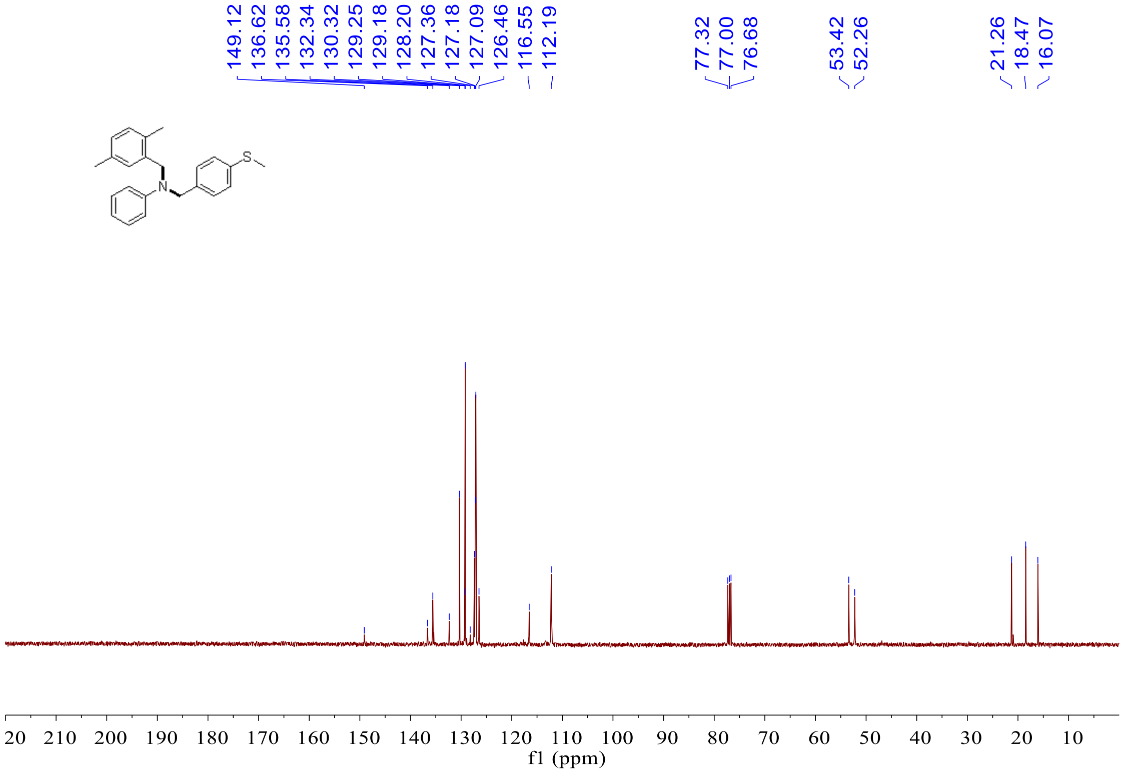

Supplementary Figure 126 ^{13}C NMR spectrum for compound **54**

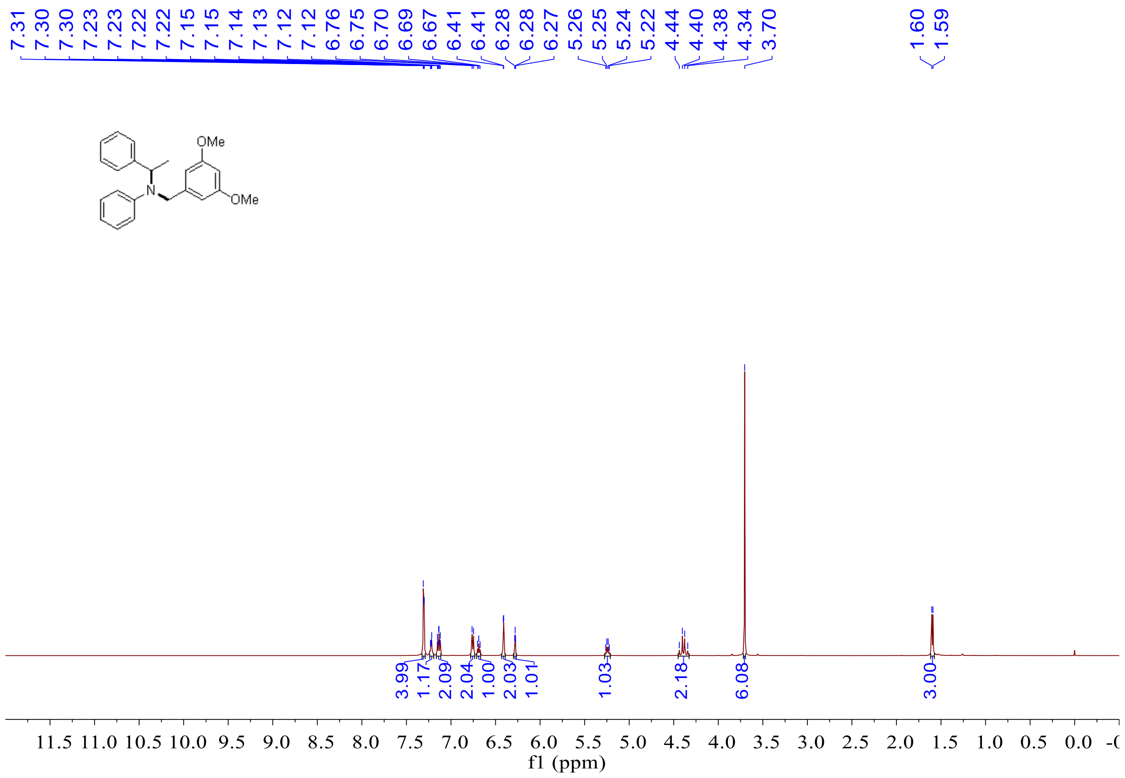

Supplementary Figure 127 ^{1}H NMR spectrum for compound **55**

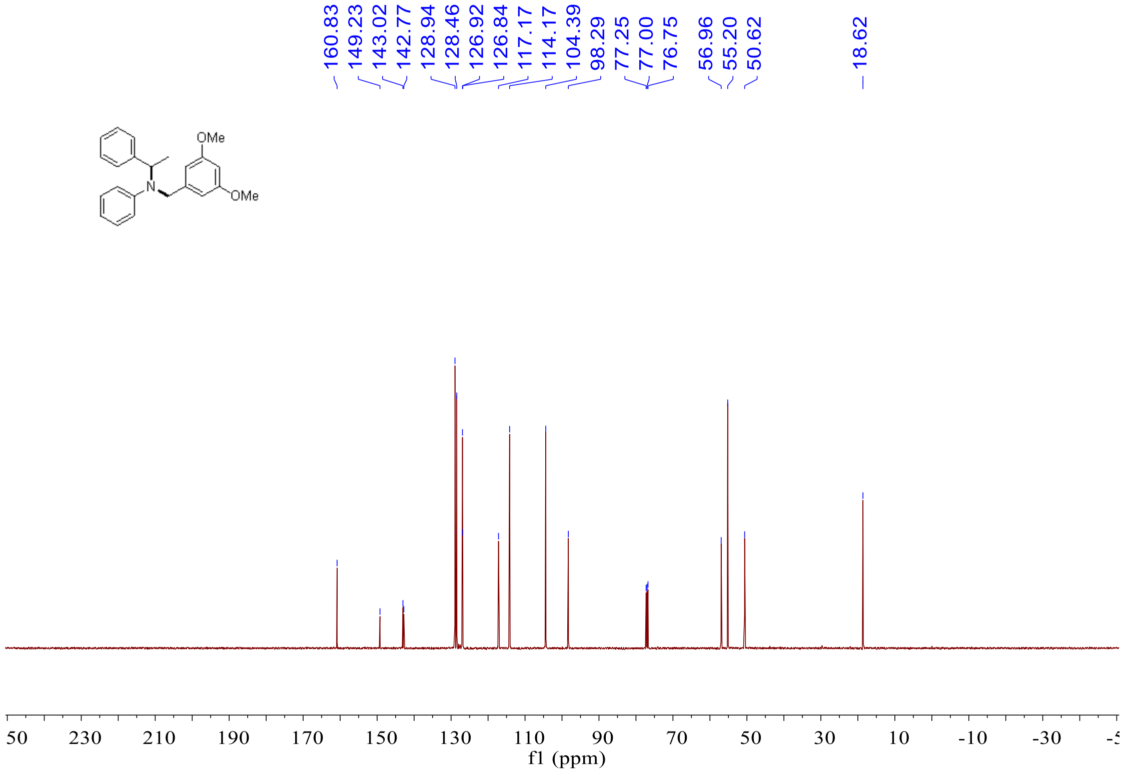

Supplementary Figure 128 ^{13}C NMR spectrum for compound **55**

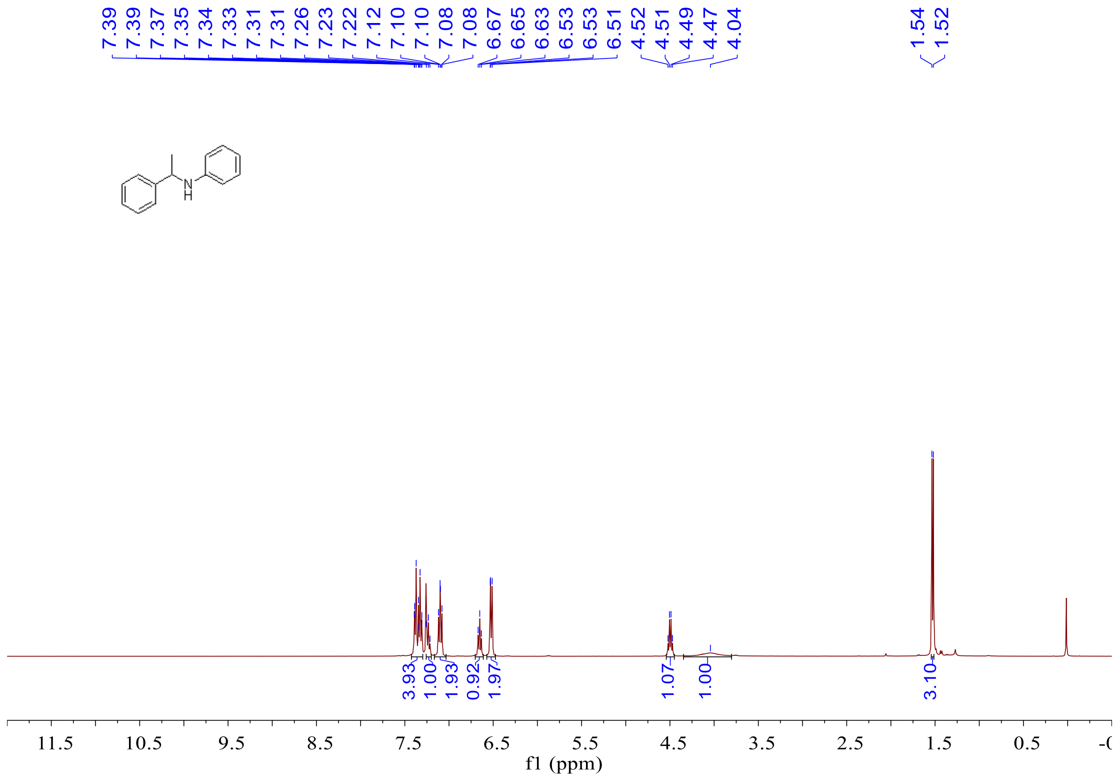

Supplementary Figure 129 ^{1}H NMR spectrum for compound **55a (56a, 57a)**

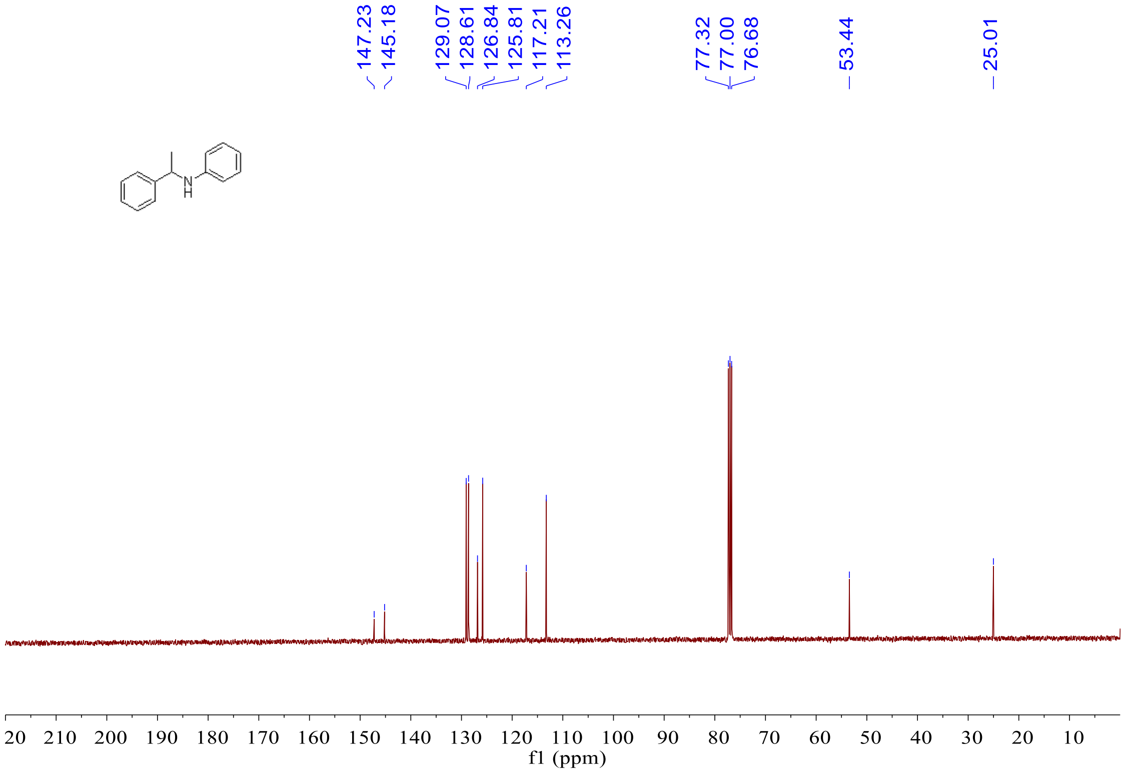

Supplementary Figure 130 ^{13}C NMR spectrum for compound **55a (56a, 57a)**

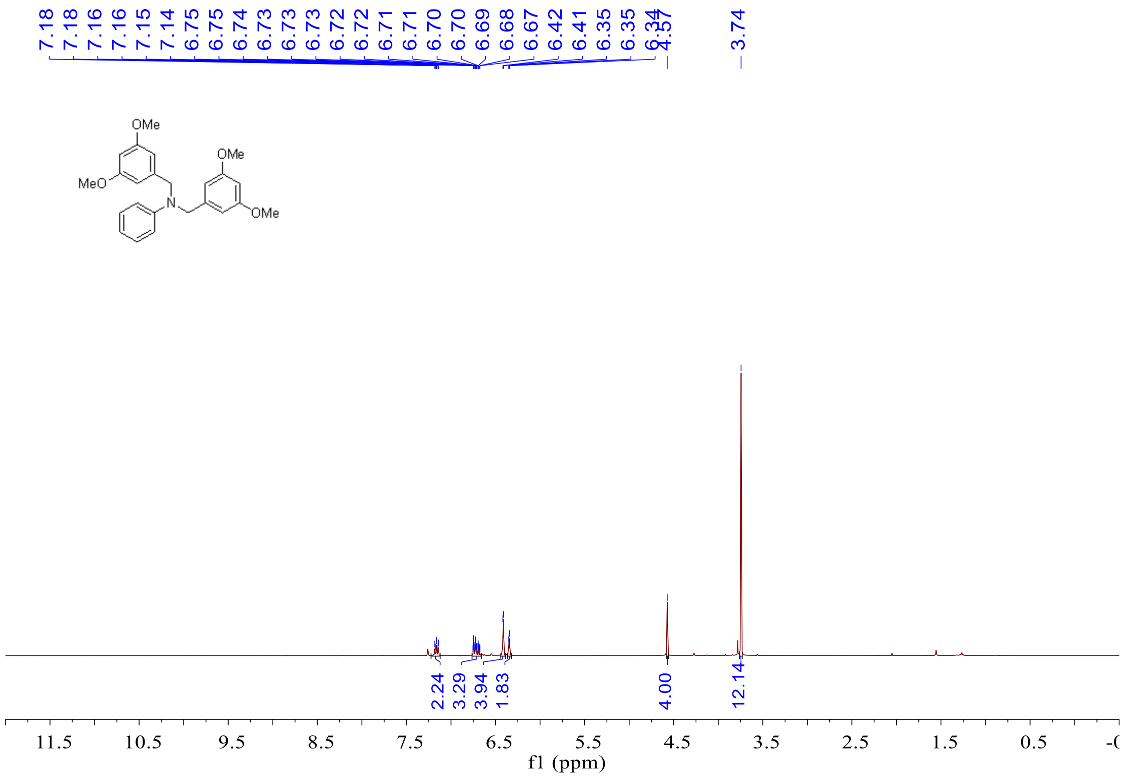

Supplementary Figure 131 ^{1}H NMR spectrum for compound **55b**

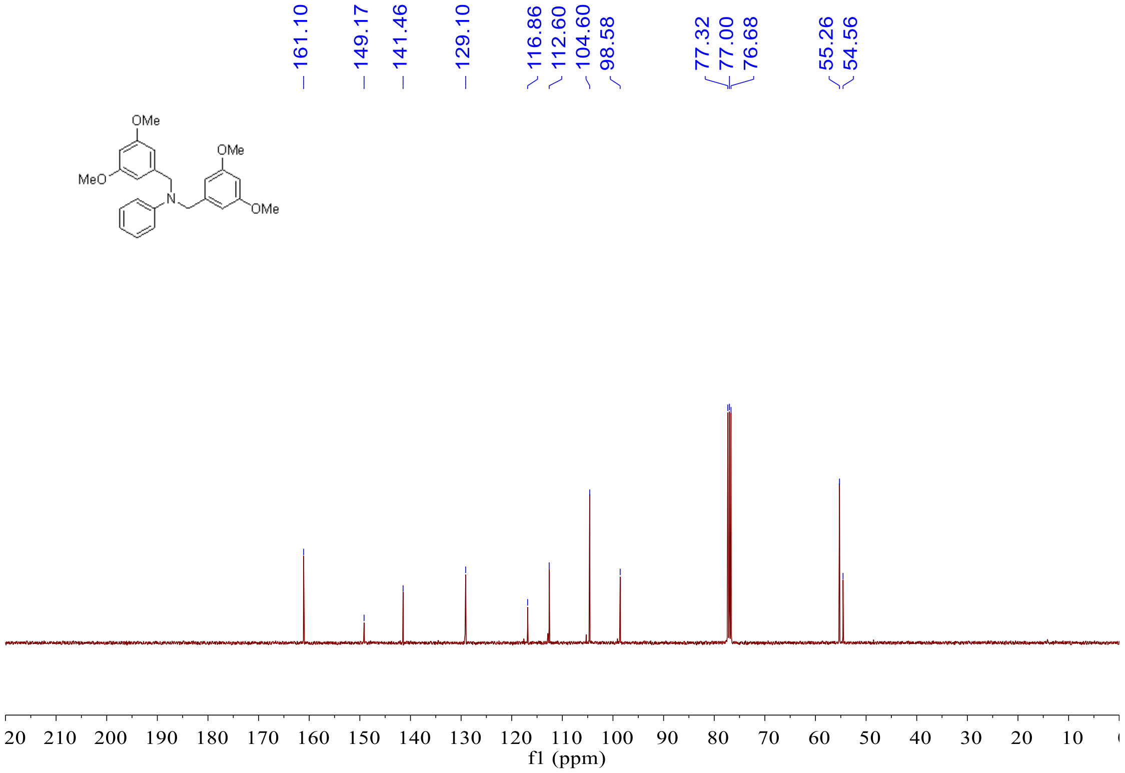

Supplementary Figure 132 ^{13}C NMR spectrum for compound **55b**

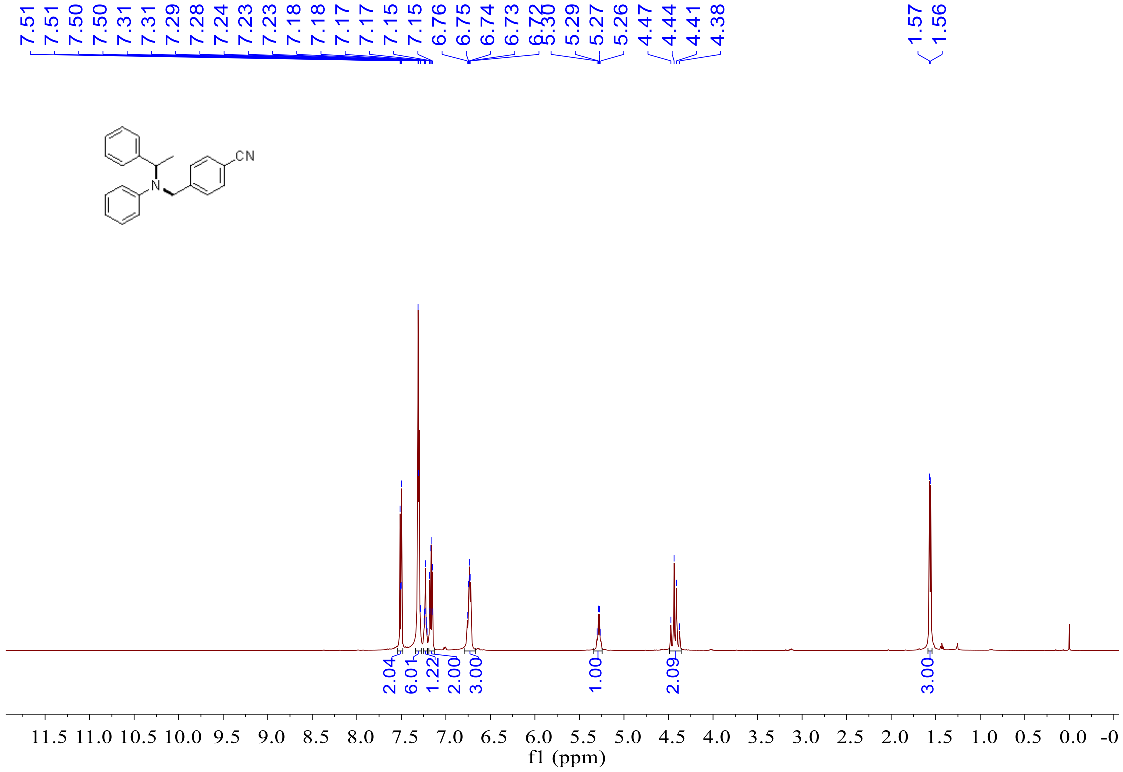

Supplementary Figure 133 ^{1}H NMR spectrum for compound **56**

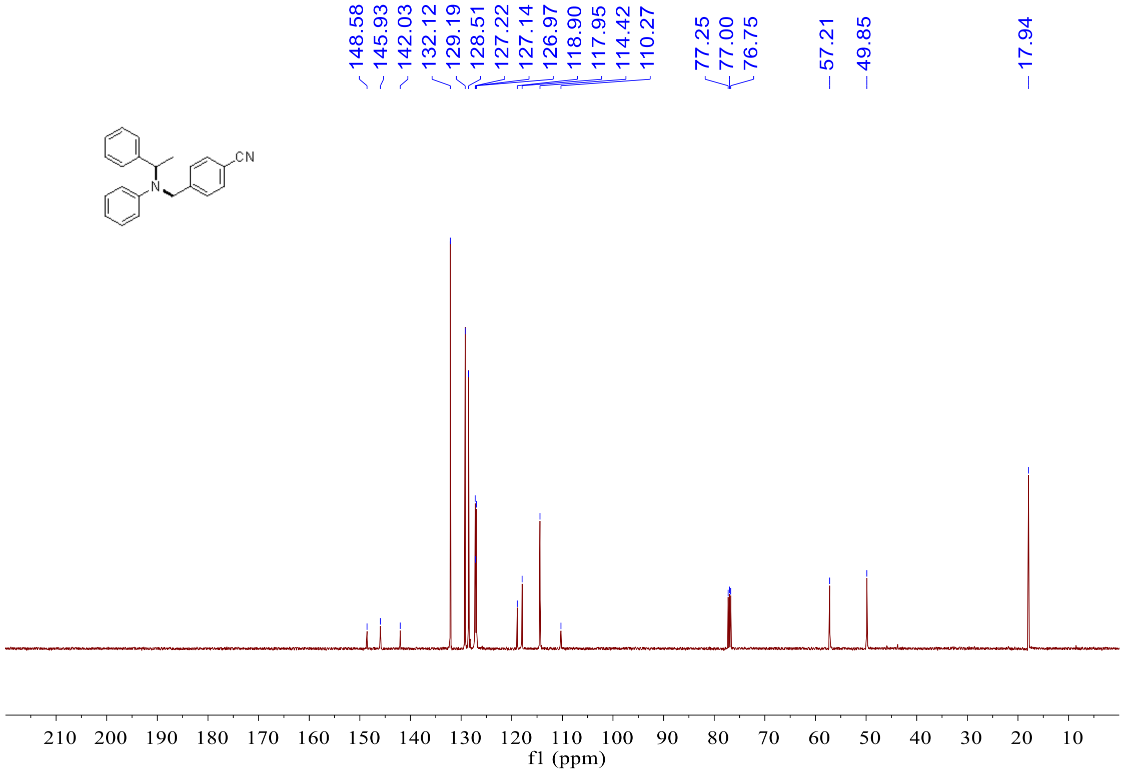

Supplementary Figure 134 ^{13}C NMR spectrum for compound **56**

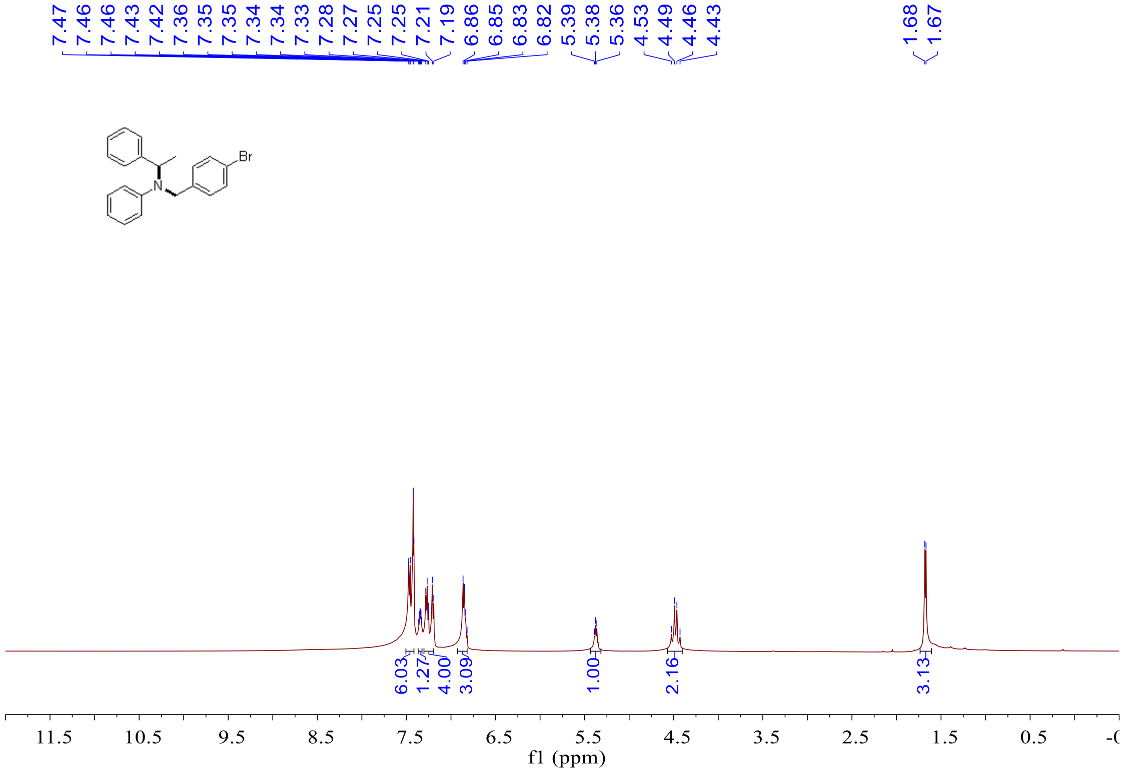

Supplementary Figure 135 ^{1}H NMR spectrum for compound **57**

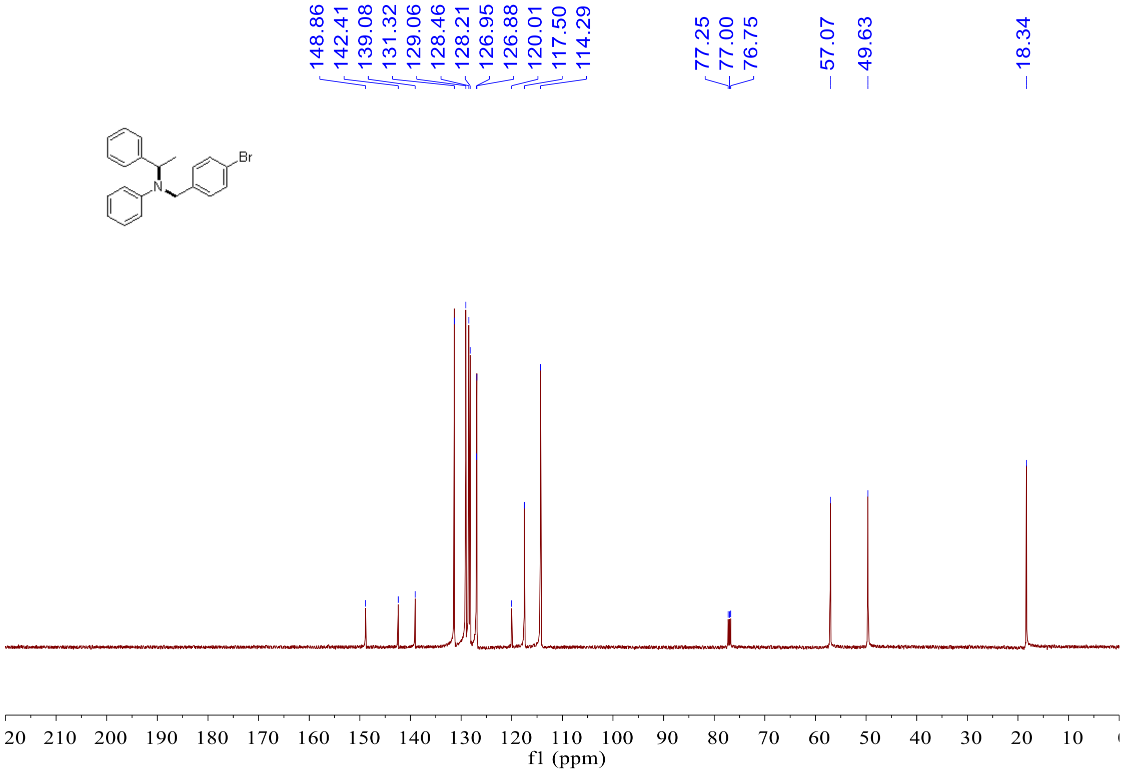

Supplementary Figure 136 ^{13}C NMR spectrum for compound **57**

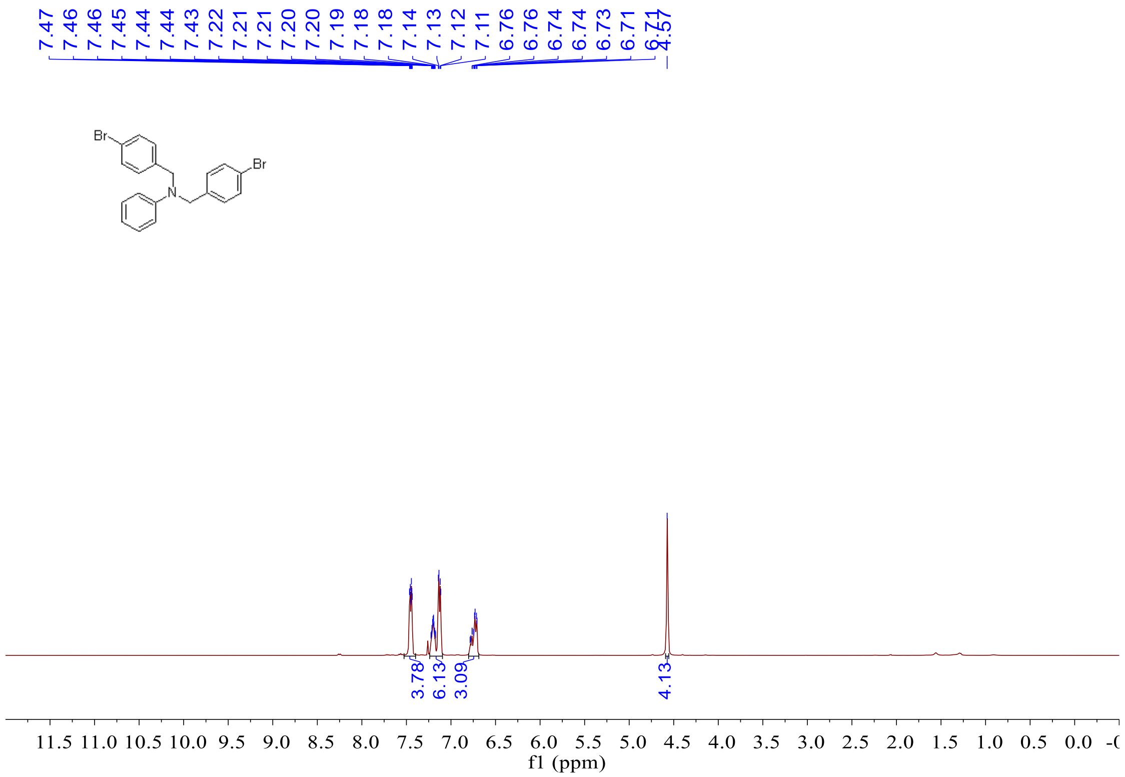

Supplementary Figure 137 ^{1}H NMR spectrum for compound **57b**

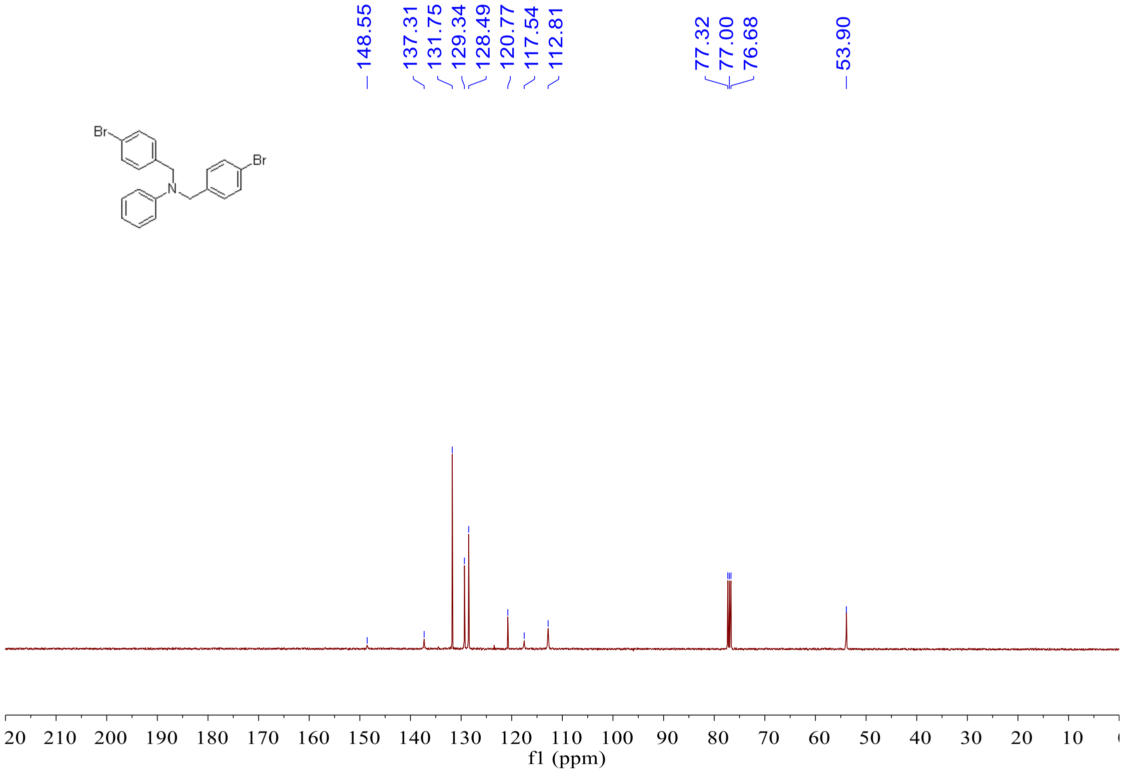

Supplementary Figure 138 ^{13}C NMR spectrum for compound **57b**

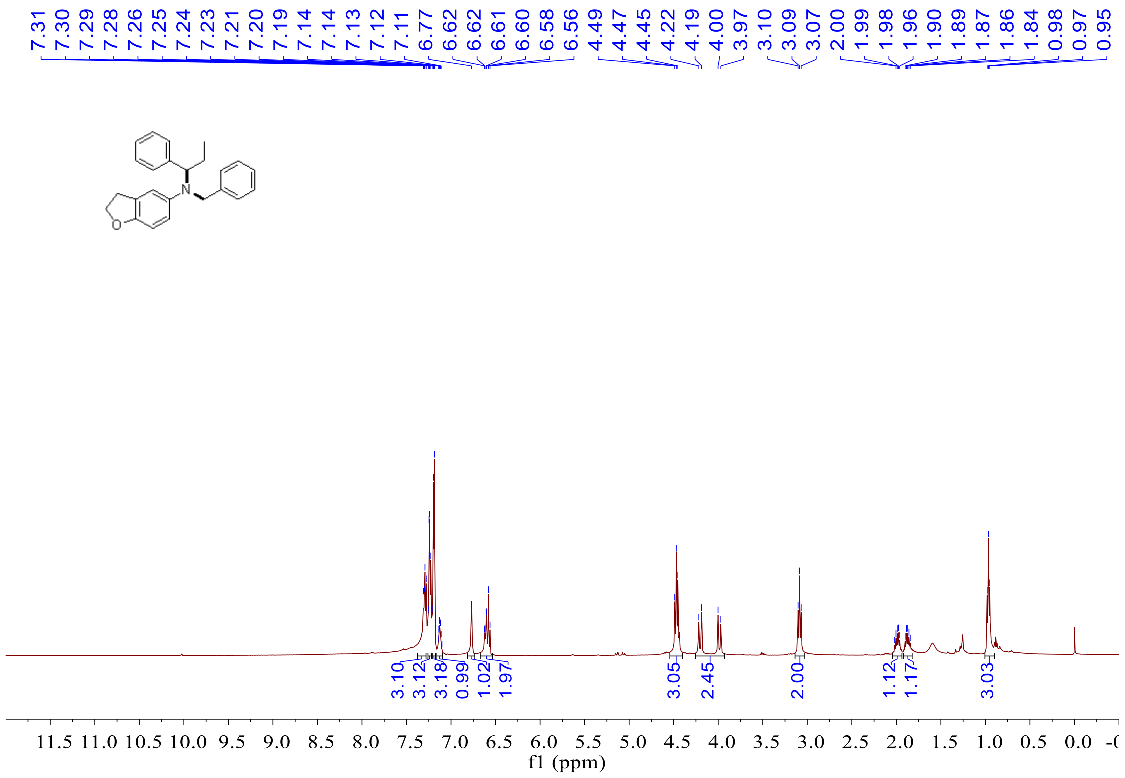

Supplementary Figure 139 ^{1}H NMR spectrum for compound **58**

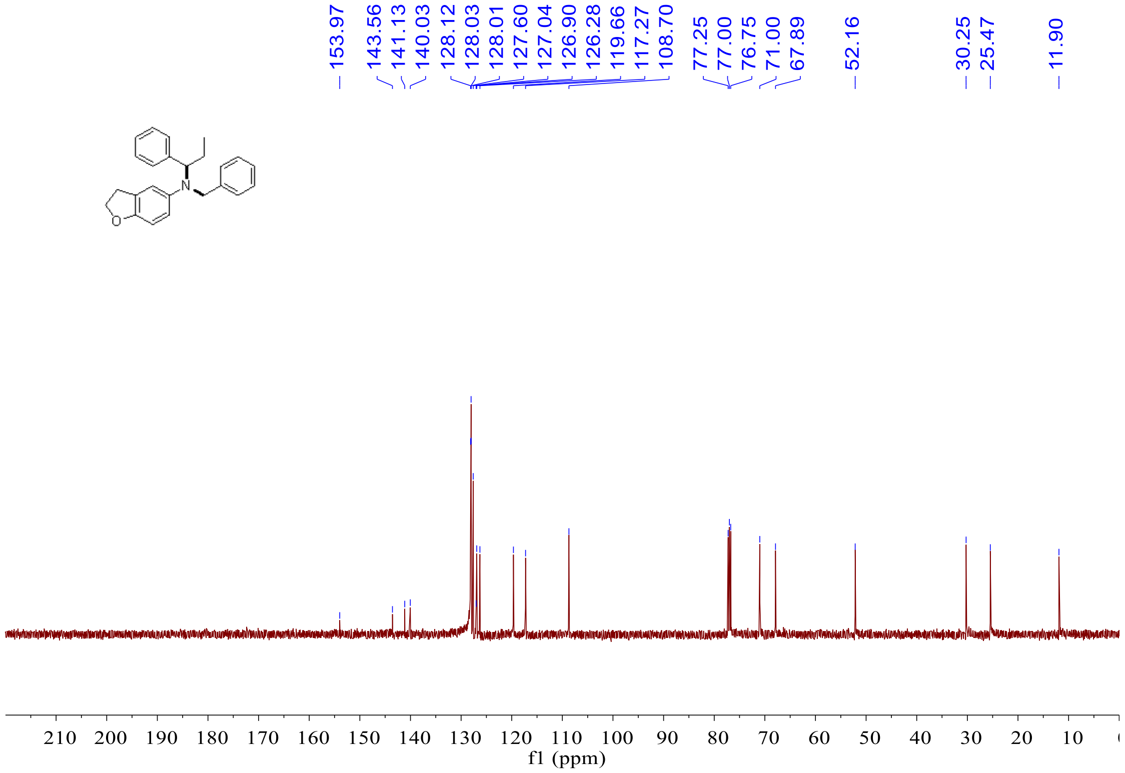

Supplementary Figure 140 ^{13}C NMR spectrum for compound **58**

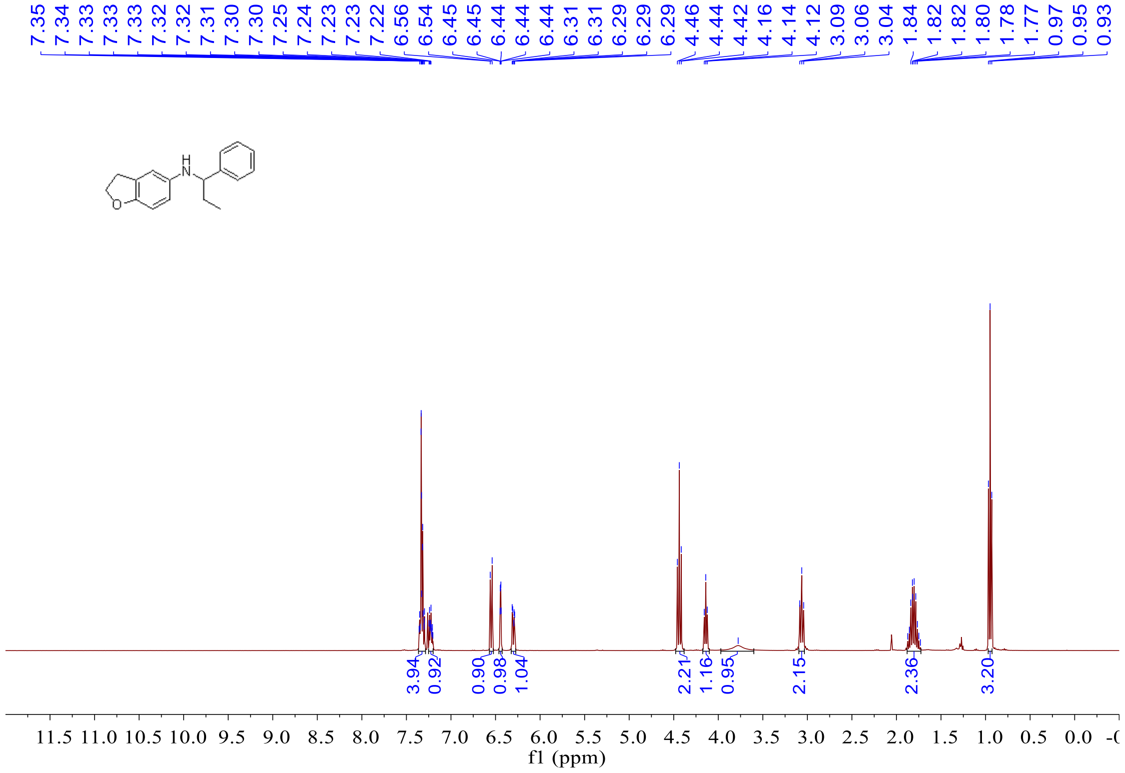

Supplementary Figure 141 ^{1}H NMR spectrum for compound **58a**

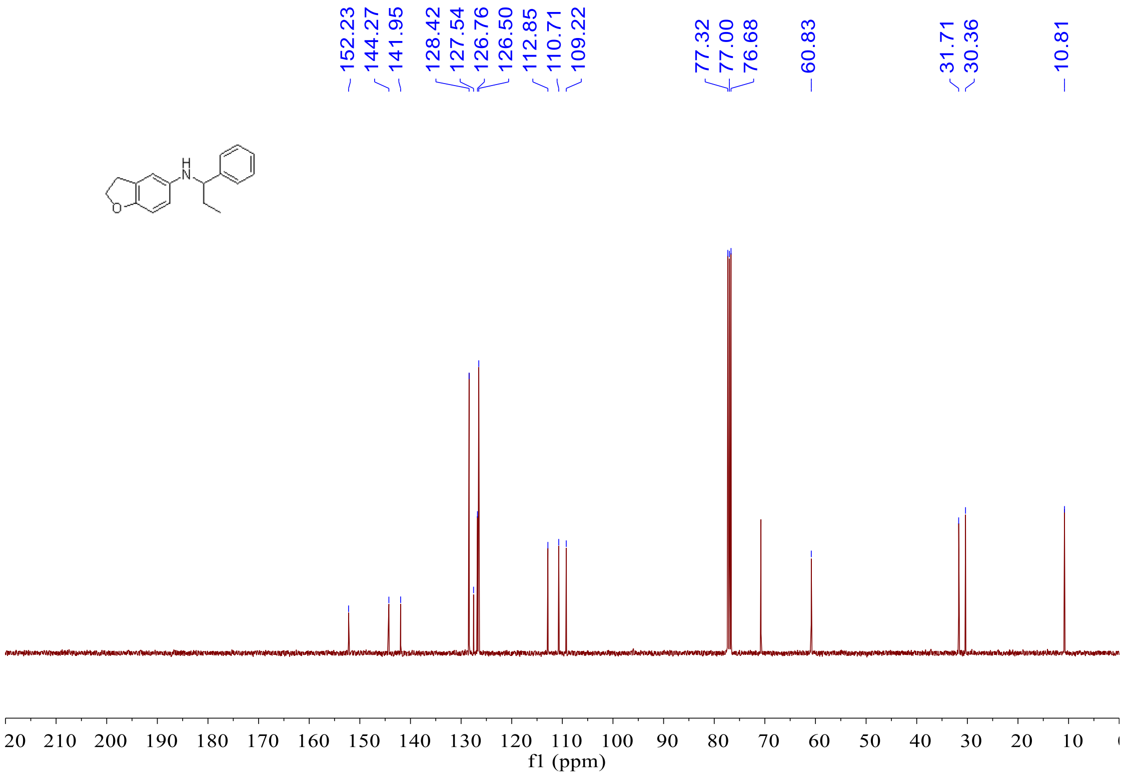

Supplementary Figure 142 ^{13}C NMR spectrum for compound **58a**

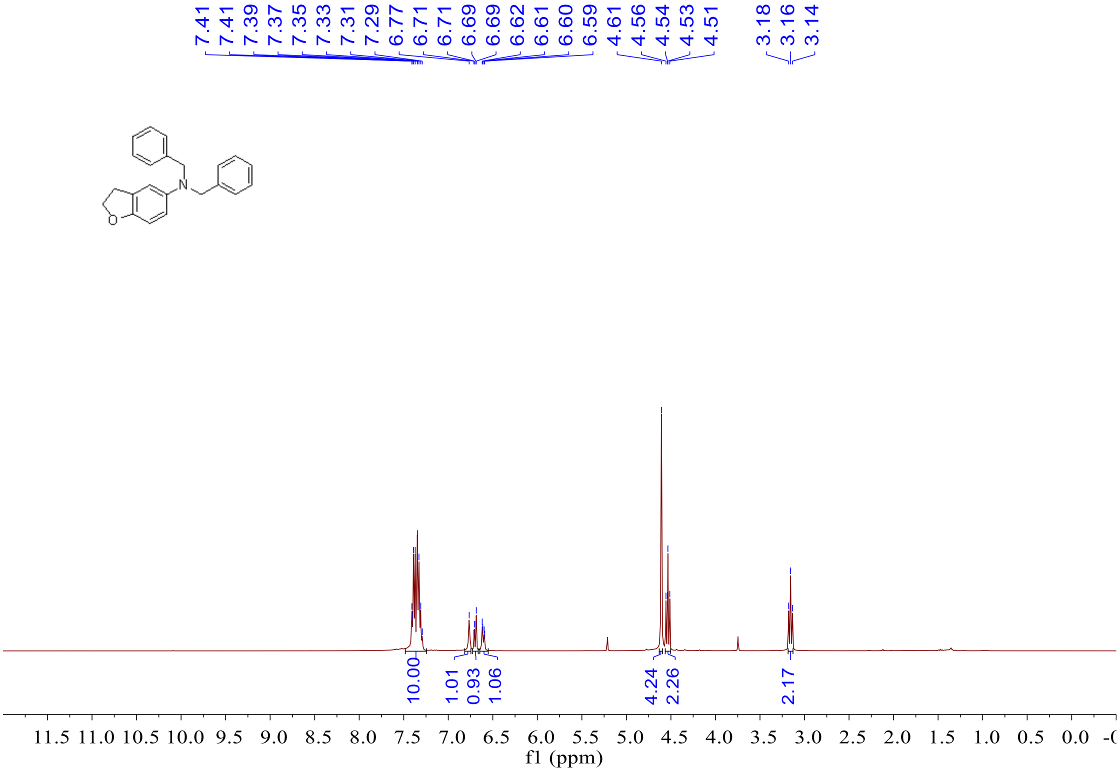

Supplementary Figure 143 ^{1}H NMR spectrum for compound **58b**

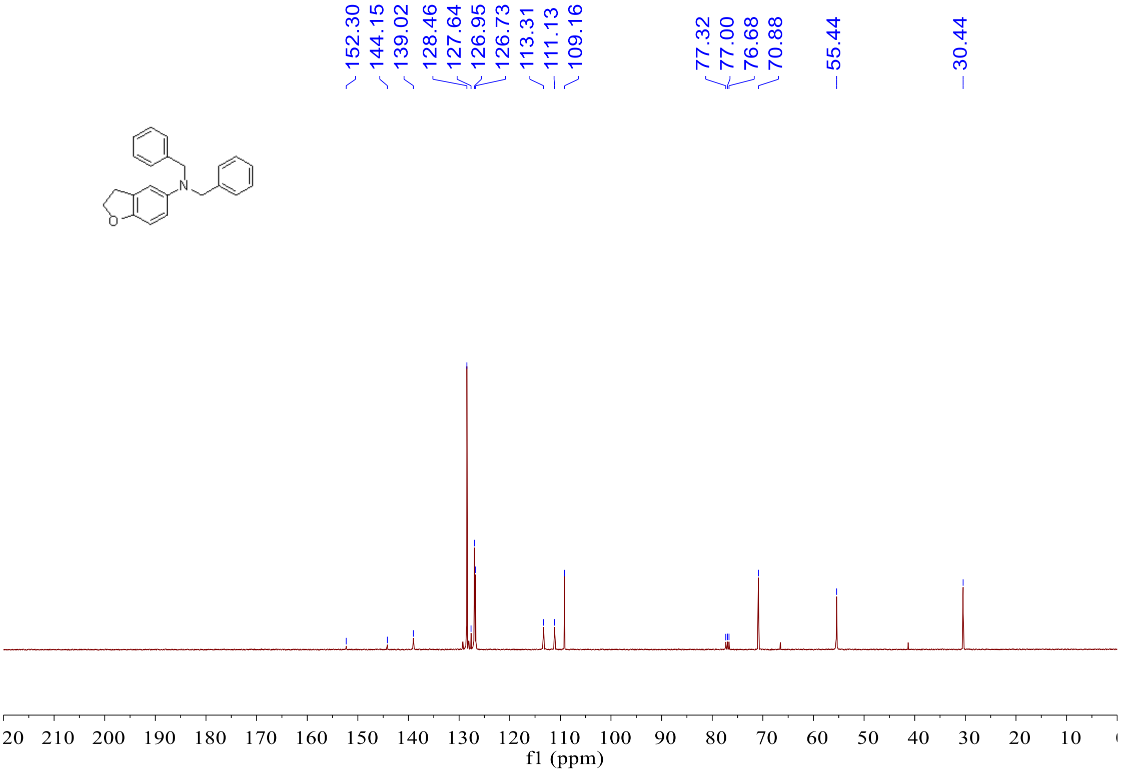

Supplementary Figure 144 ^{13}C NMR spectrum for compound **58b**

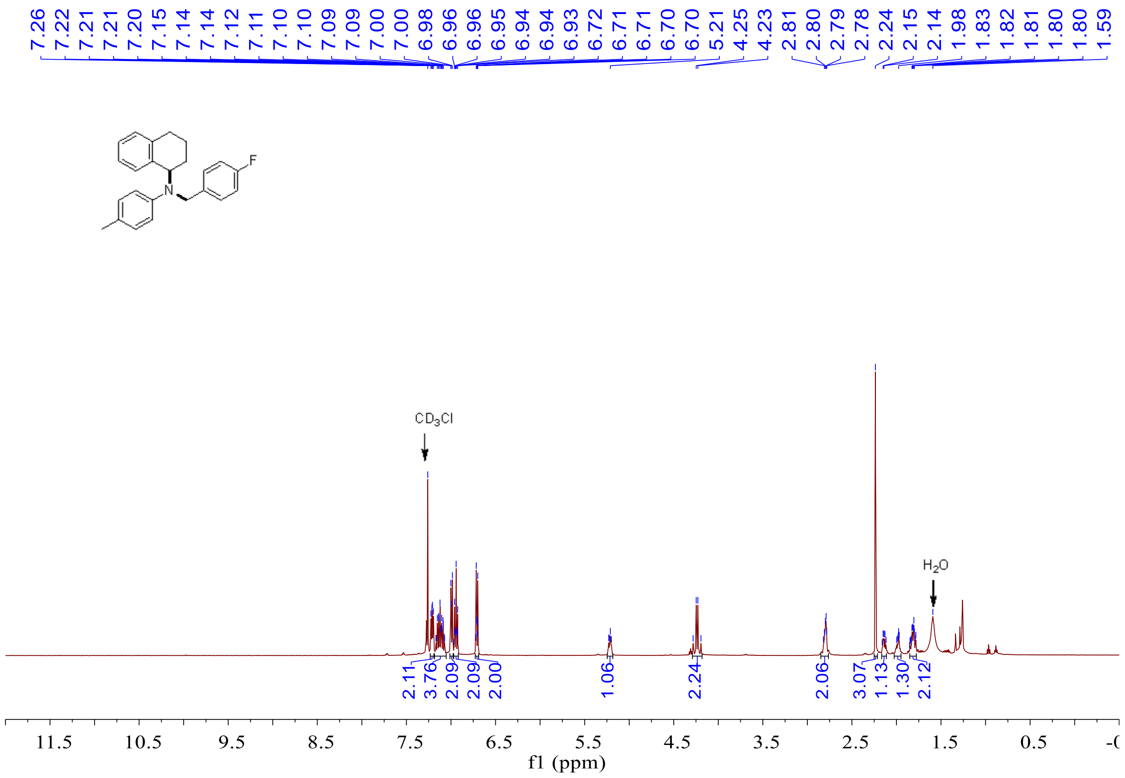

Supplementary Figure 145 ^{1}H NMR spectrum for compound **59**

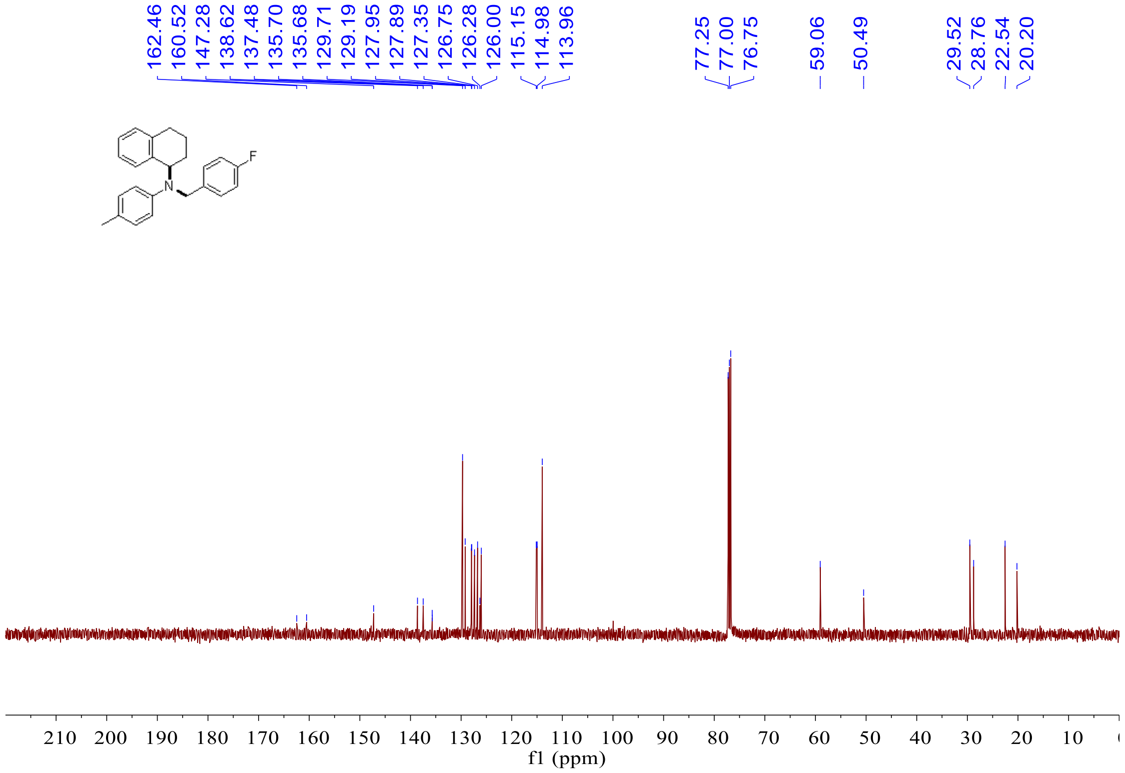

Supplementary Figure 146 ^{13}C NMR spectrum for compound **59**

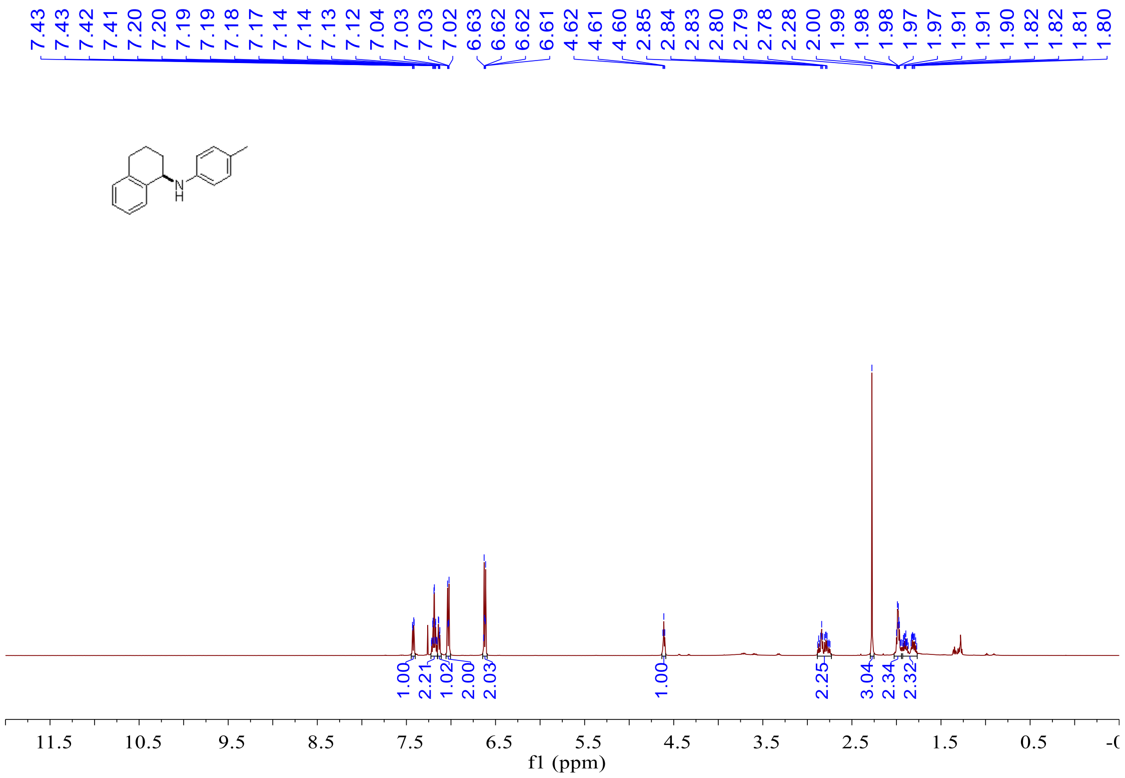

Supplementary Figure 147 ^{1}H NMR spectrum for compound **59a**

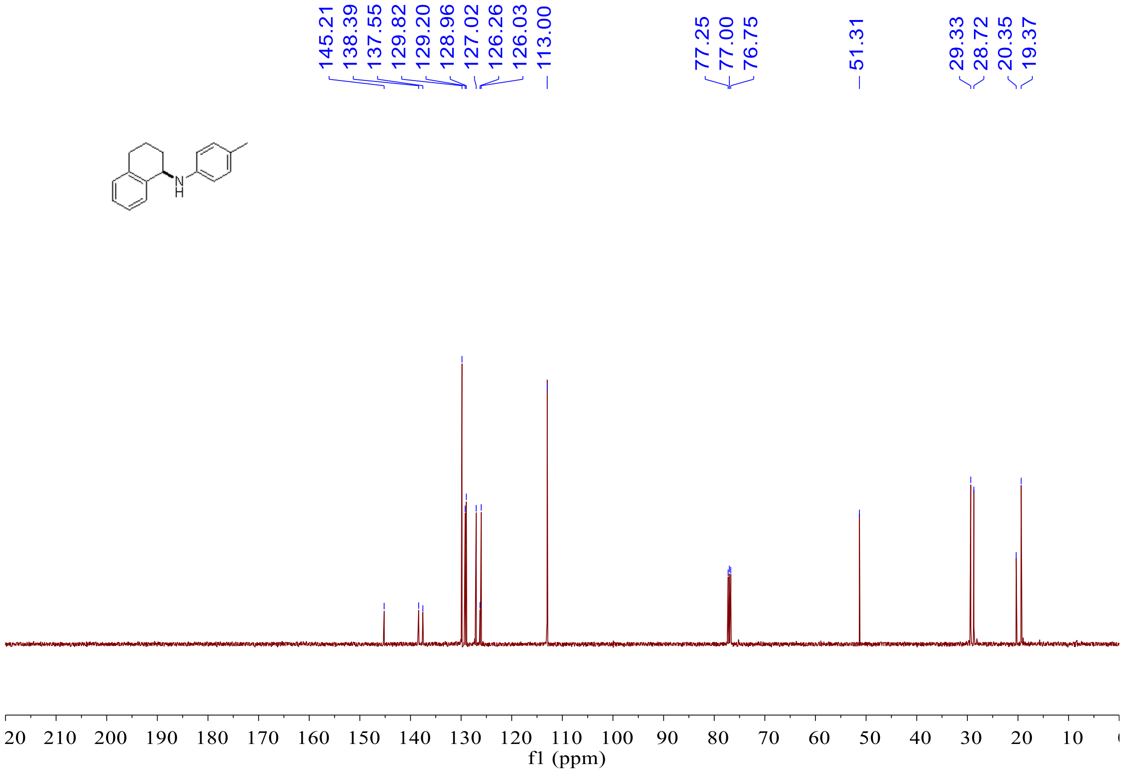

Supplementary Figure 148 ^{13}C NMR spectrum for compound **59a**

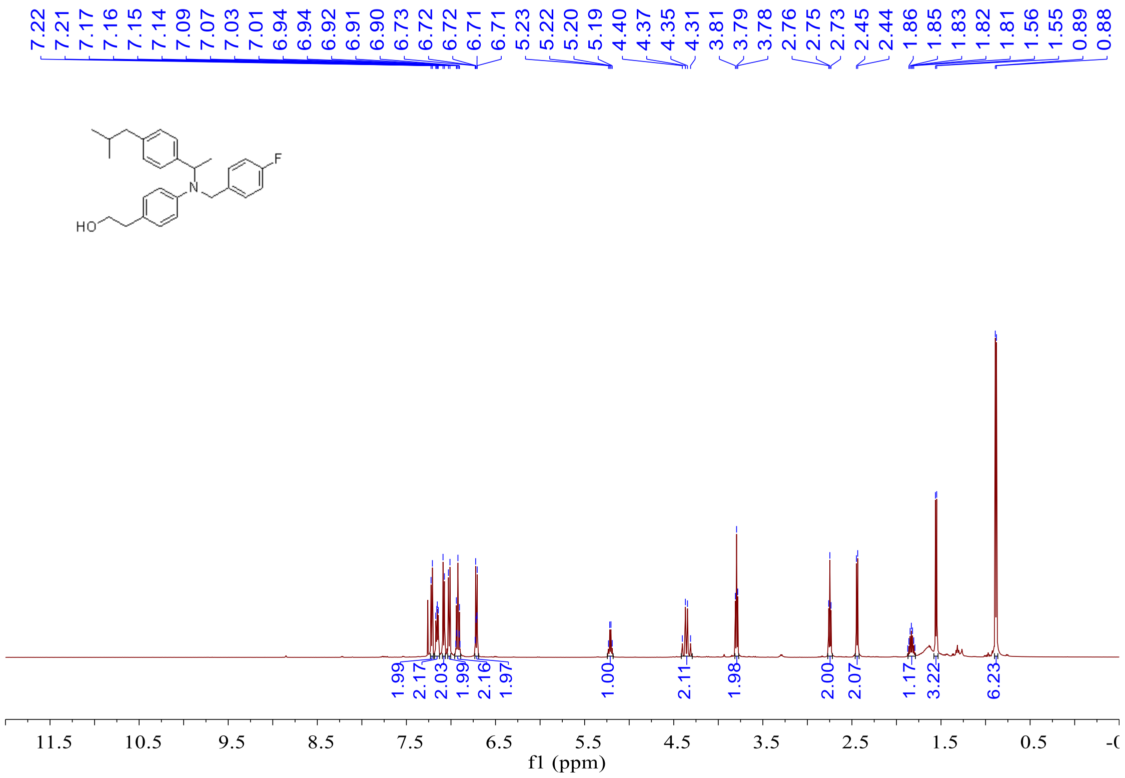

Supplementary Figure 149 ^{1}H NMR spectrum for compound **60**

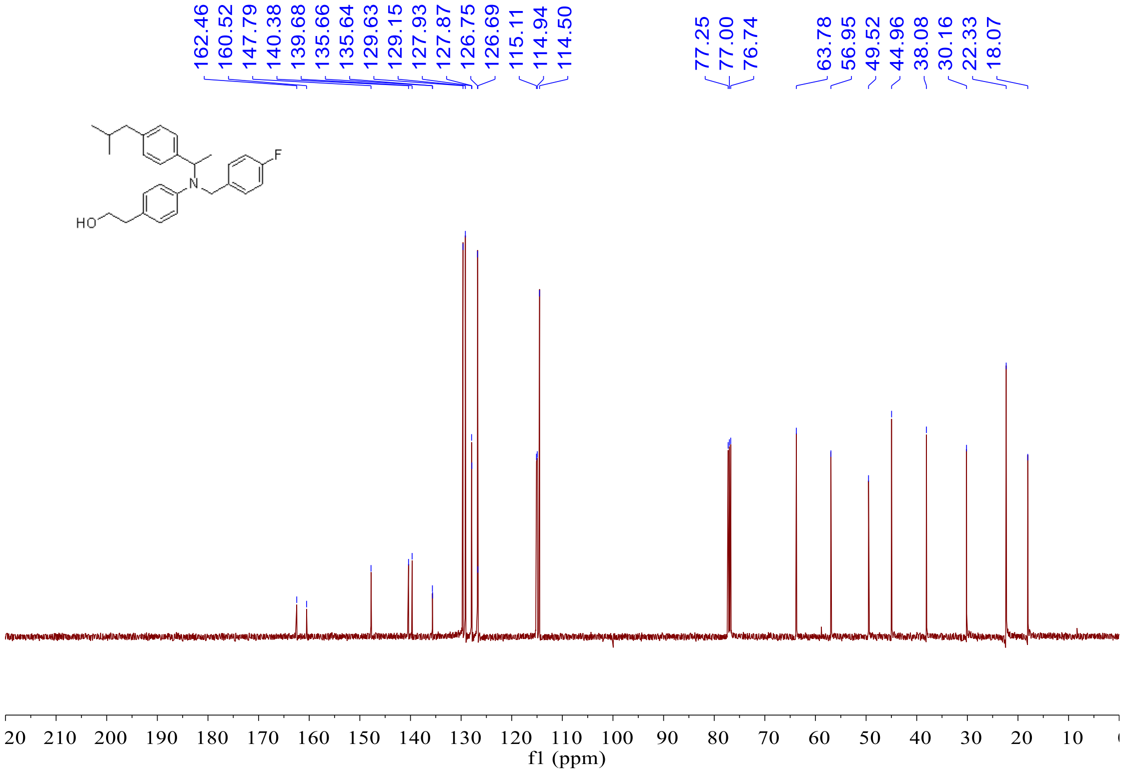

Supplementary Figure 150 ^{13}C NMR spectrum for compound **60**

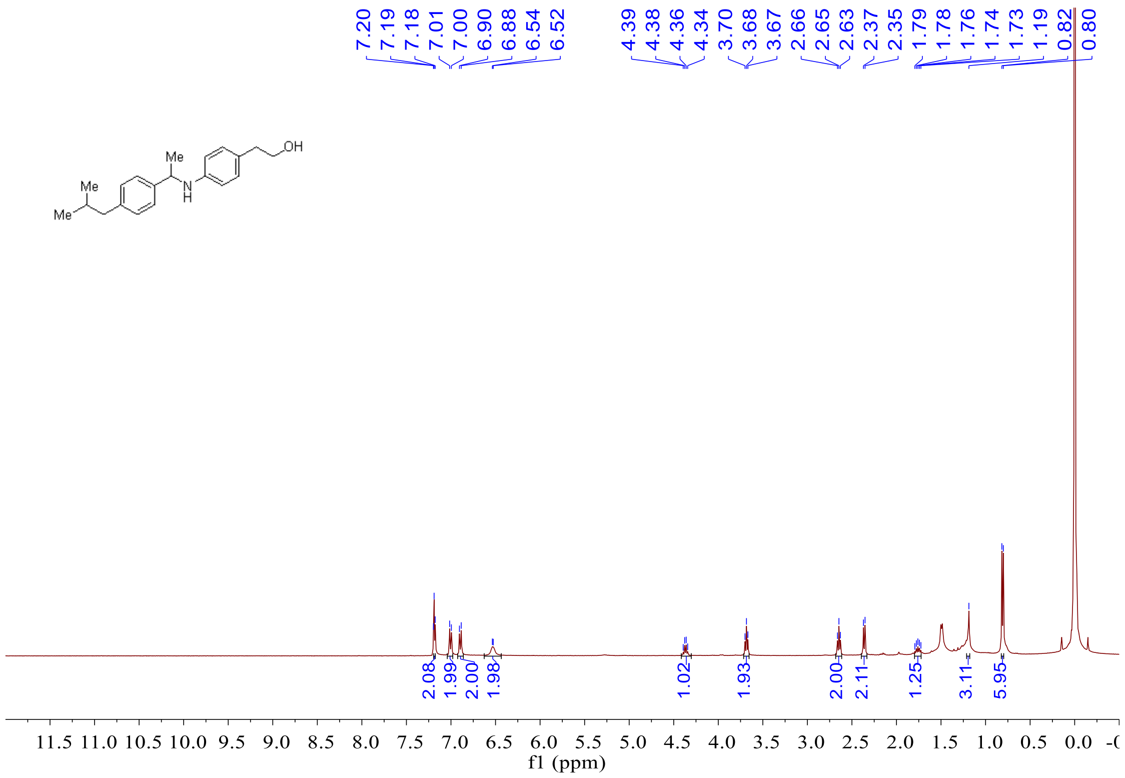

Supplementary Figure 151 ^{1}H NMR spectrum for compound **60a**

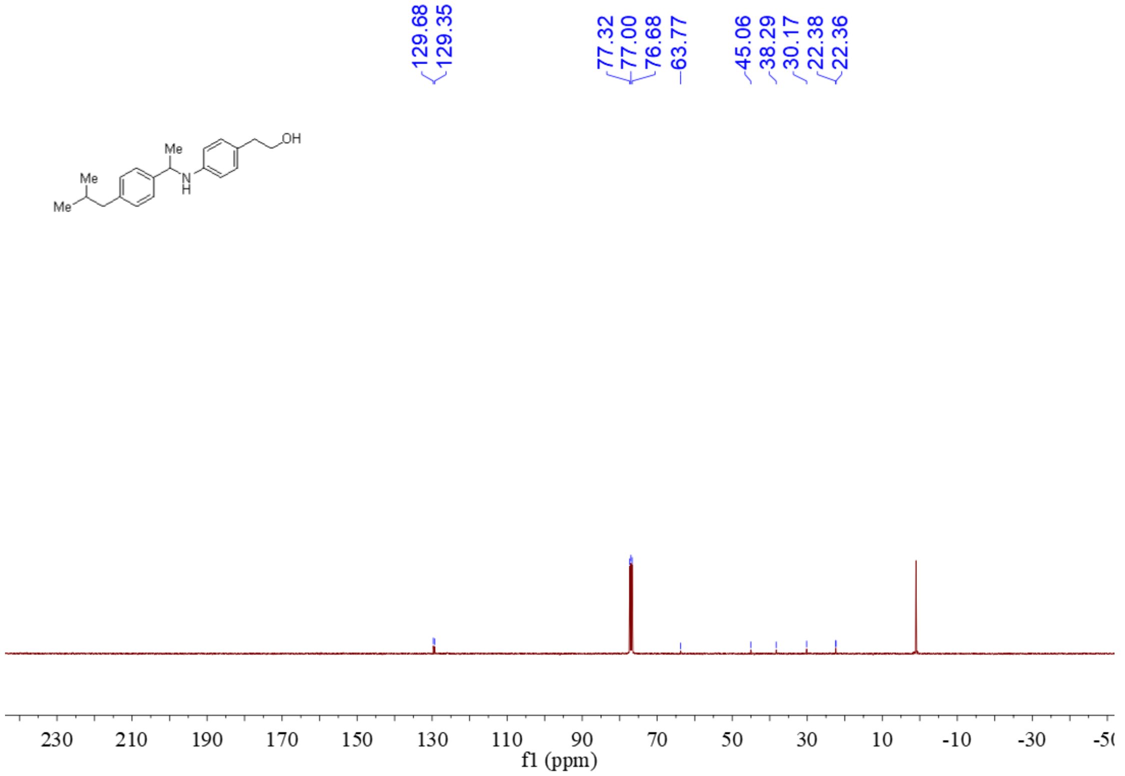

Supplementary Figure 152 ^{13}C NMR spectrum for compound **60a**

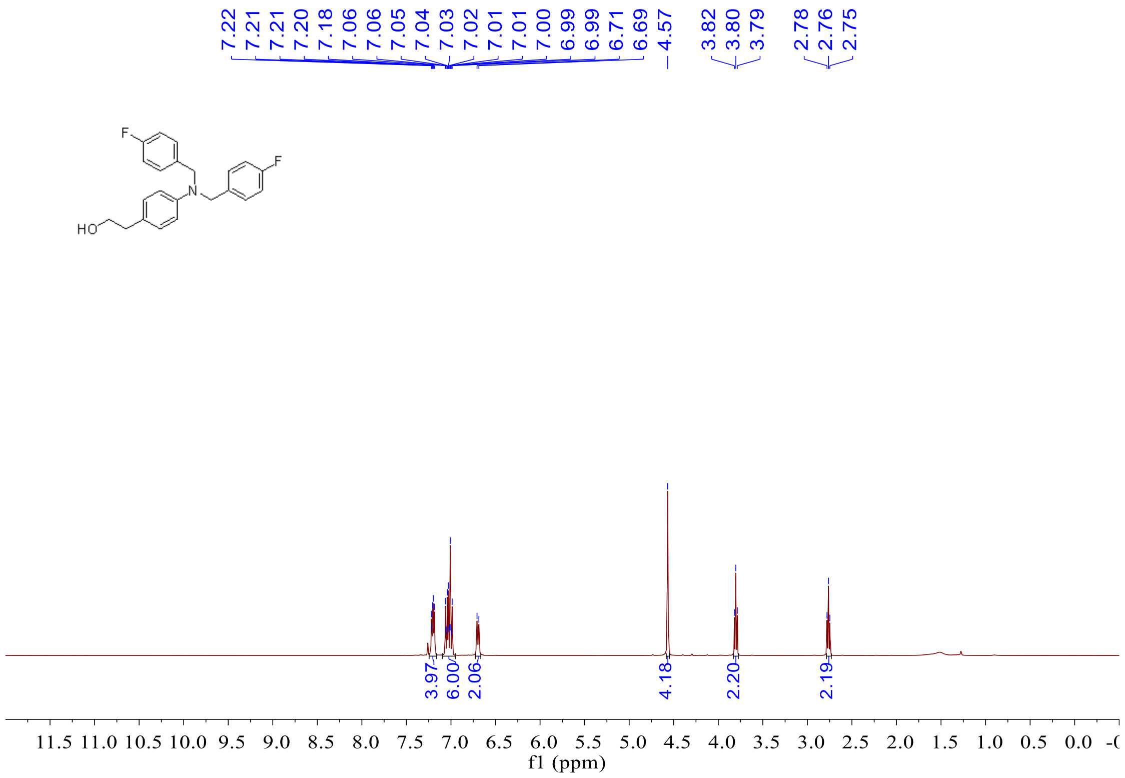

Supplementary Figure 153 ^{1}H NMR spectrum for compound **60b**

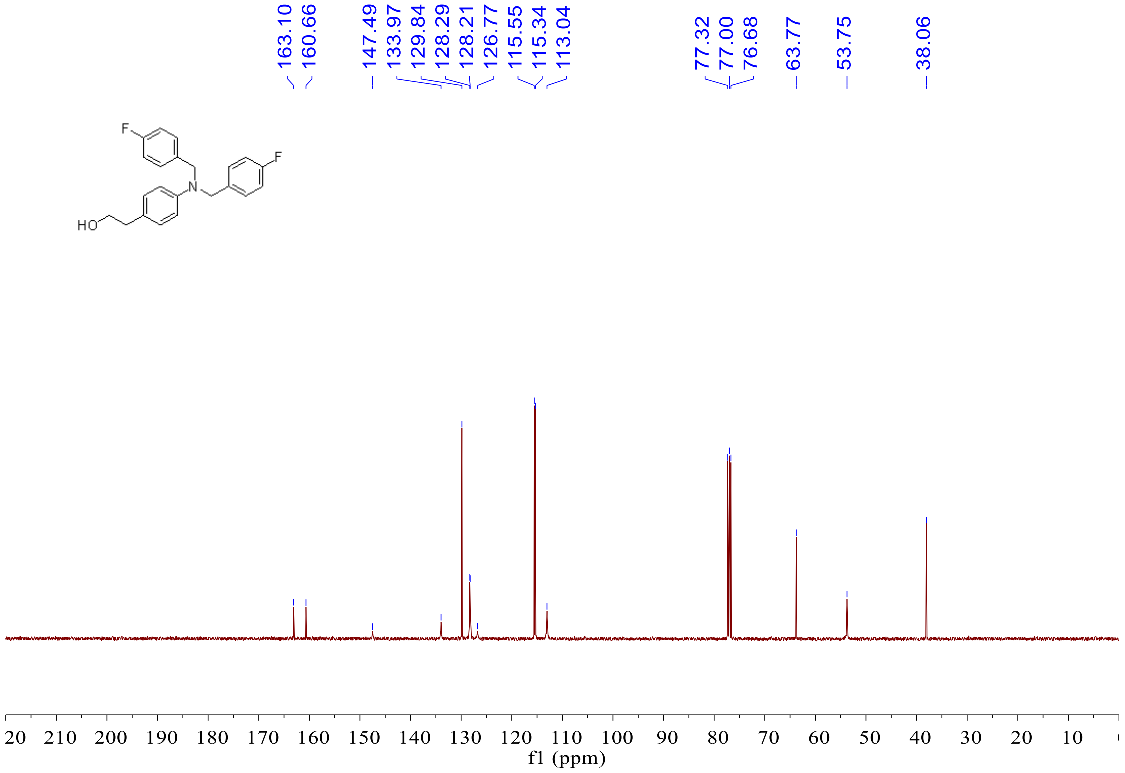

Supplementary Figure 154 ^{13}C NMR spectrum for compound **60b**

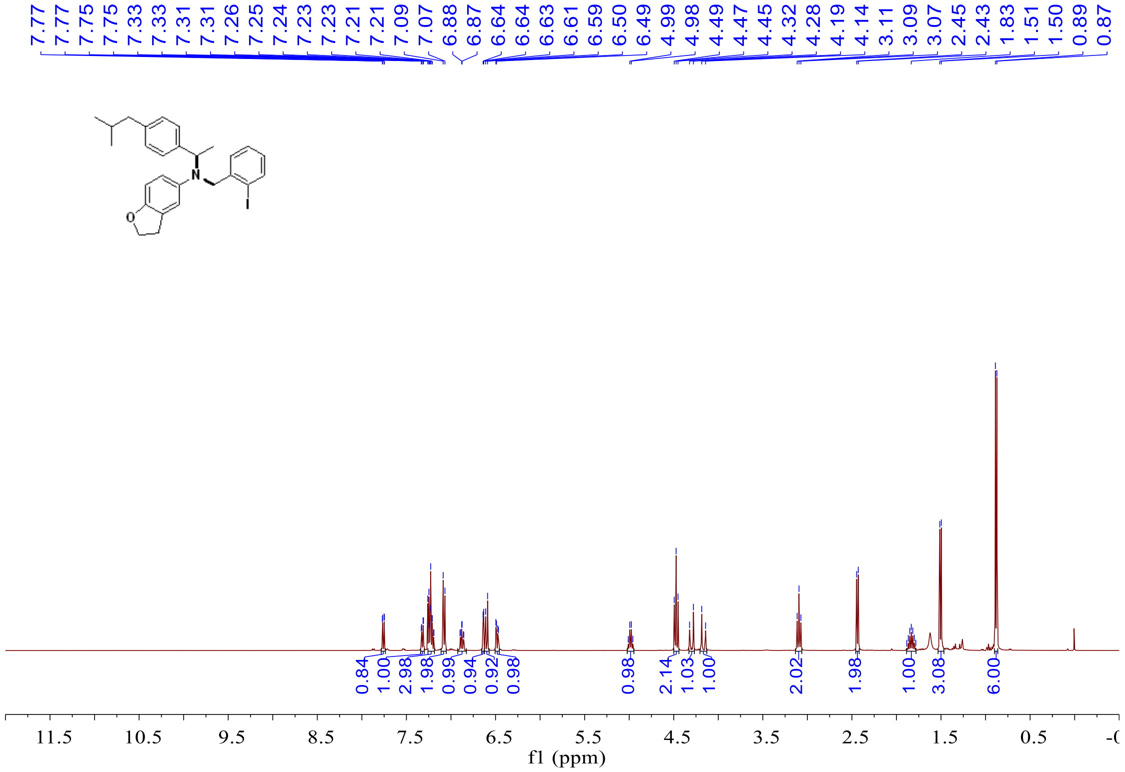

Supplementary Figure 155 ^{1}H NMR spectrum for compound **61**

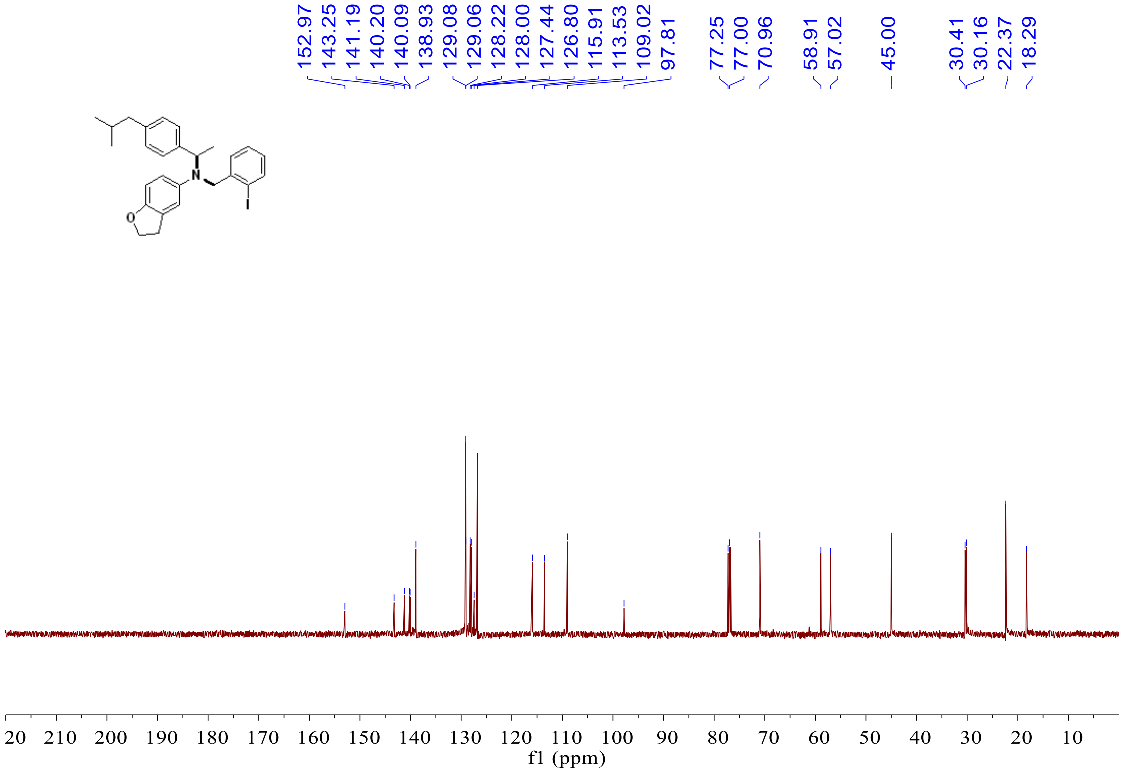

Supplementary Figure 156 ^{13}C NMR spectrum for compound **61**

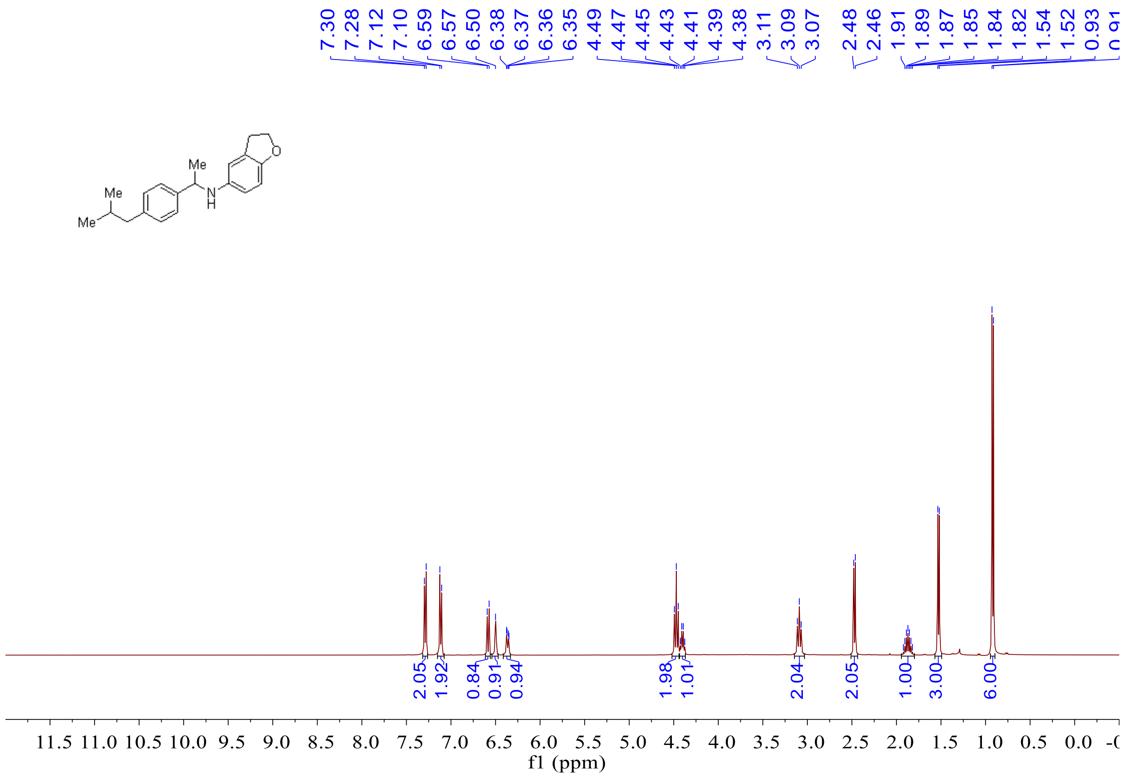

Supplementary Figure 157 ^{1}H NMR spectrum for compound **61a**

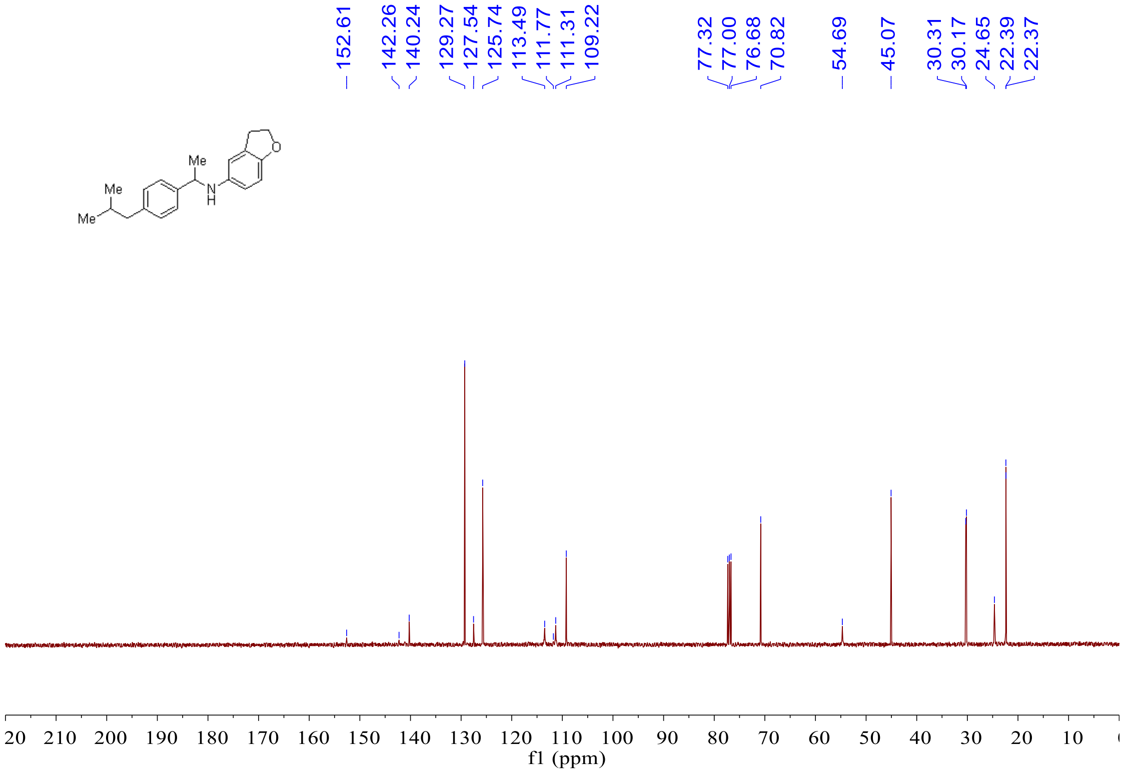

Supplementary Figure 158 ^{13}C NMR spectrum for compound **61a**

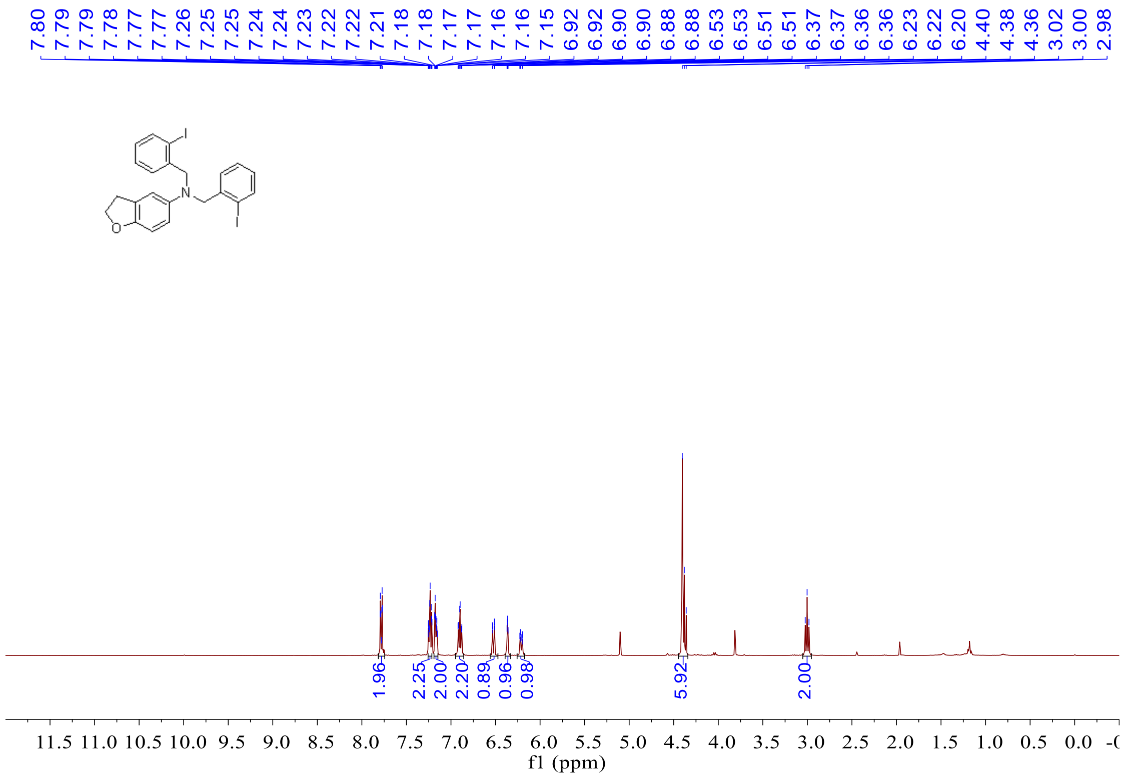

Supplementary Figure 159 ^{1}H NMR spectrum for compound **61b**

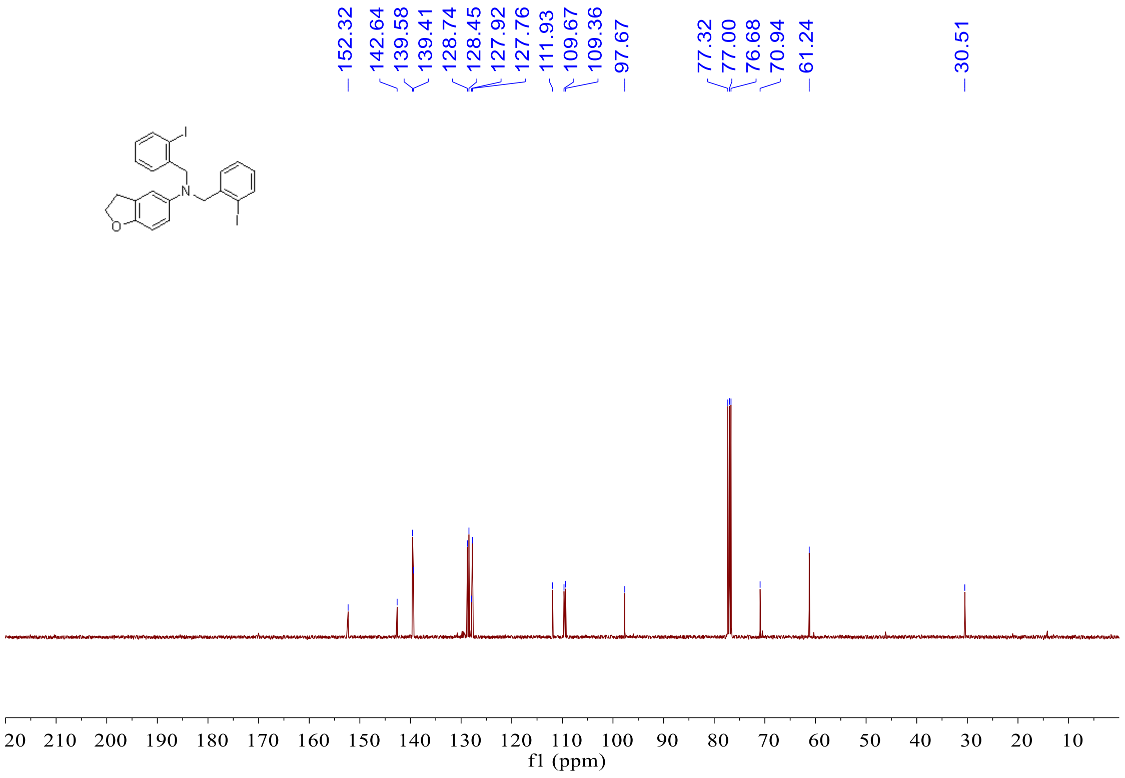

Supplementary Figure 160 ^{13}C NMR spectrum for compound **61b**

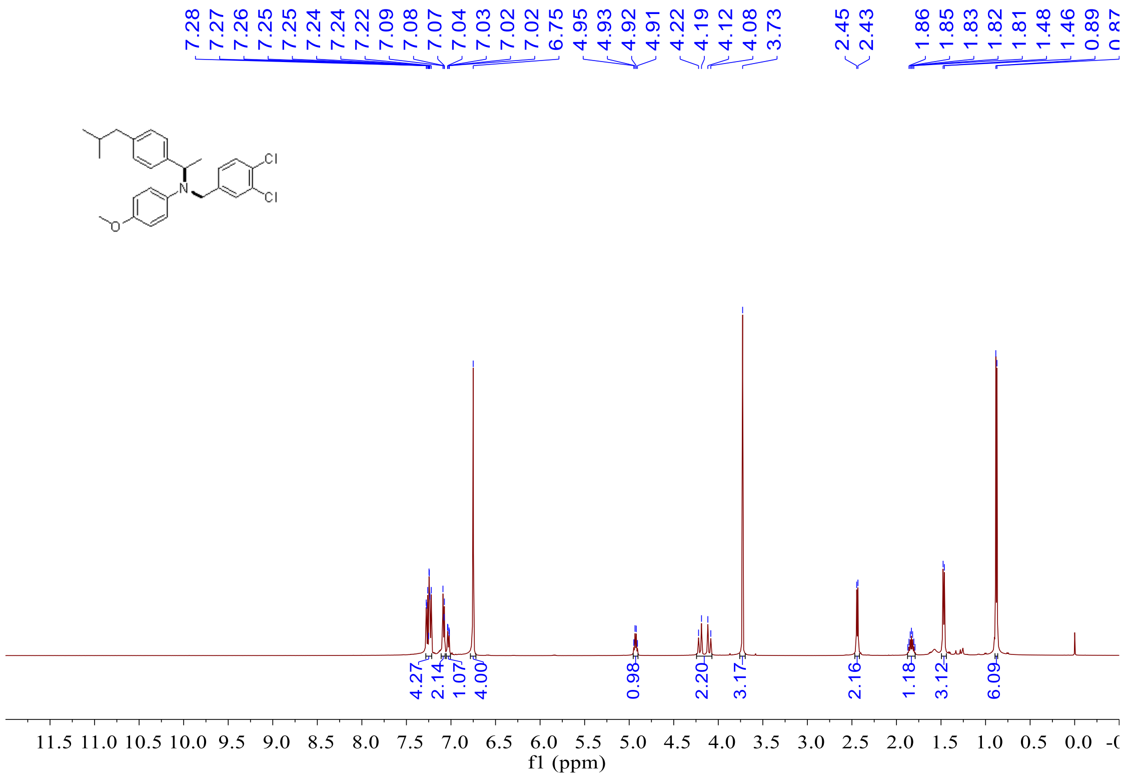

Supplementary Figure 161 ^{1}H NMR spectrum for compound **62**

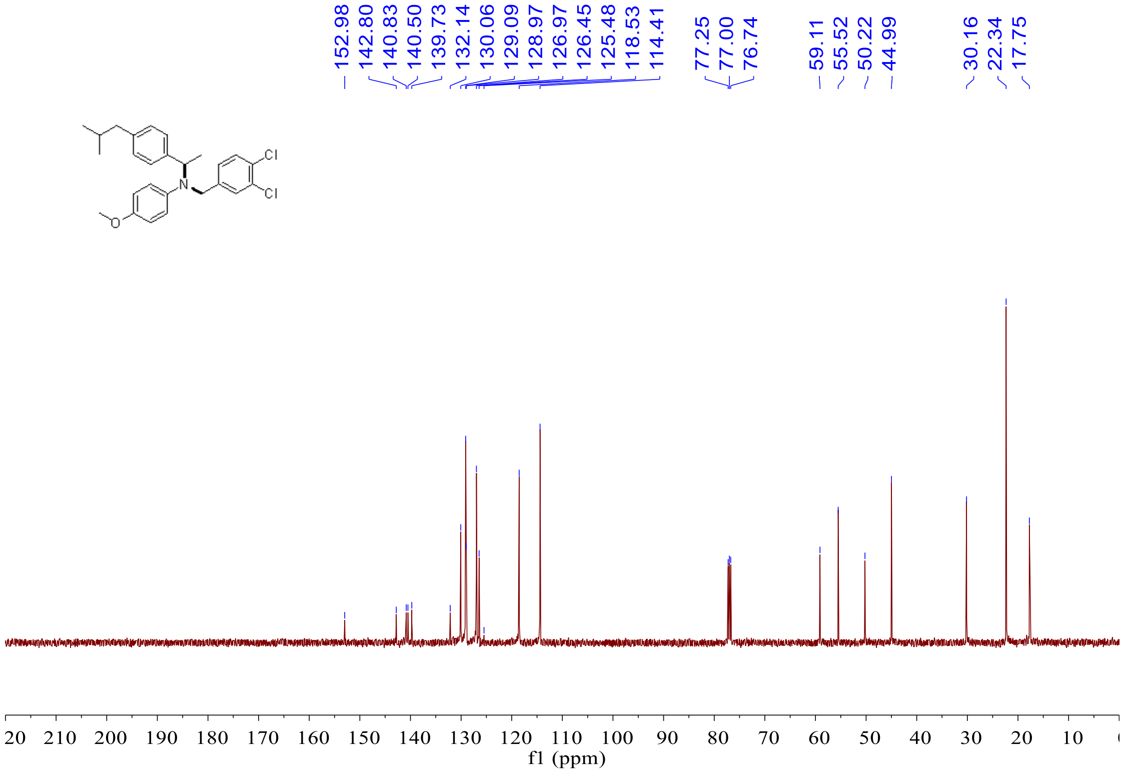

Supplementary Figure 162 ^{13}C NMR spectrum for compound **62**

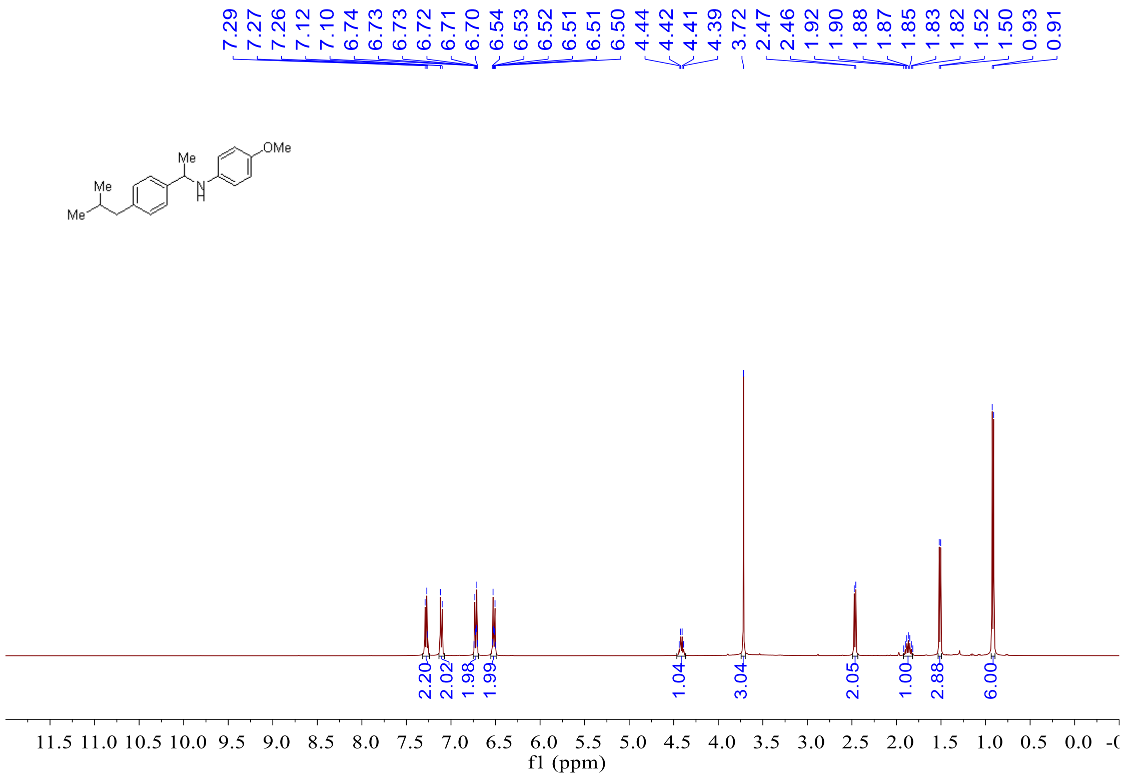

Supplementary Figure 163 ^{1}H NMR spectrum for compound **62a**

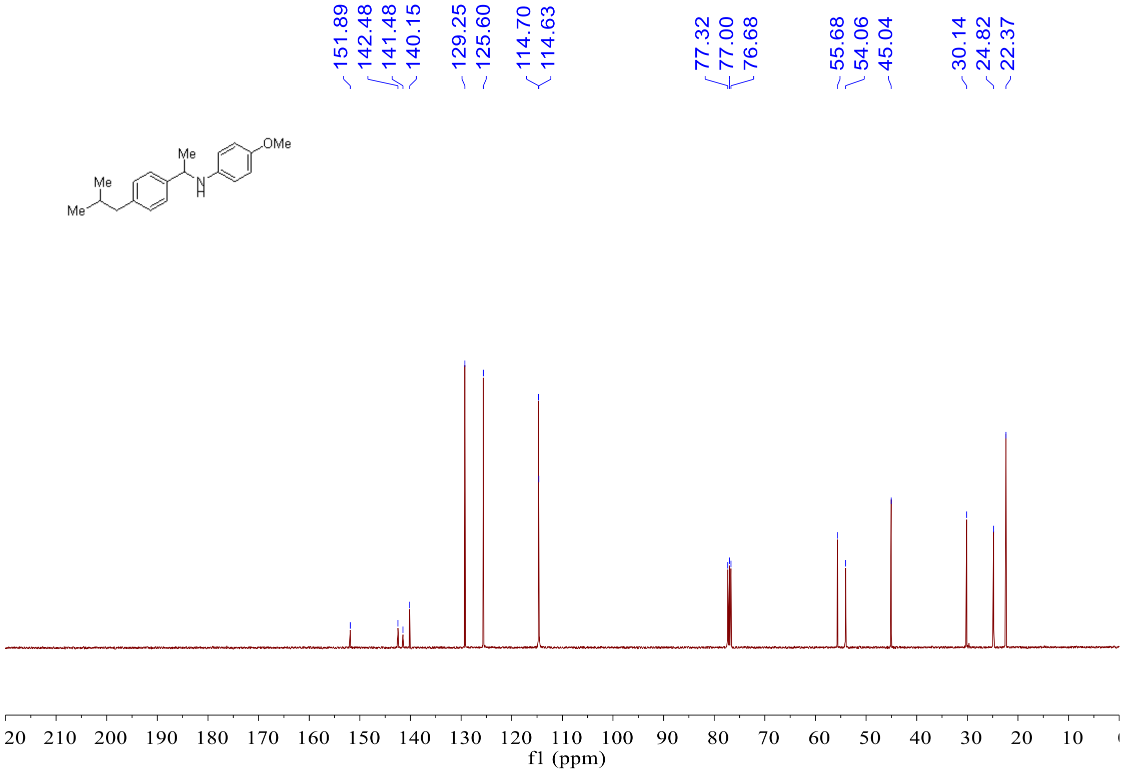

Supplementary Figure 164 ^{13}C NMR spectrum for compound **62a**

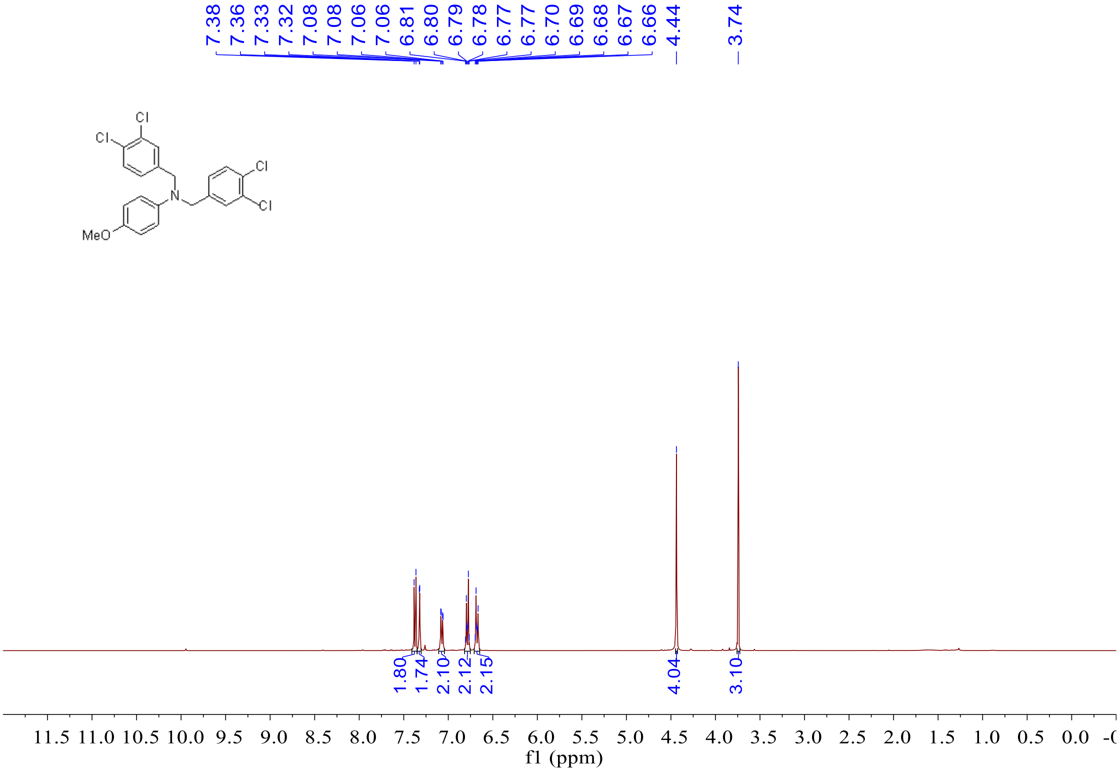

Supplementary Figure 165 ^{1}H NMR spectrum for compound **62b**

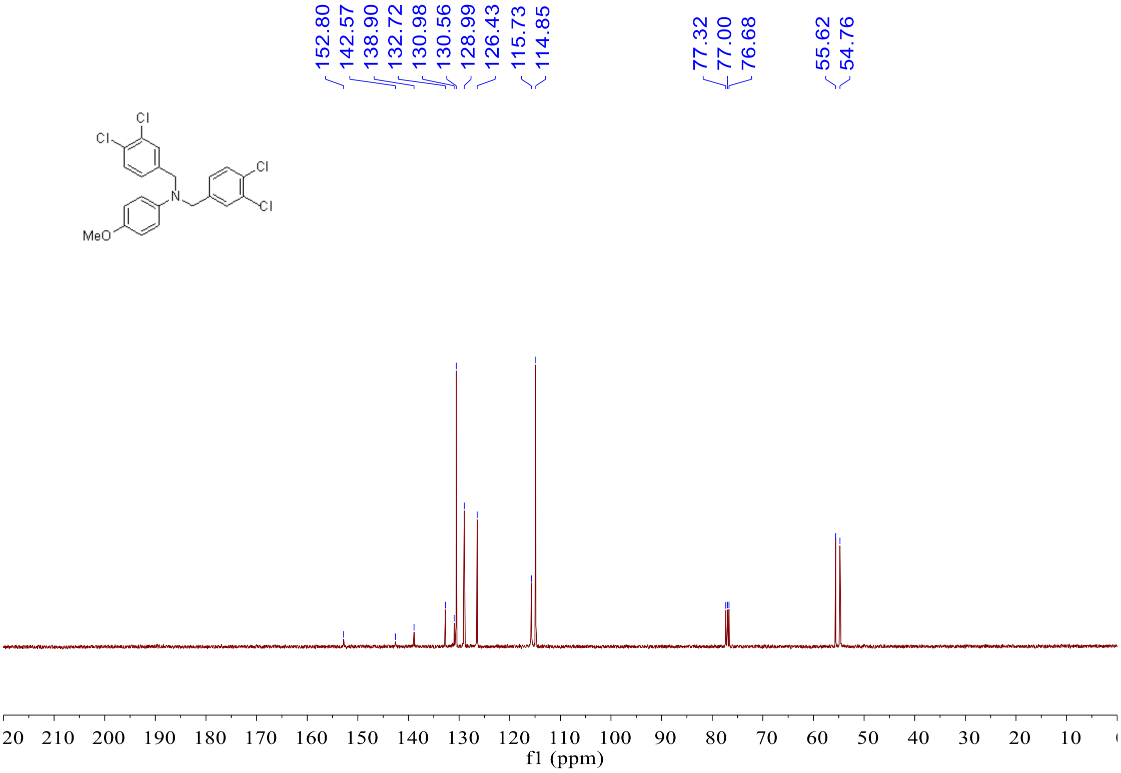

Supplementary Figure 166 ^{13}C NMR spectrum for compound **62b**

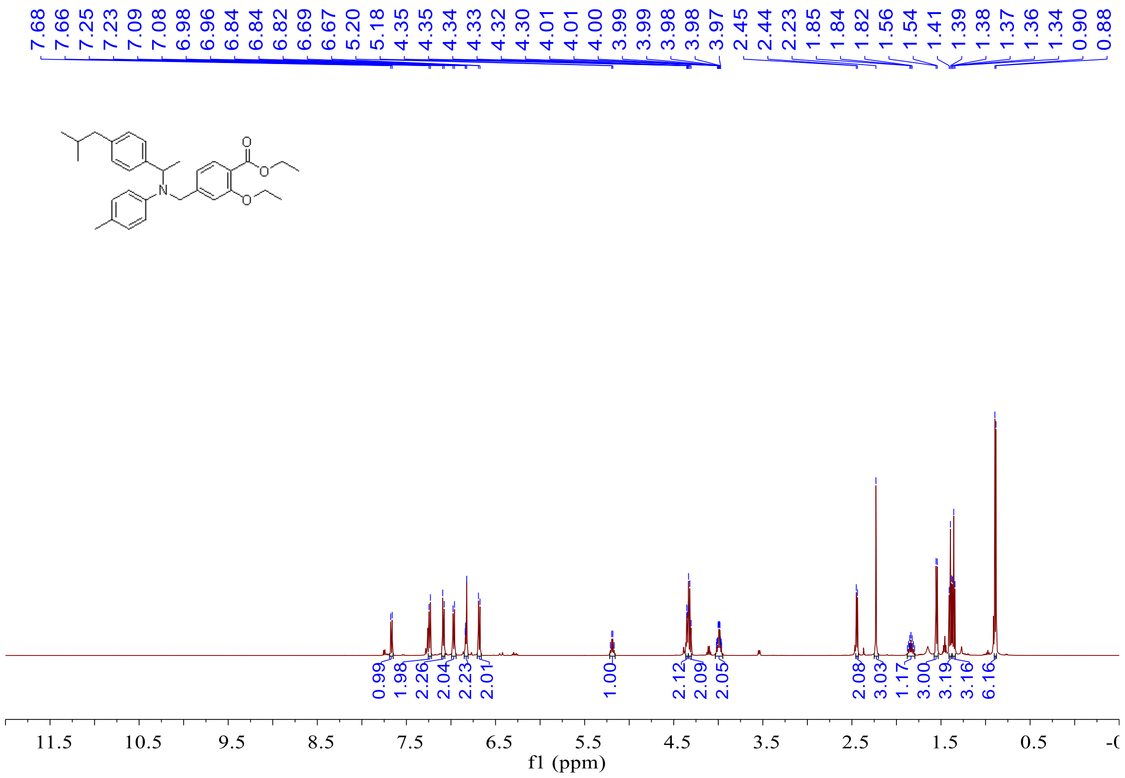

Supplementary Figure 167 ^{1}H NMR spectrum for compound **63**

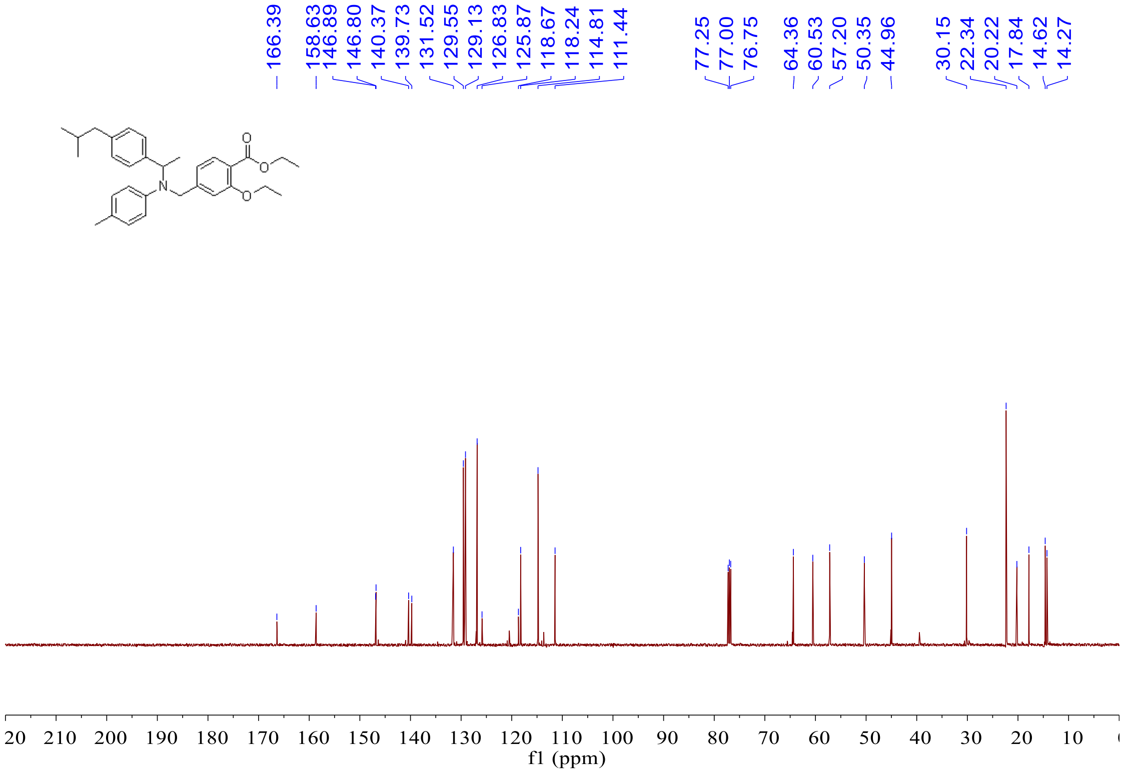

Supplementary Figure 168 ^{13}C NMR spectrum for compound **63**

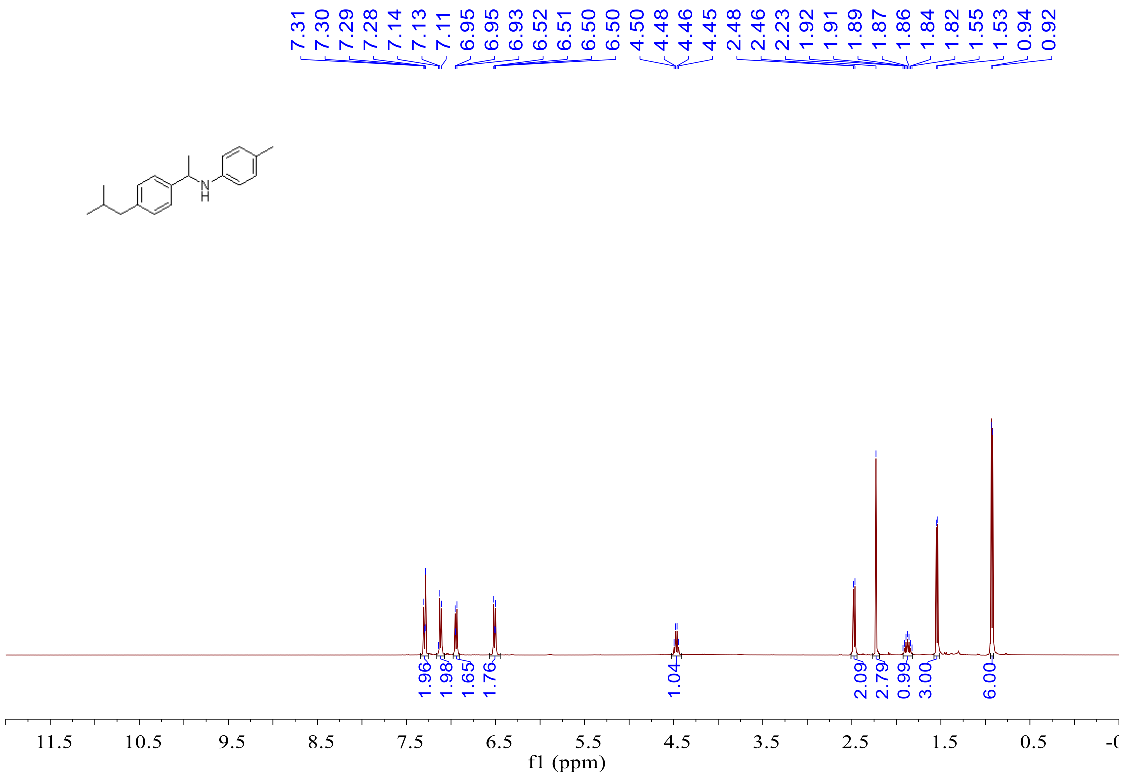

Supplementary Figure 169 ^{1}H NMR spectrum for compound **63a**

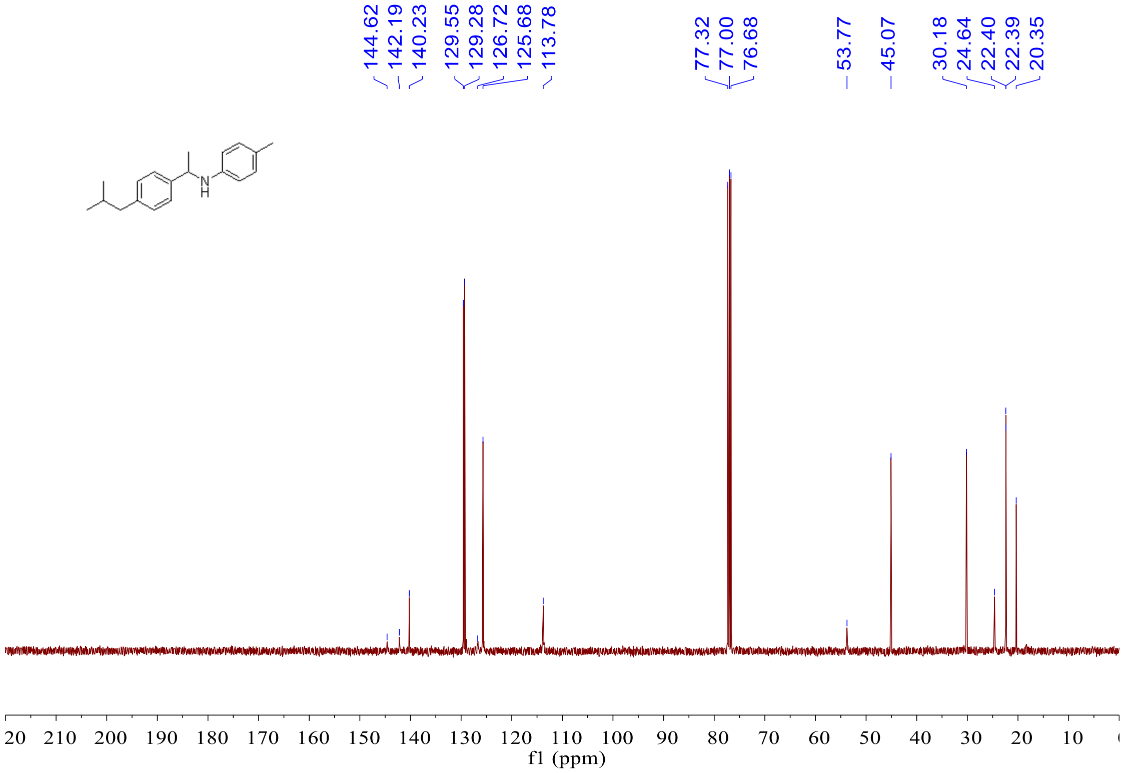

Supplementary Figure 170 ^{13}C NMR spectrum for compound **63a**

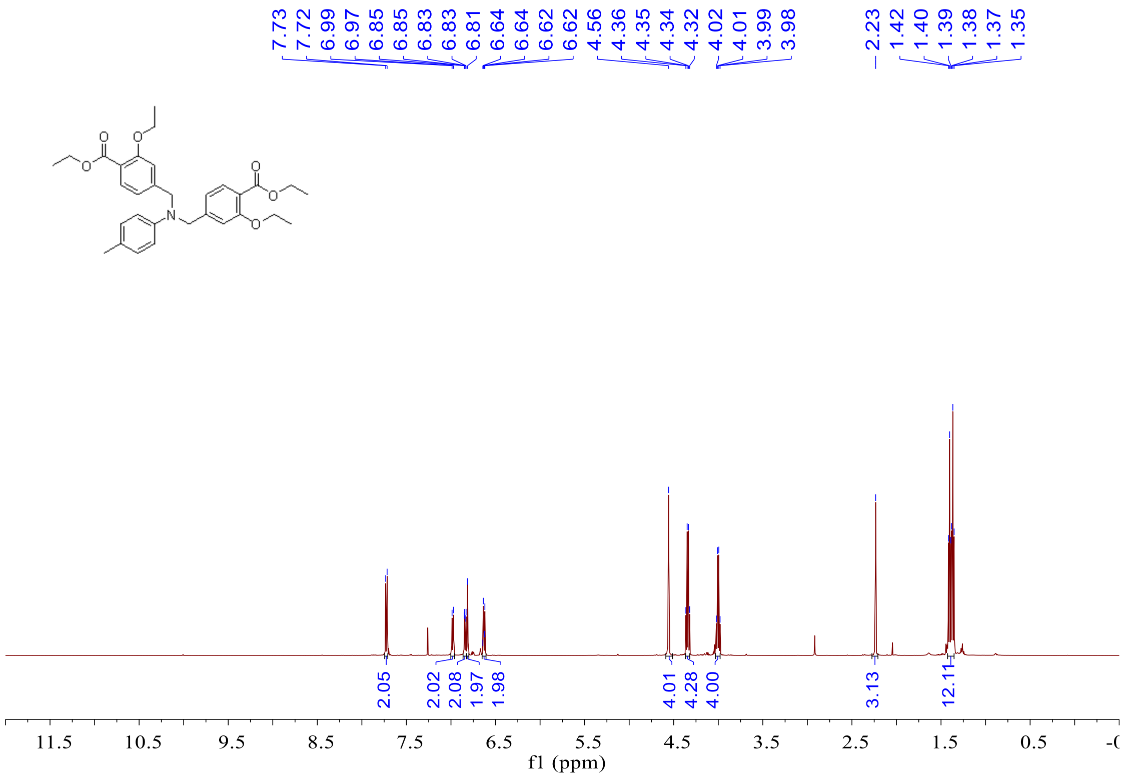

Supplementary Figure 171 ^{1}H NMR spectrum for compound **63b**

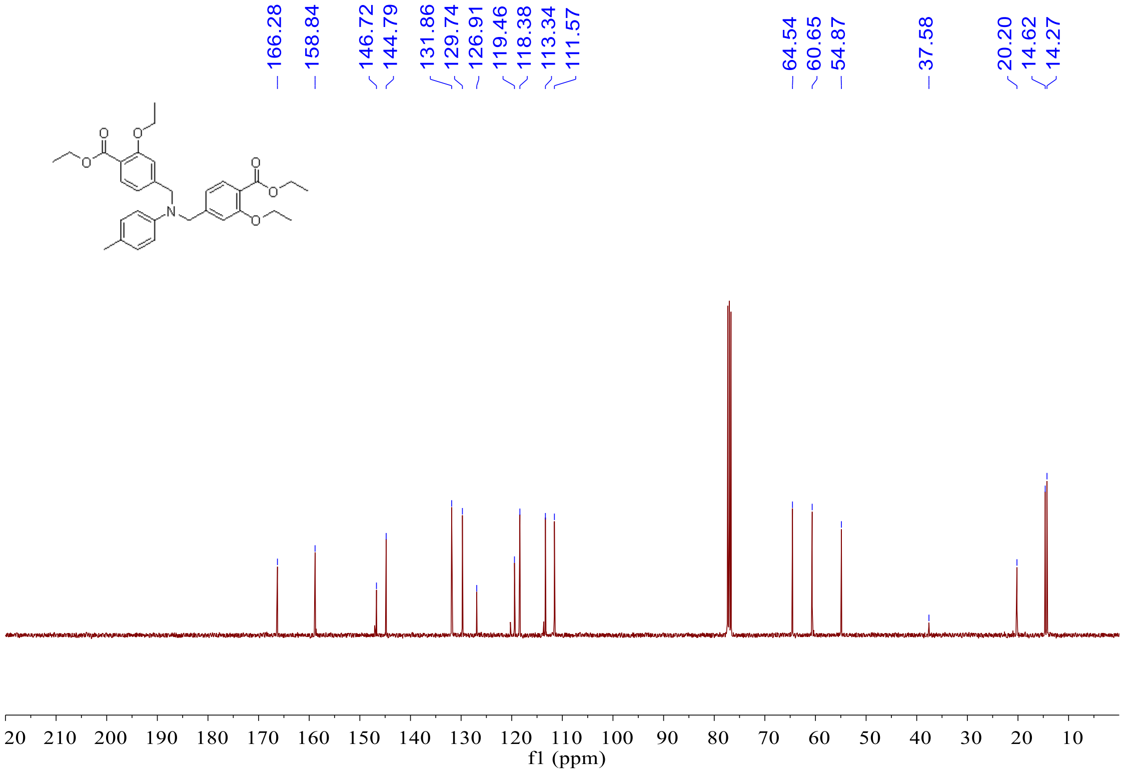

Supplementary Figure 172 ^{13}C NMR spectrum for compound **63b**

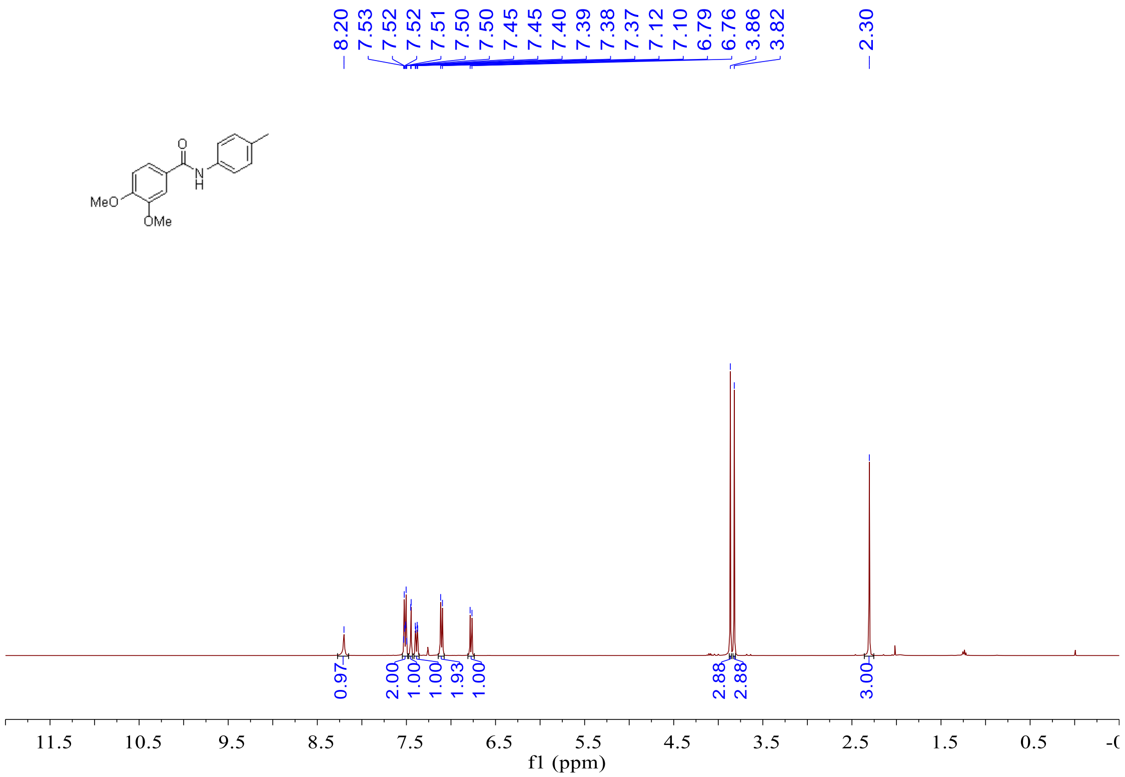

Supplementary Figure 173 ^{1}H NMR spectrum for compound **64**

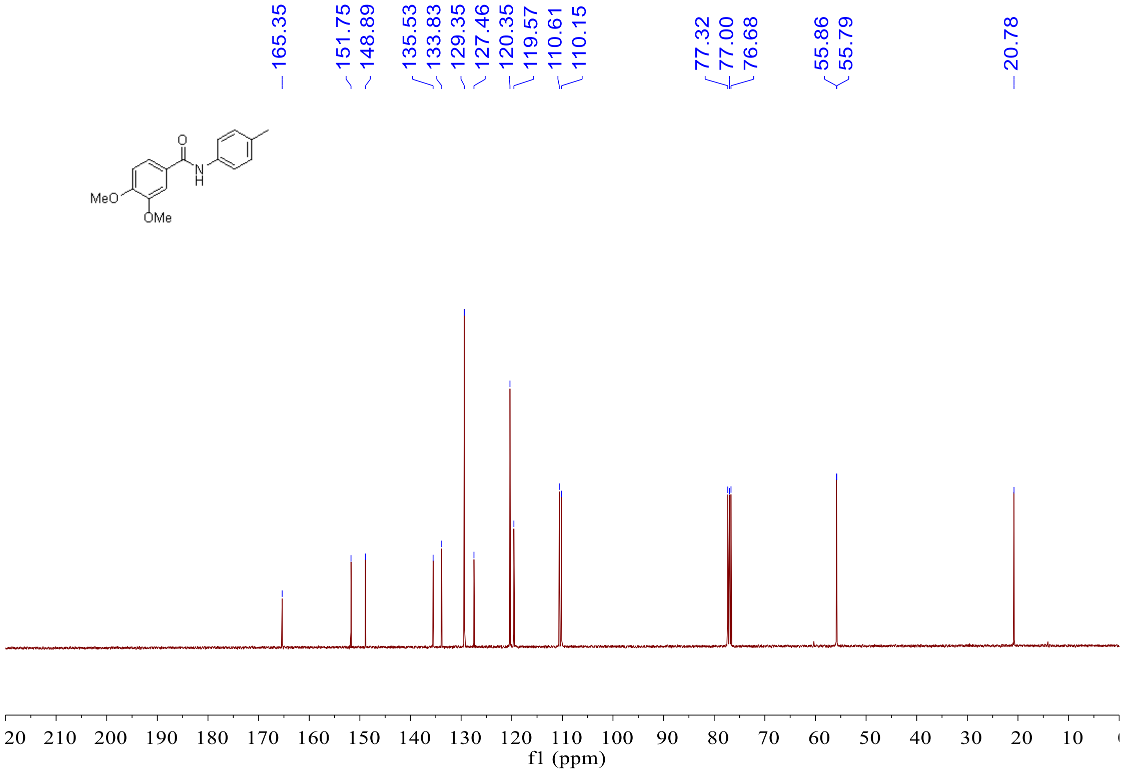

Supplementary Figure 174 ^{13}C NMR spectrum for compound **64**

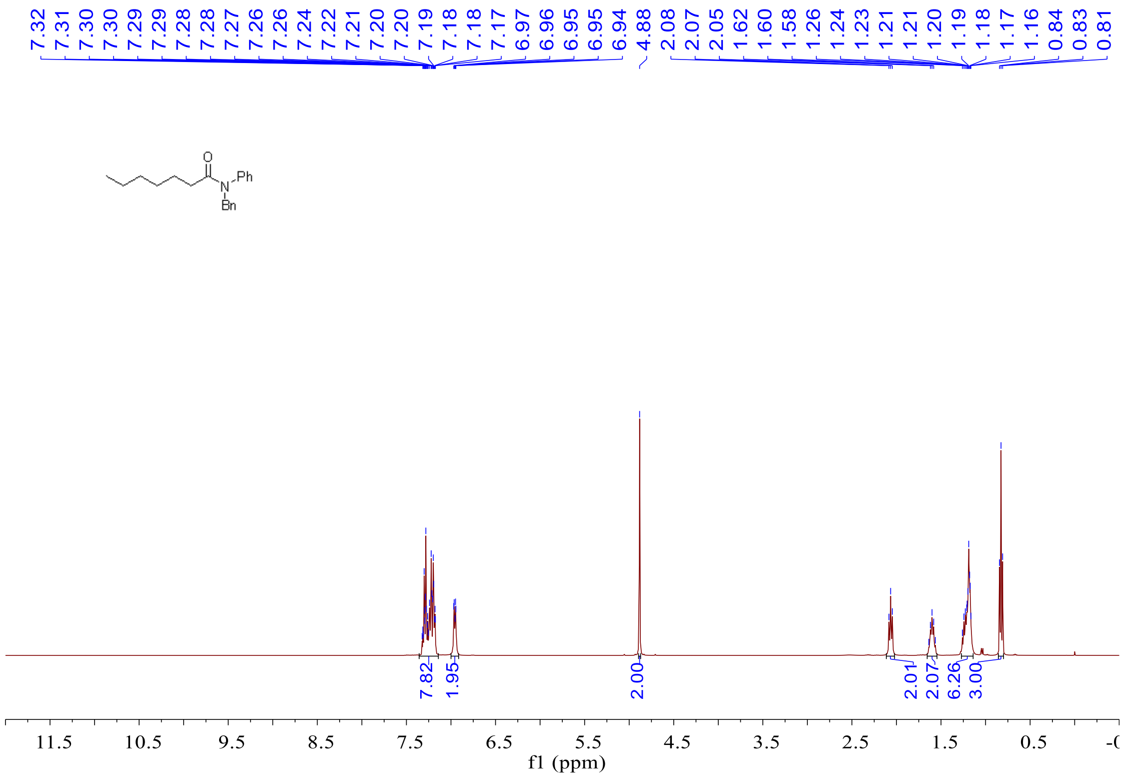

Supplementary Figure 175 ^{1}H NMR spectrum for compound **65**

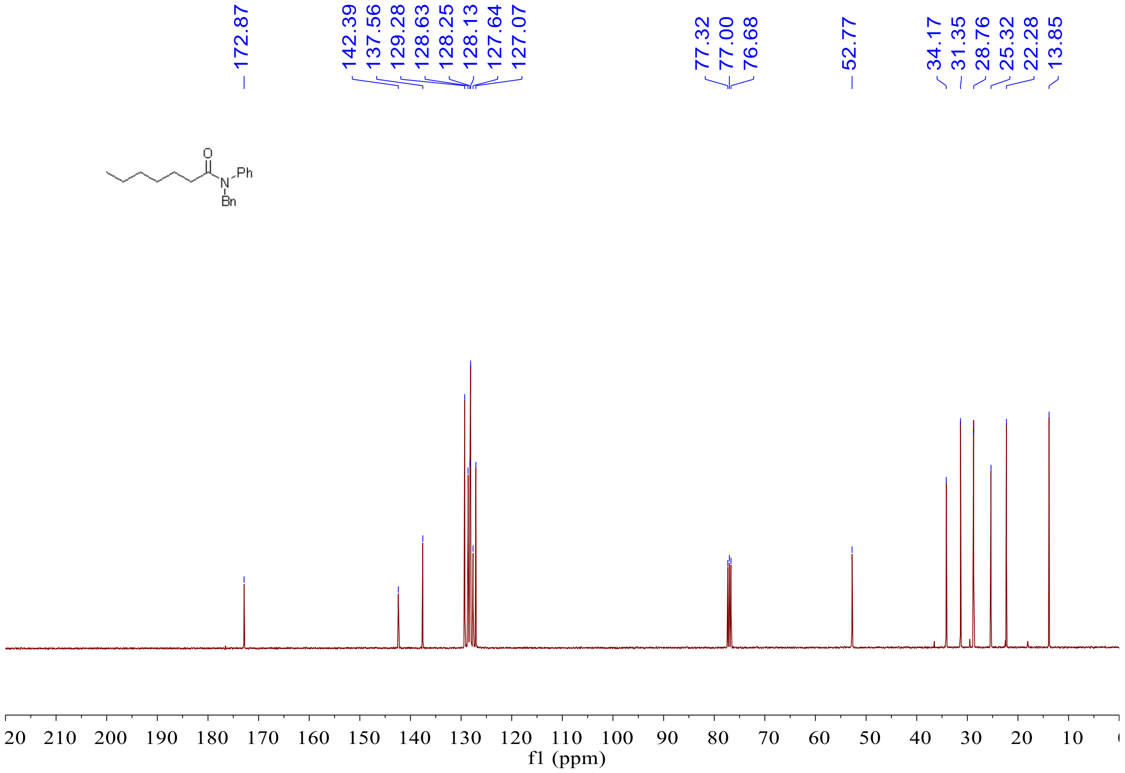

Supplementary Figure 176 ^{13}C NMR spectrum for compound **65**

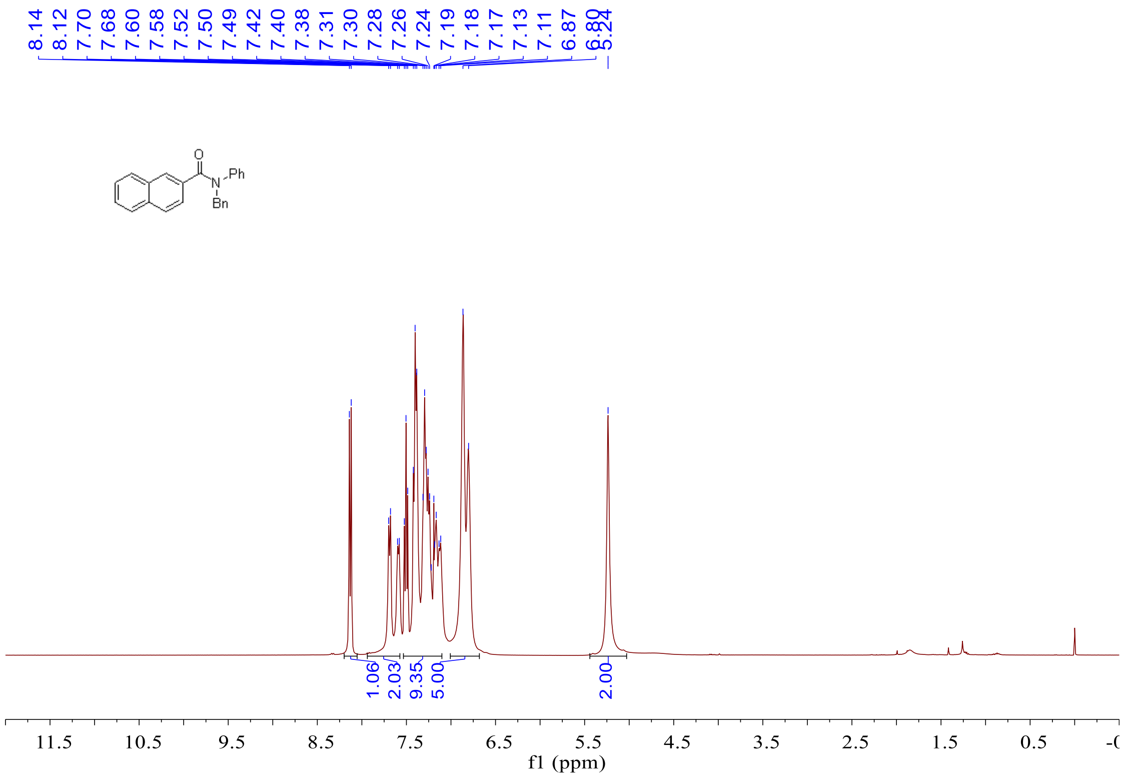

Supplementary Figure 177 ^{1}H NMR spectrum for compound **66**

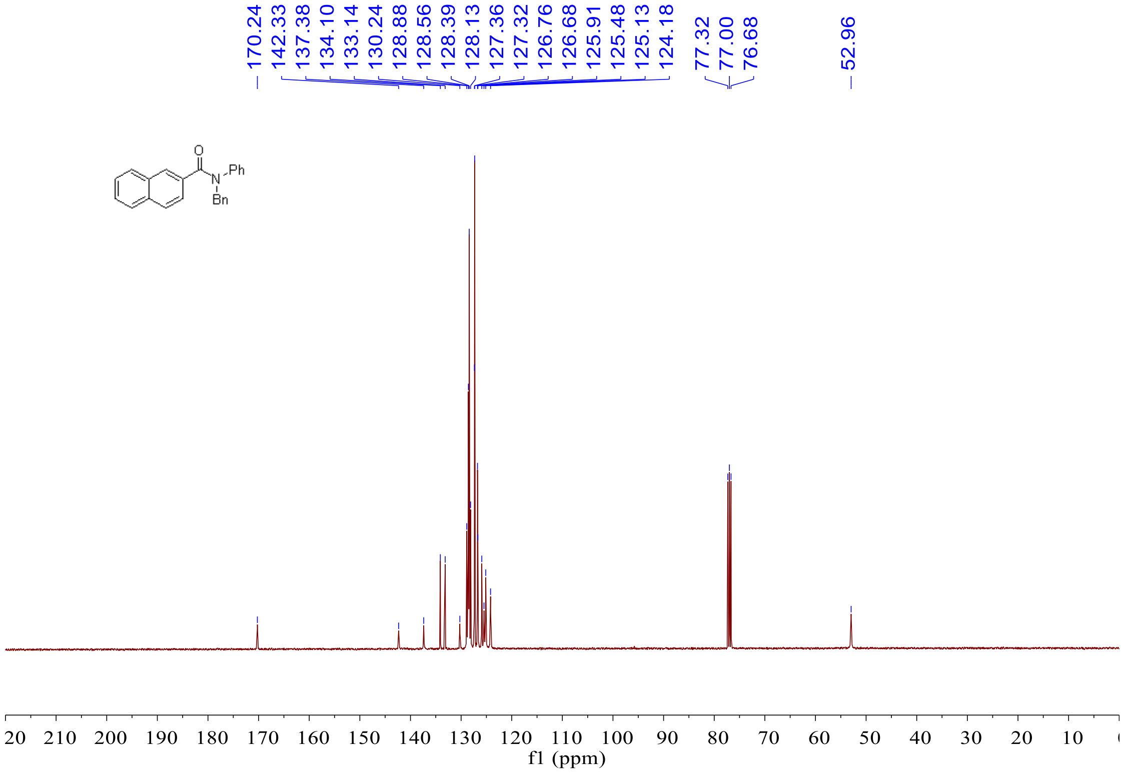

Supplementary Figure 178 ^{13}C NMR spectrum for compound **66**

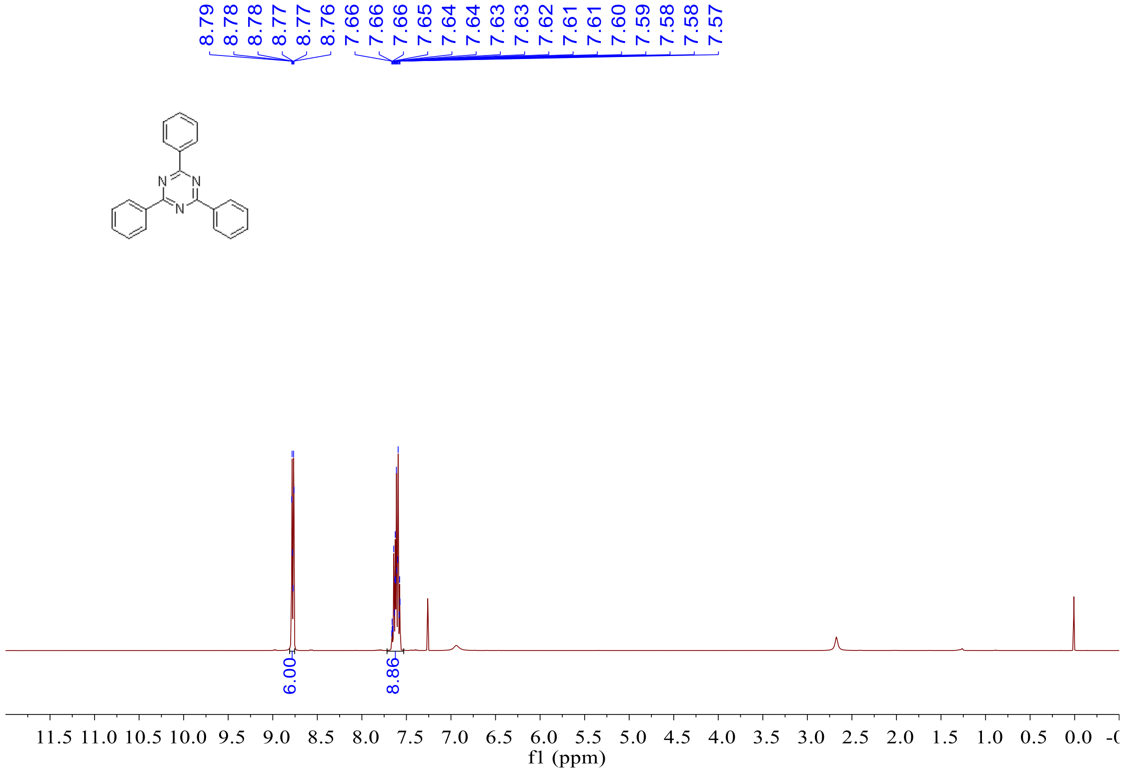

Supplementary Figure 179 ^{1}H NMR spectrum for compound **67**

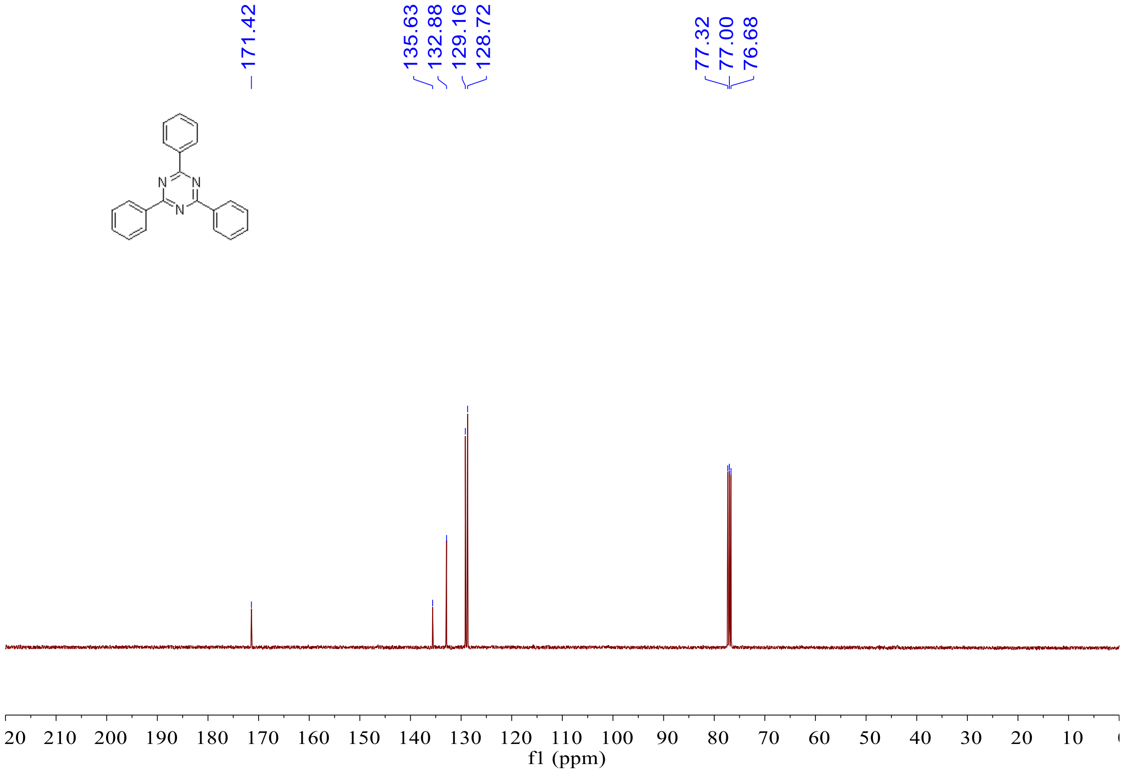

Supplementary Figure 180 ^{13}C NMR spectrum for compound **67**

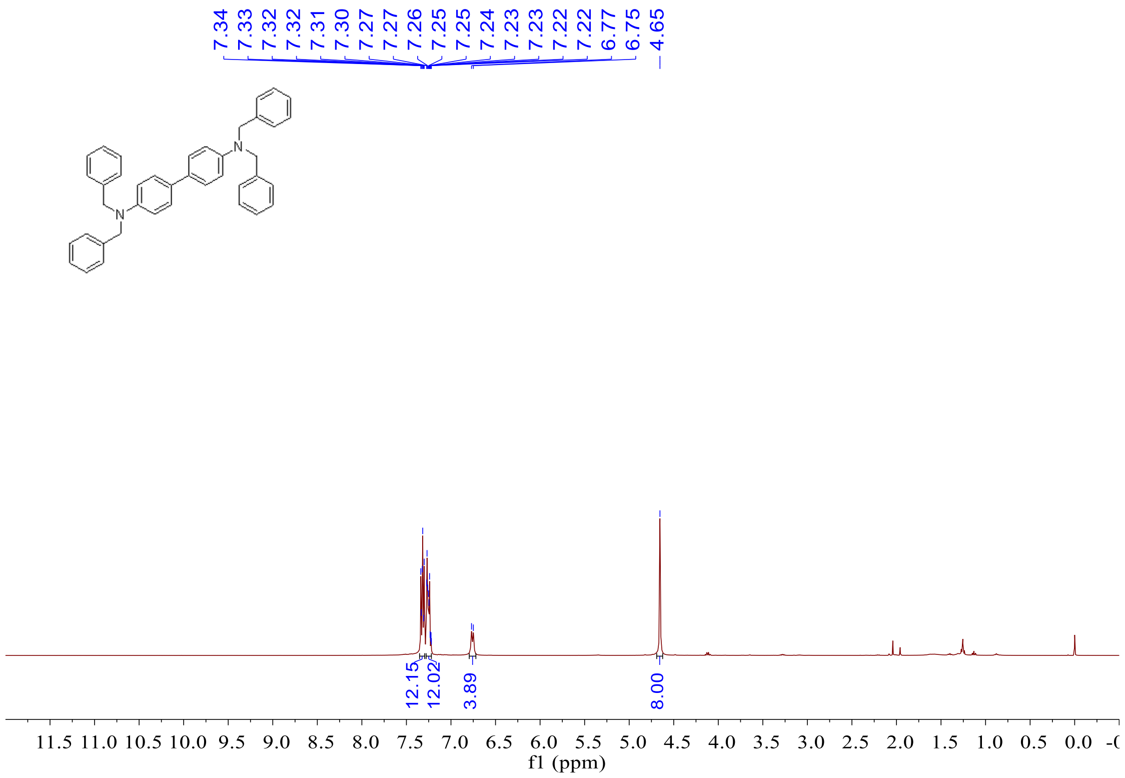

Supplementary Figure 181 ^{1}H NMR spectrum for compound **68**

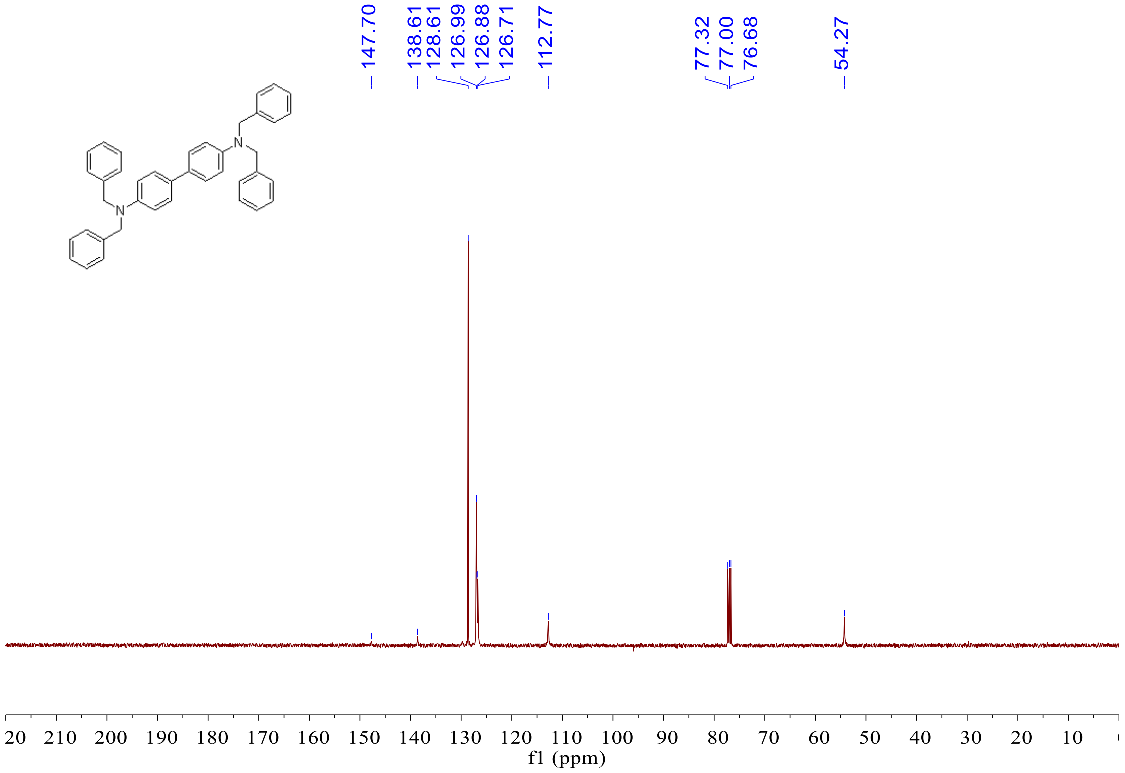

Supplementary Figure 182 ^{13}C NMR spectrum for compound **68**

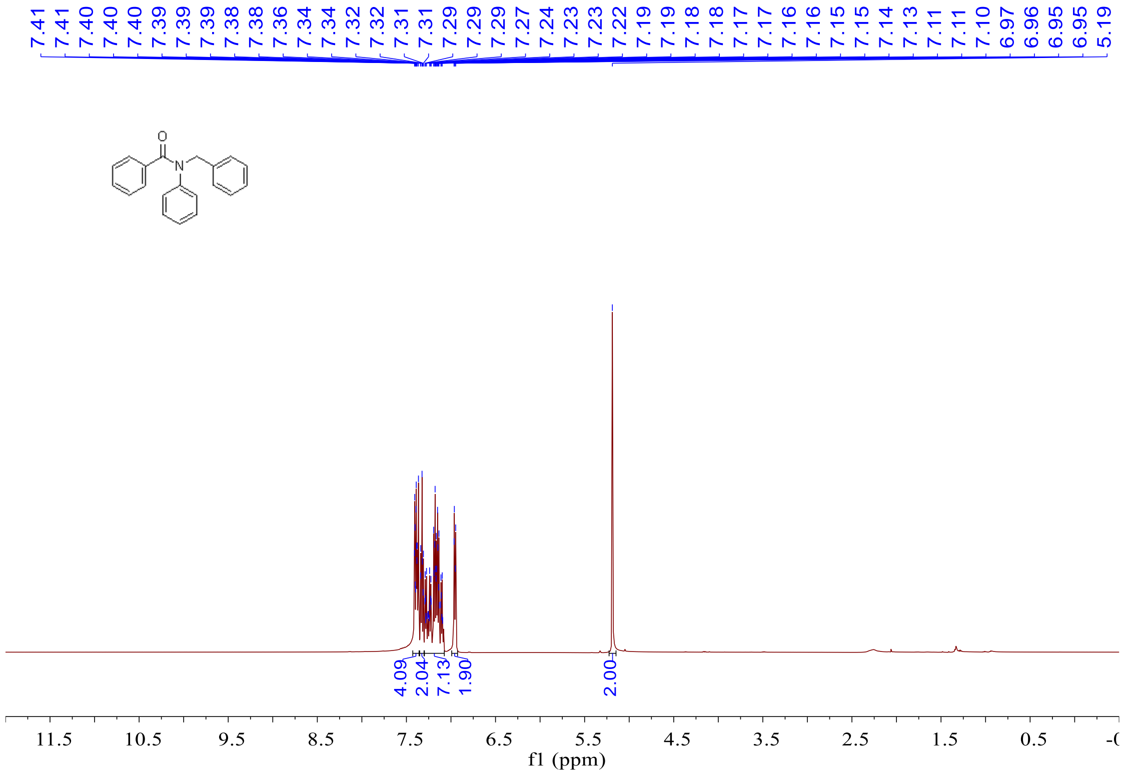

Supplementary Figure 183 ^{1}H NMR spectrum for compound **69**

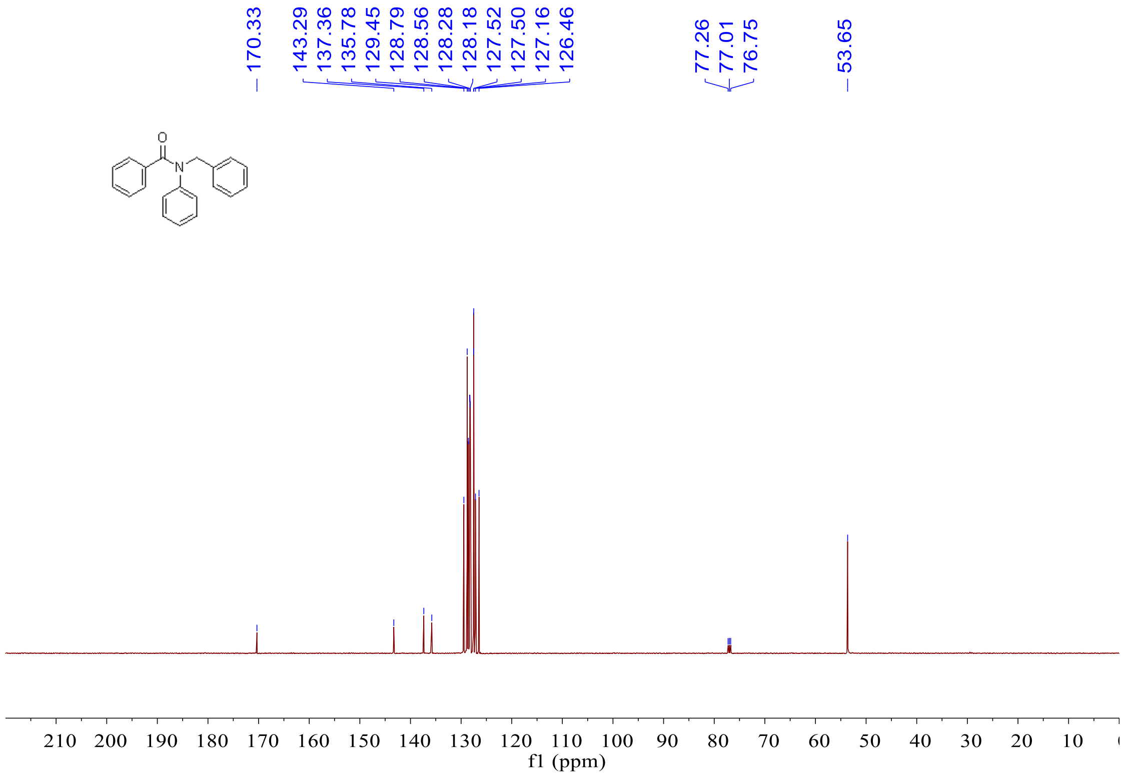

Supplementary Figure 184 ^{13}C NMR spectrum for compound **69**

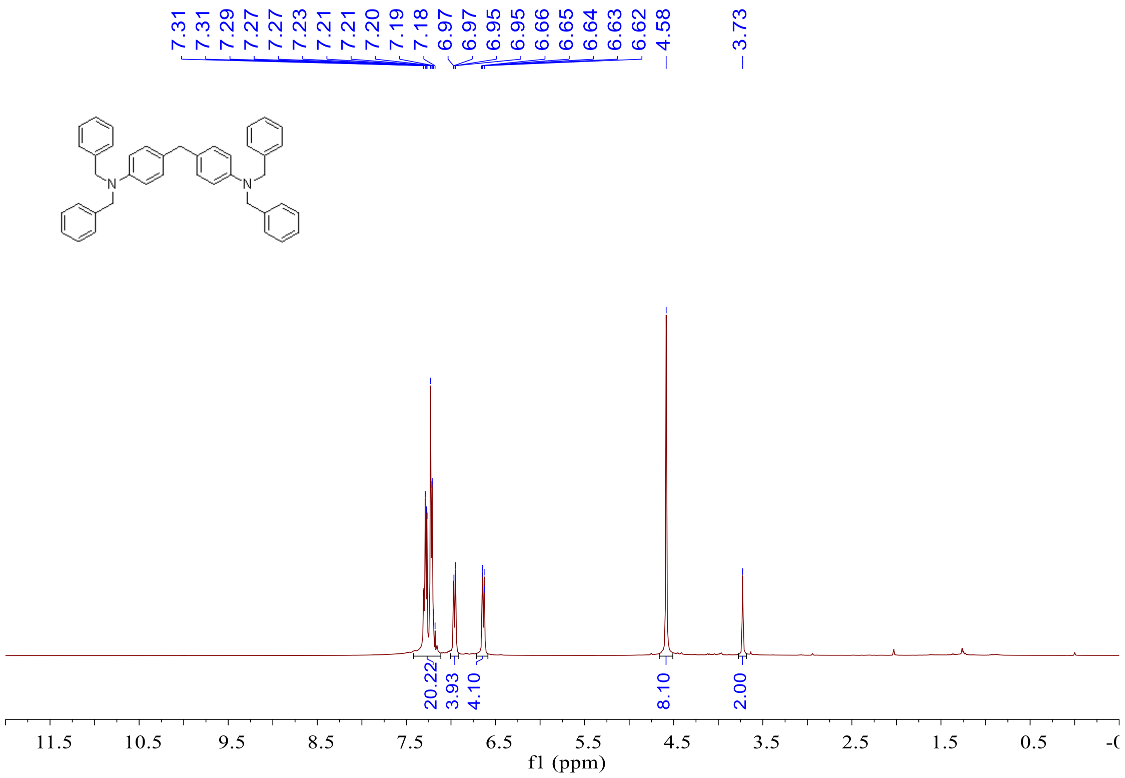

Supplementary Figure 185 ^{1}H NMR spectrum for compound **70**

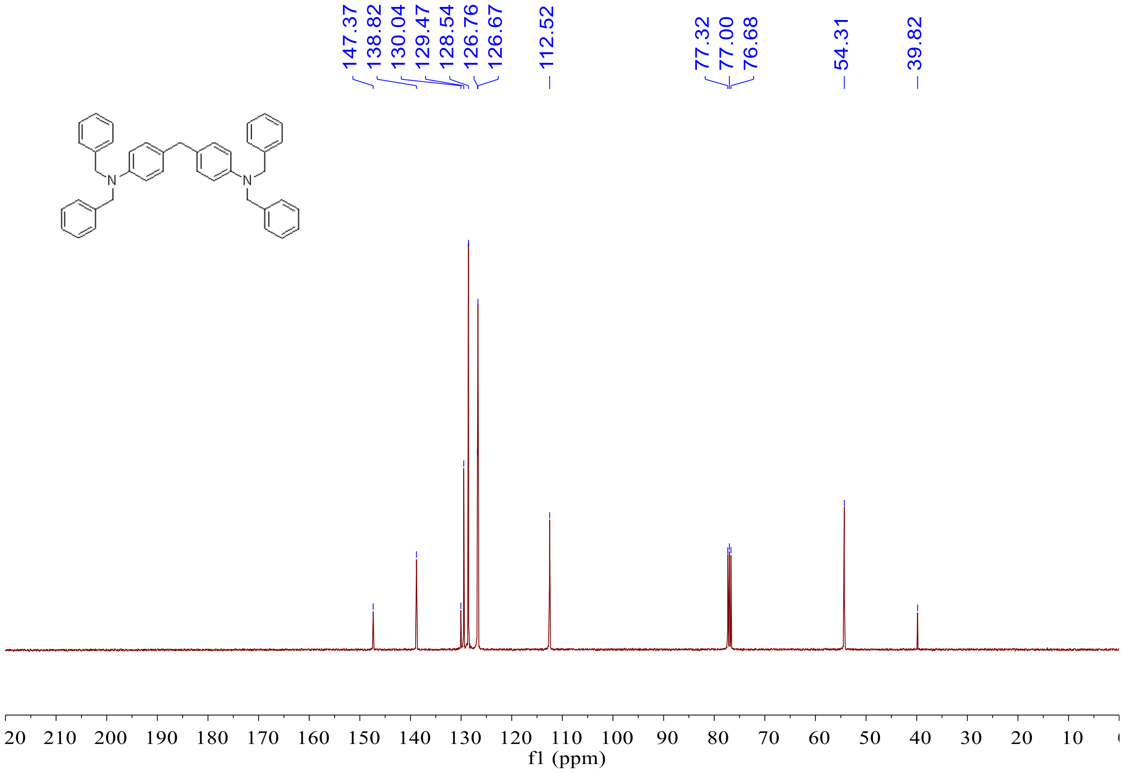

Supplementary Figure 186 ^{13}C NMR spectrum for compound **70**

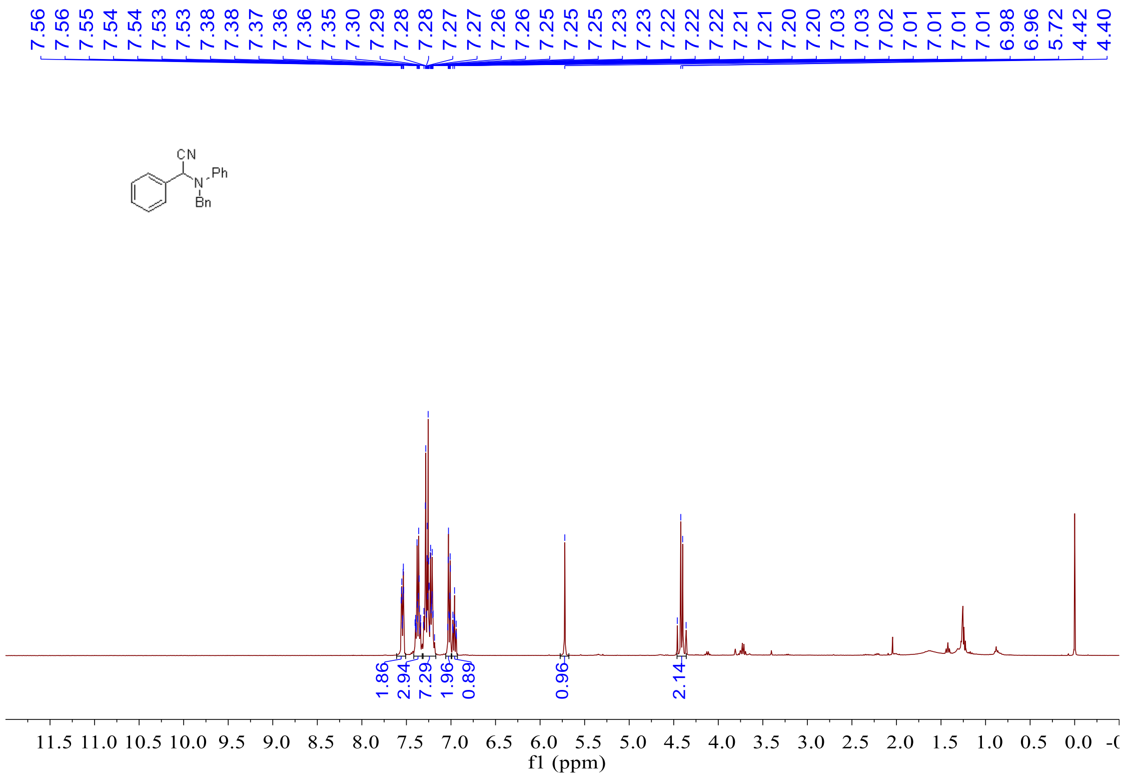

Supplementary Figure 187 ^{1}H NMR spectrum for compound **71**

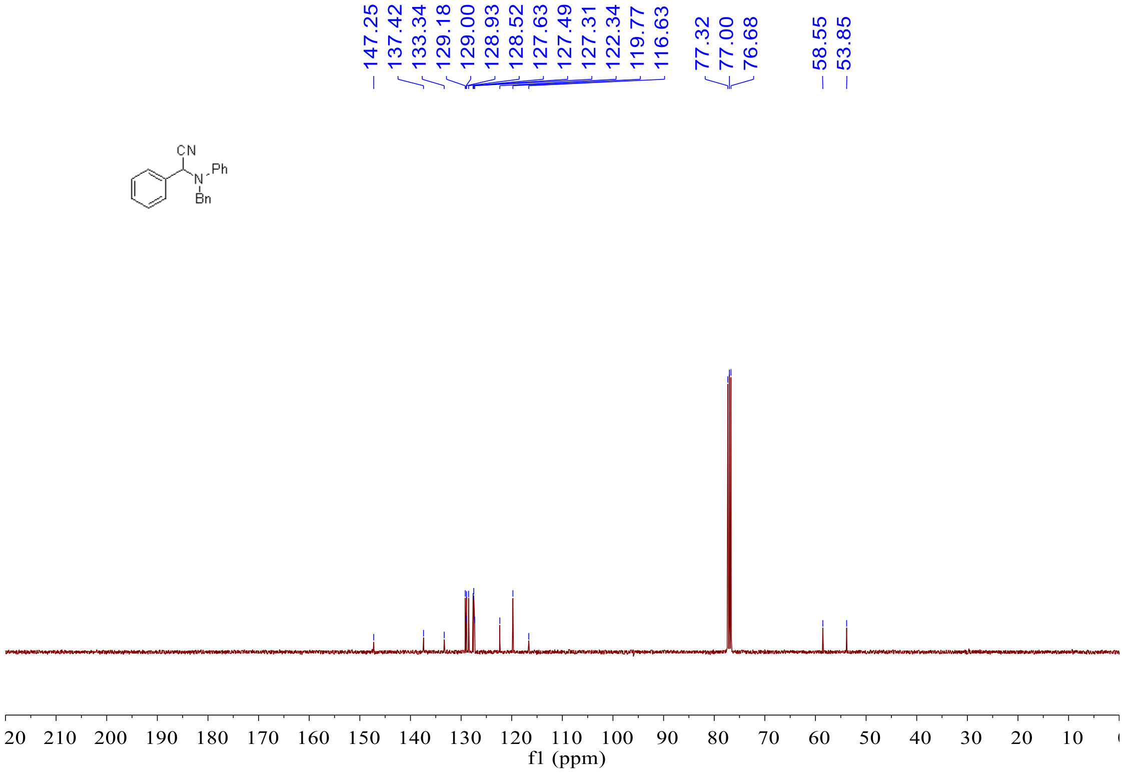

Supplementary Figure 188 ^{13}C NMR spectrum for compound **71**

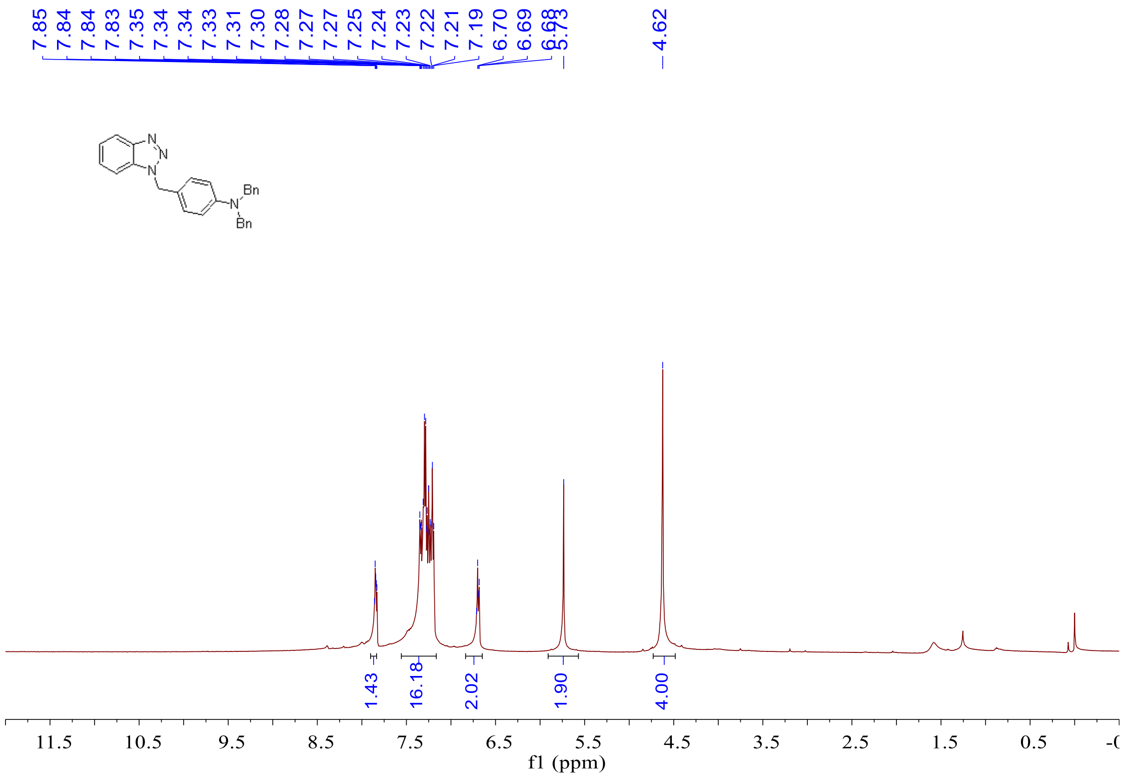

Supplementary Figure 189 ^{1}H NMR spectrum for compound **72**

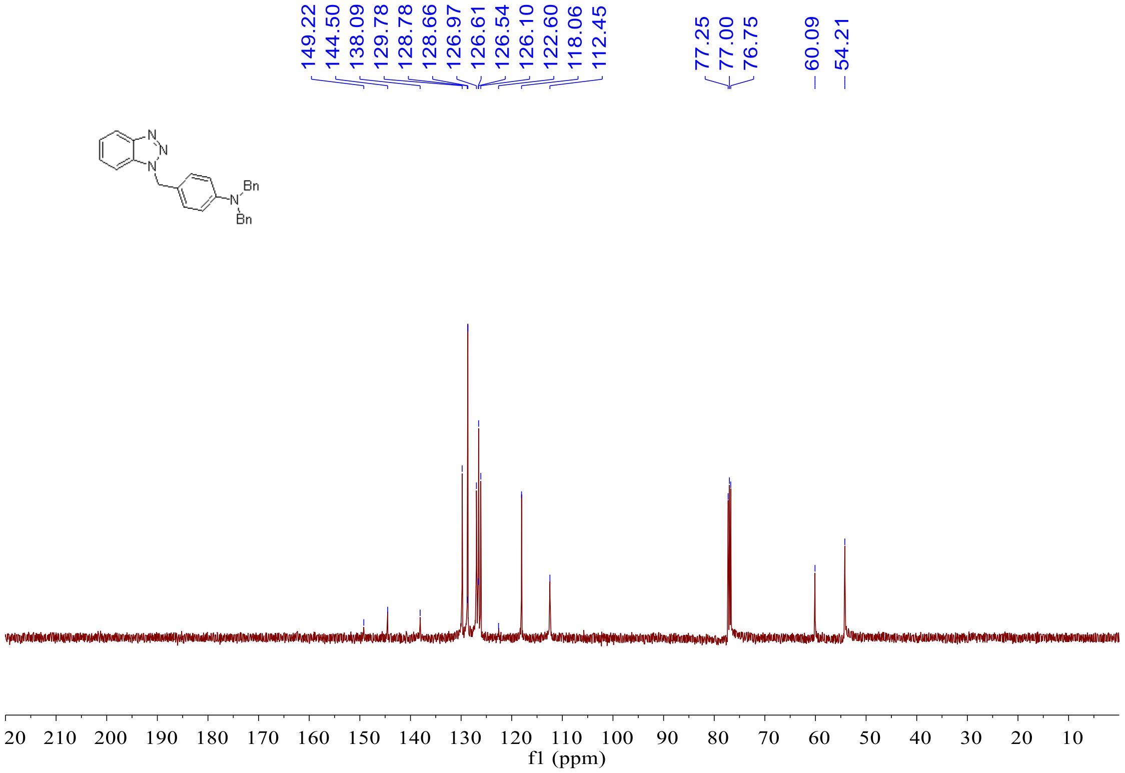

Supplementary Figure 190 ^{13}C NMR spectrum for compound **72**

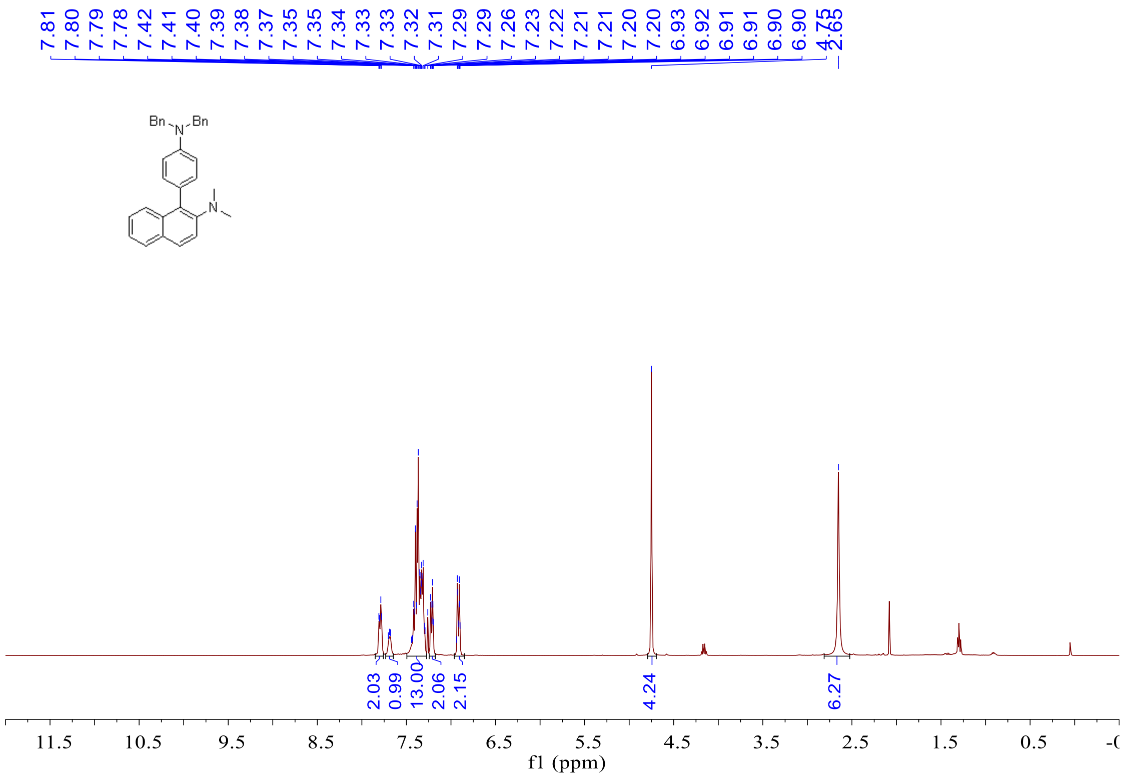

Supplementary Figure 191 ^{1}H NMR spectrum for compound **73**

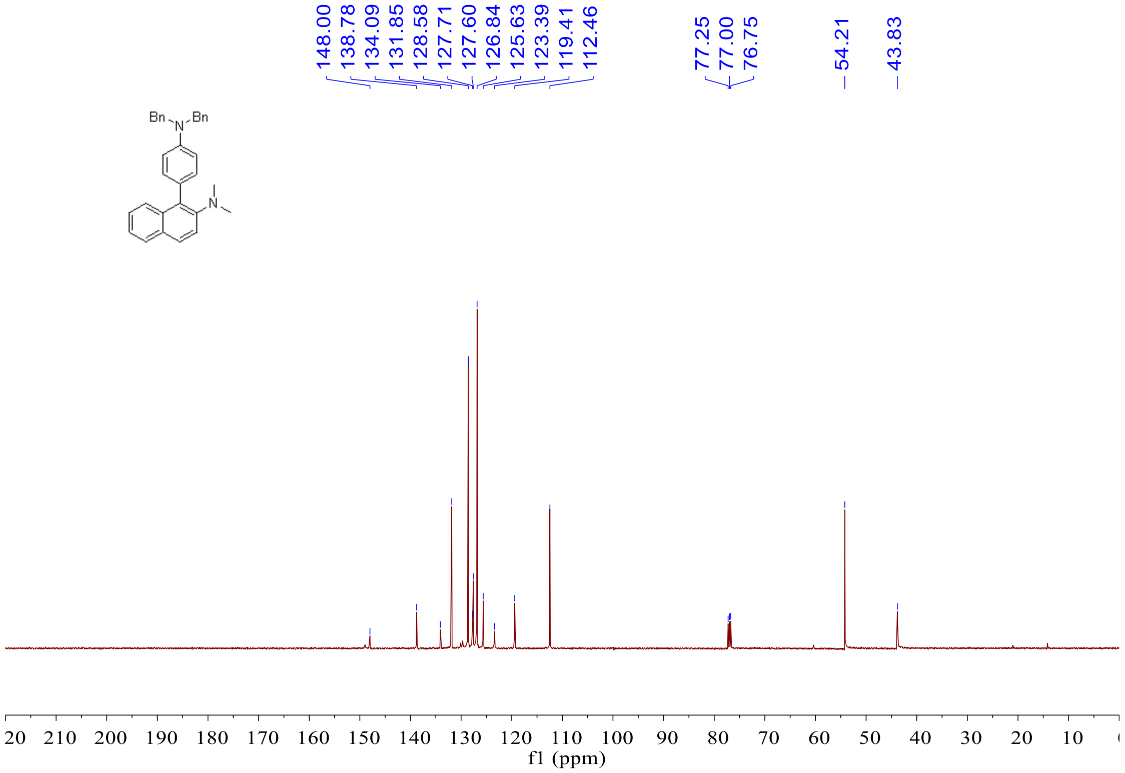

Supplementary Figure 192 ^{13}C NMR spectrum for compound **73**

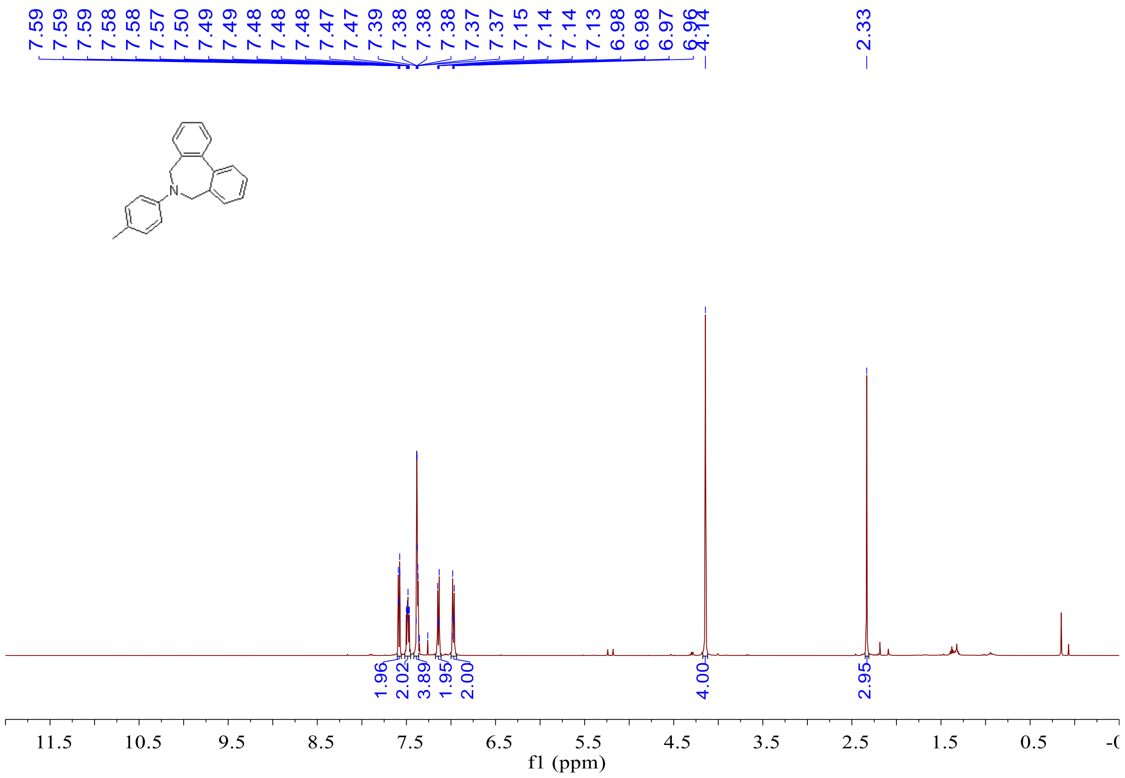

Supplementary Figure 193 ^{1}H NMR spectrum for compound **74**

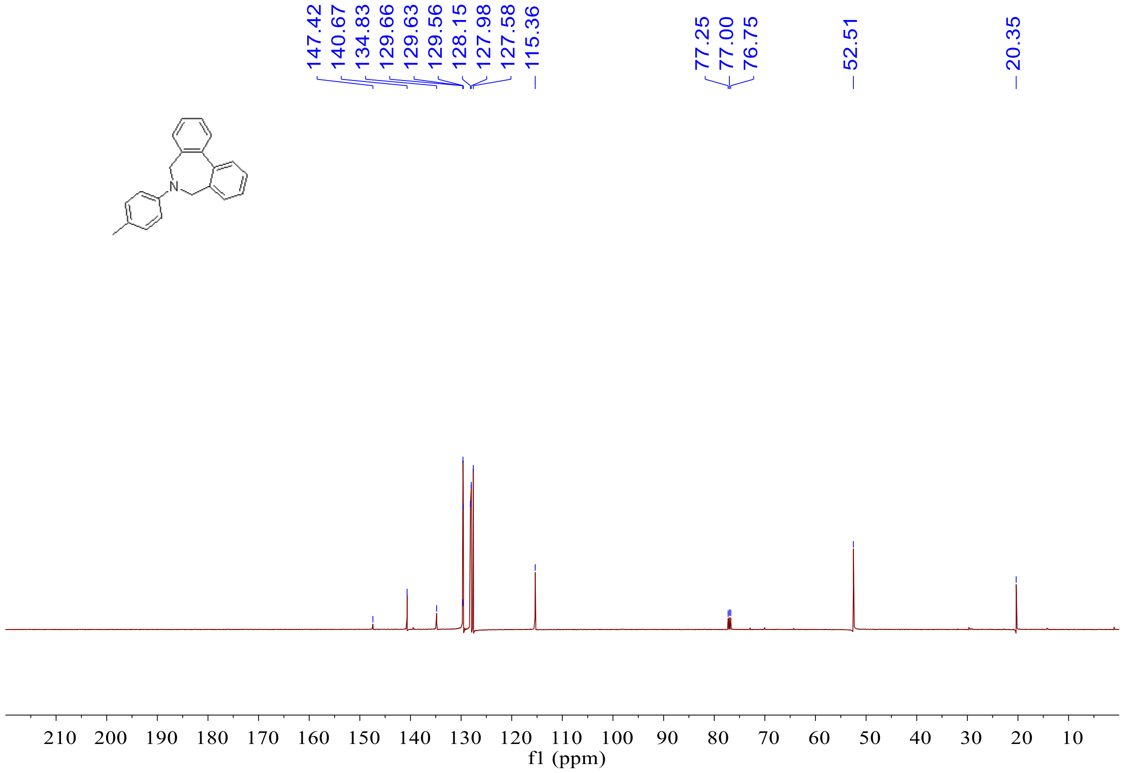

Supplementary Figure 194 ^{13}C NMR spectrum for compound **74**

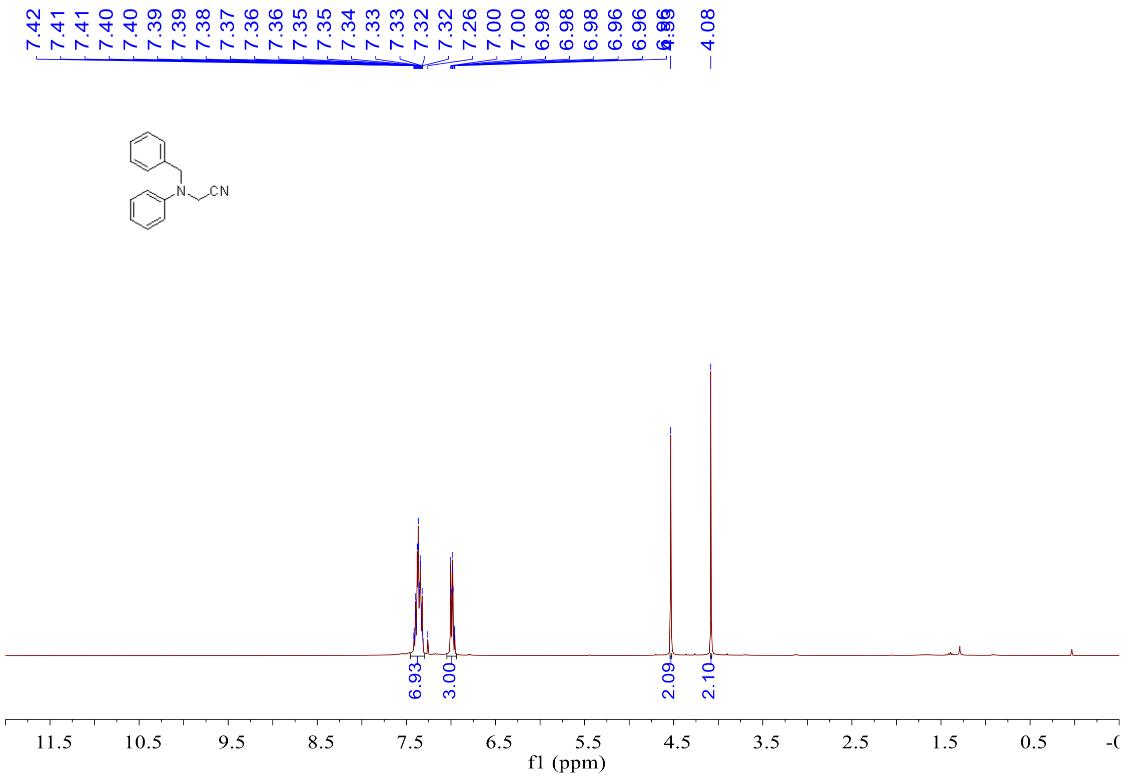

Supplementary Figure 195 ^{1}H NMR spectrum for compound **75**

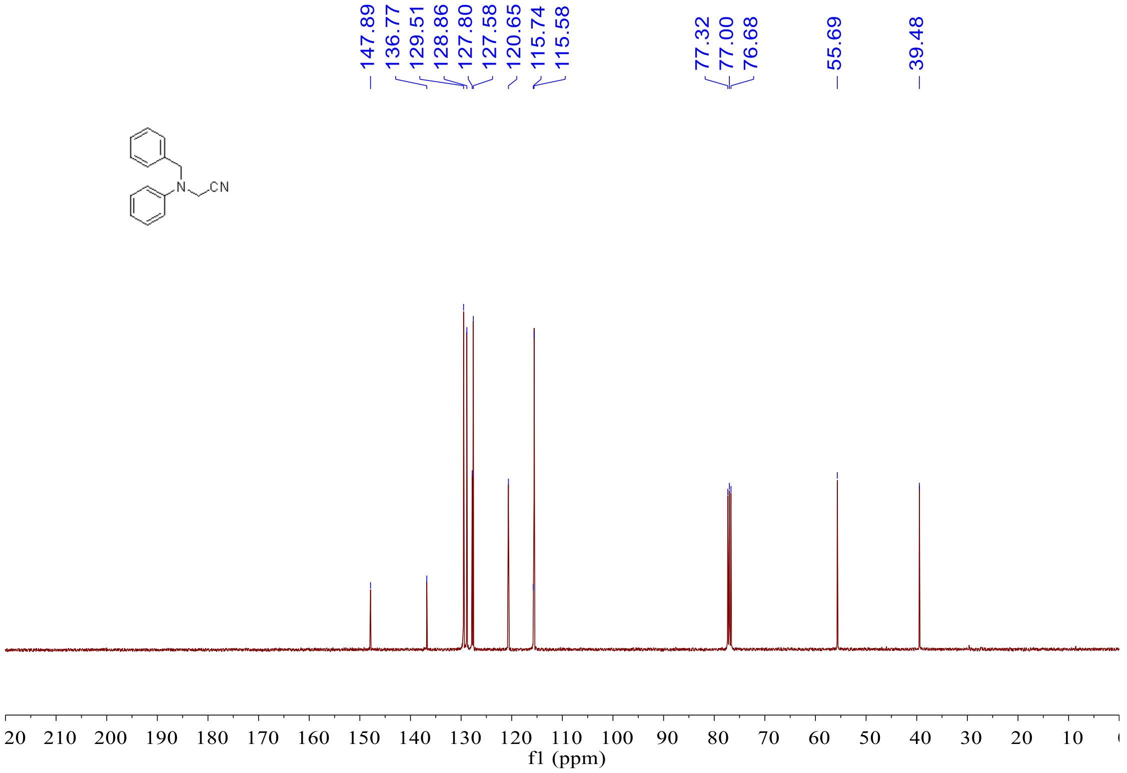

Supplementary Figure 196 ^{13}C NMR spectrum for compound **75**

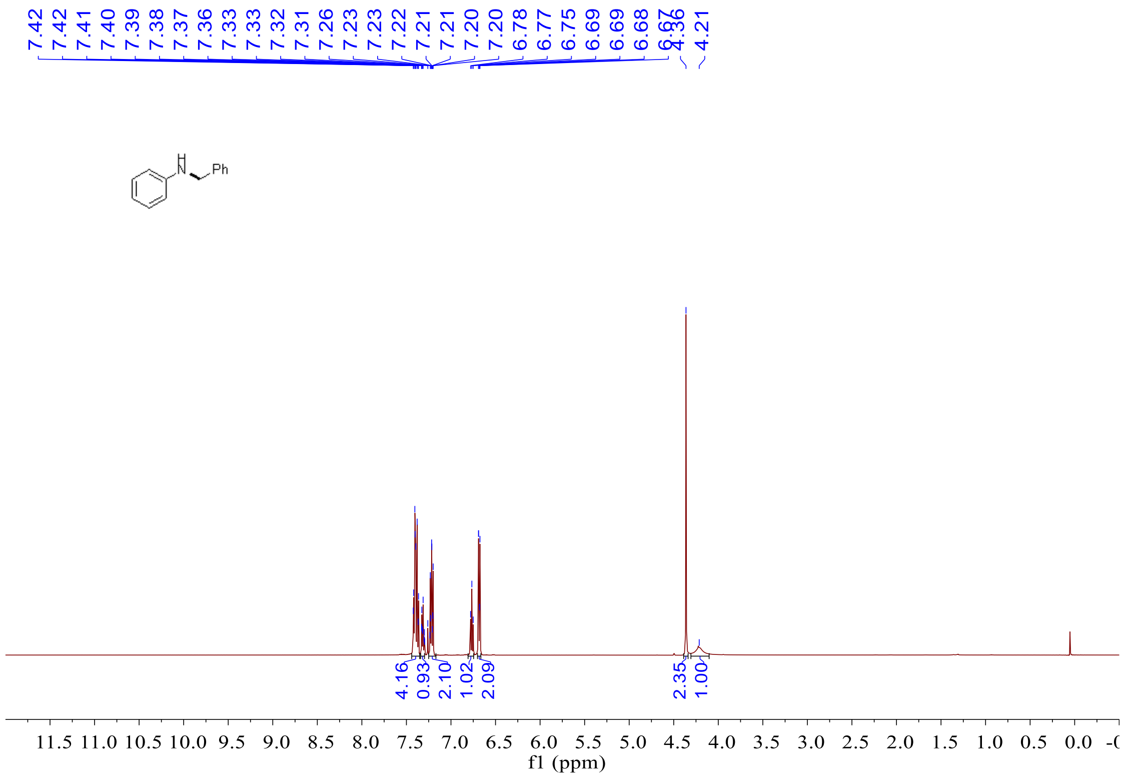

Supplementary Figure 197 ^{1}H NMR spectrum for compound **93**

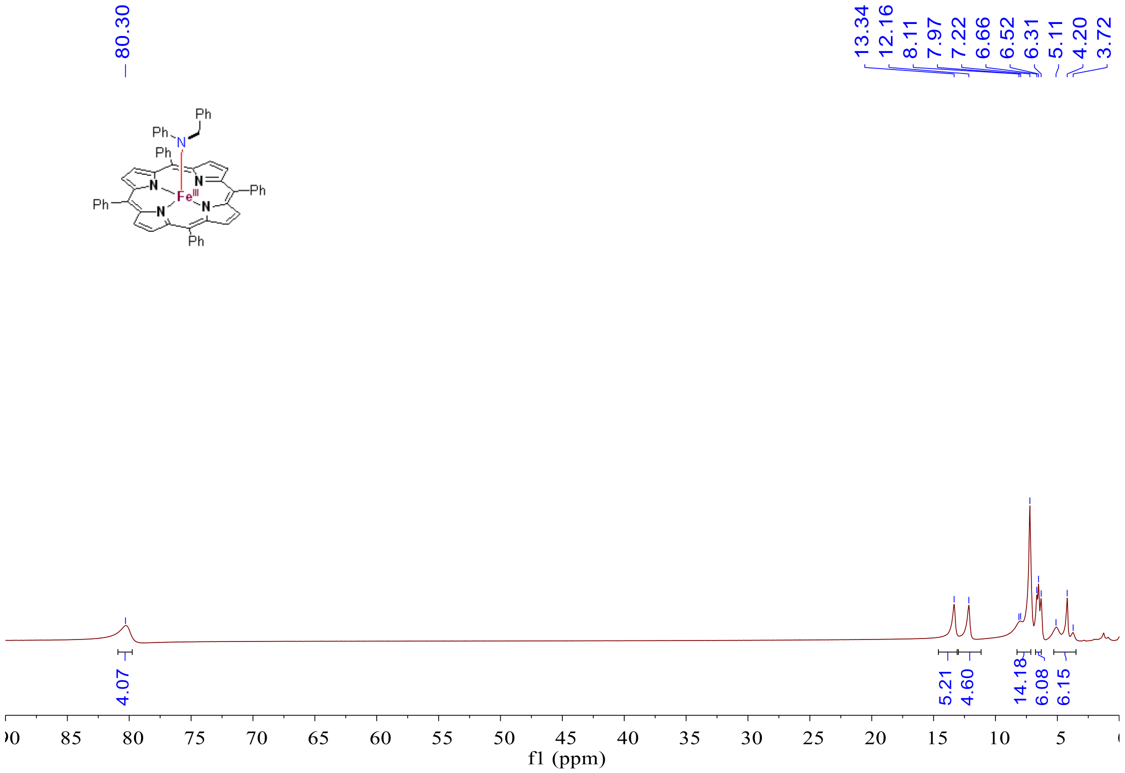

Supplementary Figure 198 ^{1}H NMR spectrum for compound **80**

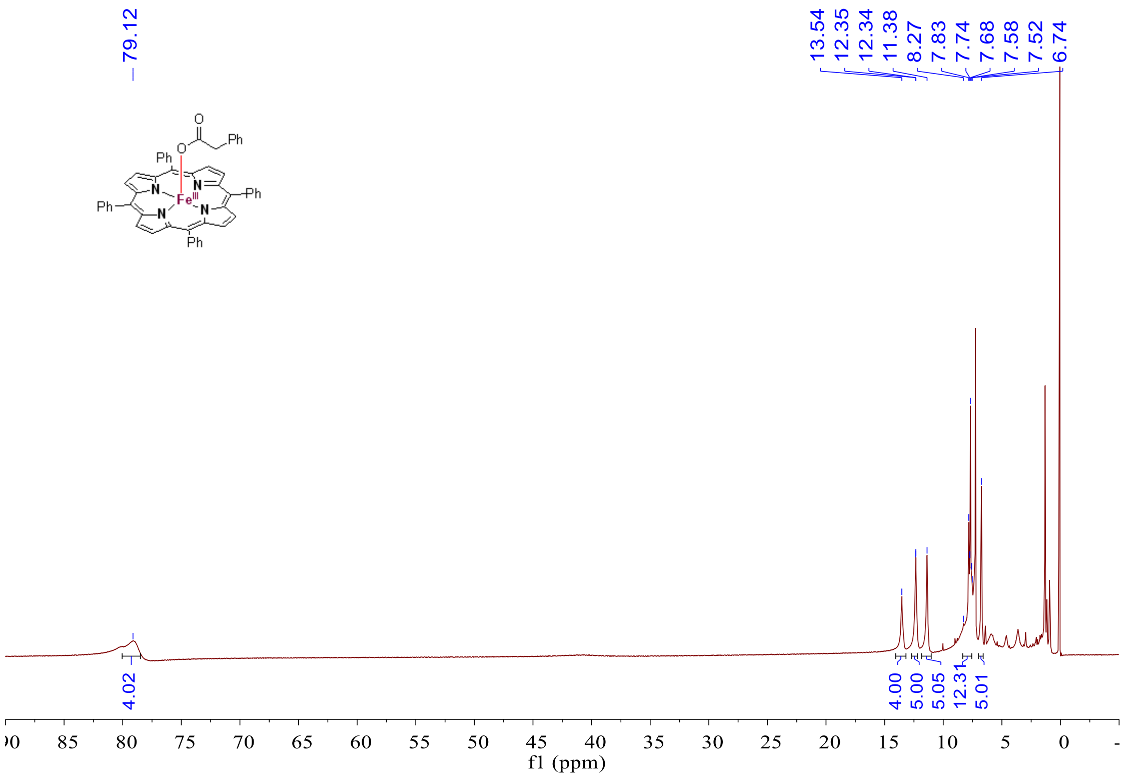

Supplementary Figure 199 ^{1}H NMR spectrum for compound **81**

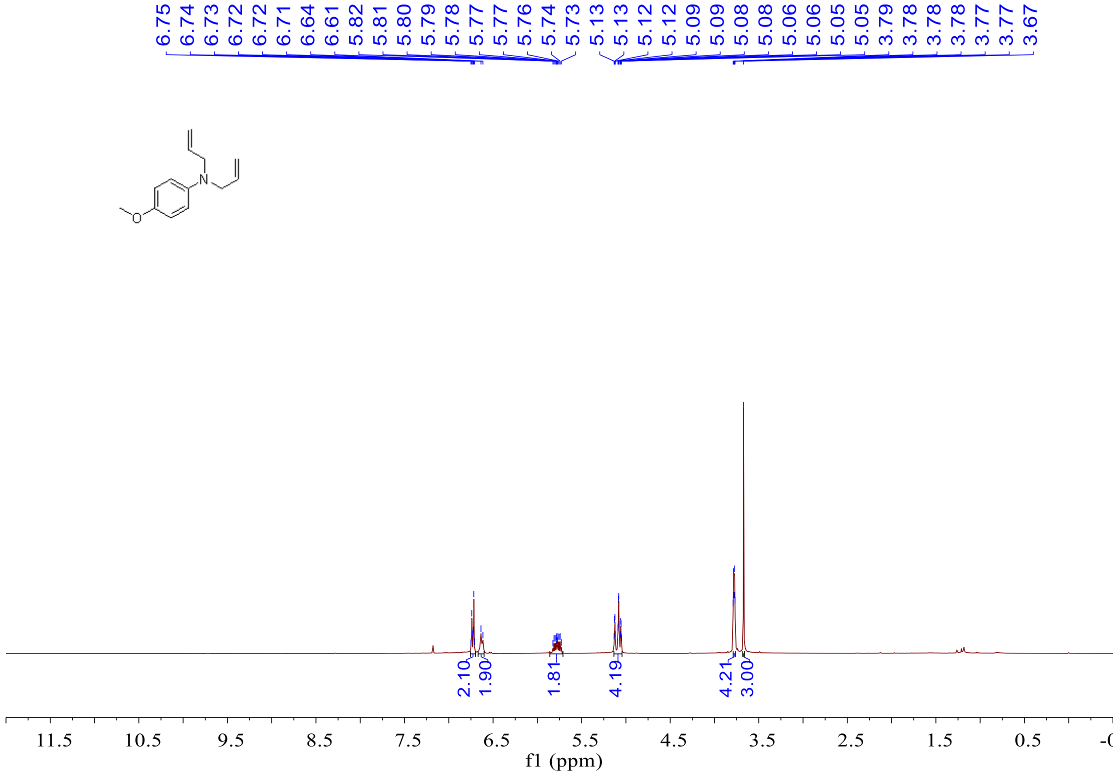

Supplementary Figure 200 ^{1}H NMR spectrum for compound **94**

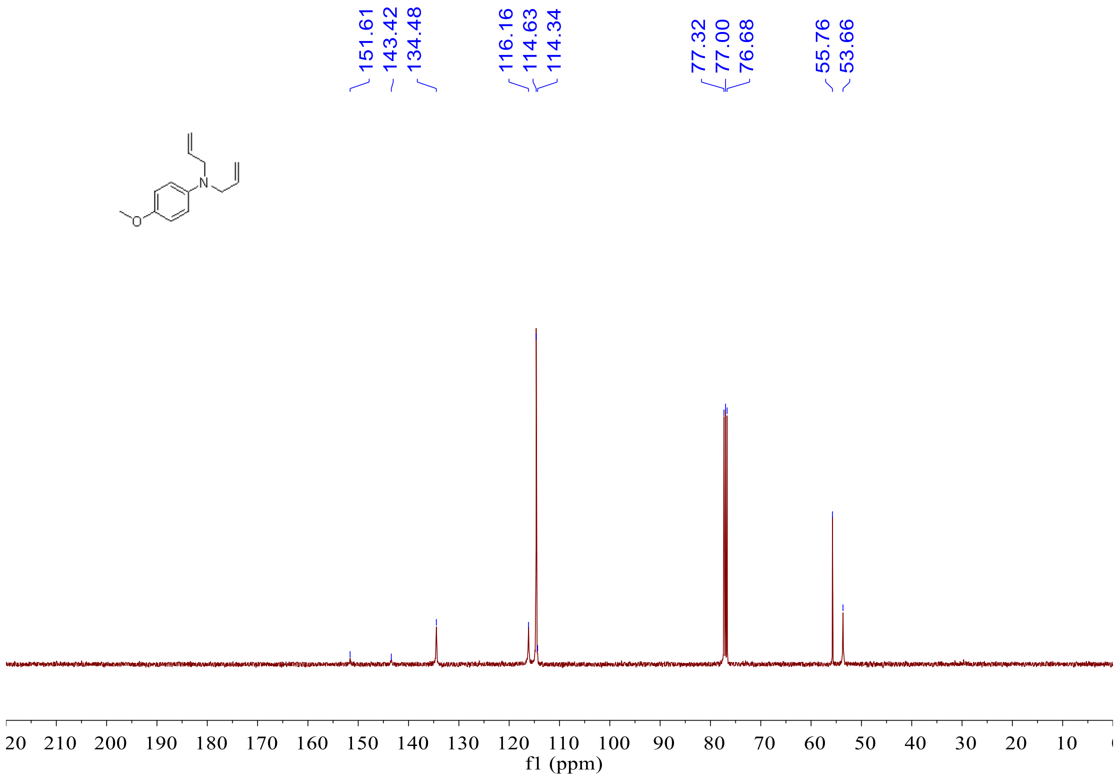

Supplementary Figure 201 ^{13}C NMR spectrum for compound **94**

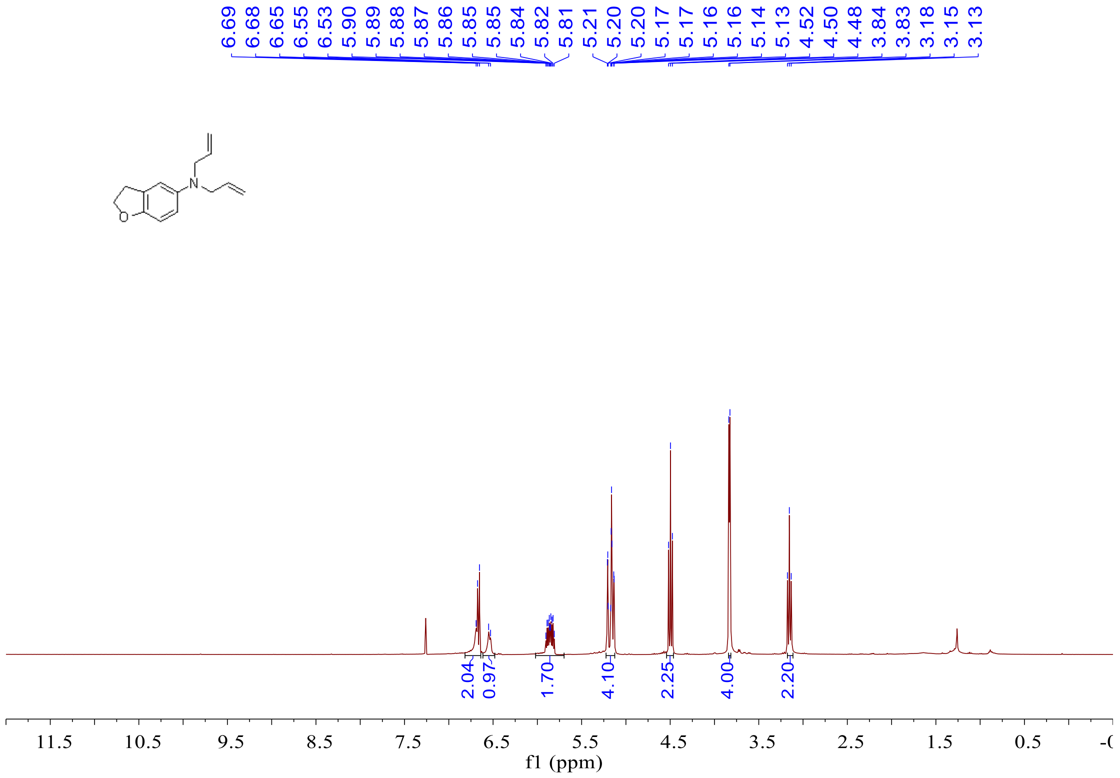

Supplementary Figure 202 ^{1}H NMR spectrum for compound **95**

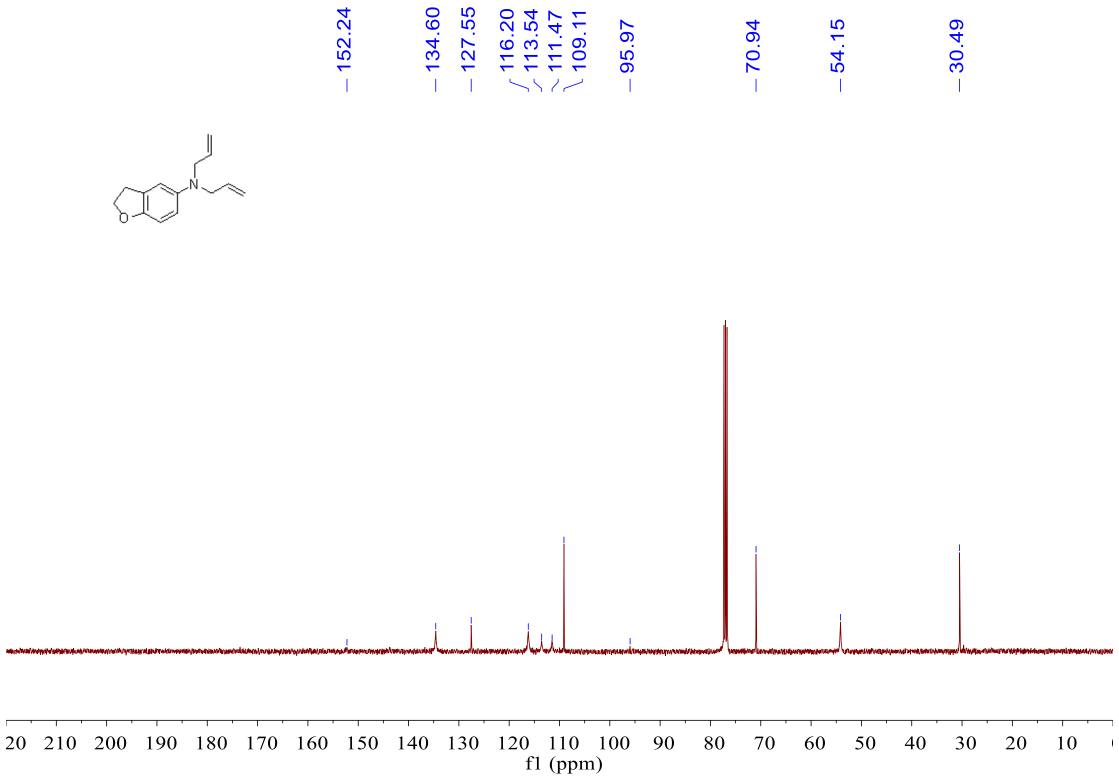

Supplementary Figure 203 ^{13}C NMR spectrum for compound **95**

Supplementary References

(1) Kucheryavy, P.; Lahanas, N.; Velasco, E.; Sun, C. J.; Lockard, J. V. Probing Framework-Restricted Metal Axial Ligation and Spin State Patterns in a Post-Synthetically Reduced Iron-Porphyrin-Based Metal-Organic Framework. *J. Phys. Chem. Lett*. **2016**, *7*, 1109-15.

(2) Jones, J. G.; McKnight, J.; Twigg, M. V. Replacement of imidazoles by azide at an iron (III) porphyrin center: part 1 replacement of N-methylimidazole in bis-(N-methylimidazole) tetraphenylporphyrinato iron(III). *J. Coord. Chem.* **2010**, *63*, 2766-2778.

(3) Pegis, M. L.; Martin, D. J.; Wise, C. F.; Brezny, A. C.; Johnson, S. I.; Johnson, L. E.; Kumar, N.; Raugei, S.; Mayer, J. M. Mechanism of Catalytic O2 Reduction by Iron Tetraphenylporphyrin. *J. Am. Chem. Soc.* **2019**, *141*, 8315-8326.

(4) Neerathilingam, N.; Bhargava Reddy, M.; Anandhan, R., Regioselective Synthesis of degrees Amides Using Visible-Light-Induced Photoredox-Catalyzed Nonaqueous Oxidative C-N Cleavage of N, N-Dibenzylanilines. *J. Org. Chem*. **2021**, *86*, 15117-15127.

(5) Phakhodee, W.; Wangngae, S.; Pattarawarapan, M. Metal-free amidation of carboxylic acids with tertiary amines. *RSC Advances.* **2016**, *6*, 60287-60290.

(6) Chen, X.; Chen, T.; Li, Q.; Zhou, Y.; Han, L. B.; Yin, S. F. Copper-catalyzed aerobic oxidative inert C-C and C-N bond cleavage: a new strategy for the synthesis of tertiary amides. *Chemistry*. **2014**, *20*, 12234-8.

(7) Huang, H.; Guo, W.; Wu, W.; Li, C. J.; Jiang, H., Copper-Catalyzed Oxidative C(sp^3)-H Functionalization for Facile Synthesis of 1,2,4-Triazoles and 1,3,5-Triazines from Amidines. *Org. Lett.* **2015**, *17*, 2894-7.

(8) Ling, X.; Xiong, Y.; Huang, R.; Zhang, X.; Zhang, S.; Chen, C., Synthesis of benzidine derivatives via $FeCl_3$.6H2O-promoted oxidative coupling of anilines. *J. Org. Chem.* **2013**, *78*, 5218-26.

(9) Dai, F.; Yang, Y.; Gu, J.; Fang, Z.; Yang, Z.; Liu, C.; He, W.; Zhu, N.; Lu, B.; Guo, K. Synthesis of Tertiary Aromatic Amides from Tertiary Benzylamines via TBAI/TBHP‐Mediated C‐N‐Bond Cleavage. *ChemistrySelect.* **2019**, *4*, 3500-3504.

(10) Xiangdong Wang, Chunqi Jia, Yadong Feng, Lianhui Wang, Xiuling Cui. One-Pot Synthesis of N-Alkyl Benzotriazoles via a Brønsted Acid-Catalyzed Three-Component

Reaction. *Adv. Synth. Catal.* **2018**, *360*, 374–378

(11) Wagner, A.; Han, W.; Mayer, P.; Ofial, A. R. Iron-Catalyzed Generation of α-Amino Nitriles from Tertiary Amines. *Advanced Synthesis & Catalysis.* **2013**, *355*, 3058-3070.

(12) Luo, M. J.; Li, Y.; Ouyang, X. H.; Li, J. H.; He, D. L. Electrochemical dehydrogenative cross-coupling of two anilines: facile synthesis of unsymmetrical biaryls. *Chem Commun (Camb).* **2020**, *56*, 2707-2710.

(13) Yu, M.; Tang, R.-Y.; Li, J.-H. Synthesis of 6,7-dihydro-5H-dibenzo[c,e]azepines and biaryls by palladium-catalyzed Ullmann reaction. *Tetrahedron.* **2009**, *65*, 3409-3416.

(14) Cismesia, M. A.; Yoon, T. P. Characterizing chain processes in visible light photoredox catalysis. *Chem. Sci*. **2015**, *6*, 5426-5434.
